# HT‐SuperSAGE of the gut tissue of a Vip3Aa‐resistant *Heliothis virescens* (Lepidoptera: Noctuidae) strain provides insights into the basis of resistance

**DOI:** 10.1111/1744-7917.12535

**Published:** 2017-12-01

**Authors:** Camilo Ayra‐Pardo, Maria E. Ochagavía, Ben Raymond, Asim Gulzar, Lianet Rodríguez‐Cabrera, Claudia Rodríguez de la Noval, Ivis Morán Bertot, Ryohei Terauchi, Kentaro Yoshida, Hideo Matsumura, Pilar Téllez Rodríguez, Daily Hernández Hernández, Orlando Borrás‐Hidalgo, Denis J. Wright

**Affiliations:** ^1^ Plant Division Centre for Genetic Engineering and Biotechnology (CIGB) Havana Cuba; ^2^ Department of Life Sciences, Faculty of Natural Sciences Imperial College London Berkshire UK; ^3^ Genetics and Genomics Research Group Iwate Biotechnology Research Center Kitakami Japan

**Keywords:** Bt resistance, *Heliothis virescens*, HT‐SuperSAGE, transcriptomics, Vip3Aa

## Abstract

Multitoxin Bt‐crops expressing insecticidal toxins with different modes of action, for example, Cry and Vip, are expected to improve resistance management in target pests. While Cry1A resistance has been relatively well characterized in some insect species, this is not the case for Vip3A, for which no mechanism of resistance has yet been identified. Here we applied HT‐SuperSAGE to analyze the transcriptome of the gut tissue of tobacco budworm *Heliothis virescens* (F.) laboratory‐selected for Vip3Aa resistance. From a total of 1 324 252 sequence reads, 5 895 126‐bp tags were obtained representing 17 751 nonsingleton unique transcripts (UniTags) from genetically similar Vip3Aa‐resistant (Vip–Sel) and susceptible control (Vip–Unsel) strains. Differential expression was significant (≥2.5 fold or ≤0.4; *P* < 0.05) for 1989 sequences (11.2% of total UniTags), where 420 represented overexpressed (OE) and 1569 underexpressed (UE) genes in Vip–Sel. BLASTN searches mapped 419 UniTags to *H. virescens* sequence contigs, of which, 416 (106 OE and 310 UE) were unambiguously annotated to proteins in NCBI nonredundant protein databases. Gene Ontology distributed 345 of annotated UniTags in 14 functional categories with metabolism (including serine‐type hydrolases) and translation/ribosome biogenesis being the most prevalent. A UniTag homologous to a particular member of the REsponse to PAThogen (REPAT) family was found among most overexpressed, while UniTags related to the putative Vip3Aa‐binding ribosomal protein S2 (RpS2) were underexpressed. qRT‐PCR of a subset of UniTags validated the HT‐SuperSAGE data. This study is the first providing lepidopteran gut transcriptome associated with Vip3Aa resistance and a foundation for future attempts to elucidate the resistance mechanism.

## Introduction

Vip (from vegetative insecticidal proteins) proteins comprises a group of pore‐forming toxins produced and secreted by *Bacillus thuringiensis* (Bt) during vegetative growth of the bacterium (Estruch *et al*., 1996). So far, identified Vip proteins have been divided into four families according to their amino acid identity: Vip1 and Vip2 act as binary toxins against some members of the Coleoptera and Hemiptera orders, Vip3 proteins are toxic to a wide variety of members of the Lepidoptera, and the recently reported Vip4 family have no known target organisms (Chakroun *et al*., 2016a,b). In addition, Vip toxins have no sequence homology to the sporulation‐associated Bt Cry δ‐endotoxins and, at least for Vip3A, binding sites in the gut of target lepidopteran larvae have shown to differ from that of lepidopteran‐specific Cry1‐class toxins (Lee *et al*., 2003; Kurtz, 2010; Liu *et al*., 2011; Abdelkefi‐Mesrati *et al*., 2011). This independent mode of action has led to the commercial release of pyramided transgenic Bt‐cotton (Kurtz *et al*., 2007) and Bt‐maize (Burkness *et al*., 2010), containing *cry*‐class genes combined with *vip3A* to minimize the risk of field‐evolved resistance.

Resistance to Cry toxins has been relatively well characterized in some insect species. Reduced binding of Cry toxins is the most commonly reported type of resistance mechanism in insects (Pigott & Ellar, 2007; Jurat‐Fuentes & Jackson, 2012; Adang *et al*., 2014). Binding of Cry toxins has shown to be affected by altered gene expression (Herrero *et al*., 2005; Guo *et al*., 2011), midgut shedding (Valaitis, 2008), or genetic mutations in putative receptors (Gahan *et al*., 2001; Gahan *et al*., 2010; Baxter *et al*., 2011; Atsumi *et al*., 2012). Also, alteration of gut proteases has been found an important factor of resistance for some insect species through compromising Bt Cry toxin activation or stability (Oppert *et al*., 1994, 1997; Forcada *et al*., 1996; Sayyed *et al*., 2001; Khajuria *et al*., 2009; Rodríguez‐Cabrera *et al*., 2010; Liu *et al*., 2014).

No mechanism of resistance to Vip3A has been identified. Only two insect proteins that interact with Vip3A have been recognized so far using the yeast two‐hybrid system. The first was a 48‐kDa glycoprotein from *Agrotis ipsilon* with homology to tenascins, which could be associated with apoptotic processes (Estruch & Yu, 2001). The second is the ribosomal protein S2 (RpS2) from *Spodoptera litura* identified in Sf21 cells (Singh *et al*., 2010). The specific interaction between Vip3A and RpS2 was validated through *in vitro* protein binding studies. Further exploration of RpS2 function by RNA interference‐mediated knockdown of gene expression both in transfected Sf21 cells and in double‐stranded RNA‐injected *S. litura* larvae resulted in a reduced toxicity of the Vip3A protein (Singh *et al*., 2010).

Barkhade and Thakare (2010) attributed resistance in a Vip3A‐selected *S. litura* strain to a significant decrease in the enzymatic activity of all major types of gut proteases, that is, azocasein, trypsin and chymotrypsin, and the presence of fewer protease isoforms in the gut fluids of resistant compared to the susceptible larvae. In fact, the protoxin‐processing step by gut juice proteases has been described to be determinant for the insecticidal potency of Vip3Aa against insects of *Spodoptera* sp. (Chakroun *et al*., 2012). Recently, two different transcriptional studies on larval gut from *Spodoptera exigua* (Bel *et al*., 2013) and *S. litura* (Song *et al*., 2016) have shown major alterations in transcripts encoding for serine‐proteases after feeding insects with a sublethal dose of the Vip3Aa toxin.

In our laboratory, 12 generations of selection with Vip3Aa of a field‐derived population of *Heliothis virescens* produced a strain (Vip–Sel) with more than 2040 fold resistance compared to the genetically similar unselected (Vip–Unsel) subpopulation (Pickett, 2009; Pickett *et al*., 2017). Genetic characterization of Vip3Aa resistance in Vip–Sel combined with bioassays indicated resistance was due to more than one locus and appeared to be relatively unstable after 13 generations without exposure to the toxin. Inheritance of resistance showed strong paternal influence and ranged from almost completely recessive (mean *h* = 0.04 if the resistant parental was female) to incompletely dominant (mean *h* = 0.53 if the resistant parental was male) (Pickett, 2009; Pickett *et al*., 2017). Further characterization of the Vip–Sel subpopulation revealed a temperature‐dependent fitness cost associated with reduced mating success, fecundity, and fertility in resistant insects (Gulzar *et al*., 2012).

The use of “omics” to study Bt resistance has taken the investigation to a new dimension allowing a much broader comparison between susceptible and resistant populations, particularly, when little or no specific details concerning the resistance mechanism exists. For example, transcriptomic comparisons between Bt‐resistant and susceptible strains have revealed alterations in mRNA abundance of a large number of genes representing a multitude of functional categories (Hernández‐Martínez *et al*., 2010; Guo *et al*., 2012; Lei *et al*., 2014; Ayra‐Pardo *et al*., 2015). It is expected many of the differentially expressed genes, most of them not previously implicated in Bt toxicity, are due to the influence of differences in genetic background between populations rather than to resistance. However, recent studies have assigned a role in Bt resistance to some intracellular genes with altered regulation since their suppression in resistant populations significantly increased the susceptibility to Bt (Guo *et al*., 2011; Ayra‐Pardo *et al*., 2015). Consequently, a new potential mechanism for Bt resistance has been proposed where the loss of receptor function could be an indirect effect of an intracellular response that modifies cell's/organism's physiology in order to deal with toxin challenge (Crickmore, 2016). The comparison of transcriptional profiling in the gut tissue between resistance and susceptible insects would allow for a better understanding of toxin adaptation processes in the former and the identification of plausible candidates for gene functional analysis to elucidate the bases of resistance.

In order to gain insight into the molecular basis of Vip3Aa resistance in the Vip–Sel strain of *H. virescens*, the transcriptional profiling of the larval gut tissue was generated and compared to that of the susceptible Vip–Unsel control using HT‐SuperSAGE (Matsumara *et al*., 2010). A subset of genes chosen among those showing the highest differential expression or with any significance to the Vip3A toxic pathway in insects was used to validate the HT‐SuperSAGE results by quantitative RT‐PCR. To our knowledge, this work is the first example of successful application of HT‐SuperSAGE to a nonmodel lepidopteran insect and a key pest species.

## Materials and methods

### Insects

The process of obtaining the *H. virescens* Vip–Sel and Vip–Unsel subpopulations, resistant and susceptible to the Vip3Aa protoxin respectively, was previously described (Pickett, 2009; Gulzar *et al*., 2012; Pickett *et al*., 2017). Briefly, a field‐derived population of *H. virescens* (WF06) was divided into two subpopulations at the larval stage of the second generation of laboratory culture. One subpopulation was left unselected (Vip–Unsel) and the other selected with Vip3Aa protoxin (Vip–Sel) at the first instar larval stage from the second generation onwards. Only larvae that had molted to at least second instar after 7 d exposure to Vip3Aa were selected to give rise to adults that will become the parents to produce the next generation. The number of larvae selected *per* generation ranged from approximately 600–1200; with the exception of the initial selection when the number of larvae (330) available was low. Both subpopulations were maintained in Imperial College London, Silwood Park and reared at 25 ± 2 °C, 70% ± 5% RH with 16 : 8 (light : dark) cycle on artificial diet.

### Vip3Aa protoxin and bioassays

Recombinant Vip3Aa protoxin, produced in *Escherichia coli*, was supplied as a lyophilized powder by Syngenta (Research Triangle Park, NC, USA); this is the same protoxin source used before for obtaining the Vip–Sel subpopulation (Pickett, 2009; Gulzar *et al*., 2012; Pickett *et al*., 2017).

Vip–Sel insects used for transcriptomic experiments were maintained unselected for six generations to avoid maternal effects. Since resistance in Vip–Sel has been found unstable in the absence of exposure to the toxin due to fitness cost issues (Pickett, 2009; Gulzar *et al*., 2012; Pickett *et al*., 2017), the Vip3Aa resistance level on insect generation prior to construct the SuperSAGE libraries was determined in duplicated feeding bioassays on artificial diet using the diet incorporation method. A stock suspension of Vip3Aa prepared in distilled water (1 mg/mL) was used to generate twofold serial dilutions of the toxin; distilled water was used as control. Assays scored the 5‐d mortality of 24 neonate larvae per dose. Estimates of LC_50_, concentration that causes death in 50% of the population expressed as μg of Vip3Aa per mL, and their 95% fiducial limits (FL) were calculated in R (http://www.r-project.org). Differences in LC_50_s were significant (*P* < 0.05) when their respective 95% FL did not overlap. Resistance ratio was expressed as the ratio of the LC_50_ of Vip–Sel to that of the susceptible Vip–Unsel.

### Total RNA extraction and construction of SuperSAGE libraries

The gut tissue was dissected from ice‐anesthetized third‐instar larvae of Vip–Sel and Vip–Unsel subpopulations (25 larvae each) that were reared on diet not contaminated with Vip3Aa by cutting off the hind‐body between the last two pairs of prolegs and pooled (five pools each containing five guts *per* insect strain). Then, total RNA was extracted from tissue pools with the total RNA isolation system (Promega, Madison, WI, USA). RNA concentration was determined at 260 nm in a GeneQuant (Amersham Pharmacia, Amersham, UK). RNA pools of Vip–Sel and Vip–Unsel were prepared using above five RNA preparations from each strain.

The SuprSAGE libraries HvR_GCCT and HvS_GCAC were constructed for Vip–Sel and Vip–Unsel, respectively, according to the procedure described by Matsumura *et al*. (2010). First, double‐stranded cDNA was synthesized using Vip–Sel and Vip–Unsel RNA pools and a biotinylated adapter‐oligo dT primer (5′‐bio‐ctgatctagaggtaccggatcccagcag(T)_17_‐3′). Double‐stranded cDNA was digested twice with the anchoring enzyme NlaIII and resulting fragments bound to streptavidin‐coated beads (Dynabeads streptavidin M‐270), nonbiotinylated cDNA fragments were removed by washing. Next step was the ligation of Illumina Adapter‐2 (annealed, oligonucleotide A: 5′‐caagcagaagacggcatacgatctaacgatgtacgcagcagcatg‐3′ and oligonucleotide B: 5′‐ctgctgcgtacatcgttagatcgtatgccgtcttctgcttg‐amino‐3′) to dscDNA fragments on the beads carrying the 4‐bp overhang (5′‐CATG‐3′) and after washing digested with EcoP15I (5′‐CAGCAG‐3′). EcoP15I‐digested and released fragments (adapter‐2‐tags) were ligated to Illumina adapters‐1 (annealed, oligonucleotide A: 5′‐acaggttcagagttctacagtccgacgatcxxxx‐3′ and oligonucleotide B: 5′‐nnxxxxgatcgtcggactgtagaactctgaacctgt‐amino‐3′), carrying the sequencing primer and a defined xxxx index sequence for sample identification, that is, GCCT for Vip–Sel and GCAC for Vip–Unsel. Tags sandwiched between two Illumina adapters were amplified by PCR using Phusion High polymerase (New England Biolabs Inc. Ipswich, MA, USA) and GEX primers (5′‐aatgatacggcgaccaccgacaggttcagagttctacagtccga‐3′ and 5′‐caagcagaagacggcatacga‐3′). The PCR reaction conditions were 98 °C for 1 min, 10 cycles at 98 °C for 30 s, and 60 °C for 30 s. Eight tubes from this PCR amplification (each 15 μL) were pooled and the PCR products were concentrated using a MinElute reaction purification kit (Qiagen GmbH, Hilden, Germany) and analyzed on 8% nondenaturing polyacrylamide gels. After staining with GelRed (Biotium, CA, USA), a band at 123–125 base pairs (bp) was cut from the gel and the DNA purified from the gel pieces. PCR products from each sample were analyzed on an Agilent Bioanalyzer 2100 (Agilent Technologies, Santa Clara, CA, USA). Equal concentrations of purified PCR products from the samples were mixed and sequenced with an Illumina Genome Analyzer II. Sequencing reactions used the GEX (DpnII) primer according to the manufacturer's instructions.

### Identification of differentially expressed tags and functional annotation

Sorting of sequence reads based on index sequences (GCCT and GCAC for HvR_GCCT and HvS_GCAC, respectively), removing incomplete and low‐quality tags and the subsequent extraction of unique sequences from reads was conducted using custom Perl scripts (Matsumura *et al*., 2011). A unique sequence was considered a singleton and removed if it was detected only once in the combined libraries. Only nonsingletons, referred as UniTags throughout the manuscript, were considered for further analysis. Statistically significant changes in tag copy number between the HvR_GCCT and HvS_GCAC libraries were analyzed by calculating a *P* value according to Audic and Claverie (1997). A tag was considered significant if *P* < 0.05.

Libraries were normalized using a method described in Gilardoni *et al*. (2010), where a normalization factor (NF) was arbitrarily designated as a value that is below the lowest “total number of tags” obtained for a library between the samples HvR_GCCT and HvS_GCAC. Then, the normalized value for each tag was calculated using the formula: (*x*/“total number of tags” of the library) × NF, where *x* is the number of copies of a given tag. Fold‐Change (FC) was calculated by dividing the number of tags in the normalized Vip–Sel (HvR_GCCT) library by the number of tags in the normalized Vip–Unsel (HvS_GCAC) library (Vip–Sel vs. Vip–Unsel). Tags absent in one of the libraries (Tag count = 0) were set to 1 for calculation.

BLASTN homology searches to a total of 63 648 sequence contigs of *H. virescens* kindly provided by Dr. Omaththage P. Perera (Perera *et al*., 2015) were carried out with the UniTag sequences. The task parameter was set to “blastn‐short” to guarantee optimal BLAST functioning for short sequences searches. Low‐complexity regions were rejected, whereas gap costs were set to 5–2, according to NCBI BLAST standard setting. A perfect 26 nucleotides (nt) match was required for sequence annotation; UniTags that matched to less than 26 nt were annotated as “no hit.” Annotations of the unigenes were performed using Blast2GO (Conesa *et al*., 2005). Hits scores <10^−4^ were used for Gene Ontology determination based on nonredundant GenBank and UniProtKB/TrEMBL protein databases.

### 3′‐RACE and qRT‐PCR experiments

In order to validate the HT‐SuperSAGE data, 10 UniTags were chosen for measurement of transcript abundance in larval guts of Vip–Sel and Vip–Unsel strains by qRT‐PCR. First, longer cDNA fragments were recovered by 3′‐RACE (Rapid Amplification of cDNA Ends) method and sequences compared against those in *H. virescens* contigs database (Perera *et al*., 2015) using the BLASTN algorithm.

For 3′‐RACE, total RNA extraction and preparation of RNA pools were carried out as described above on new dissected guts. Single‐stranded cDNA (cDNAss) was synthesized by reverse transcription with the SuperScript II Reverse Transcriptase from 1 μg of total RNA using an ADAPTER‐oligo(dT17), where ADAPTER means a sequence 5′‐AAGCAGTGGTATCAACGCAGAGTAC‐3′ added to the 5′‐end of the oligo(dT17), following the manufacturers’ directions (Invitrogen, Waltham, MA, USA). A primer oligonucleotide complementary to the ADAPTER–primer sequence was used for PCR in combination with the 26‐bp oligonucleotide corresponding to each of the 10 SuperSAGE tag sequences. For cloning the 3′‐RACE products, each fragment was purified using the PCR Clean‐Up System (Promega), ligated into the pGEM‐T Easy vector (Promega) and the resultant recombinant plasmids DNA transformed into high‐efficiency JM109 *Escherichia coli* competent cells (Promega). Recombinant colonies were randomly picked and cultured in Luria–Bertani medium containing ampicillin at 100 mg/L, followed by plasmid DNA extraction using the Wizard Plus SV Minipreps DNA Purification System (Promega). Insert‐bearing plasmids for each 3′‐RACE fragment were DNA sequenced on both strands on an ABI prism multicolor fluorescence‐based DNA analysis system (Applied Biosystems, Foster City, CA, USA), using the Taq Dye‐Deoxy Terminator Cycle Sequencing kit from the same manufacturer.

For qRT‐PCR experiments, Vip–Sel and Vip–Unsel cDNAss templates were synthesized using total RNA pools prepared as above on new dissected guts and an oligo(dT17) primer with the aid of SuperScript reverse transcriptase II (Invitrogen). The reactions were carried out with the Rotor‐Gene SYBR Green PCR kit (Qiagen GmbH, Hilden, Germany) in a Rotor‐Gene 3000 real‐time cycler (Corbett Research, Sydney, Australia). Primers annealing on the *H. virescens* contig sequence matching our selected UniTags (Table S1) and amplifying a 223 bp fragment of the *H. virescens β‐actin* gene (Genbank Acc. No. AF368030) as internal reference for transcript normalization, were designed with Primer3 software (Untergasser *et al*., 2012). The real‐time amplification and analysis was performed in duplicates in PCR reactions of 25‐μL final volume containing each primer (0.3‐μmol/L final concentration) and 12.5 ng of cDNAss as template. A dissociation curve and negative control (cDNA reaction without reverse transcriptase enzyme) were used to ensure primer specificity, that is, a single product amplified with each primer pair in every sample, and lack of contamination respectively. Amplification efficiencies of both target and reference genes were determined with the aid of standard curves generated by serial dilutions of corresponding cDNAs. Since the PCR efficiencies of the primer sets were found to be essentially equivalent for all targeted *H. virescens* sequences and for the *β‐actin* reference gene (Table S1), a single reference gene was sufficient for this study. In all cases, the relative transcript levels were expressed as “Mean Normalized Expression” data using Q‐Gene software (http://www.gene-quantification.de/qgene; Muller *et al*., 2002).

### Accession number

The tag profiling data generated in this study was deposited in the NCBI's Gene Expression Omnibus (GEO) public domain (Edgar *et al*., 2002) under the accession (GSE72228) (http://www.ncbi.nlm.nih.gov/geo/query/acc.cgi?acc=GSE72228). The GenBank accession numbers for 3′‐RACE products corresponding to extended UniTag sequences are: Tag_61 (KX230117); Tag_393 (KX230118); Tag_310 (KX230119); Tag_888 (KR871307); Tag_403 (KX247446); Tag_267 (KX247447); Tag_4722 (KX230120); Tag_1705 (KX230121); Tag_4245 (KX230122); and Tag_220 (KR871310).

## Results

### Generation of HT‐SuperSAGE libraries from gut tissue of Vip3Aa‐selected and unselected H. virescens larvae and annotation of UniTags to public databases

Before constructing the HT‐SuperSAGE libraries, the response to Vip3Aa intoxication of neonate Vip–Sel and control Vip–Unsel larvae was determined and the resistance ratio calculated. In bioassays Vip3Aa protoxin was 234 fold less toxic to Vip–Sel (LC_50_ = 103 μg/mL; 95% fiducial limits 46–226; Slope ± SE = 0.89 ± 0.15) than to Vip–Unsel (LC_50_ = 0.44 μg/mL; 95% fiducial limits 0.30–0.64; Slope ± SE = 0.21 ± 0.21).

**Figure 1:**
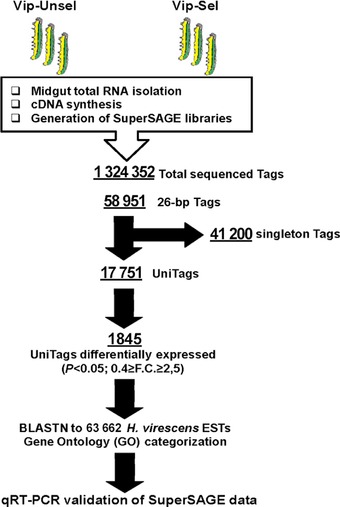
Schematic representation of the workflow followed in this study to analyze the gut transcriptomic profile in a Vip3Aa‐resistant strain of *H. virescens*. Features of HT‐SuperSAGE results are shown underlined.

The HT‐SuperSAGE libraries, HvR_GCCT and HvS_GCAC, were generated from the gut tissue of Vip–Sel larvae and Vip–Unsel larvae respectively, and used to compare the major transcriptional changes potentially associated with Vip3Aa resistance (Fig. 1). The total number of sequence reads obtained after sequencing the libraries and removing incomplete sequences, that is, without index sequences and/or anchoring enzyme sites was 1 324 252, classified according to the index sequence GCCT or GCAC in 1 056 080 reads from the HvR_GCCT library and 268 172 from the HvS_GCAC. Further analysis removed tags with a length different to 26 bp that are frequently found due to the variable distance between the recognition and cleavage sites of EcoP15I, tags containing undetermined bases (“N”), long homopolymers (>10 bp) or excessive numbers of low‐quality positions (>2 positions with quality scores <10). In total, a number of 58 951 high‐quality 26‐bp tags representing unique sequences were obtained, comprising 3 965 426‐bp tags from the HvR_GCCT (Vip–Sel) library and 19 297 from the HvS_GCAC (Vip–Unsel). These 58 951 unique sequences comprised 17 751 nonsingletons UniTags that represented 11 668 for Vip–Sel and 6083 for Vip–Unsel.

**Figure 2:**
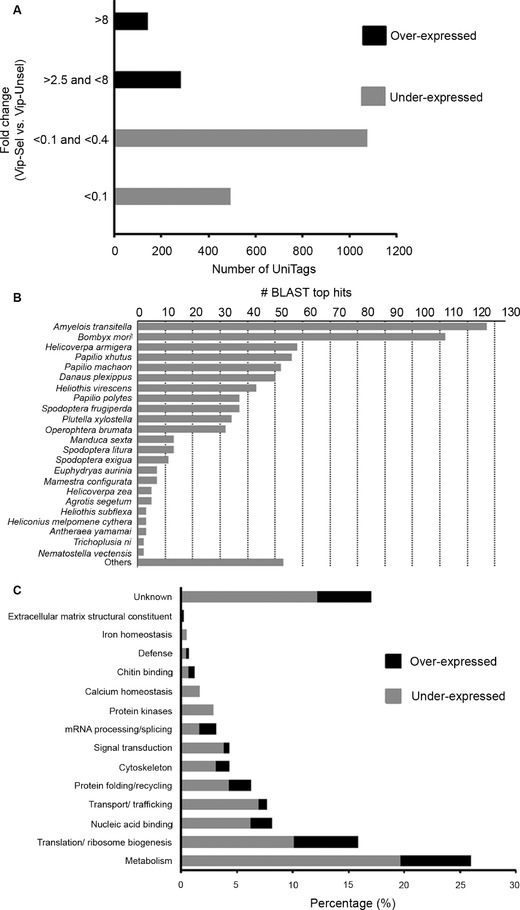
Analysis of differentially expressed UniTags. (A) Fold change (Vip–Sel vs. Vip–UnSel) distribution of the 1989 differentially expressed UniTags. (B) Top hit species distribution of *Heliothis virescens* contig sequences that matched to differentially expressed UniTags with a significant BLASTN score (*E*‐value < 1E‐04). (C) Distribution of 416 annotated UniTags in Gene Ontologiy (GO) categories based on molecular function and biological process.

Although small changes in expression levels may have biological significance, for this study we focused primarily on genes with fold change (FC) values ≥ 2.5 or ≤ 0.4. For FC calculation, a NF of 268 000 was used that is less than the “total number of tags” of the library with the lowest value between HvR_GCCT and HvS_GCAC, that is, 268 172 for the HvS_GCAC library. Based on calculated FC and *P* values and using a 95% confidence level, 1989 UniTags (11.2% of the total of 17 751) were found differentially expressed in Vip–Sel compared to Vip–Unsel: 420 had FC values ≥2.5 and were considered overexpressed (OE) unigenes, whereas, 1569 had FC values ≤0.4 and represented underexpressed (UE) unigenes. Notably, FC for most of the differentially expressed UniTags ranged between 2.5 and 8 for OE (66%) and between 0.1 and 0.4 for UE (68%) (Fig. 2A).

Sequence annotation was carried out by the BLASTN search algorithm of the 1989 UniTags against *H. virescens* sequence contigs (Perera *et al*., 2015). Unexpectedly, only a limited number of 419 of these UniTags (109 OE and 310 UE that together account for 21.06% of all regulated genes) were mapped with a perfect 26 nt match (*E*‐value ≤ 10^−4^) to a *H. virescens* sequence contig. One explanation for this observation could be associated to an insufficient depth of sequencing of previously published *H. virescens* transcriptome (Perera *et al*., 2015). However, potential mismatch in “nn” during the ligation of sequencing adapter (Adapter I) to the EcoP15I‐digested adapter‐2‐tag in the SuperSAGE library could also be relevant. Interestingly, not all the tags were located at the end of the published *H. virescens* contigs (Tables S2 and S3) as it was expected considering tag fragments are isolated from the NlaIII site closest to the poly‐A tail of cDNA (Matsumura *et al*., 2010) (see Discussion section).

### Gene ontology (GO) categorization of differentially expressed UniTags

To obtain molecular function and/or biological process categories of the 419 differentially expressed UniTags, gene ontology (GO) annotation was performed by BLASTX (using the corresponding annotated *H. virescens* sequence contigs as queries) against the nonredundant GenBank and UniProtKB/TrEMBL protein databases. A total of 416 UniTags (106 OE and 310 UE) hits with a score ≤10^−4^ to an amino‐acid sequence entry and were unambiguously annotated to the NCBI nonredundant protein databases. Most sequences showed significant matches to orthologs in *Amyelois transitella* (29.53% of annotated contigs) and *Bombyx mori* (26% of annotated contigs) (Fig. 2B). GO annotations (biological processes and/or molecular function) could be assigned to 345 of the 416 UniTags, distributed in 14 functional categories, with the remaining entries corresponding to proteins of unknown function (Fig. 2C).

Tables 1 and 2 present a summary of HT‐SuperSAGE results comprising the 40 most overexpressed and the 40 most underexpressed Vip–Sel unigenes (also, Tables S2 and S3 contain a complete list of OE and UE UniTags, their sequences, copy numbers, fold change, annotations to *H. virescens* sequence contigs database, and GO categorization).

**Table 1:** Top 40 from the list of 113 larval gut unigenes identified as overexpressed in the Vip–Sel strain by HT‐SuperSAGE

Tag code	Tag sequence†	FC‡	Hv_Contig_	Protein description	*E*‐value
Tag_61	TTGCTGGCTGCTGCAGCCACCG	74.86	8456	AFH57159|REPAT39 *Spodoptera exigua*	7E–30
Tag_55	AGAATGCTAAAACTGGGTGGTG	67.36	22902	ABH10141|HMG176 *Helicoverpa armigera*	7E–53
Tag_435	CCGCGCGGCGCGGCGCGGCAGG	33.52	11935	XP_014367764|Zinc finger protein 598 *Papilio machaon*	0E+00
Tag_544	GAGGCGGACGGCGCGGGCGCCG	24.63	35272	XP_011559079|Uncharacterized protein LOC105389633 *Plutella xylostella*	2E–30
Tag_550	GCCGCGCCCGCGCACTGCGGCG	24.12	368	XP_014362832|Cytoplasmic dynein 1 intermediate chain isoform X6 *Papilio machaon*	0E+00
Tag_326	CGCGTGGACGCGCTGCCCGCGC	24.00	7535	XP_013135438|Presenilin homolog *Papilio polytes*	8E–169
Tag_335	TCGCGCGGCCCGCGCCACCGCT	23.23	2277	XP_013188918|Protein virilizer *Amyelois transitella*	0E+00
Tag_87	AGCCCCCTCCTGCCCGACACGT	21.99	16164	XP_013135392|Leukocyte surface antigen CD53‐like isoform X1 *Papilio polytes*	7E–73
Tag_18	CGCACGGCACGCAGAAGGGTGA	20.85	31364	CCF46246|Hypothetical protein CH063_15059, partial *Colletotrichum higginsianum*	2E–14
Tag_125	TGGCCGGGCTCGCATCGCAGTT	20.68	5434	XP_012546736|P3 protein‐like *Bombyx mori*	1E–39
Tag_606	GAACGCGAGGCTGAGGACGCGT	20.57	9943	XP_013141160|Tetraspanin‐15‐like *Papilio polytes*	1E–39
Tag_393	GCAGCCGCCGTCGTGTCAGGCA	19.43	28542	NP_001298510|40S ribosomal protein S20 *Papilio polytes*	9E–81
Tag_230	GTCGCTTCCAGACGCCGGCTGA	18.35	7237	AAL62468|Ribosomal protein L3 *Spodoptera frugiperda*	0E+00
Tag_653	TCGGCGACCTCGGAAATGTCCT	18.03	17230	ACY78421|Diapause bioclock protein‐like protein *Helicoverpa armigera*	7E–128
Tag_3	GCAGCCTAGCTTCGCCCCGCGT	17.55	40219	ABH10141|HMG176 *Helicoverpa armigera*	2E–22
Tag_683	GTTGGCCCGCGAGTCCTGAAGG	16.51	20199	Q963B7|60S ribosomal protein L9 *Spodoptera frugiperda*	3E–130
Tag_442	TATCGTGTGGCAGTCGGCCGCA	16.25	949	XP_014363077|Phosphatidylinositol transfer protein alpha isoform *Papilio machaon*	9E–166
Tag_451	GAGCGGCTCGCGCTCTCCAACG	15.74	16650	ADK55517.2|Heat shock protein 90 cognate *Spodoptera litura*	4E–130
Tag_268	TACGCGGCGCTGTGGCCCTTCC	14.85	25956	EHJ71292|Hypothetical protein KGM_01454 *Danaus plexippus*	8E–25
Tag_492	CAAGCTGCTGCCCCATTCTCCT	13.97	7038	CAA06419|Carboxypeptidase A *Helicoverpa armigera*	0E+00
Tag_163	ACACGGGCGTGCGGAGGAGCAT	13.55	35527	O02443|Larval cuticle protein 1 *Helicoverpa armígera*	4E–25
Tag_310	AGCTGGATCAACAGACATCTGT	13.08	42719	ACR15971.2|Serine protease 37 *Mamestra configurata*	1E–12
Tag_527	AAACTTGAAGGTTGGGATGGCG	12.82	32968	ACB54948|Fatty acid‐binding protein 1 *Helicoverpa armigera*	6E–70
Tag_799	GGAGGGTGTCACAGGTTCCTCT	12.44	14723	XP_012552995|39S ribosomal protein L16, mitochondrial *Bombyx mori*	4E–132
Tag_548	GACGACTGGCCCTCGGTGCGCC	12.19	1209	XP_013183026|ATP‐dependent Clp protease ATP‐binding subunit clpX‐like, mitochondrial isoform X3 *Amyelois transitella*	0E+00
Tag_805	GGACGCGGGTGAAACCGCTGGC	12.19	11489	KOB70085|Putative collagen alpha‐2 IV chain protein *Operophtera brumata*	1E–32
Tag_812	AGACATTCTAAGAATGTCGGTC	11.93	34007	AAK59928|Ribosomal protein S11 *Heliothis virescens*	1E–67
Tag_561	GGCGGGCGCGGCGGCCGCGGCG	11.68	671	XP_013190420|RNA‐binding protein squid isoform X2 *Amyelois transitella*	3E–122
Tag_119	GAGTCCTCGTTGGTGTCACCTC	11.66	21108	AFO68320|Trypsin *Heliothis virescens*	4E–119
Tag_427	TTTTTTATTTCTCTCTGTACAC	11.60	5986	No hit	–
Tag_188	TGGGCAGACGCCACGCTCGCTA	10.19	7598	XP_012552008|UDP‐glucose 4‐epimerase‐like *Bombyx mori*	0E+00
Tag_888	AAACAGGGAGTCCTCACCAACA	9.65	12709	Q95V32|40S ribosomal protein S6 *Spodoptera frugiperda*	7E–161
Tag_195	GTCCAATAAATTCTTTGGGTCG	9.62	14879	NP_001298530|60S ribosomal protein L19 *Papilio polytes*	9E–95
Tag_80	CCCGGCGGTATGCCCGGCGGCA	9.16	4349	AIZ00749|Heat shock cognate 70 protein, partial *Sesamia inferens*	0E+00
Tag_190	GTCCAGTACCGGCGACGCATCT	8.91	34866	XP_001895031|Hypothetical protein Bm1_17870 *Brugia malayi*	4E–25
Tag_942	TGCCGCGGCCTGTGCGGCGAGC	8.89	1945	XP_013143932|NFX1‐type zinc finger‐containing protein 1‐like *Papilio polytes*	4E–160
Tag_50	CCCGCTGGTGGCGCCGCTCCCG	8.83	27905	KPJ13320|60S acidic ribosomal protein P2 *Papilio machaon*	2E–33
Tag_2	CACTCGGCCGAGCGGCCGGTGG	8.77	47003	KPJ15203|Hypothetical protein RR48_09230 *Papilio machaon*	9E–24
Tag_38	CACTCGGTCGAGCGGCCGGTGG	8.59	5683	No hit	–
Tag_270	ATCTGCGCAGGATGGCTCGATG	8.45	15523	XP_004931376|Trypsin, alkaline A‐like *Bombyx mori*	1E–44

^†^Tag represented as a 22‐bp sequence excluding the NlaIII site (5′‐CATG‐3′).

^‡^FC: fold change (Vip–Sel vs. Vip–Unsel).

**Table 2:** Top 40 from the list of 317 larval gut unigenes identified as underexpressed in the Vip–Sel strain by HT‐SuperSAGE

Tag code	Tag sequence†	LOG(FC)‡	Hv_Contig_	Protein description	*E*‐value
Tag_755	TATGCGAGTCATTGAGATAATA	−9.79	14845	XP_001624571|Predicted protein *Nematostella vectensis*	2E–23
Tag_4060	CTGATGCTGCTGCCGTTCCTGC	−6.78	4634	XP_012549161|Solute carrier family 12 member 6 isoform X2 *Bombyx mori*	0E+00
Tag_4722	ATTGAAATATGCATCTATTTGG	−6.15	9978	ABR88239|Chymotrypsin‐like protease C9 *Heliothis virescens*	8E–127
Tag_1852	GAACTCGAACGCTCAGGCAGAA	−6.03	21233	KPJ14522|Chorion peroxidase *Papilio machaon*	7E–70
Tag_4310	TGGCGCGCAGGCGTCGCGTGCG	−5.78	399	EHJ69302|venus kinase receptor *Danaus plexippus*	4E–96
Tag_4719	GGAACTACTCGGCGCACCCGGA	−5.78	23364	XP_013199167|Uncharacterized protein LOC106142086 *Amyelois transitella*	4E–11
Tag_3223	AACCAGTGTATGTAAGGTGTAC	−5.75	66	XP_011555786|Uncharacterized protein LOC105386838 *Plutella xylostella*	0E+00
Tag_1705	CCTGGGTGTGCCACCGCGCTCT	−5.61	9835	AFI64311|Neutral lipase *Helicoverpa armigera*	0E+00
Tag_4868	TCGCTTATGCCAGCGATAACGG	−5.56	143	EHJ65667|Putative YLP motif containing 1 *Danaus plexippus*	7E–142
Tag_4908	AGCCCACGGAACGCGCCCCCAC	−5.44	13176	XP_013182903|ATP synthase mitochondrial F1 complex assembly factor 2 isoform X1 *Amyelois transitella*	3E–147
Tag_2652	CGCACGCGCAAGTCTGCCGTCA	−5.36	13406	KPJ02027|WD repeat‐containing protein 43 *Papilio xuthus*	6E–74
Tag_4176	CGTGGCTTGCTCGAATAGGCGG	−5.30	11166	ACN29686|UDP‐N‐acetylglucosamine pyrophosphorylase *Spodoptera exigua*	1E–20
Tag_5119	AGCAGCCTGGCGAGCAGCGTGC	−5.30	25750	KPJ15001|Trafficking kinesin‐binding protein milt *Papilio machaon*	6E–67
Tag_4740	ATTGCAACGGGAGCGAAGGAAA	−5.15	16686	ADT80643|Ribosomal protein S3 *Euphydryas aurinia*	7E–158
Tag_4815	CGGCGCCTCCGCCACATTCTAT	−5.15	1883	XP_004932565|Probable aconitate hydratase, mitochondrial *Bombyx mori*	3E–38
Tag_5163	GCCCAACTGCCTGATGTGCCGA	−5.15	18739	AID66662|Desaturase *Agrotis segetum*	7E–111
Tag_2538	AAGTACTGCGAGTTTGCCGACC	−4.98	3779	XP_004928865|N‐acetylglucosamine‐6‐sulfatase‐like *Bombyx mori*	0E+00
Tag_4130	TATTAGAAGAAATAGAGATAAG	−4.98	21488	YP_009183765|NADH dehydrogenase subunit 2 (mitochondrion) *Heliothis subflexa*	9E–51
Tag_5140	TTGGCCCTGAGAGTCGTCTCTA	−4.98	16252	ABU98613|Alpha‐amylase *Helicoverpa armigera*	2E–140
Tag_1353	GTGGGCCGCATCGGCTCCATCA	−4.81	13483	KOB66490|Organic cation transporter *Operophtera brumata*	7E–132
Tag_4312	CTGGCCGCCTGGGGGTACACCA	−4.78	3630	XP_013190963|Atlastin‐like *Amyelois transitella*	0E+00
Tag_5051	TGGGCGGAGCTCCAAGAGACGG	−4.78	32076	XP_013192188|Tat‐linked quality control protein TatD *Amyelois transitella*	9E–91
Tag_5234	GCGCGCTCGGTTCCGCGATGCG	−4.78	16308	XP_013185861|Probable NADH dehydrogenase [ubiquinone] 1 alpha subcomplex subunit 12 *Amyelois transitella*	3E–64
Tag_3476	CCACCAGATCCGGCCCCTGTGC	−4.68	26585	XP_013200478|Protein transport protein Sec61 subunit beta *Amyelois transitella*	5E–16
Tag_3899	GTCCACTCACCACTGACGAGGG	−4.68	11717	XP_013192568|Carbonyl reductase [NADPH] 3‐like *Amyelois transitella*	3E–142
Tag_1327	GCCATCAACAACGCCCTGGTCG	−4.64	22796	AHL46496|Trypsin *Helicoverpa armigera*	2E–82
Tag_159	CCCGGCCACCAGCTGTTCGCGC	−4.64	19906	XP_012553210|Epidermal growth factor receptor kinase substrate 8‐like isoform X3 *Bombyx mori*	2E–65
Tag_4475	AGTTTGTGTTGACAAATGCAGA	−4.56	3175	XP_013195286|Plasminogen activator inhibitor 1 RNA‐binding protein‐like *Amyelois transitella*	1E–111
Tag_4711	GGCTACCTCAACGATGACGCGA	−4.56	9310	XP_004930170|Very long‐chain‐fatty‐acid–CoA ligase bubblegum isoform X2 *Bombyx mori*	0E+00
Tag_5002	AAGCCGACGGTTCCATCAGAAC	−4.56	27599	NP_001166668|Cuticular protein RR‐2 motif 97 precursor *Bombyx mori*	5E–37
Tag_5127	TGAGGACTTTCACACCAGAGGT	−4.56	11768	NP_001299708|Fatty acid‐binding protein‐like *Papilio xuthus*	2E–46
Tag_2601	GTGCAGCTACTCAGCGTGGTGC	−4.46	35307	XP_013197877|Solute carrier family 35 member E1 homolog *Amyelois transitella*	2E–36
Tag_2655	TTGTAATGGTGTTTATGTGATT	−4.46	23072	ACO58577|Heat shock protein 90 *Apis mellifera*	2E–44
Tag_3892	AAGATCTTCGTATACAGTCCTC	−4.44	20572	KPJ17487|Cytochrome c oxidase subunit 4 isoform 1, mitochondrial *Papilio machaon*	2E–69
Tag_2444	CGGACGTGGAACTGATCCTTGC	−4.39	19497	ABX54738|Ribosomal protein L10 *Spodoptera exigua*	2E–90
Tag_2626	CCTGCTATGCTCTTTGTTGAAC	−4.36	8800	XP_013188449|TAR DNA‐binding protein 43‐like *Amyelois transitella*	7E–167
Tag_4245	GACCCGCCGAACAGAGGCTCCA	−4.30	12755	AEA76329|Chitin binding domain 3 protein *Mamestra configurata*	1E–134
Tag_4406	CCAGCCGGTTTCCGCATAATTT	−4.30	37137	XP_011015409|Pre‐mRNA‐splicing factor 38A‐like *Populus euphratica*	5E–24
Tag_4639	CGGCTCTTTGGATGGGAAGCTG	−4.30	11302	XP_010462852|Zinc finger MYM‐type protein 1‐like *Camelina sativa*	1E–08
Tag_4871	ACTGCGAAGCCGAGGAGTGCGC	−4.30	12596	XP_013183892|Zinc transporter ZIP1‐like *Amyelois transitella*	4E–69

^†^Tag represented as a 22‐bp sequence excluding the NlaIII site (5′‐CATG‐3′).

^‡^LOG(FC): fold change (Vip–Sel vs. Vip–Unsel), expressed as the log2 value.

**Figure 3:**
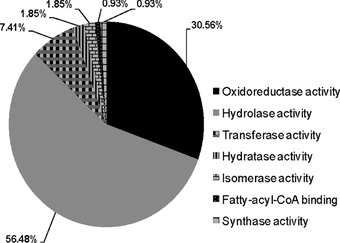
Classification of the 108 differentially expressed unigenes associated with metabolism into different catalytic activities.

Among the most prevalent GO biological processes, that is, with more than 10% of UniTags, 25.96% classified into metabolism and 15.87% into translation/ribosome biogenesis (Fig. 2C). Categories ranging between 10% and 5% of UniTags were nucleic acid binding (8.17%), transport/trafficking (7.69%), and protein folding/recycling (6.25%). For OE unigenes, 47% classified into metabolism and translation/ribosome biogenesis categories, whereas, it was 40% for the UE UniTags. The last suggests, changes in transcripts corresponding to these processes are very important for gut cells adaptation against Vip3Aa protoxin in *H. virescens*. Regarding to metabolism category, 108 UniTags distributed in seven catalytic activities were found to be regulated in Vip–Sel strain, from which, hydrolases (∼56%) and oxidoreductases (∼31%) were the most represented (Fig. 3). Interestingly, almost half the unigenes categorized as hydrolases (27 of 61 UniTags) belonged to serine‐type hydrolases (endopeptidases).

Since UniTags with homology to serine‐type endopeptidases and ribosomal proteins were highly represented in HT‐SuperSAGE data and because both activities have been linked before to the Vip3A toxic pathway, particularly in protoxin solubilization/activation and receptor binding steps, these categories were further analyzed in more detail.

### HT‐SuperSAGE of Vip–Sel revealed a high number of OE and UE UniTags with homology to serine‐type proteases

**Figure 4:**
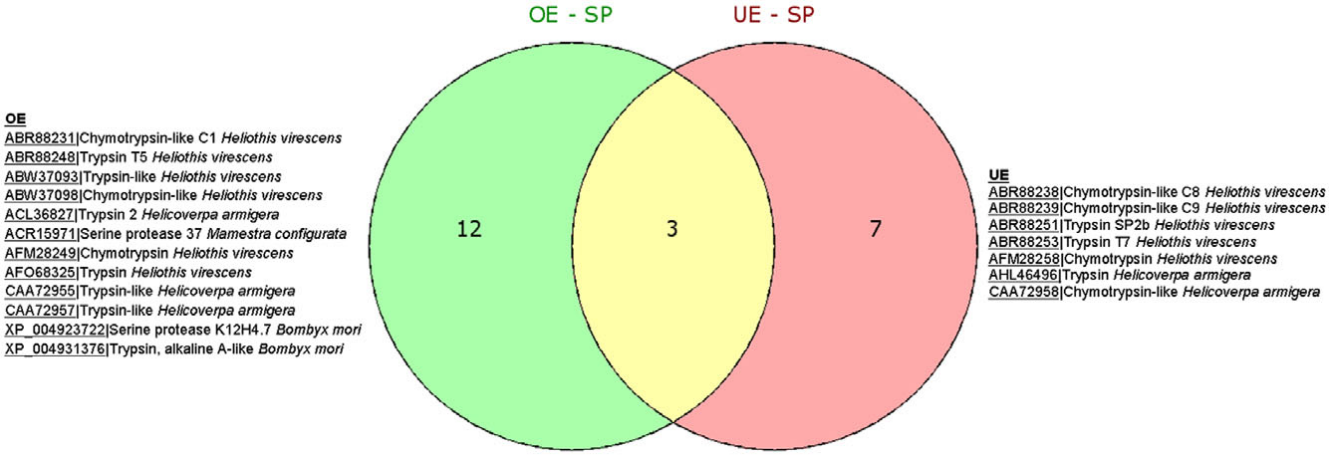
Venn diagrams showing distribution of over‐ and underexpressed serine‐type endopeptidases unigenes in Vip–Sel strain of *H. virescens*.

The HT‐SuperSAGE data suggest Vip3Aa resistance in Vip–Sel larvae has an important component of serine protease‐mediated proteolysis; 27 (∼8%) of a total of 345 GO annotated UniTags matched putative trypsin‐ and chymotrypsin‐like products. The Venn diagram of Figure 4 shows 22 of the 27 annotated serine‐proteases were considered unique, with 12 exclusively found in the OE UniTags, 7 found only in the UE unigenes, and 3 overlapped. Interestingly, there were more trypsins positively regulated than chymotrypsins in Vip–Sel, whereas, the opposite effect was observed for the group of underexpressed serine‐proteases. In Table 1, Tag_310 with homology to serine protease 37 from *Mamestra configurata* showed the highest fold change (FC = 13.08) among all overexpressed serine‐proteases, while none of the unigenes annotated as chymotrypsin was detected in the list of 40 most overexpressed genes in Vip–Sel. Thus, Tag_4722 annotated as *H. virescens* chymotrypsin‐like protease C9 was found third in the list of 40 most underexpressed UniTags (FC = –6.15) (Table 2). A differential role for trypsin‐ and chymotrypsin‐like proteases in the activation and degradation of the approximately 62 kDa toxin core of Vip3Aa has been previously proposed in *Spodoptera* sp. (Caccia *et al*., 2014).

### Vip3Aa adaptation involves differential expression of 42 UniTags with homology to ribosomal proteins

**Figure 5:**
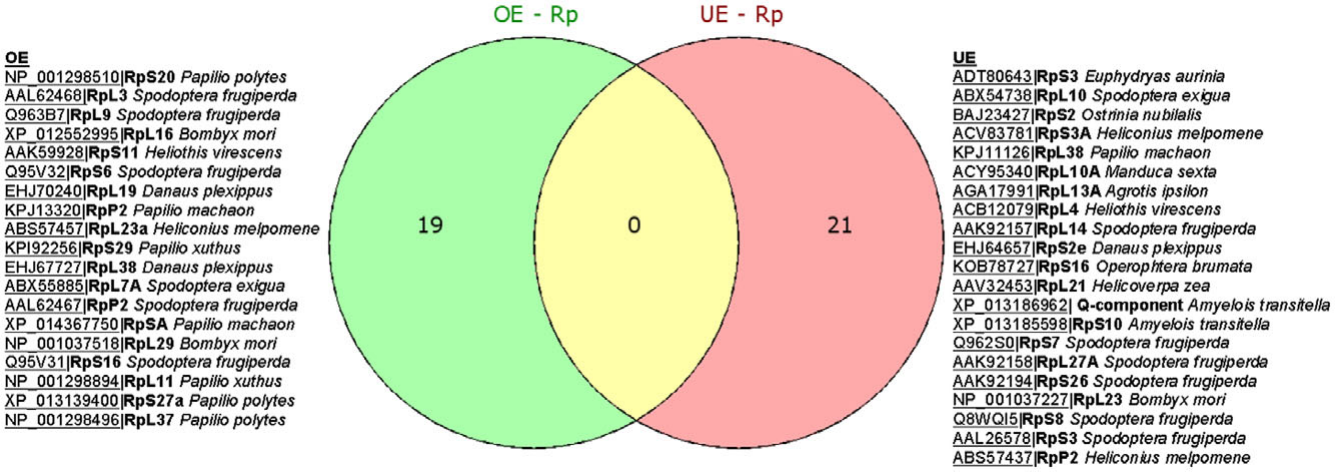
Venn diagrams showing distribution of over‐ and underexpressed ribosomal protein (Rp) unigenes in Vip–Sel strain of *H. virescens*.

Ribosomal proteins play critical roles in ribosome biogenesis, translation and posttranslational modifications of proteins. In addition to their housekeeping functions, diverse roles as caretakers of cellular stress have been informed (Kim *et al*., 2014). HT‐SuperSAGE data revealed 42 (∼12%) of the 345 GO annotated UniTags with homology to ribosomal proteins. Forty of these 42 ribosomal proteins were unique and were found distributed, in terms of gene expression regulation, as 19 and 21 for OE and UE UniTags, respectively (Fig. 5). Curiously, overexpressed unigenes were enriched in ribosomal proteins of the large subunit (RpL), that is, 12 of 19 UniTags, with 5 of them among the top 40 overexpressed UniTags (Table 1). On the contrary, the ratio between RpL and ribosomal proteins of the small subunit (RpS) in UE UniTags was equivalent (Fig. 5). Several of the ribosomal proteins found overexpressed have been previously described as major stress‐responding factors, such us, RpS20, RpS6, RpL11, and RpL37, playing important roles in cellular quality control by regulating the MDM2‐p53 pathway (Kim *et al*., 2014). RpS2, one of the two putative Vip3Aa‐binding proteins known so far, was found underexpressed (Singh *et al*., 2010).

### Validation of the HT‐SuperSAGE data by quantitative real‐time PCR (qRT‐PCR)

**Figure 6:**
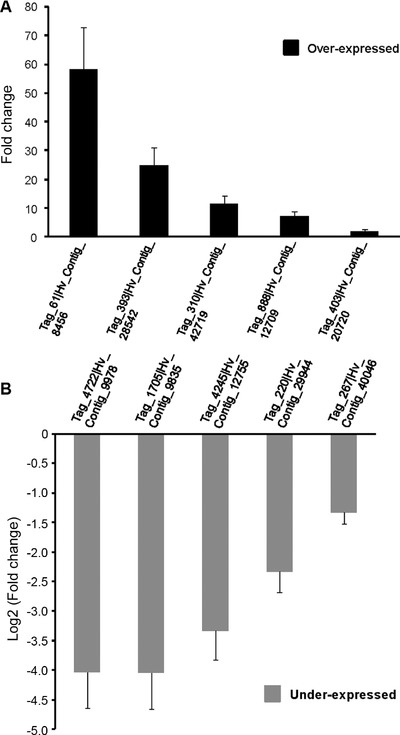
Validation of HT‐SuperSAGE data by qRT‐PCR. For each unigene, the fold change parameter is the ratio of corresponding “Mean Normalized Expression” value, calculated in the Q‐Gene software, using Vip–Sel cDNAss template to that using Vip–Unsel cDNAss template. Bars represent means of two independent replicates ± standard error.

Ten UniTags (five OE and five UE) were chosen for validating the HT‐SuperSAGE data by qRT‐PCR. For this, longer cDNA fragments were recovered by 3′‐RACE and DNA sequences verified to match the same entries as the original 26 bp tags when BLASTed against *H. virescens* sequence contigs (Perera *et al*., 2015). The validation was performed for overexpressed *serine protease 37* (Hv_Contig_42719|Tag_310), *repat39* (Hv_Contig_8456|Tag_61), *ribosomal protein S20* (*RpS20)* (Hv_Contig_28542|Tag_393), *ribosomal protein S6 (RpS6)* (Hv_Contig_12709|Tag_888), and *aminopeptidase N* (*APN*) (Hv_Contig_20720|Tag_403) and underexpressed *chymotrypsin‐like protease C9* (Hv_Contig_9978|Tag_4722), *trypsin T7* (Hv_Contig_40046|Tag_267), *neutral lipase* (Hv_Contig_9835|Tag_1705), *RpS2* (Hv_Contig_29944|Tag_220), and *chitin binding domain 3 protein* (Hv_Contig_12755|Tag_4245). All but *trypsin T7*, *APN* and *RpS2* were found among most strongly regulated UniTags in Vip–Sel (Tables 1 and 2). We chose overexpressed *APN* and underexpressed *RpS2* for the validation study because of the well‐known role of receptor for Bt Cry1‐class toxins of the former (Pigott & Ellar, 2007) and the putative Vip3A‐binding protein role informed for the last one (Singh *et al*., 2010). Also, upregulation of *repat39* gene in response to Bt toxins has been previously noted in other insect species (Bel *et al*., 2013), and overexpression of a member of the *repat* superfamily was found to protect *S. exigua* from pathogen attack (Herrero *et al*., 2007). The expression profiles determined by qRT‐PCR were consistent with and confirmed the HT‐SuperSAGE results (Fig. 6).

## Discussion

In this study, we have used HT‐SuperSAGE to quantify the expression of thousands of genes differentially regulated in the gut tissue of a Vip3Aa‐resistant strain of *H. virescens*. HT‐SuperSAGE, the result of combining an improved version of Serial Analysis Gene Expression with Next Generation Sequencing, has proven to be a valuable tag‐based transcriptome sequencing method in a wide field of applications (Bonaventure, 2010; Gilardoni *et al*., 2010; Sharbel *et al*., 2010; Gilardoni *et al*., 2011; Molina *et al*., 2011; Kahl *et al*., 2012; Draffehn *et al*., 2013). To our knowledge, this is the first report on the use of HT‐SuperSAGE to produce a digital gene‐expression profiling in a nonmodel lepidopteran pest.

In our study, a fraction of the 26‐bp tags mapped to a sequence distant from the 3′‐end of a *H. virescens* contig suggesting either cDNA digestion with *Nla*III was not very efficient or a low frequency of cleavage sites for this particular 4‐bp cutter enzyme in the genes of this insect. Any of above could have consequences in the transcription profile produced by HT‐SuperSAGE. Though *Nla*III is the standard anchoring enzyme in all versions of SAGE, other 4‐bp restriction enzymes could be also used, for example, *Dpn*II and *Bfa*I. Among all of them, we choose *Nla*III because Adapter‐2 used for libraries construction already carried a cohesive end for the *Nla*III site (5′‐CATG‐3′) (Matsumara *et al*., 2010) and due to previous findings of a similar frequency for *Nla*III and *Dpn*II restriction motifs but a lower for *Bfa*I in genes of different invertebrates, that is, *Drosophila melanogaster* and *Caenorhabditis elegans*, during *in silico* genome scan (Pleasance *et al*., 2003). Also, we performed double *Nla*III digestion during libraries construction as recommended by Gilardoni *et al*. (2010). However, cDNA of certain organisms is frequently not efficiently digested by *Nla*III but instead by DpnII (Sharbel *et al*., 2010). Future SuperSAGE studies involving lepidopteran insect species should evaluate the effect of other anchoring enzymes in addition to *Nla*III on the final expression profile by comparing tag extraction rate with different 4‐bp restriction endonucleases on the same cDNA sample.

Our results showed 11.2% of the 17 751 sequenced UniTags were differentially regulated, either overexpressed or underexpressed, in the gut tissue of Vip–Sel compared to Vip–Unsel strains. We found the amount of underexpressed unigenes in Vip–Sel was significantly greater (more than three times) than those found constitutively overexpressed, suggesting a decreased translation rate could be an important adaptation for Vip3Aa resistance in this strain of *H. virescens*. Previously, Bel *et al*. (2013) used a genome‐wide microarray to analyze transcriptional changes occurring in the gut tissue of *S. exigua* after the exposure to a sublethal concentration of Vip3Aa. These authors found 19% of all microarray unigenes had significant alterations in their expression levels, with a similar number of up‐ and downregulated unigenes. Comparison between studies using different insect and transcriptomic approaches is not straightforward but a wider transcriptional change in susceptible insects after Vip3Aa exposure may represent a less specific response triggered to cope with general stress rather than a signature of specific adaptation to the toxin. On the contrary, constitutive transcriptional alterations detected in the gut of Vip3Aa‐resistant (unexposed) insects may better reflect the outcome of adaptation following toxin selection. This hypothesis has been supported for Bt‐resistant strains of *S. exigua* and *Plutella xylostella* where transcriptional alterations found in gut cells of resistant (unexposed) insects overlapped with that triggered in susceptible counterpart strains when exposed to a toxic Bt product, suggesting a constitutive activation of defensive mechanisms against this invertebrate pathogen in the former (Hernández‐Martínez *et al*., 2010; Ayra‐Pardo *et al*., 2015). One example could be the identification of UniTags exclusively related to *repat39* among the constitutively overexpressed unigenes of *H. virescens* Vip–Sel strain against the broad response of *repat* genes, 29 different members in total, detected in susceptible *S. exigua* larvae after Vip3Aa feeding (Bel *et al*., 2013). We verified gene expression data by qRT‐PCR of 10 genes, 5 with increased and 5 with decreased expression in Vip–Sel, and found consistent results, which provide confidence in the reliability of the HT‐SuperSAGE data.

The analysis of GO functional categories showed the highest amount of annotated UniTags (108 of 345) belonged to different metabolic processes (Fig. 2C). Similar dominance of metabolism‐related unigenes was previously reported in Cry1Ab‐resistant *Diatraea saccharalis* (Guo *et al*., 2012), Cry1Ac‐resistant *P. xylostella* (Lei *et al*., 2014), and Cry1Ab‐resistant *Ostrinia furnacalis* (Xu *et al*., 2015). As in the last of above studies, we found the majority of metabolism‐related UniTags (∼76%) underexpressed in the resistant Vip–Sel strain compared to the susceptible Vip–Unsel control. In our study, metabolism GO category was enriched in hydrolase activities, particularly, serine (trypsin‐ and chymotrypsin‐type) hydrolases that represented the 25% of UniTags in this category with 12 found exclusively overexpressed and other 7 in the group of underexpressed UniTags.

Trypsin‐ and chymotrypsin‐type serine proteases are the primary digestive enzymes in the gut lumen of lepidopteran larvae. Serine‐proteases are also the major determinants of insecticidal potency for Bt Cry and Vip3A toxins by affecting their solubilization and activation in the gut lumen of target insects (Oppert, 1999; Ligthwood *et al*., 2000; Chakroun *et al*., 2012). Differential natural susceptibility for Vip3Aa between two *Spodoptera* sp. was attributed to observed differences in the rate of activation of the protoxin form (Chakroun *et al*., 2012). In fact, Vip3A‐selected resistance in *S. litura* has been associated with a reduced proteolytic activity in the larval gut fluids (Barkhade & Thakare, 2010). In *H. armigera*, biochemical analyses of resistant and susceptible insects found no differences at the level of toxin binding but it was in the activation rate of Vip3Aa protoxin with minor differences between both strains, which may or may not contribute to resistance (Chakroun *et al*., 2016a,b).

Among overexpressed serine‐type endopeptidases of *H. virescens* Vip–Sel strain, we found one with homology to Serine Protease 37 of *M. configurata* with the highest FC value during the analysis of HT‐SuperSAGE data. qRT‐PCR experiments verified the constitutive overexpression of this gene in the resistant compared to susceptible control strain. Previous transcriptomic studies of the gut tissue of Vip3Aa‐challenged larvae from two *Spodoptera* sp. have presented contradictory results regarding the regulation of this gene, which was downregulated in *S. exigua* at 8 and 24 h after toxin exposure (Bel *et al*., 2013), whereas it was upregulated in *S. litura* 24 h after feeding larvae with Vip3Aa (Song *et al*., 2016). Moreover, serine proteases were in general among the strongly repressed unigenes in the Vip3Aa‐exposed *S. exigua* larvae, while, there was 14 gut genes coding for serine proteases upregulated and 7 downregulated in *S. litura* as a result of Vip3Aa treatment. Downregulation of specific serine proteases has shown to promote survival against certain Bt toxins (Rodriguez‐Cabrera *et al*., 2010; Liu *et al*., 2014), in which case, increased expression detected for other serine proteases might represent compensatory changes to replace lost functionality.

The proteolytic processing of Vip3Aa protoxin with insect gut juice yields a fragment of approximately 62 kDa as the major product, which has been found to specifically bind and open lytic pores in brush border membrane vesicles (BBMV) of *Helicoverpa armigera*, suggesting that this is the active toxin core (Yu *et al*., 1997; Lee *et al*., 2003, 2006; Liu *et al*., 2011; Chakroun *et al*., 2012). In a recent study, *in vitro* experiments were performed to evaluate the production and degradation of the ∼62 kDa Vip3Aa active toxin core in the presence of trypsin‐ and chymotrypsin‐like serine proteases (Caccia *et al*., 2014). These authors found both processes to occur more efficiently with trypsin than chymotrypsin even at low concentrations of the enzyme. They also reported *Spodoptera frugiperda* cationic trypsin‐like peptidases together with anionic chymotrypsin‐like peptidases were important for accumulation of the ∼62 kDa active toxin fragment while the cationic chymotrypsin‐like activities mainly participated in its degradation. In the Vip–Sel strain, overexpressed serine‐proteases encoding genes were mainly for trypsin‐like activities, while, chymotrypsins dominated the underexpressed UniTags except for two, Tag_146|Hv_Contig_12402 and Tag_69|Hv_Contig_40932 with homology to *H. virescens* chymotrypsin‐like C1 (acc. No. ABR88231) and chymotrypsin‐like (acc. No. ABW37098), respectively (Table S2), which were found accompanying the overexpressed trypsin‐like proteases (Fig. 4). In future studies, it would be interesting to functionally investigate these two chymotrypsin‐like genes for any role in proteolytic degradation of the ∼62 kDa Vip3Aa active core and hence, in promoting resistance against this toxin in *H. virescens*.

The other highly represented category in the HT‐SuperSAGE data was translation/ribosome biogenesis, with 66 UniTags that accounted for ∼19% of the 345 GO annotated unigenes. Most of the components in this category corresponded to ribosomal proteins of the large and small subunits, which are known to play essential roles in ribosome biogenesis and protein translation governing cell growth, proliferation and development (Schleif, 1967; Wool, 1979; Donati *et al*., 2012). Our data showed dramatic differences in the expression of genes encoding ribosomal proteins between resistant Vip–Sel and susceptible Vip–Unsel insects, not only in terms of the expression of particular ribosomal proteins but also in the RpL/RpS ratio found in each strain (Fig. 5). Ribosomes can alter protein and RNA composition to selectively translate specific subpopulations of mRNAs in response to stress and development issues, a phenomenon described as the “ribosome filter hypothesis” (Byrne, 2009; Gilbert, 2011; Moll & Engelberg‐Kulka, 2012; Xue & Barna, 2012). Thus our differential expression data could indicate possible changes in ribosome composition in the Vip–Sel strain. The rationality of a particular ribosome composition with a high RpL/RpS ratio in resistant Vip–Sel strain merits more investigation; it could be an important adaptation for the selective translation of a subpopulation of genes involved in the cellular response and survival to Vip3Aa toxin.

A subset of ribosomal proteins has shown extra‐ribosomal functions in response to stress (Zhou *et al*., 2015). Among these, RpS20, RpL37 and others that were found overexpressed in Vip–Sel, can act as tumorigenesis suppressors in its ribosome‐free state activating the Mdm2‐p53‐MdmX signaling network that stabilizes p53 and arrests cell cycle at G2 (Daftuar *et al*., 2013). Interestingly, Tag_1134| Hv_Contig_7932 that was GO annotated as E3 ubiquitin‐protein ligase Mdm2‐like protein was identified in the group of underexpressed Vip–Sel UniTags (Table S3). Mdm2‐mediated ubiquitination targets p53 for nuclear export and degradation by the proteasome (Toledo & Wahl, 2006). At this point, it is not clear whether constitutive overexpression of these ribosomal proteins could be contributing through any extra‐ribosomal function to Vip3Aa resistance in Vip–Sel; further research on gene function analysis would be needed. Interestingly, cell cycle arrest at the G2–M transition has been associated with resistance to a Bt Cry toxin in the highly susceptible Sf9 cells (Avisar *et al*., 2005). These authors correlated Cry1C‐resistance of arrested Sf9 cells with a reduction in the Cry1C‐binding capacity and the inability to isolate lipid‐raft domains from the plasmatic membrane where the Cry1C‐receptor APN resides (Rajagopal *et al*., 2002; Zhuang *et al*., 2002). The previously described Vip3Aa‐binding protein RpS2 was found to be among underexpressed ribosomal proteins in Vip–Sel. If RpS2 is the receptor of Vip3Aa in *H. virescens*, suggested for *S. litura* by Singh *et al*. (2010), its constitutive underexpression would affect toxin binding and help to explain the Vip3Aa resistance phenotype of this strain. However, preliminary studies conducted at Prof. Juan Ferré's laboratory (Valencia, Spain) on Vip3Aa binding to BBMV prepared from Vip–Sel and Vip–Unsel guts showed no differences (unpublished). This and same finding reported in a Vip3Aa‐resistant *H. armigera* strain (Chakroun *et al*., 2016a,b) suggest binding alteration could not be part of the biochemical basis of resistance against this toxin in these insects. Transcriptomic studies like ours, combined with functional validation of specific targets, are useful to identify which alterations other than differential expression of receptor genes between resistant and susceptible insects could be contributing toward resistance. Also, unknown cellular targets of the Bt toxin can be revealed to better understand the toxic pathway of these proteins in insects and design improved insecticidal proteins and effective resistance management and monitoring strategies. In this context, where continuous exposure to lepidopteran‐active Cry1 and Cry2 toxins in Bt‐crops has made resistant insect populations to emerge, preserving Vip3Aa toxin is crucial for the technology.

In summary, we report here the first transcriptomic study of a Vip3Aa‐resistant lepidopteran insect that is also a key polyphagous pest of economically important crops in the Western hemisphere (Teran‐Vargas *et al*., 2005; Blanco *et al*., 2008). This is also the first report of successful application of HT‐SuperSAGE approach to a nonmodel lepidopteran insect. The analysis of HT‐SuperSAGE data enabled the identification of multiple factors with constitutive transcriptional alterations in the Vip3Aa‐resistant *H. virescens* Vip–Sel strain. Further gene function analysis based on HT‐SuperSAGE data will shed light on the possible molecular mechanisms behind Vip3Aa resistance in *H. virescens* and related species.

## Disclosure

The authors have no conflict of interests to declare.

## Supporting information


**Table S1**. Nucleotide sequence of the primers and experimental conditions used in quantitative RT‐PCR. All sequences read 5′ to 3′, left to right.

**Table S2**. List of overexpressed (OE) UniTags sequences, copy number, fold change, and annotations to the *Heliothis virescens* sequence contigs database.

**Table S3**. List of underexpressed (UE) UniTags sequences, copy number, and annotations to the *Heliothis virescens* sequence contigs database.
