# Supplementary material for: HT‐SuperSAGE of the gut tissue of a Vip3Aa‐resistant Heliothis virescens (Lepidoptera: Noctuidae) strain provides insights into the basis of resistance
Source: Insect Sci. 2017 Dec 1;26(3):479–98. doi: 10.1111/1744-7917.12535 (PMC6849831; doi:10.1111/1744-7917.12535)
Supplement: Supplementary file 1 — Table S1. Nucleotide sequence of the primers and experimental conditions used in quantitative RT‐PCR. All sequences read 5′ to 3′, left to right. [file INS-26-479-s001.doc]

**Table S1.**

| **Gene** | **Primer sequence (5’-3’)** | **Product size (bp)** | | **Ta^a^ (°C)** | **Cycle no.** | | **S^b^** | **E^c^** | **R^2d^** |  |
| --- | --- | --- | --- | --- | --- | --- | --- | --- | --- | --- |
| *β-Actin* | atctccaaacaggagtacgatga | 223 | 60 | | | 40 | -3.414 | 1.963 | 0.997 | |
|  | gacaatgttccgcattcattatt |  |  | | |  |  |  |  | |
| Hv_Contig_8456|Tag_61 | atagcgaatgctctgaaggaag | 232 | 59 | | | 40 | -3.964 | 1.787 | 0.992 | |
|  | aattgtccaagtgaacgctttt |  |  | | |  |  |  |  | |
| Hv_Contig_28542|Tag_393 | aaacagaagctccgtgttaagg | 188 | 59 | | | 40 | -3.378 | 1.9771 | 0.992 | |
|  | tcgatgttgatggatgtgatct |  |  | | |  |  |  |  | |
| Hv_Contig_12709|Tag_888 | ctatgtcgtcaaacgtctgctc | 225 | 59 | | | 40 | -3.649 | 1.8795 | 0.992 | |
|  | tcctgacgtcttaccttggatt |  |  | | |  |  |  |  | |
| Hv_Contig_20720|Tag_403 | gcaatgttgatgccattagaaa | 240 | 59 | | | 40 | -2.920 | 2.200 | 0.997 | |
|  | gttgctcttttgctggagttct |  |  | | |  |  |  |  | |
| Hv_Contig_40046|Tag_267 | ctctctaccacaatggcgttct | 158 | 59 | | | 40 | -3.654 | 1.8779 | 0.999 | |
|  | caaagatgtgtcggtccagtt |  |  | | |  |  |  |  | |
| Hv_Contig_42719|Tag_310 | ttctgatcggtgtcagcactt | 174 | 59 | | | 40 | -3.724 | 1.8558 | 0.998 | |
|  | cagatgtctgttgatccagctc |  |  | | |  |  |  |  | |
| Hv_Contig_29944|Tag_220 | ctctgaacgatggtaggtcctc | 219 | 59 | | | 40 | -2.907 | 2.208 | 0.995 | |
|  | ttgttaccccagtaacctctgc |  |  | | |  |  |  |  | |
| Hv_Contig_9978|Tag_4722 | ggaatctgccaacaagtacctc | 179 | 59 | | | 40 | -2.879 | 2.2251 | 0.999 | |
|  | caatcatggtgactccttcgta |  |  | | |  |  |  |  | |
| Hv_Contig_9835|Tag_1705 | ggtttcaacactgttctcgtacc | 188 | 59 | | | 40 | -3.461 | 1.9451 | 0.999 | |
|  | tagccgagaatgtggtagtcatt |  |  | | |  |  |  |  | |
| Hv_Contig_12755|Tag_4245 | cttcttctcttgctccgacataa | 228 | 59 | | | 40 | -3.472 | 1.941 | 0.994 | |
|  | atttttggcacaaacgtaagag |  |  | | |  |  |  |  | |

^a^ Optimal annealing temperature in the PCR program for a specific primer set.

^b^ Slope of the linear regression line of the amplification efficiency plot.

^c^ Amplification efficiency calculated by Q-gene as: 10^(-1/slope).

^d^ Reproducibility of the real time PCR reaction.
