# Supplementary material for: HT‐SuperSAGE of the gut tissue of a Vip3Aa‐resistant Heliothis virescens (Lepidoptera: Noctuidae) strain provides insights into the basis of resistance
Source: Insect Sci. 2017 Dec 1;26(3):479–98. doi: 10.1111/1744-7917.12535 (PMC6849831; doi:10.1111/1744-7917.12535)
Supplement: Supplementary file 2 — Table S2. List of overexpressed (OE) UniTags sequences, copy number, fold change, and annotations to the Heliothis virescens sequence contigs database. [file INS-26-479-s002.pdf]

Vip-Sel: number of Tags in the Vip-Sel library

Vip-Unsel: number of Tags in the Vip-Unsel library

Norm. Vip-Sel: number of tags in Vip-Sel normalized to 268000 (See Materials & Methods for details)

Norm. Vip-Unsel: number of tags in Vip-Unsel normalized to 268000 (See Materials & Methods for details)

p **-value: Statistical value associated with the different in tag copy number between libraries calculated according to Audic and Claverie (1997)**

FC: fold.change (Norm. Vip-Sel vs. Norm. Vip-Unsel)

Heliothis virescens CONTIG match: Identifier of the Contig sequence of Heliothis virescens (from Perera et al., 2015) that perfectly matched (26bp) with the corresponding tag in BlastN search

List of over-expressed Unitags sequences, copy number, fold change and annotations to the Heliothis virescens sequence contigs database

General classification (Process/Molecular Function)	TAG	Vip-Sel	Vip-Unsel	Norm. Vip-Sel	Norm. Vip-Unsel	*p*-value	FC	Heliothis virescens CONTIG match	Contig size (nt)	Tag position in the contig (nt)	Protein annotation: blastx	E-value
-	Tag_32 CATGTTGTGCTCTGGCTGGAACGGCG	3631	2	921.434	1.999	0.00E+00	461.013	no hit	-	-	-	-
-	Tag_211 CATGCACTCGGCCGAGCGGTCGGTGG	311	1	78.922	0.999	1.40E-29	78.973	no hit	-	-	-	-
Defense	Tag_61 CATGTTGCTGGCTGCTGCAGCCACCG	1474	5	374.055	4.997	2.26E-135	74.859	Hv_Contig_8456	1247	88-113	AFH57159|REPAT39 *Spodoptera exigua*	7.00E-30
Unknown	Tag_55 CATGAGAATGCTAAAACTGGGTGGTG	1857	7	471.248	6.996	5.57E-169	67.364	Hv_Contig_22902	646	221-246	ABH10141|HMG176 *Helicoverpa armigera*	7.00E-53
-	Tag_255 CATGGCAGCCTAGCCTCGCCCCGCGT	245	1	62.173	0.999	3.39E-23	62.213	no hit	-	-	-	-
-	Tag_293 CATGGTAGCCTAGCTTCGCCCCGCGT	215	1	54.560	0.999	2.65E-20	54.595	no hit	-	-	-	-
-	Tag_340 CATGGCAGCCTAGCTTCGCCCCACGT	180	1	45.678	0.999	6.14E-17	45.708	no hit	-	-	-	-
-	Tag_352 CATGGCAGCCTAGCTCCGCCCCGCGT	173	1	43.902	0.999	2.88E-16	43.930	no hit	-	-	-	-
-	Tag_392 CATGGCAACCTAGCTTCGCCCCGCGT	154	1	39.080	0.999	1.90E-14	39.105	no hit	-	-	-	-
-	Tag_414 CATGTGGTGGACTCCGCTGCTGAGCC	144	1	36.543	0.999	1.71E-13	36.566	no hit	-	-	-	-
-	Tag_416 CATGGCAGCCTATCTTCGCCCCGCGT	143	1	36.289	0.999	2.13E-13	36.312	no hit	-	-	-	-
-	Tag_241 CATGGCAGCCCAGCTTCGCCCCGCGT	272	2	69.025	1.999	2.36E-24	34.535	no hit	-	-	-	-
Nucleic acid binding	Tag_435 CATGCCGCGCGGCGCGGCGCGGCAGG	132	1	33.497	0.999	2.38E-12	33.519	Hv_Contig_11935	1025	846-871	XP_014367764|zinc finger protein 598 *Papilio machaon*	0.00E+00
-	Tag_436 CATGGCAGCCTAGCTTCGCCCTGCGT	132	1	33.497	0.999	2.38E-12	33.519	no hit	-	-	-	-
-	Tag_444 CATGGCAGCCTAGTTTCGCCCCGCGT	127	1	32.229	0.999	7.10E-12	32.249	no hit	-	-	-	-
-	Tag_279 CATGGCAGCCTAGCTTCGCCCCGTGT	229	2	58.113	1.999	2.84E-20	29.075	no hit	-	-	-	-
-	Tag_498 CATGCACGTGGCGCGCCAGGTGAGTG	107	1	27.153	0.999	5.57E-10	27.171	no hit	-	-	-	-
-	Tag_217 CATGAGGGCGCCAGACCCCGCCCTCG	304	3	77.146	2.998	4.43E-26	25.732	no hit	-	-	-	-
-	Tag_68 CATGCGCACGGCACGCAGAAGGGTGT	1291	13	327.615	12.992	5.18E-106	25.217	no hit	-	-	-	-
-	Tag_541 CATGTTGCGCTCTGGCTGGCCCAACG	98	1	24.869	0.999	3.93E-09	24.885	no hit	-	-	-	-
Unknown	Tag_544 CATGGAGGCGGACGGCGCGGGCGCCG	97	1	24.616	0.999	4.88E-09	24.631	Hv_Contig_35272	406	293-318	XP_011559079|uncharacterized protein LOC105389633 *Plutella xylostella*	2.00E-30
Unknown	Tag_550 CATGGCCGCGCCCGCGCACTGCGGCG	95	1	24.108	0.999	7.52E-09	24.123	Hv_Contig_368	4062	459-434	XP_014362832|cytoplasmic dynein 1 intermediate chain isoform X6 *Papilio machaon*	0.00E+00
-	Tag_235 CATGAGAATGCTAAAACTGGGTGGTA	284	3	72.070	2.998	3.35E-24	24.039	no hit	-	-	-	-
Protein folding/Recycling	Tag_326 CATGCGCGTGGACGCGCTGCCCGCGC	189	2	47.962	1.999	1.67E-16	23.997	Hv_Contig_7535	1329	1223-1248	XP_013135438|presenilin homolog *Papilio polytes*	8.00E-169
-	Tag_553 CATGCACTCGGCCGAGCAGCCGGTGG	94	1	23.854	0.999	9.34E-09	23.870	no hit	-	-	-	-
-	Tag_333 CATGACAGCCTAGCTTCGCCCCGCGT	185	2	46.947	1.999	3.96E-16	23.489	no hit	-	-	-	-
mRNA processing/splicing	Tag_335 CATGTCGCGCGGCCCGCGCCACCGCT	183	2	46.440	1.999	6.09E-16	23.235	Hv_Contig_2277	2278	2217-2242	XP_013188918|protein virilizer *Amyelois transitella*	0.00E+00
-	Tag_573 CATGCACTCGGCCGAGCGGCCGATGG	89	1	22.585	0.999	2.75E-08	22.600	no hit	-	-	-	-
-	Tag_344 CATGAGAATGCTAAAACTGGGTGGTC	176	2	44.663	1.999	2.75E-15	22.346	no hit	-	-	-	-
Unknown	Tag_87 CATGAGCCCCCTCCTGCCCGACACGT	866	10	219.764	9.994	5.36E-70	21.990	Hv_Contig_16164	842	517-542	XP_013135392|leukocyte surface antigen CD53-like isoform X1 *Papilio polytes*	7.00E-73
Unknown	Tag_18 CATGCGCACGGCACGCAGAAGGGTGA	8622	105	2187.993	104.933	0.00E+00	20.851	Hv_Contig_31364	498	360-385	CCF46246|hypothetical protein CH063_15059, partial *Colletotrichum higginsianum*	2.00E-14
-	Tag_12 CATGGCAGCCTAGCTTCGCCCCGCGC	11598	142	2943.209	141.909	0.00E+00	20.740	no hit	-	-	-	-
Unknown	Tag_125 CATGTGGCCGGGCTCGCATCGCAGTT	570	7	144.648	6.996	4.65E-46	20.677	Hv_Contig_5434	1575	809-834	XP_012546736|P3 protein-like *Bombyx mori*	1.00E-39
Cytoskeleton	Tag_606 CATGGAACGCGAGGCTGAGGACGCGT	81	1	20.555	0.999	1.54E-07	20.568	Hv_Contig_9943	1142	579-604	XP_013141160|tetraspanin-15-like *Papilio polytes*	1.00E-39
-	Tag_378 CATGCACTCGGCCGAGCGGCCAGTGG	161	2	40.857	1.999	6.91E-14	20.441	no hit	-	-	-	-
-	Tag_387 CATGCTTCTCAGATCGTAAATGGTTG	156	2	39.588	1.999	2.02E-13	19.807	no hit	-	-	-	-
-	Tag_110 CATGCGCACGGCACGCAGAAGGGTGC	700	9	177.638	8.994	9.33E-56	19.750	no hit	-	-	-	-
Translation/Ribosome biogenesis	Tag_393 CATGGCAGCCGCCGTCGTGTCAGGCA	153	2	38.827	1.999	3.83E-13	19.426	Hv_Contig_28542	553	124-149	NP_001298510|40S ribosomal protein S20 *Papilio polytes*	9.00E-81
-	Tag_281 CATGGCAGCCTGGCTTCGCCCCGCGT	228	3	57.859	2.998	5.62E-19	19.299	no hit	-	-	-	-
-	Tag_401 CATGCTCCTACAGATGTTCCGCTAAG	150	2	38.065	1.999	7.27E-13	19.045	no hit	-	-	-	-
-	Tag_291 CATGGCTGTTGAGCAGCGAAAGGACG	217	3	55.068	2.998	5.87E-18	18.368	no hit	-	-	-	-
Translation/Ribosome biogenesis	Tag_230 CATGGTCGCTTCCAGACGCCGGCTGA	289	4	73.339	3.997	1.72E-23	18.347	Hv_Contig_7237	1357	1195-1220	AAL62468|ribosomal protein L3 *Spodoptera frugiperda*	0.00E+00
-	Tag_651 CATGTACAACGACAAAATCCGTATCC	72	1	18.271	0.999	1.06E-06	18.283	no hit	-	-	-	-
-	Tag_36 CATGCGCACGGCACGCAGAAGGGTGG	3164	44	802.924	43.972	1.58E-242	18.260	no hit	-	-	-	-
-	Tag_295 CATGGGCGACCGTGCCGCTAGCGACG	215	3	54.560	2.998	8.98E-18	18.198	no hit	-	-	-	-
Primary metabolic process/oxidoreductase activity	Tag_653 CATGTCGGCGACCTCGGAAATGTCCT	71	1	18.018	0.999	1.31E-06	18.029	Hv_Contig_17230	806	340-365	ACY78421|diapause bioclock protein-like protein *Helicoverpa armigera*	7.00E-128
Unknown	Tag_3 CATGGCAGCCTAGCTTCGCCCCGCGT	103728	1501	26322.915	1500.037	0.00E+00	17.548	Hv_Contig_40219	299	64-89	ABH10141|HMG176 *Helicoverpa armigera*	2.00E-22
-	Tag_432 CATGCGCCGCTACGTGCGCGCACACG	134	2	34.005	1.999	2.19E-11	17.013	no hit	-	-	-	-
-	Tag_174 CATGGCGGCCTAGCTTCGCCCCGCGT	402	6	102.015	5.996	1.14E-31	17.013	no hit	-	-	-	-
-	Tag_679 CATGTGGCCGGGCTCGCATCGCAGTC	66	1	16.749	0.999	3.79E-06	16.759	no hit	-	-	-	-
Translation/Ribosome biogenesis	Tag_683 CATGGTTGGCCCGCGAGTCCTGAAGG	65	1	16.495	0.999	4.69E-06	16.506	Hv_Contig_20199	717	229-254	Q963B7|60S ribosomal protein L9 *Spodoptera frugiperda*	3.00E-130
-	Tag_140 CATGCGCACGGCACGCAGAAGGGTAA	516	8	130.945	7.995	6.36E-40	16.379	no hit	-	-	-	-
Transport/Trafficking	Tag_442 CATGTATCGTGTGGCAGTCGGCCGCA	128	2	32.482	1.999	7.80E-11	16.252	Hv_Contig_949	3061	2432-2457	XP_014363077|phosphatidylinositol transfer protein alpha isoform *Papilio machaon*	9.00E-166
-	Tag_684 CATGGCAGCCTAGCTTTGCCCCGCGT	64	1	16.241	0.999	5.80E-06	16.252	no hit	-	-	-	-
Protein folding/Recycling	Tag_451 CATGGAGCGGCTCGCGCTCTCCAACG	124	2	31.467	1.999	1.82E-10	15.744	Hv_Contig_16650	823	611-636	ADK55517.2|heat shock protein 90 cognate *Spodoptera litura*	4.00E-130
-	Tag_452 CATGTCGTGGACGCAGTTCCTCGAAC	124	2	31.467	1.999	1.82E-10	15.744	no hit	-	-	-	-
-	Tag_716 CATGGCAGCCTAGCTTCGCCCCGCAC	60	1	15.226	0.999	1.35E-05	15.236	no hit	-	-	-	-
-	Tag_221 CATGCGCGGGCGCGCCGGCCCGCCGA	296	5	75.116	4.997	4.79E-23	15.033	no hit	-	-	-	-
-	Tag_343 CATGAGAATGCTAAAACTGGGTGGCG	177	3	44.917	2.998	2.77E-14	14.982	no hit	-	-	-	-

-	Tag_472 CATGCATTCGGCCGAGCGGCCGGTGG	118	2	29.945	1.999	6.42E-10	14.982	no hit	-	-	-	-
Unknown	Tag_268 CATGTACGCGGCGCTGTGGCCCTTCC	234	4	59.382	3.997	1.92E-18	14.855	Hv_Contig_25956	593	65-90	EHJ71292|hypothetical protein KGM_01454 *Danaus plexippus*	8.00E-25
-	Tag_269 CATGCGCACGGCACGCAGAAGGGTAG	234	4	59.382	3.997	1.92E-18	14.855	no hit	-	-	-	-
-	Tag_347 CATGTTGTGCTCTGGCTGGAACGGCA	175	3	44.410	2.998	4.22E-14	14.813	no hit	-	-	-	-
-	Tag_732 CATGGCTGAAGGGAAGGAACACGCTC	58	1	14.719	0.999	2.06E-05	14.728	no hit	-	-	-	-
-	Tag_476 CATGCCCGCTGGTGGCGCCGCTCCCT	116	2	29.437	1.999	9.78E-10	14.728	no hit	-	-	-	-
-	Tag_734 CATGCAGGAGTGAGGGCCCTGGACGC	57	1	14.465	0.999	2.54E-05	14.474	no hit	-	-	-	-
-	Tag_741 CATGGCGGCCTAGCTTCGCCCCGCGG	56	1	14.211	0.999	3.14E-05	14.220	no hit	-	-	-	-
-	Tag_364 CATGGCAGTCTAGCTTCGCCCCGCGT	167	3	42.379	2.998	2.26E-13	14.136	no hit	-	-	-	-
-	Tag_180 CATGCACTCGGCCGAGCGACCGGTGG	387	7	98.208	6.996	3.14E-29	14.039	no hit	-	-	-	-
Primary metabolic process/hydrolase activity	Tag_492 CATGCAAGCTGCTGCCCCATTCTCCT	110	2	27.915	1.999	3.44E-09	13.966	Hv_Contig_7038	1378	1149-1174	CAA06419|carboxypeptidase A *Helicoverpa armigera*	0.00E+00
-	Tag_8 CATGGCAGCCTAGCTTCGCCCCGCGG	14940	273	3791.304	272.825	0.00E+00	13.896	no hit	-	-	-	-
-	Tag_368 CATGGCAGCCTAGCTTCACCCCGCGT	164	3	41.618	2.998	4.22E-13	13.882	no hit	-	-	-	-
-	Tag_761 CATGCACTCGGCCGTGCGGCCGGTGG	54	1	13.704	0.999	4.78E-05	13.712	no hit	-	-	-	-
-	Tag_373 CATGCGCACGGCACGCAGAAGGGTCA	162	3	41.111	2.998	6.41E-13	13.712	no hit	-	-	-	-
Chitin-binding	Tag_163 CATGACACGGGCGTGCGGAGGAGCAT	427	8	108.359	7.995	8.04E-32	13.554	Hv_Contig_35527	400	289-314	O02443|Larval cuticle protein 1 *Helicoverpa armigera*	4.00E-25
-	Tag_165 CATGCACTCGGCCGAGCGGCCGGCGG	424	8	107.598	7.995	1.50E-31	13.458	no hit	-	-	-	-
-	Tag_518 CATGACGCGGCGCGTGCGCGCATCTC	104	2	26.392	1.999	1.20E-08	13.204	no hit	-	-	-	-
Primary metabolic process/hydrolase activity	Tag_310 CATGAGCTGGATCAACAGACATCTGT	206	4	52.276	3.997	6.62E-16	13.077	Hv_Contig_42719	259	150-175	ACR15971.2|serine protease 37 *Mamestra configurata*	1.00E-12
-	Tag_396 CATGCACTCGGCCAAGCGGCCGGTGG	152	3	38.573	2.998	5.13E-12	12.866	no hit	-	-	-	-
Transport/Trafficking	Tag_527 CATGAAACTTGAAGGTTGGGATGGCG	101	2	25.631	1.999	2.24E-08	12.824	Hv_Contig_32968	462	383-408	ACB54948|fatty acid-binding protein 1 *Helicoverpa armigera*	6.00E-70
-	Tag_529 CATGCGCGTGGCGGACGGGCTCGGCG	101	2	25.631	1.999	2.24E-08	12.824	no hit	-	-	-	-
-	Tag_533 CATGGCAGCCTAGCTTCGTCCCGCGT	100	2	25.377	1.999	2.76E-08	12.697	no hit	-	-	-	-
-	Tag_537 CATGAGCCCCCTCCTGCCCGACACGC	100	2	25.377	1.999	2.76E-08	12.697	no hit	-	-	-	-
-	Tag_790 CATGCACACGGCCGAGCGGCCGGTGG	50	1	12.688	0.999	1.10E-04	12.697	no hit	-	-	-	-
-	Tag_797 CATGATCTGTGCCGGTTGGCCCAGCG	49	1	12.435	0.999	1.36E-04	12.443	no hit	-	-	-	-
Translation/Ribosome biogenesis	Tag_799 CATGGGAGGGTGTCACAGGTTCCTCT	49	1	12.435	0.999	1.36E-04	12.443	Hv_Contig_14723	898	769-794	XP_012552995|39S ribosomal protein L16, mitochondrial *Bombyx mori*	4.00E-132
Protein folding/Recycling	Tag_548 CATGGACGACTGGCCCTCGGTGCGCC	96	2	24.362	1.999	6.32E-08	12.189	Hv_Contig_1209	2843	2010-2035	XP_013183026|ATP-dependent Clp protease ATP-binding subunit clpX-like, mitochondrial isoform X3 *Amyelois transitella*	0.00E+00
Extracellular matrix structural constituent	Tag_805 CATGGGACGCGGGTGAAACCGCTGGC	48	1	12.181	0.999	1.67E-04	12.189	Hv_Contig_11489	1050	903-878	KOB70085|putative collagen alpha-2 IV chain protein *Operophtera brumata*	1.00E-32
Translation/Ribosome biogenesis	Tag_812 CATGAGACATTCTAAGAATGTCGGTC	47	1	11.927	0.999	2.06E-04	11.935	Hv_Contig_34007	437	114-139	AAK59928|ribosomal protein S11 *Heliothis virescens*	1.00E-67
-	Tag_815 CATGATCTGTGCCGGTTGGTCCAGCT	47	1	11.927	0.999	2.06E-04	11.935	no hit	-	-	-	-
-	Tag_818 CATGGAGTCCTCGTTGGTGTCACCTT	47	1	11.927	0.999	2.06E-04	11.935	no hit	-	-	-	-
-	Tag_236 CATGGCAGCCTAACTTCGCCCCGCGT	277	6	70.294	5.996	2.49E-20	11.723	no hit	-	-	-	-
mRNA processing/splicing	Tag_561 CATGGGCGGGCGCGGCGGCCGCGGCG	92	2	23.347	1.999	1.44E-07	11.681	Hv_Contig_671	3422	733-758	XP_013190420|RNA-binding protein squid isoform X2 *Amyelois transitella*	3.00E-122
Primary metabolic process/hydrolase activity	Tag_119 CATGGAGTCCTCGTTGGTGTCACCTC	597	13	151.500	12.992	4.20E-42	11.661	Hv_Contig_21108	693	568-593	AFO68320|trypsin *Heliothis virescens*	4.00E-119
-	Tag_427 CATGTTTTTTATTTCTCTCTGTACAC	137	3	34.766	2.998	1.14E-10	11.596	Hv_Contig_5986	1496	379-354	no hit	-
-	Tag_829 CATGGCAGCCCAGCTTCGCCCCGCGG	45	1	11.420	0.999	3.11E-04	11.427	no hit	-	-	-	-
-	Tag_209 CATGCGCTCGGCCGAGCGGCCGGTGG	314	7	79.683	6.996	1.13E-22	11.391	no hit	-	-	-	-
-	Tag_571 CATGGTCCAATAAATTCTTTGGGTCA	89	2	22.585	1.999	2.68E-07	11.300	no hit	-	-	-	-
-	Tag_838 CATGGCGGCCTAGCTTCGCCCCGCGC	44	1	11.166	0.999	3.82E-04	11.173	no hit	-	-	-	-
-	Tag_579 CATGGCAGCCTAGCTTCGCCTCGCGT	88	2	22.332	1.999	3.29E-07	11.173	no hit	-	-	-	-
-	Tag_98 CATGCACTCGGCCGAGCGGCCGGTGT	789	18	200.223	17.988	2.31E-54	11.131	no hit	-	-	-	-
-	Tag_851 CATGTTGTGCTCTGGCCGGCCCAGCG	42	1	10.658	0.999	5.77E-04	10.665	no hit	-	-	-	-
-	Tag_853 CATGCGCCGCGGTGGGCCCGCGCCTG	42	1	10.658	0.999	5.77E-04	10.665	no hit	-	-	-	-
-	Tag_366 CATGCCCGCTGGTGGCGCCGCTCCTG	166	4	42.126	3.997	2.47E-12	10.538	no hit	-	-	-	-
-	Tag_866 CATGCTGCAGCGCGCGCCGGGCGTGG	41	1	10.405	0.999	7.09E-04	10.411	no hit	-	-	-	-
-	Tag_158 CATGGCAGCCTAGCTTCGCCCCGCAT	448	11	113.688	10.993	8.44E-31	10.342	no hit	-	-	-	-
-	Tag_256 CATGCACCCGGCCGAGCGGCCGGTGG	244	6	61.920	5.996	2.09E-17	10.327	no hit	-	-	-	-
-	Tag_607 CATGGGTCGGCAATCCTCTTGGACTC	81	2	20.555	1.999	1.37E-06	10.284	no hit	-	-	-	-
Primary metabolic process/isomerase activity	Tag_188 CATGTGGGCAGACGCCACGCTCGCTA	361	9	91.610	8.994	5.61E-25	10.185	Hv_Contig_7598	1323	1097-1122	XP_012552008|UDP-glucose 4-epimerase-like *Bombyx mori*	0.00E+00
-	Tag_463 CATGCACTCGGCCGAACGGCCGGTGG	120	3	30.452	2.998	3.65E-09	10.157	no hit	-	-	-	-
-	Tag_260 CATGCCGGAGTCACCCGTGTCGGAGC	240	6	60.904	5.996	4.69E-17	10.157	no hit	-	-	-	-
-	Tag_615 CATGTTGTGCTCTGGCTGGCCCGACG	80	2	20.301	1.999	1.68E-06	10.157	no hit	-	-	-	-
-	Tag_883 CATGCCGTCGGCCGCAGGTGAAGCAG	39	1	9.897	0.999	1.07E-03	9.903	no hit	-	-	-	-
Translation/Ribosome biogenesis	Tag_888 CATGAAACAGGGAGTCCTCACCAACA	38	1	9.643	0.999	1.31E-03	9.649	Hv_Contig_12709	870	242-267	Q95V32|40S ribosomal protein S6 *Spodoptera frugiperda*	7.00E-161
Translation/Ribosome biogenesis	Tag_195 CATGGTCCAATAAATTCTTTGGGTCG	341	9	86.535	8.994	3.15E-23	9.621	Hv_Contig_14879	892	703-728	NP_001298530|60S ribosomal protein L19 *Papilio polytes*	9.00E-95
-	Tag_62 CATGCACTCGGCCGAGCGGCCGGTGC	1446	39	366.949	38.975	9.51E-93	9.415	no hit	-	-	-	-
-	Tag_913 CATGCGCACGGCACGCAGAAGGGTTA	37	1	9.389	0.999	1.60E-03	9.395	no hit	-	-	-	-
-	Tag_646 CATGACGCGGCGCGTGCGCGCATCTA	73	2	18.525	1.999	6.93E-06	9.269	no hit	-	-	-	-
Protein folding/Recycling	Tag_80 CATGCCCGGCGGTATGCCCGGCGGCA	1046	29	265.442	28.981	7.16E-67	9.159	Hv_Contig_4349	1752	1532-1557	AIZ00749|heat shock cognate 70 protein, partial *Sesamia inferens*	0.00E+00
-	Tag_917 CATGATCCACTGTCTCGCGCGCCGTG	36	1	9.136	0.999	1.96E-03	9.142	no hit	-	-	-	-
-	Tag_920 CATGTTGTGCTCCGGCTGGCCCAGCG	36	1	9.136	0.999	1.96E-03	9.142	no hit	-	-	-	-
-	Tag_232 CATGCACTCGGCCGAGTGGCCGGTGG	287	8	72.832	7.995	2.10E-19	9.110	no hit	-	-	-	-
-	Tag_655 CATGCCGGCGCCAGCGGCGGTGGCGG	71	2	18.018	1.999	1.04E-05	9.015	no hit	-	-	-	-
-	Tag_658 CATGCACTCGGCGGAGCGGCCGGTGG	71	2	18.018	1.999	1.04E-05	9.015	no hit	-	-	-	-
Nucleic acid binding	Tag_942 CATGTGCCGCGGCCTGTGCGGCGAGC	35	1	8.882	0.999	2.40E-03	8.888	Hv_Contig_1945	2412	1901-1876	XP_013143932|NFX1-type zinc finger-containing protein 1-like *Papilio polytes*	4.00E-160
-	Tag_949 CATGGCTGCGGCCCAGGGAGGACGCT	35	1	8.882	0.999	2.40E-03	8.888	no hit	-	-	-	-

-	Tag_659 CATGTTGCTGGCTGCTGCAGCCACCA	70	2	17.764	1.999	1.27E-05	8.888	no hit	-	-	-	-
Translation/Ribosome biogenesis	Tag_50 CATGCCCGCTGGTGGCGCCGCTCCCG	2016	58	511.598	57.963	1.88E-125	8.826	Hv_Contig_27905	563	285-310	KPJ13320|60S acidic ribosomal protein P2 *Papilio machaon*	2.00E-33
-	Tag_422 CATGAGCCCCCTCCTGCCCGACACGG	139	4	35.274	3.997	5.65E-10	8.824	no hit	-	-	-	-
Unknown	Tag_2 CATGCACTCGGCCGAGCGGCCGGTGG	123487	3575	31337.130	3572.707	0.00E+00	8.771	Hv_Contig_47003	206	115-140	KPJ15203|hypothetical protein RR48_09230 *Papilio machaon*	9.00E-24
-	Tag_668 CATGACACGGGCGTGCGGAGGAGCAG	68	2	17.256	1.999	1.89E-05	8.634	no hit	-	-	-	-
-	Tag_38 CATGCACTCGGTCGAGCGGCCGGTGG	3077	91	780.846	90.942	2.19E-188	8.586	Hv_Contig_5683	1540	1460-1485	no hit	-
Primary metabolic process/hydrolase activity	Tag_270 CATGATCTGCGCAGGATGGCTCGATG	233	7	59.128	6.996	1.37E-15	8.452	Hv_Contig_15523	867	627-652	XP_004931376|trypsin, alkaline A-like *Bombyx mori*	1.00E-44
-	Tag_976 CATGACATTGGTTTCAAGAAGCTTGG	33	1	8.374	0.999	3.59E-03	8.380	no hit	-	-	-	-
-	Tag_979 CATGCTGCAGCGCGCGCTGGGCGTTG	33	1	8.374	0.999	3.59E-03	8.380	no hit	-	-	-	-
-	Tag_981 CATGCCCGCTGGTGGCGCCGCTCTCG	33	1	8.374	0.999	3.59E-03	8.380	no hit	-	-	-	-
-	Tag_985 CATGATCTGCGCCGGCTGGCCCAGCG	33	1	8.374	0.999	3.59E-03	8.380	no hit	-	-	-	-
-	Tag_203 CATGCACTCGGCTGAGCGGCCGGTGG	326	10	82.729	9.994	4.38E-21	8.278	no hit	-	-	-	-
-	Tag_204 CATGCACTCGACCGAGCGGCCGGTGG	325	10	82.475	9.994	5.33E-21	8.253	no hit	-	-	-	-
Primary metabolic process/hydrolase activity	Tag_996 CATGTTGTGCGCCGGTGTGCTCGATG	32	1	8.121	0.999	4.39E-03	8.126	Hv_Contig_16622	824	601-626	AFO68320|trypsin *Heliothis virescens*	6.00E-97
-	Tag_1001 CATGGCAGCCTAGCTTCACCCCGCGG	32	1	8.121	0.999	4.39E-03	8.126	no hit	-	-	-	-
-	Tag_695 CATGCGCGGGCGCGCCGGCCCGCCGG	63	2	15.987	1.999	5.10E-05	7.999	no hit	-	-	-	-
-	Tag_170 CATGGCGGCGTCACACCACGCTGTGG	405	13	102.776	12.992	2.27E-25	7.911	no hit	-	-	-	-
-	Tag_1008 CATGCGGCGGTGACTCCGGAGGCCCT	31	1	7.867	0.999	5.36E-03	7.872	no hit	-	-	-	-
-	Tag_1012 CATGCGCACGGCACGCGGAAGGGTGA	31	1	7.867	0.999	5.36E-03	7.872	no hit	-	-	-	-
-	Tag_1014 CATGAGAATGCTAAAACTGGGTGGAG	31	1	7.867	0.999	5.36E-03	7.872	no hit	-	-	-	-
-	Tag_701 CATGTTGTGCTCTGGCTGACCCAACG	62	2	15.734	1.999	6.21E-05	7.872	no hit	-	-	-	-
-	Tag_89 CATGACGCGGCGCGTGCGCGCATCTT	859	28	217.987	27.982	1.02E-51	7.790	no hit	-	-	-	-
-	Tag_297 CATGCTGGCGGATCACGTCGCCAGGC	214	7	54.306	6.996	5.69E-14	7.763	no hit	-	-	-	-
-	Tag_710 CATGTTGTGCTCTGGTTGGCCCAACG	61	2	15.480	1.999	7.56E-05	7.745	no hit	-	-	-	-
-	Tag_712 CATGGGCAGAGACGTTTCCAACGAGC	61	2	15.480	1.999	7.56E-05	7.745	no hit	-	-	-	-
Unknown	Tag_177 CATGTCGGACGCGCTACGCTCATCGG	391	13	99.224	12.992	3.45E-24	7.637	Hv_Contig_21487	682	580-605	XP_015035128|uncharacterized protein Dpse_GA32788 *Drosophila pseudoobscura pseudoobscura*	2.00E-68
-	Tag_1033 CATGACGCGGCGCGTGCGCGCATCCT	30	1	7.613	0.999	6.54E-03	7.618	no hit	-	-	-	-
-	Tag_1036 CATGCACTCGGCCGAGCGACCGGTGA	30	1	7.613	0.999	6.54E-03	7.618	no hit	-	-	-	-
Primary metabolic process/hydrolase activity	Tag_468 CATGGCGCGCGGGCTGGTGGCGCCGC	119	4	30.198	3.997	2.91E-08	7.554	Hv_Contig_8166	1270	1201-1226	EHJ71294|AAA family ATPase *Danaus plexippus*	2.00E-178
Unknown	Tag_569 CATGAGAGGTGTAGCATAAGTGGGAG	89	3	22.585	2.998	1.76E-06	7.533	Hv_Contig_25755	595	33-58	XP_002426744|conserved hypothetical protein *Pediculus humanus corporis*	3.00E-27
-	Tag_724 CATGGAGTCCTCGTTGGTGTCACCTA	59	2	14.972	1.999	1.12E-04	7.491	no hit	-	-	-	-
-	Tag_1051 CATGCACTCGGCCGAGCGGCCGGTAT	29	1	7.359	0.999	7.97E-03	7.364	no hit	-	-	-	-
-	Tag_1053 CATGCACTCGGCCGAGCGGCCGGTAC	29	1	7.359	0.999	7.97E-03	7.364	no hit	-	-	-	-
-	Tag_1055 CATGGGTCGGCAATCCTCTCGGACCT	29	1	7.359	0.999	7.97E-03	7.364	no hit	-	-	-	-
-	Tag_733 CATGCTGGCAGTCGTAGCTGCTAACG	58	2	14.719	1.999	1.36E-04	7.364	no hit	-	-	-	-
-	Tag_96 CATGCACTCGGCCGAGCGGCCGGTAG	808	28	205.045	27.982	1.97E-47	7.328	no hit	-	-	-	-
-	Tag_349 CATGAGAATGCTAAAACTGGGTGGGG	173	6	43.902	5.996	2.74E-11	7.322	no hit	-	-	-	-
-	Tag_249 CATGCACTCGGCCGAGCGGCTGGTGG	257	9	65.219	8.994	4.85E-16	7.251	no hit	-	-	-	-
-	Tag_484 CATGTTGCCCAGGTGTACGCTATCAG	113	4	28.676	3.997	9.31E-08	7.174	no hit	-	-	-	-
-	Tag_485 CATGTTGTGCTCCGGCTGGCCCAACG	112	4	28.422	3.997	1.13E-07	7.110	no hit	-	-	-	-
-	Tag_1074 CATGCTCGCAGTCGTAGCTGCTAACG	28	1	7.106	0.999	9.71E-03	7.110	no hit	-	-	-	-
-	Tag_1081 CATGCGCGCCGGCTCGCTGGTCGGCA	28	1	7.106	0.999	9.71E-03	7.110	no hit	-	-	-	-
Translation/Ribosome biogenesis	Tag_290 CATGGCAAGCGTGTTCGTAAGATTCG	218	8	55.322	7.995	1.53E-13	6.920	Hv_Contig_18998	729	469-494	ABS57457|ribosomal protein L23a *Heliconius melpomene*	2.00E-32
Primary metabolic process/hydrolase activity	Tag_69 CATGTTGTGCTCTGGCTGGAACGGTG	1273	48	323.047	47.969	8.47E-71	6.734	Hv_Contig_40932	287	33-58	ABW37098|putative chymotrypsin-like proteinase *Heliothis virescens*	4.00E-29
-	Tag_765 CATGGCTGCCTAGCTTCGCCCCGCGT	53	2	13.450	1.999	3.59E-04	6.729	no hit	-	-	-	-
Translation/Ribosome biogenesis	Tag_766 CATGACATTGGTTTCAAGAAGCTTGA	53	2	13.450	1.999	3.59E-04	6.729	Hv_Contig_33509	449	255-280	KPI92256|40S ribosomal protein S29 *Papilio xuthus*	5.00E-32
-	Tag_771 CATGGAGTCCTCGTTGGTGTCACCTG	53	2	13.450	1.999	3.59E-04	6.729	no hit	-	-	-	-
Unknown	Tag_515 CATGTCGTACGAGAGCAGTCCGTTCC	105	4	26.646	3.997	4.33E-07	6.666	Hv_Contig_2018	2379	1953-1978	XP_013176241|uncharacterized protein LOC106124291 *Papilio xuthus*	0.00E+00
-	Tag_775 CATGCCGCCACCGCGGTGCCACCGCA	52	2	13.196	1.999	4.36E-04	6.602	no hit	-	-	-	-
-	Tag_1120 CATGTTCGTCAGGGTCAGCGCTTACG	26	1	6.598	0.999	1.44E-02	6.602	no hit	-	-	-	-
-	Tag_1139 CATGACAATGTAACATCTTAAATTGC	26	1	6.598	0.999	1.44E-02	6.602	no hit	-	-	-	-
Translation/Ribosome biogenesis	Tag_783 CATGCCGCGTGAAATCAAAGATATCA	51	2	12.942	1.999	5.28E-04	6.475	Hv_Contig_36338	380	105-130	EHJ67727|60S ribosomal protein L38 *Danaus plexippus*	2.00E-39
-	Tag_1144 CATGACCATTTTGTGTGAGGAGGTGG	25	1	6.344	0.999	1.75E-02	6.348	no hit	-	-	-	-
-	Tag_1151 CATGCTGCAGCGCGCGCTGAGCGTGG	25	1	6.344	0.999	1.75E-02	6.348	no hit	-	-	-	-
-	Tag_1168 CATGACCCTAACTACCCGCCACCGCC	25	1	6.344	0.999	1.75E-02	6.348	no hit	-	-	-	-
Primary metabolic process/hydrolase activity	Tag_403 CATGGACAGTACTCGCAATGTTGCTA	149	6	37.812	5.996	2.68E-09	6.306	Hv_Contig_20720	703	619-644	AAK85539|aminopeptidase N *Helicoverpa armigera*	3.00E-23
Primary metabolic process/hydrolase activity	Tag_219 CATGCTGGCAGTCGTAGCTGCTAACA	298	12	75.623	11.992	3.21E-17	6.306	Hv_Contig_11142	1070	940-965	ACL36827|trypsin 2 *Helicoverpa armigera*	7.00E-151
-	Tag_540 CATGGACGTGGCCACGCGCCGCGCCG	98	4	24.869	3.997	1.63E-06	6.221	no hit	-	-	-	-
-	Tag_793 CATGCTGCAGCGCGCGCTGGGCGTAA	49	2	12.435	1.999	7.74E-04	6.221	no hit	-	-	-	-
-	Tag_796 CATGTTGTGCTCTGGCTGGTCCAACG	49	2	12.435	1.999	7.74E-04	6.221	no hit	-	-	-	-
Transport/Trafficking	Tag_649 CATGGGCTCCGTCTCTAGGGCTGCAG	72	3	18.271	2.998	4.60E-05	6.094	Hv_Contig_10316	1117	736-761	KOB71518|Protein transport protein sec13 *Operophtera brumata*	0.00E+00
-	Tag_650 CATGTGCAGTGTGAGGATTCTGTGTT	72	3	18.271	2.998	4.60E-05	6.094	Hv_Contig_13605	945	840-865	no hit	-
-	Tag_154 CATGCCCGCTGGTGGCGCCGCTCCCA	456	19	115.719	18.988	4.16E-25	6.094	no hit	-	-	-	-
-	Tag_1187 CATGGAGGACGCCGCAGCGCCCATGC	24	1	6.090	0.999	2.12E-02	6.094	no hit	-	-	-	-
-	Tag_1188 CATGAGCCGGCTGAGCAAACAGCCGT	24	1	6.090	0.999	2.12E-02	6.094	no hit	-	-	-	-
Translation/Ribosome biogenesis	Tag_1195 CATGTGCGCGACCACCCGCGTCATCT	24	1	6.090	0.999	2.12E-02	6.094	Hv_Contig_6836	1399	1105-1130	AEB26319|seryl-tRNA synthetase *Helicoverpa armigera*	0.00E+00
-	Tag_1196 CATGGAGGACGCGGCAGCGCCCACGC	24	1	6.090	0.999	2.12E-02	6.094	no hit	-	-	-	-

-	Tag_1198 CATGCTCCGTTAATTAACCAATACCC	24	1	6.090	0.999	2.12E-02	6.094	no hit	-	-	-	-
-	Tag_24 CATGCACTCGGCCGAGCGGCCGGTGA	5396	226	1369.336	225.855	9.96E-278	6.063	no hit	-	-	-	-
-	Tag_811 CATGCGAAGCCGGGGCGGGTCGCTTG	47	2	11.927	1.999	1.13E-03	5.967	no hit	-	-	-	-
-	Tag_557 CATGCTGTGCTCTGGCTGGCCCAACG	93	4	23.600	3.997	4.17E-06	5.904	no hit	-	-	-	-
-	Tag_186 CATGGGCTGAGGACGCCGCGCCACCG	370	16	93.894	15.990	2.95E-20	5.872	no hit	-	-	-	-
Primary metabolic process/transferase activity	Tag_37 CATGTCCTGCGCACGCGTGGCGCCCC	3131	136	794.550	135.913	1.28E-158	5.846	Hv_Contig_24145	622	380-405	AMK97473|UGT40D2v2 *Helicoverpa armigera*	2.00E-110
-	Tag_1204 CATGACGGGGGACGGGTCTCCCGCCT	23	1	5.837	0.999	2.57E-02	5.840	no hit	-	-	-	-
-	Tag_1207 CATGGCAGCCGCCGTCGTGTCAGGCG	23	1	5.837	0.999	2.57E-02	5.840	no hit	-	-	-	-
-	Tag_1212 CATGTGGCGGTGACTCCGGTGGCCCG	23	1	5.837	0.999	2.57E-02	5.840	no hit	-	-	-	-
-	Tag_1214 CATGCGCACGACACGCAGAAGGGTGA	23	1	5.837	0.999	2.57E-02	5.840	no hit	-	-	-	-
Primary metabolic process/oxidoreductase activity	Tag_1218 CATGCAGCTCAACGACCAGTTCCGAC	23	1	5.837	0.999	2.57E-02	5.840	Hv_Contig_21281	688	416-441	XP_013192952|inactive hydroxysteroid dehydrogenase-like protein 1 isoform X2 *Amyelois transitella*	2.00E-76
-	Tag_1230 CATGGACGGGCTCCCGGTGCCGCGCG	23	1	5.837	0.999	2.57E-02	5.840	no hit	-	-	-	-
-	Tag_1231 CATGCACGCGGCCGAGCGGCCGGTGG	23	1	5.837	0.999	2.57E-02	5.840	no hit	-	-	-	-
-	Tag_1235 CATGCGGCGGTGACCCCGGTGGCCCC	23	1	5.837	0.999	2.57E-02	5.840	no hit	-	-	-	-
-	Tag_336 CATGGCGGCCGCAAAAGAAGCAATGG	182	8	46.186	7.995	1.33E-10	5.777	no hit	-	-	-	-
-	Tag_674 CATGCGCACGGCACGCAGAAGGGTAT	67	3	17.002	2.998	1.17E-04	5.671	no hit	-	-	-	-
Translation/Ribosome biogenesis	Tag_386 CATGCGGCCTGGAGATGCCCGATCTC	156	7	39.588	6.996	3.48E-09	5.659	Hv_Contig_27404	571	425-450	XP_013195182|translation machinery-associated protein 16 homolog *Amyelois transitella*	53-88
-	Tag_570 CATGTCGCTGGGGGAGAAGCTGGAGT	89	4	22.585	3.997	8.78E-06	5.650	no hit	-	-	-	-
Protein folding/Recycling	Tag_1248 CATGATTGCCCTGCCCCCGCCCCGCC	22	1	5.583	0.999	3.11E-02	5.586	Hv_Contig_8821	1220	697-722	XP_011564371|ubiquitin-conjugating enzyme E2-24 kDa isoform X1 *Plutella xylostella*	3.00E-105
-	Tag_1249 CATGGACGAGCTCCCGGTGCCGCACG	22	1	5.583	0.999	3.11E-02	5.586	no hit	-	-	-	-
mRNA processing/splicing	Tag_1251 CATGAGTTCTGGTGGTACTCGCGTGT	22	1	5.583	0.999	3.11E-02	5.586	Hv_Contig_16399	832	120-145	XP_013185327|RNA-binding protein Rsf1 *Amyelois transitella*	8.00E-41
-	Tag_1253 CATGAGGACGACTTTGAAGACGTGAC	22	1	5.583	0.999	3.11E-02	5.586	no hit	-	-	-	-
Nucleic acid binding	Tag_1259 CATGTGTCAGCGTTCGTGCCGGTGCA	22	1	5.583	0.999	3.11E-02	5.586	Hv_Contig_12568	993	364-339	XP_004928597.2|zyxin *Bombyx mori*	3.00E-64
-	Tag_1265 CATGCTGTGCTCTGGCTGGCCCAGCG	22	1	5.583	0.999	3.11E-02	5.586	no hit	-	-	-	-
-	Tag_56 CATGGCAGCCTAGCTTCGCCCCGCGA	1745	81	442.826	80.948	9.53E-86	5.471	no hit	-	-	-	-
-	Tag_499 CATGCTGTGGATCATCGGCCGCTAGC	107	5	27.153	4.997	1.41E-06	5.434	no hit	-	-	-	-
-	Tag_353 CATGTACTCGGCCGAGCGGCCGGTGG	171	8	43.394	7.995	1.00E-09	5.428	no hit	-	-	-	-
-	Tag_589 CATGCCTGCTGGTGGTGCCGCTCCCA	85	4	21.570	3.997	1.83E-05	5.396	no hit	-	-	-	-
-	Tag_1278 CATGCACGAGCGAGGGCTCGGCCTGA	21	1	5.329	0.999	3.76E-02	5.333	no hit	-	-	-	-
Translation/Ribosome biogenesis	Tag_1282 CATGTTGAGTCTGGCGACCGTGCCTC	21	1	5.329	0.999	3.76E-02	5.333	Hv_Contig_12933	975	747-772	ABX55885|ribosomal protein L7A *Spodoptera exigua*	1.00E-136
-	Tag_1283 CATGCTGCAGCGCGCGCTGGGCATGG	21	1	5.329	0.999	3.76E-02	5.333	no hit	-	-	-	-
-	Tag_1293 CATGCGCCGCGGTGGGCCCGCGCCTA	21	1	5.329	0.999	3.76E-02	5.333	no hit	-	-	-	-
-	Tag_1301 CATGGAGGACGCTGCAGCGCCCACGC	21	1	5.329	0.999	3.76E-02	5.333	no hit	-	-	-	-
-	Tag_849 CATGCGGCGGTAACTCCGGTGGCCCT	42	2	10.658	1.999	2.88E-03	5.333	no hit	-	-	-	-
Unknown	Tag_273 CATGCTCCGTTAATTAACCAATACTC	231	11	58.621	10.993	1.58E-12	5.333	Hv_Contig_5986	1496	448-423	no hit	-
Nucleic acid binding	Tag_118 CATGCGCGCCGGCTCGCTGGTCGGCC	621	30	157.590	29.981	7.36E-31	5.256	Hv_Contig_3143	2001	1530-1555	EHJ74904|putative DNA repair protein xp-e *Danaus plexippus*	0.00E+00
-	Tag_699 CATGTTGTGCTCTGGCTGGCCCAGCC	62	3	15.734	2.998	2.96E-04	5.248	no hit	-	-	-	-
-	Tag_604 CATGTGGTGGTGCGATGCGATGAGCT	82	4	20.809	3.997	3.17E-05	5.206	no hit	-	-	-	-
Translation/Ribosome biogenesis	Tag_216 CATGCCTGCTGGTGGTGCCGCTCCCG	305	15	77.399	14.990	8.97E-16	5.163	Hv_Contig_27713	566	277-252	AAL62467|60S acidic ribosomal protein P2 *Spodoptera frugiperda*	5.00E-27
-	Tag_528 CATGTTGTGCCCTGGCTGGCCCAACG	101	5	25.631	4.997	4.19E-06	5.129	no hit	-	-	-	-
Primary metabolic process/hydrolase activity	Tag_166 CATGTTGTGCGCTGGTATCTTAGACG	420	21	106.583	20.987	6.19E-21	5.079	Hv_Contig_13773	938	634-659	AFO68325|trypsin *Heliothis virescens*	2.00E-180
-	Tag_1311 CATGCTGCTGGGATGCGCTACCTCTA	20	1	5.075	0.999	4.54E-02	5.079	no hit	-	-	-	-
-	Tag_1319 CATGGACGAGCCCCCGGTGCCGCGCG	20	1	5.075	0.999	4.54E-02	5.079	no hit	-	-	-	-
-	Tag_1321 CATGCGAAGGCTAAAGCGTAAAAGGT	20	1	5.075	0.999	4.54E-02	5.079	no hit	-	-	-	-
-	Tag_1325 CATGGCGGCCGCAAAAGAAGCAATGA	20	1	5.075	0.999	4.54E-02	5.079	no hit	-	-	-	-
-	Tag_1326 CATGCGCCGCGGTGGGCCCGCGCCTC	20	1	5.075	0.999	4.54E-02	5.079	no hit	-	-	-	-
-	Tag_1329 CATGCGCACGGCACGCAGAAGGGTCT	20	1	5.075	0.999	4.54E-02	5.079	no hit	-	-	-	-
-	Tag_1332 CATGCGGCGGTGACTCCGGTGACCCG	20	1	5.075	0.999	4.54E-02	5.079	no hit	-	-	-	-
-	Tag_1334 CATGGCAGCCAAGCTTCGCCCCGCGT	20	1	5.075	0.999	4.54E-02	5.079	no hit	-	-	-	-
-	Tag_1338 CATGAGCCCCCTCCTGCCCGACACGA	20	1	5.075	0.999	4.54E-02	5.079	no hit	-	-	-	-
-	Tag_1343 CATGCTGCAGCACGTCACGCGCACAC	20	1	5.075	0.999	4.54E-02	5.079	no hit	-	-	-	-
-	Tag_1349 CATGGCAGCCTAGCTTCGCCCCGCCT	20	1	5.075	0.999	4.54E-02	5.079	no hit	-	-	-	-
-	Tag_612 CATGCGCGTGGCGGACGGGCTCGGCC	80	4	20.301	3.997	4.56E-05	5.079	no hit	-	-	-	-
-	Tag_617 CATGGGTCGGCAATCCTCTTGGACTA	80	4	20.301	3.997	4.56E-05	5.079	no hit	-	-	-	-
-	Tag_319 CATGCCCGCTGGTGGCGCCGCTCCCC	199	10	50.500	9.994	1.17E-10	5.053	no hit	-	-	-	-
-	Tag_471 CATGGGGCTGGACATCCCGCCGCCCG	118	6	29.945	5.996	8.06E-07	4.994	no hit	-	-	-	-
-	Tag_629 CATGCGGCGATGACTCCGGTGGCCCT	78	4	19.794	3.997	6.54E-05	4.952	no hit	-	-	-	-
-	Tag_877 CATGCTCCGTTAATTAACCAATACGC	39	2	9.897	1.999	5.01E-03	4.952	no hit	-	-	-	-
-	Tag_272 CATGCACTCGGCCGGGCGGCCGGTGG	231	12	58.621	11.992	6.65E-12	4.888	no hit	-	-	-	-
-	Tag_280 CATGCAAGCGCTGCAAGAGGACCGCA	229	12	58.113	11.992	9.47E-12	4.846	no hit	-	-	-	-
-	Tag_885 CATGCTGCAGTGCGCGCTGGGCGTGG	38	2	9.643	1.999	6.01E-03	4.825	no hit	-	-	-	-
-	Tag_896 CATGCTCGTGCAAAGCCCCGCTTGCC	38	2	9.643	1.999	6.01E-03	4.825	no hit	-	-	-	-
-	Tag_897 CATGTTGTGCTCTGGCTGGAACGGTC	38	2	9.643	1.999	6.01E-03	4.825	no hit	-	-	-	-
Translation/Ribosome biogenesis	Tag_57 CATGGGCTGAGGACGCCGCGCCACCA	1676	89	425.316	88.943	2.26E-75	4.782	Hv_Contig_11605	1043	833-858	XP_014367750|40S ribosomal protein SA *Papilio machaon*	3.00E-162
-	Tag_489 CATGTCGTGCTCTGGCTGGCCCAACG	111	6	28.168	5.996	2.80E-06	4.698	no hit	-	-	-	-
-	Tag_906 CATGTTGCGCTCTGGCTGGCCCAGCG	37	2	9.389	1.999	7.20E-03	4.698	no hit	-	-	-	-
-	Tag_563 CATGGCAGCCTAGCTTCGCCCCGCAG	92	5	23.347	4.997	2.10E-05	4.672	no hit	-	-	-	-

-	Tag_316 CATGTCCTGCGCACGCGTGGCGCCCT	202	11	51.261	10.993	2.77E-10	4.663	no hit	-	-	-	-
-	Tag_743 CATGCCCGCTGGTGGCGCCGCTCCGG	55	3	13.957	2.998	1.05E-03	4.655	no hit	-	-	-	-
-	Tag_292 CATGCACTCAGCCGAGCGGCCGGTGG	217	12	55.068	11.992	7.76E-11	4.592	no hit	-	-	-	-
Translation/Ribosome biogenesis	Tag_758 CATGCTGGTTGGACGCACAGTGGTGA	54	3	13.704	2.998	1.25E-03	4.571	Hv_Contig_4980	1646	1477-1502	XP_013192117|eukaryotic initiation factor 4A-III *Amyelois transitella*	0.00E+00
-	Tag_763 CATGGCACGCAAACTTTGGGATGCGG	54	3	13.704	2.998	1.25E-03	4.571	no hit	-	-	-	-
-	Tag_924 CATGGCAGCCTAGCATCGCCCCGCGT	36	2	9.136	1.999	8.63E-03	4.571	no hit	-	-	-	-
Chitin-binding	Tag_92 CATGGGTCGGCAATCCTCTTGGACTT	827	46	209.867	45.970	7.54E-37	4.565	Hv_Contig_35958	389	349-374	ADB43611|chitin deacetylase 5a *Helicoverpa armigera*	1.00E-67
-	Tag_769 CATGGCACGCAAACTTTGGGATGCGC	53	3	13.450	2.998	1.50E-03	4.486	no hit	-	-	-	-
Unknown	Tag_574 CATGTTATCGCAGGACGTGCGCACGC	88	5	22.332	4.997	4.23E-05	4.469	Hv_Contig_6838	1398	1231-1256	XP_011565030|transmembrane emp24 domain-containing protein 2 *Plutella xylostella*	5.00E-121
-	Tag_580 CATGCGGAAAGTACTACAGGGTCTGG	88	5	22.332	4.997	4.23E-05	4.469	no hit	-	-	-	-
Unknown	Tag_511 CATGTTCTCCCTGGCCTAGAGGCCCG	105	6	26.646	5.996	8.01E-06	4.444	Hv_Contig_36632	373	285-260	XP_002425519|conserved hypothetical protein *Pediculus humanus corporis*	1.00E-20
-	Tag_945 CATGCCTCCTGGCTCCAGGGTGCTGG	35	2	8.882	1.999	1.03E-02	4.444	no hit	-	-	-	-
-	Tag_948 CATGGACTCCGCCGAGGGCGAGGGCG	35	2	8.882	1.999	1.03E-02	4.444	no hit	-	-	-	-
-	Tag_517 CATGTTGTGCTCTGGCTGGAACGGTA	104	6	26.392	5.996	9.52E-06	4.401	no hit	-	-	-	-
Nucleic acid binding	Tag_95 CATGCGCGTGGCGGACGGGCTCGGCT	813	47	206.314	46.970	3.13E-35	4.392	Hv_Contig_8535	1240	1158-1183	XP_011565761|replication factor C subunit 2-like *Plutella xylostella*	0.00E+00
-	Tag_661 CATGCCGCAGCGCGCGCTGGGCGTGG	69	4	17.510	3.997	3.23E-04	4.380	no hit	-	-	-	-
Primary metabolic process/hydrolase activity	Tag_106 CATGGGGCTGGACATCCCGCCGCCCA	735	43	186.520	42.972	1.00E-31	4.340	Hv_Contig_5369	1587	1236-1261	KPJ10102|Lysosomal aspartic protease *Papilio machaon*	0.00E+00
-	Tag_312 CATGCCCGCTGGTGGCGCCGCTCCAG	205	12	52.023	11.992	6.16E-10	4.338	no hit	-	-	-	-
-	Tag_103 CATGCTGCAGCGCGCGCTGGGCGTGC	750	44	190.326	43.972	2.87E-32	4.328	no hit	-	-	-	-
-	Tag_953 CATGAATTGATCCGTGCCGACCCACC	34	2	8.628	1.999	1.23E-02	4.317	no hit	-	-	-	-
-	Tag_955 CATGGACAAGTACCACCCTGGCTACG	34	2	8.628	1.999	1.23E-02	4.317	no hit	-	-	-	-
-	Tag_962 CATGATCCGTGCCGGTTGGTCCAGCG	34	2	8.628	1.999	1.23E-02	4.317	no hit	-	-	-	-
-	Tag_242 CATGGCGTATCCTGGCCCCAGCGCTG	269	16	68.264	15.990	1.89E-12	4.269	no hit	-	-	-	-
-	Tag_362 CATGTCCTGCGCACGCGTGGCGCCCA	168	10	42.633	9.994	2.66E-08	4.266	no hit	-	-	-	-
-	Tag_677 CATGTCGCTGGGGGAGAAGCTGGAGA	67	4	17.002	3.997	4.57E-04	4.253	no hit	-	-	-	-
-	Tag_317 CATGACGCGGCGCGTGCGCGCATCTG	201	12	51.007	11.992	1.22E-09	4.253	no hit	-	-	-	-
Signal transduction	Tag_49 CATGTGAGCTCGCCGGCGAGCGAGGC	2040	123	517.688	122.921	7.08E-83	4.212	Hv_Contig_50	6564	6427-6452	XP_004926833|plexin A3 *Bombyx mori*	0.00E+00
-	Tag_406 CATGTGGCGGTGACTCCGGTGGCCCT	149	9	37.812	8.994	1.91E-07	4.204	no hit	-	-	-	-
-	Tag_972 CATGCTGCAGCGCGCACTGGGCGTGG	33	2	8.374	1.999	1.47E-02	4.190	no hit	-	-	-	-
Primary metabolic process/hydrolase activity	Tag_84 CATGTTGTGCTCTGGCTGGCCCAATG	938	57	238.035	56.963	7.75E-39	4.179	Hv_Contig_13904	932	626-651	ABR88248|trypsin T5 *Heliothis virescens*	3.00E-145
Primary metabolic process/hydrolase activity	Tag_5 CATGTTGTGCTCTGGCTGGCCCAACG	29930	1821	7595.296	1819.832	0.00E+00	4.174	Hv_Contig_46294	214	44-69	CAA72957|trypsin-like protease *Helicoverpa armigera*	1.00E-16
-	Tag_481 CATGGTCCCAAGGCCCGCCGGTACCT	113	7	28.676	6.996	7.17E-06	4.099	no hit	-	-	-	-
-	Tag_483 CATGTTGTGCTCTGGCCGGCCCAACG	113	7	28.676	6.996	7.17E-06	4.099	no hit	-	-	-	-
-	Tag_991 CATGCTGCAGCGCGCGTTGGGCGTGG	32	2	8.121	1.999	1.75E-02	4.063	no hit	-	-	-	-
Primary metabolic process/hydrolase activity	Tag_77 CATGGGTGGTGCACCTCCGCGTTGCT	1116	70	283.206	69.955	1.08E-44	4.048	Hv_Contig_10648	1075	114-139	ABR88240|serine protease SP1 *Heliothis virescens*	5.00E-136
-	Tag_621 CATGGAGGACGCCGCAGCGCCCACAC	79	5	20.048	4.997	1.99E-04	4.012	no hit	-	-	-	-
-	Tag_622 CATGCTGCAGCGCGCGCTGGGCGCGG	79	5	20.048	4.997	1.99E-04	4.012	no hit	-	-	-	-
-	Tag_813 CATGTTGTGCTCTGGCTGGCTCAACG	47	3	11.927	2.998	4.25E-03	3.978	no hit	-	-	-	-
Protein folding/Recycling	Tag_7 CATGCTGCAGCGCGCGCTGGGCGTGG	18875	1219	4789.883	1218.218	0.00E+00	3.932	Hv_Contig_1372	2724	2406-2431	ADK55517.2|heat shock protein 90 cognate *Spodoptera litura*	0.00E+00
-	Tag_497 CATGCCCGGCGGTATGCCCGGCGGCG	108	7	27.407	6.996	1.66E-05	3.918	no hit	-	-	-	-
Primary metabolic process/hydrolase activity	Tag_294 CATGTTGTGCTCCGGTGTGCTCGATG	215	14	54.560	13.991	1.37E-09	3.900	Hv_Contig_46774	209	53-78	AFO68320|trypsin *Heliothis virescens*	2.00E-32
-	Tag_825 CATGTTGTACTCTGGCTGGCCCAACG	46	3	11.673	2.998	5.05E-03	3.894	no hit	-	-	-	-
Nucleic acid binding	Tag_704 CATGTGCTCGGCGTGCCCGCGCCAGT	61	4	15.480	3.997	1.27E-03	3.872	Hv_Contig_46659	210	124-149	XP_012548675|PR domain zinc finger protein 10-like isoform X4 *Bombyx mori*	4.00E-24
Primary metabolic process/hydrolase activity	Tag_15 CATGTTGTGCTCTGGCTGGCCCAGCG	9867	649	2503.935	648.584	0.00E+00	3.861	Hv_Contig_20684	704	6_31	ABW37093|putative trypsin-like proteinase *Heliothis virescens*	3.00E-31
-	Tag_400 CATGCGGTGGTGACTCCGGTGGCCCT	151	10	38.319	9.994	4.61E-07	3.834	no hit	-	-	-	-
-	Tag_514 CATGCACTCGGTCGAGCGGCCGGTGA	105	7	26.646	6.996	2.74E-05	3.809	no hit	-	-	-	-
-	Tag_1016 CATGGTAGCCTAGCTTCGCCCCGCGG	30	2	7.613	1.999	2.48E-02	3.809	no hit	-	-	-	-
-	Tag_1019 CATGAAGGATGTGCCTGGATGGGAGG	30	2	7.613	1.999	2.48E-02	3.809	no hit	-	-	-	-
-	Tag_1021 CATGGGGGACGCCGCAGCGCCCACGC	30	2	7.613	1.999	2.48E-02	3.809	no hit	-	-	-	-
-	Tag_1024 CATGGACCCCAAGTTCCTGAGGAACA	30	2	7.613	1.999	2.48E-02	3.809	no hit	-	-	-	-
-	Tag_193 CATGCGGCGGTGGCTCCGGTGGCCCT	344	23	87.296	22.985	3.72E-14	3.798	no hit	-	-	-	-
-	Tag_370 CATGCACTCGGCCGAGCGGCCGGTCG	164	11	41.618	10.993	1.75E-07	3.786	no hit	-	-	-	-
-	Tag_723 CATGTTGCGTGAACGCGGCTGTGTGC	59	4	14.972	3.997	1.78E-03	3.745	no hit	-	-	-	-
-	Tag_725 CATGCACTCGGCCGAGCGGCCGGTAA	59	4	14.972	3.997	1.78E-03	3.745	no hit	-	-	-	-
-	Tag_469 CATGGGGCTAACCGACCGTGTGTGAA	118	8	29.945	7.995	1.02E-05	3.745	no hit	-	-	-	-
Primary metabolic process/hydrolase activity	Tag_146 CATGTTCGTCAGGGTCAGCGCGTACC	494	34	125.362	33.978	3.67E-19	3.689	Hv_Contig_12402	1001	837-862	ABR88231|chymotrypsin-like protease C1 *Heliothis virescens*	0.00E+00
-	Tag_247 CATGCGGCGGTGACTCCGGCGGCCCT	261	18	66.234	17.988	7.97E-11	3.682	no hit	-	-	-	-
-	Tag_1037 CATGTTGTGCTCTGGCTGGCCCAATC	29	2	7.359	1.999	2.95E-02	3.682	no hit	-	-	-	-
-	Tag_1041 CATGATCTGTGCCGGTTGGTCCGGCG	29	2	7.359	1.999	2.95E-02	3.682	no hit	-	-	-	-
Cytoskeleton	Tag_1047 CATGACTGCGTGCATAGCAGGTCTTC	29	2	7.359	1.999	2.95E-02	3.682	Hv_Contig_3757	1868	149-124	KPJ16326|Myosin-Vb *Papilio machaon*	0.00E+00
-	Tag_1057 CATGAATACAATGCTGAAGATGACGC	29	2	7.359	1.999	2.95E-02	3.682	no hit	-	-	-	-
-	Tag_1059 CATGCCTCCTGGCTCCAGGGTGCTGC	29	2	7.359	1.999	2.95E-02	3.682	no hit	-	-	-	-
-	Tag_437 CATGCGGCAGTGACTCCGGTGGCCCT	130	9	32.990	8.994	4.52E-06	3.668	no hit	-	-	-	-
Protein folding/Recycling	Tag_238 CATGCACGAGCGAGGGCTCGGCCTGG	272	19	69.025	18.988	4.23E-11	3.635	Hv_Contig_1727	2507	381-356	XP_012553124|protein sel-1 homolog 1 isoform X2 *Bombyx mori*	0.00E+00
Cytoskeleton	Tag_52 CATGGACTCCGCCGAGGGCGAGGGTG	1996	140	506.522	139.910	6.46E-71	3.620	Hv_Contig_29832	530	407-432	XP_008294349|tubulin alpha chain-like *Stegastes partitus*	1.00E-34
-	Tag_262 CATGGGTCGGCAATCCTCTCGGACTA	239	17	60.651	16.989	8.80E-10	3.570	no hit	-	-	-	-
-	Tag_736 CATGCACTCGGCCTAGCGGCCGGTGG	56	4	14.211	3.997	2.93E-03	3.555	no hit	-	-	-	-

-	Tag_1063 CATGCTGCAGCGCGCGCTGGGTGTGG	28	2	7.106	1.999	3.49E-02	3.555	no hit	-	-	-	-
Cytoskeleton	Tag_1064 CATGTGCAGCCTTGTTGGCACACGAT	28	2	7.106	1.999	3.49E-02	3.555	Hv_Contig_153	5015	3803-3778	XP_012547232|pericentriolar material 1 protein isoform X5 *Bombyx mori*	0.00E+00
-	Tag_1067 CATGCCTGCTGGTGGTGCCGCTCCAG	28	2	7.106	1.999	3.49E-02	3.555	no hit	-	-	-	-
-	Tag_1069 CATGCGGTGGTGACTCCGGTGGCCCG	28	2	7.106	1.999	3.49E-02	3.555	no hit	-	-	-	-
-	Tag_1077 CATGGCGGCGTCACACCACGCTGTGA	28	2	7.106	1.999	3.49E-02	3.555	no hit	-	-	-	-
-	Tag_662 CATGCAGGCTGCAGCTCCATTCTCCT	69	5	17.510	4.997	1.05E-03	3.504	no hit	-	-	-	-
-	Tag_666 CATGCGCTGGCACCCGCCTCGCCGCA	69	5	17.510	4.997	1.05E-03	3.504	no hit	-	-	-	-
-	Tag_860 CATGAAGGCGGTTCCCCCAGGGCGAG	41	3	10.405	2.998	1.17E-02	3.470	no hit	-	-	-	-
mRNA processing/splicing	Tag_10 CATGGAGGACGCCGCAGCGCCCACGC	12548	922	3184.289	921.409	0.00E+00	3.456	Hv_Contig_22	7324	7183-7208	XP_013194875|pre-mRNA-processing-splicing factor 8 *Amyelois transitella*	0.00E+00
-	Tag_313 CATGCCTCCTGGCTCCAGGGTGCTGT	203	15	51.515	14.990	2.94E-08	3.437	no hit	-	-	-	-
Unknown	Tag_754 CATGTGGGAGAAGGAATACGGCCCCG	54	4	13.704	3.997	4.06E-03	3.428	Hv_Contig_30533	516	416-441	EHJ72631|hypothetical protein KGM_18901 *Danaus plexippus*	2.00E-24
Primary metabolic process/hydrolase activity	Tag_759 CATGCACGGCAGCTGGATCCCTAGCT	54	4	13.704	3.997	4.06E-03	3.428	Hv_Contig_31422	496	121-146	AFM28262|chymotrypsin, partial *Heliothis virescens*	1.00E-102
-	Tag_1084 CATGCACTCGGCCGAGCGGCCGGAGG	27	2	6.852	1.999	4.13E-02	3.428	no hit	-	-	-	-
-	Tag_1087 CATGTATCGTGTGGCAGTCGGCCGCG	27	2	6.852	1.999	4.13E-02	3.428	no hit	-	-	-	-
-	Tag_1094 CATGTCGTGCTCTGGCTGGCCCAGCG	27	2	6.852	1.999	4.13E-02	3.428	no hit	-	-	-	-
-	Tag_1099 CATGTTGTGCTCAGGCTGGCCCAACG	27	2	6.852	1.999	4.13E-02	3.428	no hit	-	-	-	-
-	Tag_1101 CATGCGGCGGTGACTCCGGTGGTCCG	27	2	6.852	1.999	4.13E-02	3.428	no hit	-	-	-	-
-	Tag_556 CATGCCCGTATTCTCATCAGGCAAAG	94	7	23.854	6.996	1.63E-04	3.410	no hit	-	-	-	-
Translation/Ribosome biogenesis	Tag_670 CATGACGCCGGAGCTGGGCGAGGACC	67	5	17.002	4.997	1.45E-03	3.403	Hv_Contig_1119	2911	1269-1294	XP_013164109|fragile X mental retardation syndrome-related protein 1 isoform X1 *Papilio xuthus*	0.00E+00
-	Tag_676 CATGGTCGCTTCCAGACGCCGGCTGG	67	5	17.002	4.997	1.45E-03	3.403	no hit	-	-	-	-
Translation/Ribosome biogenesis	Tag_244 CATGGACCCCAAGTTCCTGAGGAACC	267	20	67.756	19.987	2.80E-10	3.390	Hv_Contig_13599	945	763-788	NP_001037518|ribosomal protein L29 *Bombyx mori*	5.00E-18
-	Tag_350 CATGGGTCGGCAATCCTCTTGGACTG	173	13	43.902	12.992	3.83E-07	3.379	no hit	-	-	-	-
-	Tag_559 CATGCGGCGGTGACTCCGGTGGCTCT	93	7	23.600	6.996	1.92E-04	3.374	no hit	-	-	-	-
Protein folding/Recycling	Tag_30 CATGGCGACCCCGCTGGTGCGCGAGG	4404	333	1117.597	332.786	1.18E-142	3.358	Hv_Contig_33231	456	222-247	XP_004933282|proteasome subunit beta type-4 *Bombyx mori*	1.00E-41
-	Tag_135 CATGGGTCGGCAATCCTCTCGGACTG	528	40	133.990	39.974	1.27E-18	3.352	no hit	-	-	-	-
-	Tag_624 CATGTTGTGCTCTGGCTGGCCCAACT	79	6	20.048	5.996	6.14E-04	3.343	no hit	-	-	-	-
-	Tag_1119 CATGCGCGCGGCACGCAGAAGGGTGA	26	2	6.598	1.999	4.88E-02	3.301	no hit	-	-	-	-
-	Tag_1124 CATGGAGTGTTGCGCGTTAACGGTCC	26	2	6.598	1.999	4.88E-02	3.301	no hit	-	-	-	-
-	Tag_1127 CATGCTGCAGCGCGTGCTGGGCGTGG	26	2	6.598	1.999	4.88E-02	3.301	no hit	-	-	-	-
Primary metabolic process/hydrolase activity	Tag_17 CATGATCTGTGCCGGTTGGTCCAGCG	9184	714	2330.611	713.542	2.75E-286	3.266	Hv_Contig_14824	894	647-672	CAA72955|trypsin-like protease *Helicoverpa armigera*	1.00E-130
-	Tag_778 CATGCCGGCTGCGCGCGCAGCCTCAG	51	4	12.942	3.997	6.58E-03	3.238	no hit	-	-	-	-
-	Tag_689 CATGCTGCGGCGCGCGCTGGGCGTGG	63	5	15.987	4.997	2.73E-03	3.200	no hit	-	-	-	-
-	Tag_202 CATGGGCTGGCGCGGTGGCTTCAACC	327	26	82.982	25.983	1.42E-11	3.194	no hit	-	-	-	-
-	Tag_787 CATGTCCACGCGCTCGCACCGCTACA	50	4	12.688	3.997	7.71E-03	3.174	no hit	-	-	-	-
-	Tag_425 CATGATCTGTGCCGGTTGGTCCAGCC	137	11	34.766	10.993	1.27E-05	3.163	no hit	-	-	-	-
Unknown	Tag_899 CATGCCTGCTCGTGCTTCAGACCCAC	37	3	9.389	2.998	2.23E-02	3.132	Hv_Contig_1330	2751	2429-2454	KPI98384|hypothetical protein RR46_09600 *Papilio xuthus*	2.00E-169
-	Tag_905 CATGACCTGTGCCGGCTGGCCCAGCG	37	3	9.389	2.998	2.23E-02	3.132	no hit	-	-	-	-
-	Tag_908 CATGTGAGCTCGCCGGCGAGCGAGGT	37	3	9.389	2.998	2.23E-02	3.132	no hit	-	-	-	-
-	Tag_644 CATGGGGAGGTTCTGCTCAGGACTGT	74	6	18.779	5.996	1.34E-03	3.132	no hit	-	-	-	-
-	Tag_41 CATGCACGCGCGCGCGGGCGACGCGC	2702	220	685.683	219.859	2.52E-81	3.119	no hit	-	-	-	-
-	Tag_271 CATGCGGCGGCGACTCCGGTGGCCCT	233	19	59.128	18.988	1.82E-08	3.114	no hit	-	-	-	-
-	Tag_19 CATGCGGCGGTGACTCCGGTGGCCCC	8505	700	2158.302	699.551	2.41E-248	3.085	no hit	-	-	-	-
mRNA processing/splicing	Tag_588 CATGTCGGATGCCTACCTCGGGGCTG	85	7	21.570	6.996	6.66E-04	3.083	Hv_Contig_20288	715	464-489	KPJ02294|Putative U5 small nuclear ribonucleoprotein 200 kDa helicase *Papilio xuthus*	6.00E-108
-	Tag_257 CATGGGTGGTGCACCTCCGCGTTGCG	242	20	61.412	19.987	1.27E-08	3.073	no hit	-	-	-	-
Translation/Ribosome biogenesis	Tag_150 CATGGAGTGTTGCGCGTTAACGGTCG	481	40	122.063	39.974	1.54E-15	3.054	Hv_Contig_22213	663	209-234	Q95V31|40S ribosomal protein S16 *Spodoptera frugiperda*	3.00E-95
-	Tag_714 CATGCTCCGCTAATCTACCAACACTC	60	5	15.226	4.997	4.36E-03	3.047	no hit	-	-	-	-
Translation/Ribosome biogenesis	Tag_803 CATGAAGTGGTTCCAACAGAAATACG	48	4	12.181	3.997	1.05E-02	3.047	Hv_Contig_5248	1605	732-757	NP_001298894|60S ribosomal protein L11 *Papilio xuthus*	4.00E-119
Translation/Ribosome biogenesis	Tag_124 CATGATCGCACACGCCGGCGCCGTGG	571	48	144.902	47.969	6.28E-18	3.021	Hv_Contig_10163	1127	940-965	XP_013192781|probable tRNA N6-adenosine threonylcarbamoyltransferase *Amyelois transitella*	0.00E+00
-	Tag_656 CATGGAGTGTTGCGCGTTAACGGTCA	71	6	18.018	5.996	2.13E-03	3.005	no hit	-	-	-	-
-	Tag_507 CATGCGGCGGTGACTCCAGTGGCCCT	106	9	26.899	8.994	1.94E-04	2.991	no hit	-	-	-	-
-	Tag_816 CATGGGTCGGCAATCCTCTCGGACGT	47	4	11.927	3.997	1.23E-02	2.984	no hit	-	-	-	-
Nucleic acid binding	Tag_602 CATGGTTCCAAAACCGTCGCGCGAAG	82	7	20.809	6.996	1.05E-03	2.975	Hv_Contig_13411	953	712-737	XP_013186282|homeobox protein Hox-A7-like *Amyelois transitella*	1.00E-88
-	Tag_44 CATGGGTCGGCAATCCTCTCGGACTT	2325	200	590.012	199.872	9.06E-66	2.952	no hit	-	-	-	-
-	Tag_824 CATGTTGTGCTCTGACTGGCCCAACG	46	4	11.673	3.997	1.44E-02	2.920	no hit	-	-	-	-
-	Tag_827 CATGTCCTGCGCACGCGTGGCGCCCG	46	4	11.673	3.997	1.44E-02	2.920	no hit	-	-	-	-
-	Tag_447 CATGCTCGCAGTCGTAGCTGCTAACA	126	11	31.975	10.993	6.60E-05	2.909	no hit	-	-	-	-
Primary metabolic process/oxidoreductase activity	Tag_199 CATGGCACGCAAACTTTGGGATGCGT	332	29	84.251	28.981	1.24E-10	2.907	Hv_Contig_8262	1263	1171-1196	XP_013200704|retinol dehydrogenase 11-like *Amyelois transitella*	6.00E-121
-	Tag_616 CATGCGGAAAGTACTACAGGGTCTGT	80	7	20.301	6.996	1.42E-03	2.902	no hit	-	-	-	-
Primary metabolic process/hydrolase activity	Tag_4 CATGCGGCGGTGACTCCGGTGGCCCT	79387	6961	20145.932	6956.535	0.00E+00	2.896	Hv_Contig_14443	909	655-680	AFM28249|chymotrypsin *Heliothis virescens*	7.00E-150
-	Tag_966 CATGCCTGCTGGTGGTGCCGCTCCCC	34	3	8.628	2.998	3.58E-02	2.878	no hit	-	-	-	-
-	Tag_227 CATGTTGTGCTCTGGCTGGCCCAACC	292	26	74.100	25.983	2.47E-09	2.852	no hit	-	-	-	-
-	Tag_491 CATGCGGCGGTGACTCCGGTGACCCT	110	10	27.915	9.994	2.72E-04	2.793	no hit	-	-	-	-
-	Tag_680 CATGCGGCGGTGTCTCCGGTGGCCCT	66	6	16.749	5.996	4.50E-03	2.793	no hit	-	-	-	-
-	Tag_631 CATGCGACGGTGACTCCGGTGGCCCT	77	7	19.540	6.996	2.21E-03	2.793	no hit	-	-	-	-
mRNA processing/splicing	Tag_577 CATGCGCCTGCCACCTCCTCCCGGGG	88	8	22.332	7.995	1.09E-03	2.793	Hv_Contig_13052	968	832-857	XP_013185622|splicing factor 3A subunit 2 isoform X1 *Amyelois transitella*	3.00E-139
-	Tag_225 CATGCGCCGCGGTGGGCCCGCGCCTT	293	27	74.354	26.983	5.25E-09	2.756	no hit	-	-	-	-
-	Tag_751 CATGGACTCCGCCGAGGGCGAGGGTC	54	5	13.704	4.997	1.08E-02	2.742	no hit	-	-	-	-

Primary metabolic process/hydrolase activity	Tag_586 CATGGCTGCACTGCAAAATTGGTGAT	86	8	21.824	7.995	1.46E-03	2.730	Hv_Contig_5238	1606	36-11	XP_004923722|putative serine protease K12H4.7 *Bombyx mori*	0.00E+00
Translation/Ribosome biogenesis	Tag_223 CATGGTCTTCAAGGACGACGATAGAC	295	28	74.862	27.982	9.48E-09	2.675	Hv_Contig_13427	952	506-531	XP_013139400|ubiquitin-40S ribosomal protein S27a isoform X2 *Papilio polytes*	1.00E-84
-	Tag_696 CATGCGGCGGTGGCTCCGGTGGCCCG	63	6	15.987	5.996	6.98E-03	2.666	no hit	-	-	-	-
Cytoskeleton	Tag_101 CATGCGCAGTGCCCGCGTCGCGTGCG	755	72	191.595	71.954	7.95E-20	2.663	Hv_Contig_42	6714	6601-6626	XP_013169164|myosin heavy chain, non-muscle *Papilio xuthus*	0.00E+00
unknown	Tag_160 CATGTTGCGTGAACGCGGCTGTGTGT	437	42	110.897	41.973	4.90E-12	2.642	Hv_Contig_3059	2024	1967-1992	XP_014361499|protein yellow-like *Papilio machaon*	0.00E+00
-	Tag_700 CATGCGGCGGTGATTCCGGTGGCCCT	62	6	15.734	5.996	8.05E-03	2.624	no hit	-	-	-	-
-	Tag_521 CATGCGGCGGTGACTCTGGTGGCCCT	103	10	26.138	9.994	7.36E-04	2.615	no hit	-	-	-	-
-	Tag_859 CATGCCGTCGACCACAGGTGAAGCAG	41	4	10.405	3.997	3.04E-02	2.603	no hit	-	-	-	-
Translation/Ribosome biogenesis	Tag_861 CATGCGGCCAGTTCCTGTGGTCTTCC	41	4	10.405	3.997	3.04E-02	2.603	Hv_Contig_35982	389	264-239	NP_001298496|60S ribosomal protein L37 *Papilio polytes*	6.00E-50
-	Tag_867 CATGTCCTGCGCACGCGTGGCGCCTC	41	4	10.405	3.997	3.04E-02	2.603	no hit	-	-	-	-
Unknown	Tag_100 CATGCGGCGCGGTGTGACGTCACAGG	757	74	192.103	73.953	3.23E-19	2.598	Hv_Contig_2569	2167	1840-1865	XP_004929441|WEB family protein At4g27595, chloroplastic-like isoform X1 *Bombyx mori*	1.00E-180
-	Tag_780 CATGTTGTGCTCGGGCTGGCCCAACG	51	5	12.942	4.997	1.67E-02	2.590	no hit	-	-	-	-
Nucleic acid binding	Tag_183 CATGTCCACGCGCTCGCACCGCTACG	382	38	96.940	37.976	2.97E-10	2.553	Hv_Contig_11722	1036	776-801	XP_004922819|zinc finger protein 330 homolog *Bombyx mori*	1.00E-143
Signal transduction	Tag_869 CATGCGGGCGCTGCGCGTGCTGCCGC	40	4	10.151	3.997	3.51E-02	2.539	Hv_Contig_3666	1889	507-482	XP_013200279|protein LMBR1L *Amyelois transitella*	0.00E+00
-	Tag_871 CATGTTGTGTTCTGGCTGGCCCAACG	40	4	10.151	3.997	3.51E-02	2.539	no hit	-	-	-	-
Unknown	Tag_660 CATGCCCAGCTCCGCCGCCAGGCATC	70	7	17.764	6.996	6.02E-03	2.539	Hv_Contig_9034	1206	624-649	EHJ72715|hypothetical protein KGM_16094 *Danaus plexippus*	6.00E-132
-	Tag_263 CATGGGTCGGCAATCCTCTCGGACTC	239	24	60.651	23.985	6.84E-07	2.529	no hit	-	-	-	-
-	Tag_9 CATGCGGCGGTGACTCCGGTGGCCCG	12982	1305	3294.425	1304.163	5.17E-287	2.526	no hit	-	-	-	-
