# Supplementary material for: HT‐SuperSAGE of the gut tissue of a Vip3Aa‐resistant Heliothis virescens (Lepidoptera: Noctuidae) strain provides insights into the basis of resistance
Source: Insect Sci. 2017 Dec 1;26(3):479–98. doi: 10.1111/1744-7917.12535 (PMC6849831; doi:10.1111/1744-7917.12535)
Supplement: Supplementary file 3 — Table S3. List of underexpressed (UE) UniTags sequences, copy number, and annotations to the Heliothis virescens sequence contigs database. [file INS-26-479-s003.pdf]

Vip-Sel: number of Tags in the Vip-Sel library

Vip-Unsel: number of Tags in the Vip-Unsel library

Norm. Vip-Sel: number of tags in Vip-Sel normalized to 268000 (See Materials & Methods for details)

Norm. Vip-Unsel: number of tags in Vip-Unsel normalized to 268000 (See Materials & Methods for details)

***p*-value: Statistical value associated with the different in tag copy number between libraries calculated according to Audic and Claverie (1997)**

LOG(FC): fold change (Norm. Vip-Sel vs. Norm. Vip-Unsel) expressed as the log2 value.

Heliothis virescens CONTIG match: Identifier of the Contig sequence of Heliothis virescens (from Perera et al., 2015) that perfectly matched (26bp) with the corresponding tag in BlastN search

List of under-expressed Unitags sequences, copy number and annotations to the Heliothis virescens sequence contigs database

General classification (Process/Molecular Function)	TAG	Vip-Sel	Vip-Unsel	Norm. Vip-Sel	Norm. Vip-Unsel	*p*-value	LOG(FC)	Heliothis virescens CONTIG match	Contig size (nt)	Tag position in the contig (nt)	Protein annotation: blastx	E-value
-	Tag_4628 CATGCTGGTGGCGGCTATGGCCGAGC	1	379	0.254	378.757	8.53E-262	-10.54	no hit	-	-	-	-
-	Tag_4876 CATGCTGGTGGCAGCGATGGCTGAGC	1	375	0.254	374.759	5.02E-259	-10.53	no hit	-	-	-	-
-	Tag_4680 CATGTATGCGAGTCATTGAGATAAAG	1	282	0.254	281.819	1.20E-194	-10.12	no hit	-	-	-	-
-	Tag_3843 CATGTATGCGAGTCATTGAGATAATT	2	561	0.508	560.640	0.00E+00	-10.11	no hit	-	-	-	-
-	Tag_2632 CATGTATGCGAGTCATTGAGATAAAA	5	1282	1.269	1281.178	0.00E+00	-9.98	no hit	-	-	-	-
Unknown	Tag_755 CATGTATGCGAGTCATTGAGATAATA	54	12136	13.704	12128.216	0.00E+00	-9.79	Hv_Contig_14845	893	28-53	XP_001624571|predicted protein *Nematostella vectensis*	2.00E-23
-	Tag_2982 CATGTATGCGAGTCATTGAGATAAGA	4	676	1.015	675.566	0.00E+00	-9.38	no hit	-	-	-	-
-	Tag_3801 CATGTATGCGAGTCATTGAGATAACA	2	326	0.508	325.791	5.53E-223	-9.33	no hit	-	-	-	-
-	Tag_1378 CATGTATGCGAGTCATTGAGATAATG	19	3061	4.822	3059.037	0.00E+00	-9.31	no hit	-	-	-	-
-	Tag_3683 CATGTTATACTTTTAGTGAGTCACCA	2	274	0.508	273.824	4.55E-187	-9.08	no hit	-	-	-	-
-	Tag_551 CATGTTATACTTTTAGTGAGTCACCG	95	10448	24.108	10441.299	0.00E+00	-8.76	no hit	-	-	-	-
-	Tag_4332 CATGTCGTGAGTGCTGTTATATGACG	1	69	0.254	68.956	1.61E-47	-8.09	no hit	-	-	-	-
-	Tag_2488 CATGCACGGGAAAGTGGCCCGGCTCA	6	306	1.523	305.804	3.85E-202	-7.65	no hit	-	-	-	-
-	Tag_2527 CATGTCGTGAGTGCTGTTATATGACT	5	209	1.269	208.866	2.67E-137	-7.36	no hit	-	-	-	-
-	Tag_4724 CATGCCGTGAACCACGCCGTGGCAGA	1	41	0.254	40.974	2.56E-28	-7.34	no hit	-	-	-	-
-	Tag_4807 CATGATCTGCGCCGGTATCCTGGATG	1	38	0.254	37.976	2.87E-26	-7.23	no hit	-	-	-	-
-	Tag_4982 CATGTACGCGAGTCATTGAGATAATA	1	38	0.254	37.976	2.87E-26	-7.23	no hit	-	-	-	-
-	Tag_3106 CATGTTATACTTTTAGTGAGTCACTG	3	99	0.761	98.937	3.98E-65	-7.02	no hit	-	-	-	-
-	Tag_4190 CATGCAATCAGAACTCTGAACCTGTA	1	32	0.254	31.979	3.54E-22	-6.98	no hit	-	-	-	-
-	Tag_5202 CATGGAACTCGAACGCTCAGGCAGAG	1	32	0.254	31.979	3.54E-22	-6.98	no hit	-	-	-	-
-	Tag_3482 CATGCGGCGCTCAACCGTCACCATCA	2	61	0.508	60.961	1.29E-40	-6.91	no hit	-	-	-	-
-	Tag_3323 CATGGCTCGTGGTCACCTTGCCGCGG	3	89	0.761	88.943	2.52E-58	-6.87	no hit	-	-	-	-
Transport/Trafficking	Tag_4060 CATGCTGATGCTGCTGCCGTTCCTGC	1	28	0.254	27.982	1.86E-19	-6.78	Hv_Contig_4634	1703	601-626	XP_012549161|solute carrier family 12 member 6 isoform X2 *Bombyx mori*	0.00E+00
-	Tag_3057 CATGTATGCGAGTCATTGAAAAAAAA	3	83	0.761	82.947	2.99E-54	-6.77	no hit	-	-	-	-
-	Tag_4351 CATGCACGGGAAGGTGGCCCGGCTCT	1	27	0.254	26.983	8.88E-19	-6.73	no hit	-	-	-	-
-	Tag_4454 CATGGGGCCGTGGCTGCGCTGATGTG	1	25	0.254	24.984	2.02E-17	-6.62	no hit	-	-	-	-
-	Tag_1018 CATGCACGGGAAAGTGGCCCGGCTCG	30	707	7.613	706.547	0.00E+00	-6.54	no hit	-	-	-	-
-	Tag_4115 CATGTCGTGAGTGCTGTTATATGACA	1	23	0.254	22.985	4.56E-16	-6.50	no hit	-	-	-	-
-	Tag_4974 CATGCACGGGAAAGTGGCCTGGCTCT	1	23	0.254	22.985	4.56E-16	-6.50	no hit	-	-	-	-
-	Tag_2822 CATGTGCGCGTGGAAGCCGCGCTGTG	4	90	1.015	89.942	1.00E-57	-6.47	no hit	-	-	-	-
-	Tag_4900 CATGTTATGCTTTTAGTGAGTCACCG	1	22	0.254	21.986	2.16E-15	-6.44	no hit	-	-	-	-
-	Tag_3601 CATGCTTGCTGCAAAAAACTGCTGGG	2	41	0.508	40.974	4.52E-27	-6.34	no hit	-	-	-	-
-	Tag_4488 CATGAGGCTCTCATTAGGCGGCTGGG	1	19	0.254	18.988	2.28E-13	-6.23	no hit	-	-	-	-
-	Tag_4732 CATGCGCGGGAAAGTGGCCCGGCTCT	1	19	0.254	18.988	2.28E-13	-6.23	no hit	-	-	-	-
-	Tag_1336 CATGCACGGGAAAGTGGCCCGGCTCC	20	365	5.075	364.766	2.12E-223	-6.17	no hit	-	-	-	-
Primary metabolic process/hydrolase activity	Tag_4722 CATGATTGAAATATGCATCTATTTGG	1	18	0.254	17.988	1.07E-12	-6.15	Hv_Contig_9978	1139	976-1001	ABR88239|chymotrypsin-like protease C9 *Heliothis virescens*	8.00E-127
-	Tag_3700 CATGGTGGGCCGCATCGGCTCCATCG	2	34	0.508	33.978	2.28E-22	-6.06	no hit	-	-	-	-
-	Tag_4616 CATGCTAGAGTACGCGCCCGCAGTAG	1	17	0.254	16.989	5.04E-12	-6.06	no hit	-	-	-	-
-	Tag_233 CATGCACGGGAAAGTGGCCCGGCTCT	286	4844	72.578	4840.893	0.00E+00	-6.06	no hit	-	-	-	-
Primary metabolic process/oxidoreductase activity	Tag_1852 CATGGAACTCGAACGCTCAGGCAGAA	11	183	2.791	182.883	5.96E-112	-6.03	Hv_Contig_21233	689	218-193	KPJ14522|Chorion peroxidase *Papilio machaon*	7.00E-70
-	Tag_4839 CATGCATCTGGTCGGCTTCAGCTTGG	1	16	0.254	15.990	2.36E-11	-5.98	no hit	-	-	-	-
-	Tag_1670 CATGCCCGGCCACCAGCTGTTCGCGA	13	205	3.299	204.869	2.14E-124	-5.96	no hit	-	-	-	-
-	Tag_1850 CATGTGCGCGCTGGTTCCTTGTGGGG	11	172	2.791	171.890	1.32E-104	-5.94	no hit	-	-	-	-
Protein kinases	Tag_4310 CATGTGGCGCGCAGGCGTCGCGTGCG	1	14	0.254	13.991	5.12E-10	-5.78	Hv_Contig_399	3990	43-68	EHJ69302|venus kinase receptor *Danaus plexippus*	4.00E-96
-	Tag_4316 CATGAGCTGCGACGATGCGCCGCCGT	1	14	0.254	13.991	5.12E-10	-5.78	no hit	-	-	-	-
Unknown	Tag_4719 CATGGGAACTACTCGGCGCACCCGGA	1	14	0.254	13.991	5.12E-10	-5.78	Hv_Contig_23364	636	494-469	XP_013199167|uncharacterized protein LOC106142086 *Amyelois transitella*	4.00E-11
-	Tag_4957 CATGGCTCGTGGTCACCTTGCCGCGT	1	14	0.254	13.991	5.12E-10	-5.78	no hit	-	-	-	-
-	Tag_5109 CATGGACACAGCGCTCAACGAGCAGT	1	14	0.254	13.991	5.12E-10	-5.78	no hit	-	-	-	-
Primary metabolic process/transferase activity	Tag_3223 CATGAACCAGTGTATGTAAGGTGTAC	3	41	0.761	40.974	5.44E-26	-5.75	Hv_Contig_66	6298	1833-1858	XP_011555786|uncharacterized protein LOC105386838 *Plutella xylostella*	0.00E+00
-	Tag_3262 CATGTGCGCGCTGGTTCCTTGTGGGA	3	41	0.761	40.974	5.44E-26	-5.75	no hit	-	-	-	-

-	Tag_4202 CATGTCCAGCACGTCGAAACCAACGG	1	13	0.254	12.992	2.37E-09	-5.68	no hit	-	-	-	-
-	Tag_4660 CATGTCGCTTACGCCAGCGACAACGG	1	13	0.254	12.992	2.37E-09	-5.68	no hit	-	-	-	-
Primary metabolic process/hydrolase activity	Tag_1705 CATGCCTGGGTGTGCCACCGCGCTCT	13	161	3.299	160.897	3.48E-95	-5.61	Hv_Contig_9835	1150	787-812	AFI64311|neutral lipase *Helicoverpa armigera*	0.00E+00
-	Tag_4380 CATGCGCACGCGCAAGTCTGCCGTCG	1	12	0.254	11.992	1.10E-08	-5.56	no hit	-	-	-	-
-	Tag_4484 CATGCAGCGCCGCTGACCACCTCGCG	1	12	0.254	11.992	1.10E-08	-5.56	no hit	-	-	-	-
Nucleic acid binding	Tag_4868 CATGTCGCTTATGCCAGCGATAACGG	1	12	0.254	11.992	1.10E-08	-5.56	Hv_Contig_143	5100	4657-4632	EHJ65667|putative YLP motif containing 1 *Danaus plexippus*	7.00E-142
-	Tag_3460 CATGGGGCCGTGGCTGCGCTGATGAT	2	24	0.508	23.985	1.04E-15	-5.56	no hit	-	-	-	-
-	Tag_3526 CATGCTCACCGCGTCCGCTGAGGAGG	2	24	0.508	23.985	1.04E-15	-5.56	no hit	-	-	-	-
-	Tag_4261 CATGGGGCCGTGGCTGCGCTGATGTA	1	11	0.254	10.993	5.03E-08	-5.44	no hit	-	-	-	-
-	Tag_4263 CATGTACGGGAAAGTGGCCCGGCTCT	1	11	0.254	10.993	5.03E-08	-5.44	no hit	-	-	-	-
-	Tag_4661 CATGTGTGGCGCAGCACGAGCTTCGT	1	11	0.254	10.993	5.03E-08	-5.44	no hit	-	-	-	-
-	Tag_4832 CATGGCAGTGGCGGCGCCTAAGCCAG	1	11	0.254	10.993	5.03E-08	-5.44	no hit	-	-	-	-
Transport/Trafficking	Tag_4908 CATGAGCCCACGGAACGCGCCCCCAC	1	11	0.254	10.993	5.03E-08	-5.44	Hv_Contig_13176	963	141-166	XP_013182903|ATP synthase mitochondrial F1 complex assembly factor 2 isoform X1 *Amyelois transitella*	3.00E-147
-	Tag_5114 CATGGGGCCGTGGCTGCGCTGATGTC	1	11	0.254	10.993	5.03E-08	-5.44	no hit	-	-	-	-
-	Tag_5229 CATGGGCGCCGGACGCAAGATCCTGC	1	11	0.254	10.993	5.03E-08	-5.44	no hit	-	-	-	-
Translation/Ribosome biogenesis	Tag_2652 CATGCGCACGCGCAAGTCTGCCGTCA	5	52	1.269	51.967	2.64E-31	-5.36	Hv_Contig_13406	953	741-716	KPJ02027|WD repeat-containing protein 43 *Papilio xuthus*	6.00E-74
Primary metabolic process/hydrolase activity	Tag_4176 CATGCGTGGCTTGCTCGAATAGGCGG	1	10	0.254	9.994	2.30E-07	-5.30	Hv_Contig_11166	1068	465-440	ACN29686|UDP-N-acetylglucosamine pyrophosphorylase *Spodoptera exigua*	1.00E-20
-	Tag_4657 CATGCACGGGAAAGTGGCCCGACTCT	1	10	0.254	9.994	2.30E-07	-5.30	no hit	-	-	-	-
-	Tag_4691 CATGGCTCGTGGTCACCTTGCCGCAA	1	10	0.254	9.994	2.30E-07	-5.30	no hit	-	-	-	-
-	Tag_4714 CATGCACGGGAAAGTGGCCCGGTTCT	1	10	0.254	9.994	2.30E-07	-5.30	no hit	-	-	-	-
-	Tag_5026 CATGACAGCTGCGGCATCTGCTCGGT	1	10	0.254	9.994	2.30E-07	-5.30	no hit	-	-	-	-
-	Tag_5074 CATGTATGCGATTCATTGAGATAATA	1	10	0.254	9.994	2.30E-07	-5.30	no hit	-	-	-	-
Transport/Trafficking	Tag_5119 CATGAGCAGCCTGGCGAGCAGCGTGC	1	10	0.254	9.994	2.30E-07	-5.30	Hv_Contig_25750	596	496-521	KPJ15001|Trafficking kinesin-binding protein milt *Papilio machaon*	6.00E-67
-	Tag_3109 CATGCCGTGAACCACGCCGTGGCAGT	3	29	0.761	28.981	4.43E-18	-5.25	no hit	-	-	-	-
-	Tag_3721 CATGGGACTCACCGAGTACGACGCTA	2	19	0.508	18.988	2.02E-12	-5.23	no hit	-	-	-	-
-	Tag_3779 CATGCTGCCCCCGACTGCCGCGCGGG	2	18	0.508	17.988	9.10E-12	-5.15	no hit	-	-	-	-
-	Tag_4309 CATGCTGCGGCCCCTGCGGCGGGTGT	1	9	0.254	8.994	1.04E-06	-5.15	no hit	-	-	-	-
-	Tag_4347 CATGGGGCCGTAGCTGCGCTGATGCT	1	9	0.254	8.994	1.04E-06	-5.15	no hit	-	-	-	-
-	Tag_4377 CATGCAAATGTCGCGGCGAGTCCGCT	1	9	0.254	8.994	1.04E-06	-5.15	no hit	-	-	-	-
Translation/Ribosome biogenesis	Tag_4740 CATGATTGCAACGGGAGCGAAGGAAA	1	9	0.254	8.994	1.04E-06	-5.15	Hv_Contig_16686	822	53-28	ADT80643|ribosomal protein S3 *Euphydryas aurinia*	7.00E-158
-	Tag_4760 CATGCGCGCGCTGGTTCCTTGTGGGT	1	9	0.254	8.994	1.04E-06	-5.15	no hit	-	-	-	-
Primary metabolic process/hydratase activity	Tag_4815 CATGCGGCGCCTCCGCCACATTCTAT	1	9	0.254	8.994	1.04E-06	-5.15	Hv_Contig_1883	2433	1017-1042	XP_004932565|probable aconitate hydratase, mitochondrial *Bombyx mori*	3.00E-38
-	Tag_4902 CATGTGCGAGGGCAGTCAGTCATAGC	1	9	0.254	8.994	1.04E-06	-5.15	no hit	-	-	-	-
-	Tag_5029 CATGCTGCCCCCGACTGCCGCGCGAA	1	9	0.254	8.994	1.04E-06	-5.15	no hit	-	-	-	-
-	Tag_5135 CATGGTTGGTGCGCGCGGGGGCCTTC	1	9	0.254	8.994	1.04E-06	-5.15	no hit	-	-	-	-
Primary metabolic process/hydrolase activity	Tag_5163 CATGGCCCAACTGCCTGATGTGCCGA	1	9	0.254	8.994	1.04E-06	-5.15	Hv_Contig_18739	760	542-567	AID66662|desaturase *Agrotis segetum*	7.00E-111
-	Tag_2792 CATGTCGTGAGTGCTGTTATATGACC	4	36	1.015	35.977	9.17E-22	-5.15	no hit	-	-	-	-
-	Tag_3856 CATGCACGGGAAAGCGGCCCGGCTCT	2	17	0.508	16.989	4.07E-11	-5.06	no hit	-	-	-	-
Primary metabolic process/hydrolase activity	Tag_2538 CATGAAGTACTGCGAGTTTGCCGACC	5	40	1.269	39.974	1.67E-23	-4.98	Hv_Contig_3779	1863	1655-1680	XP_004928865|N-acetylglucosamine-6-sulfatase-like *Bombyx mori*	0.00E+00
-	Tag_2376 CATGGGGCCGTGGCTGCGCTGATGTT	6	48	1.523	47.969	7.97E-28	-4.98	no hit	-	-	-	-
Primary metabolic process/oxidoreductase activity	Tag_4130 CATGTATTAGAAGAAATAGAGATAAG	1	8	0.254	7.995	4.68E-06	-4.98	Hv_Contig_21488	682	88-63	YP_009183765|NADH dehydrogenase subunit 2 (mitochondrion) *Heliothis subflexa*	9.00E-51
-	Tag_4462 CATGTGAGCACGCTCCACGCCGCCCA	1	8	0.254	7.995	4.68E-06	-4.98	no hit	-	-	-	-
-	Tag_4499 CATGGCGGGCCCGCGCCCGCGCACTT	1	8	0.254	7.995	4.68E-06	-4.98	no hit	-	-	-	-
-	Tag_4581 CATGTTGTAATGGTGTTTATGTGATA	1	8	0.254	7.995	4.68E-06	-4.98	no hit	-	-	-	-
-	Tag_4588 CATGTTGTGGACTCCGTTGCTGAGCA	1	8	0.254	7.995	4.68E-06	-4.98	no hit	-	-	-	-
-	Tag_4632 CATGAAATAAAACAAAACAATTATGA	1	8	0.254	7.995	4.68E-06	-4.98	no hit	-	-	-	-
-	Tag_4712 CATGCTGCCCCCGACTGCCGCGCACG	1	8	0.254	7.995	4.68E-06	-4.98	no hit	-	-	-	-
-	Tag_4731 CATGTGTGGGGAGGAGTGAGCGAGGT	1	8	0.254	7.995	4.68E-06	-4.98	no hit	-	-	-	-
-	Tag_4738 CATGCGCAGCTGGCCGATCTCCTTGC	1	8	0.254	7.995	4.68E-06	-4.98	no hit	-	-	-	-
-	Tag_4799 CATGCACGGGAAAGTGGCCCAGCTCT	1	8	0.254	7.995	4.68E-06	-4.98	no hit	-	-	-	-
-	Tag_4814 CATGAACCAGTGTATGTAAGGTGTGC	1	8	0.254	7.995	4.68E-06	-4.98	no hit	-	-	-	-
-	Tag_5112 CATGCCGTACGCCAGGACGCTGGAGA	1	8	0.254	7.995	4.68E-06	-4.98	no hit	-	-	-	-
Primary metabolic process/hydrolase activity	Tag_5140 CATGTTGGCCCTGAGAGTCGTCTCTA	1	8	0.254	7.995	4.68E-06	-4.98	Hv_Contig_16252	838	675-700	ABU98613|alpha-amylase *Helicoverpa armigera*	2.00E-140
-	Tag_5170 CATGGACCTAGGCGCGAGTACGCAGT	1	8	0.254	7.995	4.68E-06	-4.98	no hit	-	-	-	-
-	Tag_2541 CATGCCCGGCCACCAGCTGTTCGCGG	5	38	1.269	37.976	3.23E-22	-4.90	no hit	-	-	-	-
-	Tag_3923 CATGTTATACTTTTAGTGAGTCACAA	2	15	0.508	14.990	8.02E-10	-4.88	no hit	-	-	-	-
-	Tag_3082 CATGCCGGGCCGCGCCGGTGCCGCCG	3	22	0.761	21.986	1.52E-13	-4.85	no hit	-	-	-	-
Transport/Trafficking	Tag_1353 CATGGTGGGCCGCATCGGCTCCATCA	19	135	4.822	134.913	7.18E-73	-4.81	Hv_Contig_13483	949	717-742	KOB66490|Organic cation transporter *Operophtera brumata*	7.00E-132

-	Tag_4041 CATGCGTCTCGGTTGCGTCGTGGGCT	1	7	0.254	6.996	2.09E-05	-4.78	no hit	-	-	-	-
-	Tag_4123 CATGCTGCCCCCGACTGCCGCGCGTC	1	7	0.254	6.996	2.09E-05	-4.78	no hit	-	-	-	-
-	Tag_4143 CATGGGTCCCGAGTGTATGGGGTGGA	1	7	0.254	6.996	2.09E-05	-4.78	no hit	-	-	-	-
-	Tag_4160 CATGGCTCGCAGCCGCCCCCTGGTGT	1	7	0.254	6.996	2.09E-05	-4.78	no hit	-	-	-	-
Primary metabolic process/GTPase activity	Tag_4312 CATGCTGGCCGCCTGGGGGTACACCA	1	7	0.254	6.996	2.09E-05	-4.78	Hv_Contig_3630	1894	1493-1518	XP_013190963|atlastin-like *Amyelois transitella*	0.00E+00
-	Tag_4513 CATGGCTGCGGCCCAAGGAGTGAACG	1	7	0.254	6.996	2.09E-05	-4.78	no hit	-	-	-	-
-	Tag_4577 CATGCCCGGCCACCAGCTGTTCACGC	1	7	0.254	6.996	2.09E-05	-4.78	no hit	-	-	-	-
-	Tag_4585 CATGCCCGACCACCAGCTGTTCGCGC	1	7	0.254	6.996	2.09E-05	-4.78	no hit	-	-	-	-
-	Tag_4784 CATGCCCGGCCACCAACTGTTCGCGC	1	7	0.254	6.996	2.09E-05	-4.78	no hit	-	-	-	-
-	Tag_4818 CATGTTGTAATGGTGTTTATGTGATC	1	7	0.254	6.996	2.09E-05	-4.78	no hit	-	-	-	-
-	Tag_4837 CATGGCGCCGCTCGCGCCGCCCGCGC	1	7	0.254	6.996	2.09E-05	-4.78	no hit	-	-	-	-
Primary metabolic process/hydrolase activity	Tag_5051 CATGTGGGCGGAGCTCCAAGAGACGG	1	7	0.254	6.996	2.09E-05	-4.78	Hv_Contig_32076	482	153-178	XP_013192188|tat-linked quality control protein TatD *Amyelois transitella*	9.00E-91
-	Tag_5121 CATGGGACTCACCGAGTACGACGTCG	1	7	0.254	6.996	2.09E-05	-4.78	no hit	-	-	-	-
-	Tag_5146 CATGCGCGCCGCTGGGACTGCGCCGC	1	7	0.254	6.996	2.09E-05	-4.78	no hit	-	-	-	-
-	Tag_5176 CATGAGCTCAGACGTCGCGGCCTCGG	1	7	0.254	6.996	2.09E-05	-4.78	no hit	-	-	-	-
-	Tag_5188 CATGTATGCGTGTCATTGAGATAATA	1	7	0.254	6.996	2.09E-05	-4.78	no hit	-	-	-	-
Primary metabolic process/oxidoreductase activity	Tag_5234 CATGGCGCGCTCGGTTCCGCGATGCG	1	7	0.254	6.996	2.09E-05	-4.78	Hv_Contig_16308	835	12_37	XP_013185861|probable NADH dehydrogenase [ubiquinone] 1 alpha subcomplex subunit 12 *Amyelois transitella*	3.00E-64
-	Tag_3146 CATGGCCATCAACAACGCCCTGGTCA	3	21	0.761	20.987	6.65E-13	-4.78	no hit	-	-	-	-
-	Tag_3876 CATGGGGCCGTGGCTGCACTGATGCT	2	14	0.508	13.991	3.53E-09	-4.78	no hit	-	-	-	-
-	Tag_4029 CATGCCTGGGTGTGCCACCGCGCTCC	2	14	0.508	13.991	3.53E-09	-4.78	no hit	-	-	-	-
-	Tag_884 CATGGGGCCGTGGCTGCGCTGATGCA	39	269	9.897	268.827	4.31E-142	-4.76	no hit	-	-	-	-
-	Tag_1407 CATGCCCGGCCACCAGCTGTTCGCGT	18	123	4.568	122.921	4.93E-66	-4.75	no hit	-	-	-	-
-	Tag_3282 CATGGGACTCACCGAGTACGACGCCT	3	20	0.761	19.987	2.90E-12	-4.71	no hit	-	-	-	-
-	Tag_3871 CATGCGCCCGCGCCGGCGTGAGGTGA	2	13	0.508	12.992	1.54E-08	-4.68	no hit	-	-	-	-
Primary metabolic process/oxidoreductase activity	Tag_3899 CATGGTCCACTCACCACTGACGAGGG	2	13	0.508	12.992	1.54E-08	-4.68	Hv_Contig_11717	1036	791-816	XP_013192568|carbonyl reductase [NADPH] 3-like *Amyelois transitella*	3.00E-142
Primary metabolic process/hydrolase activity	Tag_1327 CATGGCCATCAACAACGCCCTGGTCG	20	127	5.075	126.919	5.15E-67	-4.64	Hv_Contig_22796	649	301-326	AHL46496|trypsin *Helicoverpa armigera*	2.00E-82
Cytoskeleton	Tag_159 CATGCCCGGCCACCAGCTGTTCGCGC	442	2801	112.166	2799.203	0.00E+00	-4.64	Hv_Contig_19906	725	416-391	XP_012553210|epidermal growth factor receptor kinase substrate 8-like isoform X3 *Bombyx mori*	2.00E-65
-	Tag_840 CATGGGACTCACCGAGTACGACGCTG	44	273	11.166	272.825	9.69E-141	-4.61	no hit	-	-	-	-
-	Tag_2229 CATGCCTGGGTGTGCCACCGCGCTCG	7	43	1.776	42.972	7.53E-24	-4.60	no hit	-	-	-	-
-	Tag_619 CATGCCGTGAACCACGCCGTGGCAGC	79	481	20.048	480.691	4.70E-245	-4.58	no hit	-	-	-	-
-	Tag_3643 CATGATTCATTACTTGTACTGTAATG	2	######	0.508	11.992	6.67007E-08	-4.56245111	no hit	-	-	-	-
-	Tag_3916 CATGAGGCAGACGGTCGCTGTGGAGG	2	12	0.508	11.992	6.67E-08	-4.56	no hit	-	-	-	-
-	Tag_3920 CATGCGGCGCCTCGCGCACCCCGACA	2	12	0.508	11.992	6.67E-08	-4.56	no hit	-	-	-	-
-	Tag_3981 CATGCACTGCCGCCGGGGCACTGCCC	2	12	0.508	11.992	6.67E-08	-4.56	no hit	-	-	-	-
-	Tag_2568 CATGAGCCAGGAGGTGGACGTGCCCG	5	30	1.269	29.981	4.00E-17	-4.56	no hit	-	-	-	-
-	Tag_4091 CATGTGGGAGACCTTCGCCACCAAAT	1	6	0.254	5.996	9.19E-05	-4.56	no hit	-	-	-	-
-	Tag_4092 CATGATCTGAGTTCGAACCGGTGTAG	1	6	0.254	5.996	9.19E-05	-4.56	no hit	-	-	-	-
-	Tag_4103 CATGCTGGTCGCGGTGGCGTTCGCCG	1	6	0.254	5.996	9.19E-05	-4.56	no hit	-	-	-	-
-	Tag_4127 CATGGAGTTGGGCGGATGACATTTGA	1	6	0.254	5.996	9.19E-05	-4.56	no hit	-	-	-	-
-	Tag_4135 CATGTCCGTGCGCTCCAGCGATCGTG	1	6	0.254	5.996	9.19E-05	-4.56	no hit	-	-	-	-
-	Tag_4151 CATGTCAAAGCTGCGCCCTTGCTCCA	1	6	0.254	5.996	9.19E-05	-4.56	no hit	-	-	-	-
-	Tag_4199 CATGCTGCGCCCACGTGCGGCGCGGC	1	6	0.254	5.996	9.19E-05	-4.56	no hit	-	-	-	-
-	Tag_4259 CATGCCCACGTGCGGCGCCTTCACGC	1	6	0.254	5.996	9.19E-05	-4.56	no hit	-	-	-	-
mRNA processing/splicing	Tag_4475 CATGAGTTTGTGTTGACAAATGCAGA	1	6	0.254	5.996	9.19E-05	-4.56	Hv_Contig_3175	1994	158-133	XP_013195286|plasminogen activator inhibitor 1 RNA-binding protein-like *Amyelois transitella*	1.00E-111
-	Tag_4502 CATGATTCCACACAAGACTGAGCGCT	1	6	0.254	5.996	9.19E-05	-4.56	no hit	-	-	-	-
-	Tag_4650 CATGGCCCCTGCGCAAGGATGACACA	1	6	0.254	5.996	9.19E-05	-4.56	no hit	-	-	-	-
Primary metabolic process/fatty-acyl-CoA binding	Tag_4711 CATGGGCTACCTCAACGATGACGCGA	1	6	0.254	5.996	9.19E-05	-4.56	Hv_Contig_9310	1188	378-403	XP_004930170|very long-chain-fatty-acid--CoA ligase bubblegum isoform X2 *Bombyx mori*	0.00E+00
-	Tag_4727 CATGGGACTCACCGAGTACGATGCCG	1	6	0.254	5.996	9.19E-05	-4.56	no hit	-	-	-	-
-	Tag_4755 CATGCTGCGGCAACGCGGCGGCGCAC	1	6	0.254	5.996	9.19E-05	-4.56	no hit	-	-	-	-
-	Tag_4763 CATGTGCACGCTGGCTCCCTGTGGGT	1	6	0.254	5.996	9.19E-05	-4.56	no hit	-	-	-	-
-	Tag_4963 CATGATCCGCGAACGAAGTCGCTGGT	1	6	0.254	5.996	9.19E-05	-4.56	no hit	-	-	-	-
-	Tag_4977 CATGGGAACTACTGCGTGCACCCGGA	1	6	0.254	5.996	9.19E-05	-4.56	no hit	-	-	-	-
Chitin-binding	Tag_5002 CATGAAGCCGACGGTTCCATCAGAAC	1	6	0.254	5.996	9.19E-05	-4.56	Hv_Contig_27599	569	328-353	NP_001166668|cuticular protein RR-2 motif 97 precursor *Bombyx mori*	5.00E-37
-	Tag_5022 CATGCGTCGGCCGGGAGGCGCTCAGA	1	6	0.254	5.996	9.19E-05	-4.56	no hit	-	-	-	-
-	Tag_5031 CATGCGGCCCGCGAACGTCTTACTTA	1	6	0.254	5.996	9.19E-05	-4.56	no hit	-	-	-	-
Transport/Trafficking	Tag_5127 CATGTGAGGACTTTCACACCAGAGGT	1	6	0.254	5.996	9.19E-05	-4.56	Hv_Contig_11768	1001	423-448	NP_001299708|fatty acid-binding protein-like *Papilio xuthus*	2.00E-46
-	Tag_5236 CATGGGGCCGTGACTGCGCTGATGCG	1	6	0.254	5.996	9.19E-05	-4.56	no hit	-	-	-	-
-	Tag_1568 CATGTGCGCGCTGGCTCCCTGTGGGA	15	87	3.807	86.944	1.33E-45	-4.51	no hit	-	-	-	-

-	Tag_581 CATGGCTCGTGGTCACCTTGCCGCGA	87	492	22.078	491.684	4.89E-246	-4.48	no hit	-	-	-	-
Transport/Trafficking	Tag_2601 CATGGTGCAGCTACTCAGCGTGGTGC	5	28	1.269	27.982	7.22E-16	-4.46	Hv_Contig_35307	405	271-296	XP_013197877|solute carrier family 35 member E1 homolog *Amyelois transitella*	2.00E-36
Protein folding/Recycling	Tag_2655 CATGTTGTAATGGTGTTTATGTGATT	5	28	1.269	27.982	7.22E-16	-4.46	Hv_Contig_23072	642	519-544	ACO58577|heat shock protein 90 *Apis mellifera*	2.00E-44
Primary metabolic process/oxidoreductase activity	Tag_3892 CATGAAGATCTTCGTATACAGTCCTC	2	11	0.508	10.993	2.86E-07	-4.44	Hv_Contig_20572	707	434-459	KPJ17487|Cytochrome c oxidase subunit 4 isoform 1, mitochondrial *Papilio machaon*	2.00E-69
-	Tag_3993 CATGAGCTCAGACGTCGCGGCCTCAT	2	11	0.508	10.993	2.86E-07	-4.44	no hit	-	-	-	-
-	Tag_2989 CATGCCCGGCCACCAGCTGTTCGCAC	4	22	1.015	21.986	8.28E-13	-4.44	no hit	-	-	-	-
Translation/Ribosome biogenesis	Tag_2444 CATGCGGACGTGGAACTGATCCTTGC	6	32	1.523	31.979	1.14E-17	-4.39	Hv_Contig_19497	737	431-406	ABX54738|ribosomal protein L10 *Spodoptera exigua*	2.00E-90
-	Tag_3276 CATGGCGCTGCAGGTGGACGTGTCTG	3	16	0.761	15.990	9.77E-10	-4.39	no hit	-	-	-	-
-	Tag_519 CATGGGACTCACCGAGTACGACGCCA	104	541	26.392	540.653	2.16E-264	-4.36	no hit	-	-	-	-
Nucleic acid binding	Tag_2626 CATGCCTGCTATGCTCTTTGTTGAAC	5	26	1.269	25.983	1.28E-14	-4.36	Hv_Contig_8800	1221	1140-1165	XP_013188449|TAR DNA-binding protein 43-like *Amyelois transitella*	7.00E-167
-	Tag_4056 CATGAATCCAATAAGCAGATCGCTGT	1	5	0.254	4.997	3.99E-04	-4.30	no hit	-	-	-	-
-	Tag_4165 CATGTGTGCGCGGACGCACGGGACCG	1	5	0.254	4.997	3.99E-04	-4.30	no hit	-	-	-	-
-	Tag_4174 CATGCGACCACCGCGCGGCGCGACGC	1	5	0.254	4.997	3.99E-04	-4.30	no hit	-	-	-	-
-	Tag_4181 CATGATCTGAGTTCAAACCGGTGGAA	1	5	0.254	4.997	3.99E-04	-4.30	no hit	-	-	-	-
-	Tag_4212 CATGTTGTCGTTGGGGCCTTAGACAT	1	5	0.254	4.997	3.99E-04	-4.30	no hit	-	-	-	-
Chitin-binding	Tag_4245 CATGGACCCGCCGAACAGAGGCTCCA	1	5	0.254	4.997	3.99E-04	-4.30	Hv_Contig_12755	984	144-169	AEA76329|chitin binding domain 3 protein *Mamestra configurata*	1.00E-134
-	Tag_4306 CATGCCCGGTCACCAGCTGTTCGCGC	1	5	0.254	4.997	3.99E-04	-4.30	no hit	-	-	-	-
-	Tag_4371 CATGTTGCTTCGCTGTGACCGCCGTA	1	5	0.254	4.997	3.99E-04	-4.30	no hit	-	-	-	-
-	Tag_4394 CATGCTCCCGGTCGGCAGCGATGGGG	1	5	0.254	4.997	3.99E-04	-4.30	no hit	-	-	-	-
mRNA processing/splicing	Tag_4406 CATGCCAGCCGGTTTCCGCATAATTT	1	5	0.254	4.997	3.99E-04	-4.30	Hv_Contig_37137	362	277-252	XP_011015409|pre-mRNA-splicing factor 38A-like *Populus euphratica*	5.00E-24
-	Tag_4489 CATGCCCGGCCGCCAGCTGTTCGCGC	1	5	0.254	4.997	3.99E-04	-4.30	no hit	-	-	-	-
-	Tag_4527 CATGCTGCTGTTAGCAATAAAGAGTA	1	5	0.254	4.997	3.99E-04	-4.30	no hit	-	-	-	-
-	Tag_4528 CATGATCGTCCGAGACACGCCCCCTC	1	5	0.254	4.997	3.99E-04	-4.30	no hit	-	-	-	-
-	Tag_4539 CATGTAATCTGAAATTTATATTTTTT	1	5	0.254	4.997	3.99E-04	-4.30	no hit	-	-	-	-
-	Tag_4606 CATGTCCGGCATCGTGCAGAGCGTCA	1	5	0.254	4.997	3.99E-04	-4.30	no hit	-	-	-	-
-	Tag_4608 CATGGAGCCGTGGCTGCGCTGATGCT	1	5	0.254	4.997	3.99E-04	-4.30	no hit	-	-	-	-
-	Tag_4624 CATGGGACCGTGGCTGCGCTGATGCT	1	5	0.254	4.997	3.99E-04	-4.30	no hit	-	-	-	-
Nucleic acid binding	Tag_4639 CATGCGGCTCTTTGGATGGGAAGCTG	1	5	0.254	4.997	3.99E-04	-4.30	Hv_Contig_11302	1060	457-482	XP_010462852|zinc finger MYM-type protein 1-like *Camelina sativa*	1.00E-08
-	Tag_4786 CATGCCCGGCCACCAGCTGTCCGCGC	1	5	0.254	4.997	3.99E-04	-4.30	no hit	-	-	-	-
-	Tag_4831 CATGCCCCAGCGCAGCACCGAGGACG	1	5	0.254	4.997	3.99E-04	-4.30	no hit	-	-	-	-
-	Tag_4834 CATGGGACTCACCGAGTACGACGCGG	1	5	0.254	4.997	3.99E-04	-4.30	no hit	-	-	-	-
-	Tag_4864 CATGGTAGCGAGCGTGCGCCCCATCG	1	5	0.254	4.997	3.99E-04	-4.30	no hit	-	-	-	-
Transport/Trafficking	Tag_4871 CATGACTGCGAAGCCGAGGAGTGCGC	1	5	0.254	4.997	3.99E-04	-4.30	Hv_Contig_12596	992	456-431	XP_013183892|zinc transporter ZIP1-like *Amyelois transitella*	4.00E-69
-	Tag_4889 CATGACAGCGAGCAAGAAACCAAAAG	1	5	0.254	4.997	3.99E-04	-4.30	no hit	-	-	-	-
-	Tag_4894 CATGGCCGACGCTACGCTGCTGCCCA	1	5	0.254	4.997	3.99E-04	-4.30	no hit	-	-	-	-
Primary metabolic process/hydrolase activity	Tag_4925 CATGCCGGCAGCCCTGGCTGCCTTCA	1	5	0.254	4.997	3.99E-04	-4.30	Hv_Contig_11981	1023	702-677	XP_013186138|pseudouridine-5'-phosphatase-like *Amyelois transitella*	1.00E-124
-	Tag_4943 CATGGGACTCACCGAGTACGACGCAA	1	5	0.254	4.997	3.99E-04	-4.30	no hit	-	-	-	-
-	Tag_5000 CATGGGATGCGAGTGAAACCGCGGAA	1	5	0.254	4.997	3.99E-04	-4.30	no hit	-	-	-	-
-	Tag_5009 CATGCCTGAGTTTCGACGCCTGGCAG	1	5	0.254	4.997	3.99E-04	-4.30	no hit	-	-	-	-
-	Tag_5066 CATGCATGGGAAAGTGGCCCGGCTCT	1	5	0.254	4.997	3.99E-04	-4.30	no hit	-	-	-	-
-	Tag_5200 CATGGCGACACGCGCCTAAGTGCGGT	1	5	0.254	4.997	3.99E-04	-4.30	no hit	-	-	-	-
-	Tag_5226 CATGGAACTCGAACGCTCAGGCAAAA	1	5	0.254	4.997	3.99E-04	-4.30	no hit	-	-	-	-
-	Tag_3451 CATGCTGTCACCCACAGCGGGAGGGG	2	10	0.508	9.994	1.22E-06	-4.30	no hit	-	-	-	-
-	Tag_3483 CATGGCCTACGCCGCCTGCATCGGTC	2	10	0.508	9.994	1.22E-06	-4.30	no hit	-	-	-	-
-	Tag_3724 CATGCGCGAGTCCAACTCGCACTTGT	2	10	0.508	9.994	1.22E-06	-4.30	no hit	-	-	-	-
-	Tag_3789 CATGGCCGACTATCCGCAGCGCGTAT	2	10	0.508	9.994	1.22E-06	-4.30	no hit	-	-	-	-
-	Tag_3816 CATGGTACTCGAGTCATCGCGGCCGC	2	10	0.508	9.994	1.22E-06	-4.30	no hit	-	-	-	-
-	Tag_3908 CATGTGCGCGCTGGCTCCCTGTGGAT	2	10	0.508	9.994	1.22E-06	-4.30	no hit	-	-	-	-
Translation/Ribosome biogenesis	Tag_4016 CATGATTCATTACTTGTACTGTAATT	2	10	0.508	9.994	1.22E-06	-4.30	Hv_Contig_1020	3004	2938-2963	AAL83698|translation elongation factor 2 *Spodoptera exigua*	0.00E+00
-	Tag_3267 CATGCGCTAGAGACAGCGTCGTGCCT	3	15	0.761	14.990	4.11E-09	-4.30	no hit	-	-	-	-
Unknown	Tag_3019 CATGATGGCGTCCACGCACGTGTACG	4	20	1.015	19.987	1.46E-11	-4.30	Hv_Contig_6655	1417	1243-1268	EHJ78723|hypothetical protein KGM_00713 *Danaus plexippus*	0.00E+00
-	Tag_1150 CATGGTTGGTGCGCGCGGGGGCCTTT	25	120	6.344	119.923	4.14E-59	-4.24	no hit	-	-	-	-
-	Tag_2040 CATGGGACTCACCGAGTACGACGCAG	9	42	2.284	41.973	7.61E-22	-4.20	no hit	-	-	-	-
-	Tag_3206 CATGGTTGGTGCGCGCGGGGGCCTTG	3	14	0.761	13.991	1.72E-08	-4.20	no hit	-	-	-	-
-	Tag_1432 CATGCTGCCCCCGACTGCCGCGCGAG	18	83	4.568	82.947	4.49E-41	-4.18	no hit	-	-	-	-
Unknown	Tag_1071 CATGCGCGCCGCGGCGCTGCGCTGCG	28	127	7.106	126.919	2.00E-61	-4.16	Hv_Contig_2680	2130	91-116	XP_004925890|uncharacterized protein LOC101737344 *Bombyx mori*	5.00E-24
-	Tag_3455 CATGTCCGCGAACGGGTCGGCGGCGG	2	9	0.508	8.994	5.10E-06	-4.15	no hit	-	-	-	-
-	Tag_3544 CATGCCCGGCCACCGGCTGTTCGCGC	2	9	0.508	8.994	5.10E-06	-4.15	no hit	-	-	-	-

Protein folding/Recycling	Tag_3631 CATGTACGCGCCGAGGCCGGTGGCAA	2	9	0.508	8.994	5.10E-06	-4.15	Hv_Contig_10425	1028	653-628	XP_013183557|proteasome subunit beta type-4 *Amyelois transitella*	2.00E-161
-	Tag_3751 CATGGGCGGATGCGGCGCGACCGGCG	2	9	0.508	8.994	5.10E-06	-4.15	no hit	-	-	-	-
-	Tag_3799 CATGCCCGTACAGAAGCAGACCCATG	2	9	0.508	8.994	5.10E-06	-4.15	no hit	-	-	-	-
-	Tag_3857 CATGATCGTCCGAGACACGCCCCCAG	2	9	0.508	8.994	5.10E-06	-4.15	no hit	-	-	-	-
-	Tag_3904 CATGATTCATTACTTGTACTGTAATC	2	9	0.508	8.994	5.10E-06	-4.15	no hit	-	-	-	-
-	Tag_2250 CATGTTGTAATGGTGTTTATGTGATG	7	31	1.776	30.980	2.13E-16	-4.12	no hit	-	-	-	-
-	Tag_2600 CATGCGTGTCCAGCCGGCCCGGCCAG	5	22	1.269	21.986	3.74E-12	-4.11	no hit	-	-	-	-
-	Tag_892 CATGAGGCAGACGGTCGCTGTGGGAG	38	159	9.643	158.898	1.69E-74	-4.04	no hit	-	-	-	-
Unknown	Tag_1671 CATGCTGCTGTCGCTCGCCGTCGTCG	13	54	3.299	53.965	1.26E-26	-4.03	Hv_Contig_6366	1449	56-31	XP_004921893|putative uncharacterized protein DDB_G0282133 *Bombyx mori*	0.00E+00
Primary metabolic process/oxidoreductase activity	Tag_261 CATGTGCGCGCTGGTTCCTTGTGGGT	239	982	60.651	981.370	0.00E+00	-4.02	Hv_Contig_17403	800	83-108	AGV76311|mitochondrial aldehyde dehydrogenase, partial *Spodoptera litura*	1.00E-29
-	Tag_3319 CATGTCCGTGCGCTCCAGCGACCGAA	3	12	0.761	11.992	2.89E-07	-3.98	no hit	-	-	-	-
-	Tag_3510 CATGTGGCGGCCGACGGACAAAACGA	2	8	0.508	7.995	2.11E-05	-3.98	no hit	-	-	-	-
-	Tag_3641 CATGTGACGGTCCCCGCACACGGCGC	2	8	0.508	7.995	2.11E-05	-3.98	no hit	-	-	-	-
-	Tag_3656 CATGCTGCTAGGCGCCGAGCGCGCCG	2	8	0.508	7.995	2.11E-05	-3.98	no hit	-	-	-	-
-	Tag_3705 CATGCCGCGCCTCACCGCGACGCCGG	2	8	0.508	7.995	2.11E-05	-3.98	no hit	-	-	-	-
-	Tag_3798 CATGCACGGGAAAGTGGCCCGGCCCT	2	8	0.508	7.995	2.11E-05	-3.98	no hit	-	-	-	-
-	Tag_3853 CATGCATGGCAGCTGGAACCCCAACA	2	8	0.508	7.995	2.11E-05	-3.98	no hit	-	-	-	-
-	Tag_3878 CATGGCCATGACGGTGGCCTTGTGTG	2	8	0.508	7.995	2.11E-05	-3.98	no hit	-	-	-	-
-	Tag_4069 CATGGGCCGTGGCTGCGCTGATGCTT	1	4	0.254	3.997	1.70E-03	-3.98	no hit	-	-	-	-
Protein kinases	Tag_4074 CATGCAAGCGCGGCTCGACGCGCCCC	1	4	0.254	3.997	1.70E-03	-3.98	Hv_Contig_9514	1173	728-753	XP_013184730|serine/threonine-protein kinase Genghis Khan *Amyelois transitella*	0.00E+00
-	Tag_4079 CATGCGCTAGAGACAGCGTCGTGCCC	1	4	0.254	3.997	1.70E-03	-3.98	no hit	-	-	-	-
-	Tag_4083 CATGGGACTCACCGTGTACGACGCCG	1	4	0.254	3.997	1.70E-03	-3.98	no hit	-	-	-	-
-	Tag_4095 CATGGAGGCGCTGCTCGGCTGAGCTC	1	4	0.254	3.997	1.70E-03	-3.98	no hit	-	-	-	-
-	Tag_4120 CATGAATGCCGCTACGCTGATTTCTG	1	4	0.254	3.997	1.70E-03	-3.98	no hit	-	-	-	-
-	Tag_4140 CATGAGGCGGACGGTCGCTGTGGGTA	1	4	0.254	3.997	1.70E-03	-3.98	no hit	-	-	-	-
-	Tag_4148 CATGGGACCAACAAGGCAAGAACAGC	1	4	0.254	3.997	1.70E-03	-3.98	no hit	-	-	-	-
-	Tag_4163 CATGGCCCTGGCGGCGCTGGGCGTGA	1	4	0.254	3.997	1.70E-03	-3.98	no hit	-	-	-	-
-	Tag_4169 CATGCCCGGCTTCCCCGACGGCGCGT	1	4	0.254	3.997	1.70E-03	-3.98	no hit	-	-	-	-
-	Tag_4333 CATGCTGGACCTGGCGCAGGTGGCTC	1	4	0.254	3.997	1.70E-03	-3.98	no hit	-	-	-	-
-	Tag_4336 CATGTCCGTGGCTCACGTGTCCTCGA	1	4	0.254	3.997	1.70E-03	-3.98	no hit	-	-	-	-
-	Tag_4338 CATGGCTCTAGTCGGTGTCTGCAGGG	1	4	0.254	3.997	1.70E-03	-3.98	no hit	-	-	-	-
Unknown	Tag_4382 CATGATTTCTGTCACGGCTCCGGCGC	1	4	0.254	3.997	1.70E-03	-3.98	Hv_Contig_2751	2112	1585-1560	KOB72012|Uncharacterized protein OBRU01_13167 *Operophtera brumata*	1.00E-92
-	Tag_4430 CATGCCTGGGTGTGCCACCGCGCTTT	1	4	0.254	3.997	1.70E-03	-3.98	no hit	-	-	-	-
-	Tag_4435 CATGAAAATTGCAATCAGTTCTACAG	1	4	0.254	3.997	1.70E-03	-3.98	no hit	-	-	-	-
Iron homeostasis	Tag_4466 CATGTCGCTCTTTGTGTGCCCTAATT	1	4	0.254	3.997	1.70E-03	-3.98	Hv_Contig_7833	1302	835-860	XP_013188441|cytosolic Fe-S cluster assembly factor NUBP1 homolog *Amyelois transitella*	0.00E+00
Transport/Trafficking	Tag_4506 CATGCTCATCTTCACAACGATGGCTT	1	4	0.254	3.997	1.70E-03	-3.98	Hv_Contig_2221	2303	2163-2188	XP_013189413|peptide transporter family 1 isoform X2 *Amyelois transitella*	0.00E+00
-	Tag_4536 CATGGAGGACGCCGCGGCACCCACGT	1	4	0.254	3.997	1.70E-03	-3.98	no hit	-	-	-	-
Translation/Ribosome biogenesis	Tag_4543 CATGGGTAATGAGATGGCTCACTATG	1	4	0.254	3.997	1.70E-03	-3.98	Hv_Contig_1866	2439	1366-1391	XP_013166978|glycine--tRNA ligase *Papilio xuthus*	0.00E+00
-	Tag_4551 CATGTGCGACGTTCTTTGTGACGTGA	1	4	0.254	3.997	1.70E-03	-3.98	no hit	-	-	-	-
Primary metabolic process/oxidoreductase activity	Tag_4562 CATGAAGTCAAGAGTTCCCATTGAAG	1	4	0.254	3.997	1.70E-03	-3.98	Hv_Contig_2948	2057	411-436	XP_013194058|glutamate dehydrogenase, mitochondrial isoform X1 *Amyelois transitella*	0.00E+00
-	Tag_4610 CATGATCTGTGCTGCCCAACTCGGCG	1	4	0.254	3.997	1.70E-03	-3.98	no hit	-	-	-	-
-	Tag_4629 CATGCCCGGCTTTCCCGGCGGCGCGA	1	4	0.254	3.997	1.70E-03	-3.98	no hit	-	-	-	-
-	Tag_4673 CATGGCAGTGGCGGCGCCTAAGCCAA	1	4	0.254	3.997	1.70E-03	-3.98	no hit	-	-	-	-
-	Tag_4688 CATGAACAATTAAAAAATACTGTAAT	1	4	0.254	3.997	1.70E-03	-3.98	no hit	-	-	-	-
-	Tag_4694 CATGGTAATGATTATTTTCAAAGATC	1	4	0.254	3.997	1.70E-03	-3.98	no hit	-	-	-	-
-	Tag_4699 CATGGGGCCGTGTCTGCGCTGATGCT	1	4	0.254	3.997	1.70E-03	-3.98	no hit	-	-	-	-
-	Tag_4734 CATGAATGAAACCAAAATATATTAAG	1	4	0.254	3.997	1.70E-03	-3.98	no hit	-	-	-	-
-	Tag_4741 CATGATCTGTGCCGGCTGGCCCAGGA	1	4	0.254	3.997	1.70E-03	-3.98	no hit	-	-	-	-
Protein folding/Recycling	Tag_4803 CATGGACTGAAATCCCTGTGCCTAAT	1	4	0.254	3.997	1.70E-03	-3.98	Hv_Contig_2864	2077	2028-2053	XP_004932233|gamma-glutamyltranspeptidase 1 isoform X4 *Bombyx mori*	0.00E+00
-	Tag_4849 CATGGCTCGCGAGCTGTCCGGCACAG	1	4	0.254	3.997	1.70E-03	-3.98	no hit	-	-	-	-
-	Tag_4853 CATGCGCGCCGCGGCGCTGCGCTGAG	1	4	0.254	3.997	1.70E-03	-3.98	no hit	-	-	-	-
-	Tag_4855 CATGGAGGGCGACGCGTACGGCGGCG	1	4	0.254	3.997	1.70E-03	-3.98	no hit	-	-	-	-
-	Tag_4872 CATGGGTGGTGCACCTCCGCGTTGTC	1	4	0.254	3.997	1.70E-03	-3.98	no hit	-	-	-	-
-	Tag_4909 CATGAAGATTGGCCGCATTGAGGAGA	1	4	0.254	3.997	1.70E-03	-3.98	no hit	-	-	-	-
-	Tag_4920 CATGCGCGCGCCGCCGCTGCACCTCG	1	4	0.254	3.997	1.70E-03	-3.98	no hit	-	-	-	-
mRNA processing/splicing	Tag_4947 CATGCCAGAGTATGAAGTGATGTCGA	1	4	0.254	3.997	1.70E-03	-3.98	Hv_Contig_3865	1846	1663-1688	XP_004931734|pre-mRNA-splicing regulator female-lethal(2)D *Bombyx mori*	1.00E-163
Translation/Ribosome biogenesis	Tag_4950 CATGATCTTGAGGACCTCATCGTTCA	1	4	0.254	3.997	1.70E-03	-3.98	Hv_Contig_23862	627	146-121	BAJ23427|ribosomal protein S2, partial *Ostrinia nubilalis*	3.00E-102
-	Tag_4965 CATGCACGAGAAAGTGGCCCGGCTCT	1	4	0.254	3.997	1.70E-03	-3.98	no hit	-	-	-	-

-	Tag_5021 CATGATCTGTGCTGCCCAACTCGGAG	1	4	0.254	3.997	1.70E-03	-3.98	no hit	-	-	-	-
-	Tag_5033 CATGGGGTACCTGGCAAAAGCTTTAG	1	4	0.254	3.997	1.70E-03	-3.98	no hit	-	-	-	-
-	Tag_5037 CATGCTGCCCTCGACTGCCGCGCGCG	1	4	0.254	3.997	1.70E-03	-3.98	no hit	-	-	-	-
-	Tag_5085 CATGTCGACGCTGGAGACGGCGGCCG	1	4	0.254	3.997	1.70E-03	-3.98	no hit	-	-	-	-
Signal transduction	Tag_5091 CATGAGCGTGAGGAAAGACGGCGCGA	1	4	0.254	3.997	1.70E-03	-3.98	Hv_Contig_1388	2714	1970-1995	XP_013196029|RING finger protein 10 *Amyelois transitella*	0.00E+00
-	Tag_5125 CATGGGGCCTCACAAGGGCAAGGCGG	1	4	0.254	3.997	1.70E-03	-3.98	no hit	-	-	-	-
-	Tag_5149 CATGCCGCCCCCGACTGCCGCGCGCG	1	4	0.254	3.997	1.70E-03	-3.98	no hit	-	-	-	-
Signal transduction	Tag_5187 CATGCAGACCTACTTAGGTAACACCA	1	4	0.254	3.997	1.70E-03	-3.98	Hv_Contig_1862	2441	2184-2209	XP_011558285|WD and tetratricopeptide repeats protein 1 *Plutella xylostella*	0.00E+00
Cytoskeleton	Tag_5194 CATGGGGCGGCGCGGGCGCGCGGCGT	1	4	0.254	3.997	1.70E-03	-3.98	Hv_Contig_3788	1862	1603-1628	XP_013179701|serine/arginine repetitive matrix protein 1-like *Papilio xuthus*	4.00E-151
Primary metabolic process/hydrolase activity	Tag_5213 CATGGAGGAGTTCGTGCAAGTGGCCA	1	4	0.254	3.997	1.70E-03	-3.98	Hv_Contig_15470	869	753-778	XP_004922526|dual specificity protein phosphatase 23-like *Bombyx mori*	1.00E-139
-	Tag_5235 CATGCTGGGCGCGCTGTTCCACTACA	1	4	0.254	3.997	1.70E-03	-3.98	no hit	-	-	-	-
-	Tag_2755 CATGCGTGCCGGCTCGCTGGTCGGCC	4	16	1.015	15.990	4.15E-09	-3.98	no hit	-	-	-	-
-	Tag_1034 CATGTTGTGGACTCCGTTGCTGAGCC	30	117	7.613	116.925	3.58E-54	-3.94	no hit	-	-	-	-
-	Tag_2081 CATGCACGGCAGCTGGACCCCTAGCA	8	31	2.030	30.980	8.58E-16	-3.93	no hit	-	-	-	-
-	Tag_554 CATGGGGCCGTGGCTGCGCTGATGCC	94	360	23.854	359.769	5.53E-161	-3.91	no hit	-	-	-	-
-	Tag_1683 CATGAGCTCAGACGTCGCGGCCTCAG	13	49	3.299	48.969	1.24E-23	-3.89	no hit	-	-	-	-
-	Tag_2105 CATGATTGTGTGAGCCTGTGCGGCAG	8	30	2.030	29.981	3.39E-15	-3.88	no hit	-	-	-	-
-	Tag_2906 CATGGGACTCACCGAGTACGGCGCCG	4	15	1.015	14.990	1.67E-08	-3.88	no hit	-	-	-	-
-	Tag_2976 CATGTGGTAGACTACATCAACCCTAG	4	15	1.015	14.990	1.67E-08	-3.88	no hit	-	-	-	-
-	Tag_2325 CATGAGGCAGACGGTCGCTGTGGGAA	7	26	1.776	25.983	2.21E-13	-3.87	no hit	-	-	-	-
-	Tag_3028 CATGCACCGCCGTGGGGCCGCGCCTT	3	11	0.761	10.993	1.16E-06	-3.85	no hit	-	-	-	-
-	Tag_3102 CATGAGGCAGACGGTCGCTGTGGGAC	3	11	0.761	10.993	1.16E-06	-3.85	no hit	-	-	-	-
-	Tag_3204 CATGGGACCAACAAGGCAAGAACGGG	3	11	0.761	10.993	1.16E-06	-3.85	no hit	-	-	-	-
-	Tag_2030 CATGCCCGGGGCGCTGTGGCCAGCAC	9	33	2.284	32.979	2.06E-16	-3.85	no hit	-	-	-	-
-	Tag_2400 CATGGGGCCGCGGCTGCGCTGATGCT	6	22	1.523	21.986	1.46E-11	-3.85	no hit	-	-	-	-
-	Tag_1790 CATGGTAATGATTATTTTCAAAGATT	11	40	2.791	39.974	1.97E-19	-3.84	no hit	-	-	-	-
-	Tag_2543 CATGAACGTGCCCCCTGCTACCTCGG	5	18	1.269	17.988	9.72E-10	-3.83	no hit	-	-	-	-
-	Tag_2560 CATGCTGGCGGCGCGCTACTCGCCGA	5	18	1.269	17.988	9.72E-10	-3.83	no hit	-	-	-	-
Translation/Ribosome biogenesis	Tag_2643 CATGATATGGCACAGCGTGCAAGCCC	5	18	1.269	17.988	9.72E-10	-3.83	Hv_Contig_10264	1121	735-760	P62924|Eukaryotic translation initiation factor 5A *Spodoptera exigua*	9.00E-110
-	Tag_956 CATGTGCGCGCTGGTTCCTTGTGGGC	34	122	8.628	121.922	7.89E-55	-3.82	no hit	-	-	-	-
Protein folding/Recycling	Tag_1134 CATGAGCCAGGAGGTGGACGTGCCCA	26	92	6.598	91.941	1.13E-41	-3.80	Hv_Contig_7932	1291	722-747	XP_004930715|E3 ubiquitin-protein ligase Mdm2-like *Bombyx mori*	2.00E-83
-	Tag_3551 CATGGGGCTAGCCGACCGTGTGTGAG	2	7	0.508	6.996	8.56E-05	-3.78	no hit	-	-	-	-
-	Tag_3586 CATGCTCAGAATTAATACGACAATTT	2	7	0.508	6.996	8.56E-05	-3.78	no hit	-	-	-	-
-	Tag_3655 CATGATCACAGATCGCGCCCAGGCCC	2	7	0.508	6.996	8.56E-05	-3.78	no hit	-	-	-	-
-	Tag_3707 CATGGCGGAAGGGTTAGTAGAAAATG	2	7	0.508	6.996	8.56E-05	-3.78	no hit	-	-	-	-
-	Tag_3858 CATGAGCGGGATCTCTCTCAGCCCTG	2	7	0.508	6.996	8.56E-05	-3.78	no hit	-	-	-	-
-	Tag_3865 CATGCCAGGGTGCATCACCTCGATGG	2	7	0.508	6.996	8.56E-05	-3.78	no hit	-	-	-	-
-	Tag_3925 CATGATTGCCGTCGTGGTCGGAAGAG	2	7	0.508	6.996	8.56E-05	-3.78	no hit	-	-	-	-
Protein folding/Recycling	Tag_4006 CATGCGCGAGCAGATGGCGCGCGAGT	2	7	0.508	6.996	8.56E-05	-3.78	Hv_Contig_43902	243	190-215	XP_011551239|LIX1-like protein *Plutella xylostella*	2.00E-40
Primary metabolic process/oxidoreductase activity	Tag_2793 CATGTCGGTCAACGACCTGCCTGTTG	4	14	1.015	13.991	6.60E-08	-3.78	Hv_Contig_4868	1662	640-665	XP_013185136|peroxiredoxin-2-like *Amyelois transitella*	2.00E-135
-	Tag_2833 CATGCCCAGCGTCAGCGCCGCGCCCG	4	14	1.015	13.991	6.60E-08	-3.78	no hit	-	-	-	-
-	Tag_1422 CATGATTGTGTGAGCCTGTGCGGCAA	18	61	4.568	60.961	6.51E-28	-3.74	no hit	-	-	-	-
-	Tag_3099 CATGCACGGGAAAGTGGCTCGGCTCT	3	10	0.761	9.994	4.62E-06	-3.71	no hit	-	-	-	-
-	Tag_3359 CATGACAGGGAACTGTCACCTATGGG	3	10	0.761	9.994	4.62E-06	-3.71	no hit	-	-	-	-
-	Tag_2438 CATGATCGTCCGAGACACGCCCCCAA	6	20	1.523	19.987	2.23E-10	-3.71	no hit	-	-	-	-
-	Tag_1141 CATGCACGGCAGCTGGACCCCTAGCG	26	86	6.598	85.945	3.62E-38	-3.70	no hit	-	-	-	-
-	Tag_1801 CATGGCCTACGCCGCCTGCATCGGCA	11	36	2.791	35.977	4.41E-17	-3.69	no hit	-	-	-	-
Unknown	Tag_1495 CATGCAATCAGAACTCTGACCAGTGA	16	52	4.060	51.967	9.65E-24	-3.68	Hv_Contig_4291	1763	1080-1105	XP_001892989|hypothetical protein Bm1_07595 *Brugia malayi*	4.00E-23
-	Tag_3002 CATGGGGCCGTGGCCGCGCTGATGCT	4	13	1.015	12.992	2.59E-07	-3.68	no hit	-	-	-	-
-	Tag_3018 CATGAGCGGGATCTCTCTCAGCCCCA	4	13	1.015	12.992	2.59E-07	-3.68	no hit	-	-	-	-
Translation/Ribosome biogenesis	Tag_2460 CATGGTATGGACCTCACCACTGACAA	6	19	1.523	18.988	8.58E-10	-3.64	Hv_Contig_9917	1143	281-306	ACV83781|ribosomal protein S3A *Heliconius melpomene*	3.00E-108
-	Tag_71 CATGGGGCCGTGGCTGCGCTGATGCT	1260	3907	319.749	3904.494	0.00E+00	-3.61	no hit	-	-	-	-
-	Tag_1882 CATGCAGGCGTCCCTCTCGCACACGC	10	31	2.538	30.980	1.06E-14	-3.61	no hit	-	-	-	-
Transport/Trafficking	Tag_2380 CATGTTAGAGAATGTTATAATCTTTG	6	18	1.523	17.988	3.27E-09	-3.56	Hv_Contig_3682	1816	1730-1755	P55277|V-type proton ATPase 16 kDa proteolipid subunit *Heliothis virescens*	4.00E-93
-	Tag_2935 CATGTGGTCAGGCGCACGCGGCGGTA	4	12	1.015	11.992	9.98E-07	-3.56	no hit	-	-	-	-
-	Tag_3022 CATGTGTGGGGAGGAGTGAGCGAGGA	4	12	1.015	11.992	9.98E-07	-3.56	no hit	-	-	-	-
-	Tag_3424 CATGGGGCCGTGACTGCGCTGATGCT	2	6	0.508	5.996	3.41E-04	-3.56	no hit	-	-	-	-
-	Tag_3488 CATGCGGCCCGCGGGCCGCGCGTTGA	2	6	0.508	5.996	3.41E-04	-3.56	no hit	-	-	-	-

-	Tag_3524 CATGATCATTGTTCCTGAGATGGTGG	2	6	0.508	5.996	3.41E-04	-3.56	no hit	-	-	-	-
Primary metabolic process/synthase activity	Tag_3536 CATGTAAACTTTCTACGACCTTCTCA	2	6	0.508	5.996	3.41E-04	-3.56	Hv_Contig_4889	1659	1343-1368	XP_013194701|sialic acid synthase *Amyelois transitella*	0.00E+00
-	Tag_3581 CATGTTATACTTTCAGTGAGTCACCG	2	6	0.508	5.996	3.41E-04	-3.56	no hit	-	-	-	-
Calcium homeostasis	Tag_3593 CATGTGATGCAATAAAAGTGAATTGC	2	6	0.508	5.996	3.41E-04	-3.56	Hv_Contig_29524	536	489-514	XP_004922430|calphotin *Bombyx mori*	6.00E-09
-	Tag_3596 CATGGTTGGACCCATTGAGGAAGTTA	2	6	0.508	5.996	3.41E-04	-3.56	no hit	-	-	-	-
-	Tag_3652 CATGAGGCTGCCAAGGCCCTTGACAC	2	6	0.508	5.996	3.41E-04	-3.56	no hit	-	-	-	-
-	Tag_3667 CATGGAGGAAGATCAGTAGATTTAGG	2	6	0.508	5.996	3.41E-04	-3.56	no hit	-	-	-	-
-	Tag_3689 CATGATTGTGTGAGCCTGTGCGGCAT	2	6	0.508	5.996	3.41E-04	-3.56	no hit	-	-	-	-
-	Tag_3795 CATGCCCGGCTTCCCCCGGCGGCGCG	2	6	0.508	5.996	3.41E-04	-3.56	no hit	-	-	-	-
-	Tag_3837 CATGTCGCCCACAGCGCCGGCGTTGT	2	6	0.508	5.996	3.41E-04	-3.56	no hit	-	-	-	-
-	Tag_3850 CATGCTGCCCCCGACTGCCGCGTGCG	2	6	0.508	5.996	3.41E-04	-3.56	no hit	-	-	-	-
Calcium homeostasis	Tag_3874 CATGTGTCCAAACGGTCACACTTTTA	2	6	0.508	5.996	3.41E-04	-3.56	Hv_Contig_10463	1109	1026-1051	AAV91413|myosin 3 light chain *Lonomia obliqua*	3.00E-103
-	Tag_3907 CATGTTCGCCGCCGCGCCGTTGCAAA	2	6	0.508	5.996	3.41E-04	-3.56	no hit	-	-	-	-
-	Tag_3922 CATGAACCAGTGTATGTAAGGTGTAA	2	6	0.508	5.996	3.41E-04	-3.56	no hit	-	-	-	-
Cytoskeleton	Tag_3937 CATGTAGCGCCAAAATGATTTTGTAC	2	6	0.508	5.996	3.41E-04	-3.56	Hv_Contig_27904	563	471-496	XP_014355707|muscle-specific protein 20 *Papilio machaon*	2.00E-42
-	Tag_3943 CATGGGACGCGGATGAAACCGCTGGC	2	6	0.508	5.996	3.41E-04	-3.56	no hit	-	-	-	-
-	Tag_3972 CATGCGTGCCGCGTCAGACGCGGTCC	2	6	0.508	5.996	3.41E-04	-3.56	no hit	-	-	-	-
Nucleic acid binding	Tag_3976 CATGTTCCCGGCGCTGCAGGCGCGGC	2	6	0.508	5.996	3.41E-04	-3.56	Hv_Contig_12830	980	584-609	XP_013143123|T-box transcription factor TBX1-like *Papilio polytes*	2.00E-42
Translation/Ribosome biogenesis	Tag_4010 CATGAAGTTGAATGCCCACCGCCTAG	2	6	0.508	5.996	3.41E-04	-3.56	Hv_Contig_25370	601	136-161	AEB77710|lysyl-tRNA synthetase *Antheraea pernyi*	1.00E-96
-	Tag_4012 CATGCACGTTCGAGGCGTGCGCCGCA	2	6	0.508	5.996	3.41E-04	-3.56	no hit	-	-	-	-
Nucleic acid binding	Tag_3112 CATGTCCCAGCCGACCAGCGCGTACT	3	9	0.761	8.994	1.80E-05	-3.56	Hv_Contig_1118	2911	308-283	XP_014358694|maternal protein exuperantia *Papilio machaon*	0.00E+00
-	Tag_3175 CATGCTGCCCCCGACCGCCGCGCGCG	3	9	0.761	8.994	1.80E-05	-3.56	no hit	-	-	-	-
Cytoskeleton	Tag_3246 CATGCCGGAGCCGGTGCCGCCGCCCA	3	9	0.761	8.994	1.80E-05	-3.56	Hv_Contig_2860	2078	564-539	XP_013196024|tubulin beta chain-like isoform X1 *Amyelois transitella*	0.00E+00
Transport/Trafficking	Tag_3254 CATGGGGACACGGCGCCGCGGCTCTG	3	9	0.761	8.994	1.80E-05	-3.56	Hv_Contig_885	3131	2484-2509	XP_013187665|potassium/sodium hyperpolarization-activated cyclic nucleotide-gated channel 2 isoform X3 *Amyelois transitella*	0.00E+00
Primary metabolic process/hydrolase activity	Tag_3291 CATGTGCCGCCGACGTTTGATGTTCA	3	9	0.761	8.994	1.80E-05	-3.56	Hv_Contig_322	4191	3919-3944	KOB76772|Beta-galactosidase *Operophtera brumata*	0.00E+00
-	Tag_4071 CATGGCGGCCGCTGCTGACGTCCTTA	1	3	0.254	2.998	7.05E-03	-3.56	no hit	-	-	-	-
-	Tag_4085 CATGCATTTTCTGTGGTACCTTACTG	1	3	0.254	2.998	7.05E-03	-3.56	no hit	-	-	-	-
Unknown	Tag_4098 CATGCTGCGCGCCAAGCACGTCAAGA	1	3	0.254	2.998	7.05E-03	-3.56	Hv_Contig_6170	1475	687-712	XP_004923868|transmembrane protein 205 *Bombyx mori*	1.00E-97
-	Tag_4108 CATGCGAAGCAAAACAGTTATTAAAT	1	3	0.254	2.998	7.05E-03	-3.56	no hit	-	-	-	-
-	Tag_4109 CATGAACTTGATGAAATTATCGAGTA	1	3	0.254	2.998	7.05E-03	-3.56	no hit	-	-	-	-
-	Tag_4112 CATGGAGGCGCTACTCGGCTGAGCCC	1	3	0.254	2.998	7.05E-03	-3.56	no hit	-	-	-	-
-	Tag_4150 CATGGAATGAATCAAGAGAATGTAAG	1	3	0.254	2.998	7.05E-03	-3.56	no hit	-	-	-	-
-	Tag_4153 CATGCTTCAACCAAGCCCGTATGGAG	1	3	0.254	2.998	7.05E-03	-3.56	no hit	-	-	-	-
-	Tag_4180 CATGCGGGCGTCAGTCCAGCTCCCGC	1	3	0.254	2.998	7.05E-03	-3.56	no hit	-	-	-	-
-	Tag_4185 CATGTAGGAGCGTGCGGCGTGCTGTT	1	3	0.254	2.998	7.05E-03	-3.56	no hit	-	-	-	-
-	Tag_4198 CATGTGCGCGTGGAAGCCGCGCTGCG	1	3	0.254	2.998	7.05E-03	-3.56	no hit	-	-	-	-
-	Tag_4206 CATGGTCTACGCCGCCTGCATCGGCC	1	3	0.254	2.998	7.05E-03	-3.56	no hit	-	-	-	-
Primary metabolic process/oxidoreductase activity	Tag_4211 CATGGCGAGAATTTCGGTAAAGCTGT	1	3	0.254	2.998	7.05E-03	-3.56	Hv_Contig_10836	1087	1012-1037	AKD01727|alcohol dehydrogenase 5, partial *Helicoverpa armigera*	8.00E-128
-	Tag_4217 CATGTTGTAATGGTGTTTATGTGAGT	1	3	0.254	2.998	7.05E-03	-3.56	no hit	-	-	-	-
-	Tag_4218 CATGAAATAAAACAAAACAATTATGC	1	3	0.254	2.998	7.05E-03	-3.56	no hit	-	-	-	-
-	Tag_4220 CATGACAATGTAACATCTTAAACTGT	1	3	0.254	2.998	7.05E-03	-3.56	no hit	-	-	-	-
-	Tag_4227 CATGTCGGCGAGCGTACAAGTGTTGC	1	3	0.254	2.998	7.05E-03	-3.56	no hit	-	-	-	-
-	Tag_4228 CATGAGCGACGGCGGCGCGTGCGGCC	1	3	0.254	2.998	7.05E-03	-3.56	no hit	-	-	-	-
-	Tag_4234 CATGTGCGAGGTGGTGGCCGAGCTCG	1	3	0.254	2.998	7.05E-03	-3.56	no hit	-	-	-	-
-	Tag_4236 CATGCTCCGCTAATCTACCAACACTG	1	3	0.254	2.998	7.05E-03	-3.56	no hit	-	-	-	-
-	Tag_4244 CATGCTCCCAAACATCCCGATCTGAG	1	3	0.254	2.998	7.05E-03	-3.56	no hit	-	-	-	-
-	Tag_4279 CATGCCAGGGTGCATCACCTCGATGC	1	3	0.254	2.998	7.05E-03	-3.56	no hit	-	-	-	-
-	Tag_4281 CATGGACTGGGCCGTGGTGCCCCCTC	1	3	0.254	2.998	7.05E-03	-3.56	no hit	-	-	-	-
-	Tag_4285 CATGTGCGCGCTGGTTCCTTGCGGGT	1	3	0.254	2.998	7.05E-03	-3.56	no hit	-	-	-	-
-	Tag_4286 CATGCCCGTACAGAAGCAGACCCGCT	1	3	0.254	2.998	7.05E-03	-3.56	no hit	-	-	-	-
-	Tag_4317 CATGCGTACCGCGTCAGACGCGGTCG	1	3	0.254	2.998	7.05E-03	-3.56	no hit	-	-	-	-
-	Tag_4323 CATGCCGAAGGTCGCGGGTTCGATTC	1	3	0.254	2.998	7.05E-03	-3.56	no hit	-	-	-	-
-	Tag_4327 CATGCCCGACTTCCCCGGCGGCGCGG	1	3	0.254	2.998	7.05E-03	-3.56	no hit	-	-	-	-
-	Tag_4330 CATGTATGCGAGTCATCGAGATAAAG	1	3	0.254	2.998	7.05E-03	-3.56	no hit	-	-	-	-
-	Tag_4359 CATGTACGTGCGCTCCAGCGACCGTT	1	3	0.254	2.998	7.05E-03	-3.56	no hit	-	-	-	-
-	Tag_4412 CATGTTGTAGAGTACTAAGTTGAAAC	1	3	0.254	2.998	7.05E-03	-3.56	no hit	-	-	-	-
-	Tag_4422 CATGTGGGCGGATGCCACGCTCGCAG	1	3	0.254	2.998	7.05E-03	-3.56	no hit	-	-	-	-
-	Tag_4428 CATGGCGCTCAGCCACGGTACCAGGG	1	3	0.254	2.998	7.05E-03	-3.56	no hit	-	-	-	-

-	Tag_4449 CATGGCCGACTATCTGCAGCGCGTAG	1	3	0.254	2.998	7.05E-03	-3.56	no hit	-	-	-	-
Unknown	Tag_4453 CATGCCCTCGGGGCATTGCGCACTGC	1	3	0.254	2.998	7.05E-03	-3.56	Hv_Contig_7980	1286	716-741	XP_004931375|uncharacterized protein LOC101737697 *Bombyx mori*	2.00E-62
-	Tag_4456 CATGGTCCGCGTACCGTCTGGTACGT	1	3	0.254	2.998	7.05E-03	-3.56	no hit	-	-	-	-
-	Tag_4467 CATGGTGGCCACTACAGCCACGGACG	1	3	0.254	2.998	7.05E-03	-3.56	no hit	-	-	-	-
-	Tag_4470 CATGAAGCTCGCGGCCACTTGAACGA	1	3	0.254	2.998	7.05E-03	-3.56	no hit	-	-	-	-
-	Tag_4473 CATGCGTGCCGGCTCGCTGGTCGGCA	1	3	0.254	2.998	7.05E-03	-3.56	no hit	-	-	-	-
-	Tag_4474 CATGGCGTGTCGAGACGAGTCCATCA	1	3	0.254	2.998	7.05E-03	-3.56	no hit	-	-	-	-
-	Tag_4486 CATGGGCTTCGGTCTGTTTGGCTAAA	1	3	0.254	2.998	7.05E-03	-3.56	no hit	-	-	-	-
-	Tag_4487 CATGTGTGCAGTGTGACTGTCTGAGG	1	3	0.254	2.998	7.05E-03	-3.56	no hit	-	-	-	-
-	Tag_4507 CATGTCGCTTATGCCAGCGACAATGA	1	3	0.254	2.998	7.05E-03	-3.56	no hit	-	-	-	-
-	Tag_4514 CATGCCCGGCCAGCAGCTGTTCGCGC	1	3	0.254	2.998	7.05E-03	-3.56	no hit	-	-	-	-
-	Tag_4517 CATGCAAGCGCTGCAAGAGGACCGAG	1	3	0.254	2.998	7.05E-03	-3.56	no hit	-	-	-	-
-	Tag_4518 CATGTCCACTTGCAATCTAACAGATG	1	3	0.254	2.998	7.05E-03	-3.56	no hit	-	-	-	-
-	Tag_4529 CATGGGCCCGCGCCGGCACTAGCGGC	1	3	0.254	2.998	7.05E-03	-3.56	no hit	-	-	-	-
-	Tag_4532 CATGCCGATGGTGGAGAAGACGTCTG	1	3	0.254	2.998	7.05E-03	-3.56	no hit	-	-	-	-
-	Tag_4537 CATGAAGTACTGCGAGTTTGCCGACA	1	3	0.254	2.998	7.05E-03	-3.56	no hit	-	-	-	-
-	Tag_4541 CATGATTGCCCTGCCCCCGCCCCGCA	1	3	0.254	2.998	7.05E-03	-3.56	no hit	-	-	-	-
-	Tag_4552 CATGTGCGCGCTGGCTCCCTGTGGAC	1	3	0.254	2.998	7.05E-03	-3.56	no hit	-	-	-	-
-	Tag_4559 CATGGAAAATATTATTCAGGTATTTG	1	3	0.254	2.998	7.05E-03	-3.56	no hit	-	-	-	-
-	Tag_4571 CATGTCGAGTCTGGCGACCGCGCCCC	1	3	0.254	2.998	7.05E-03	-3.56	no hit	-	-	-	-
-	Tag_4580 CATGCCGTGAACCACGCCGTGGCGGC	1	3	0.254	2.998	7.05E-03	-3.56	no hit	-	-	-	-
-	Tag_4584 CATGCGTATCAGCTCCGTCAGCTTTG	1	3	0.254	2.998	7.05E-03	-3.56	no hit	-	-	-	-
-	Tag_4587 CATGCACCGCCGTGGGGCCGCGCCTG	1	3	0.254	2.998	7.05E-03	-3.56	no hit	-	-	-	-
-	Tag_4633 CATGCCCGTACAGAGGCAGACCCGTG	1	3	0.254	2.998	7.05E-03	-3.56	no hit	-	-	-	-
Signal transduction	Tag_4646 CATGTACAACGCGACCGTATCGACAA	1	3	0.254	2.998	7.05E-03	-3.56	Hv_Contig_9078	1203	723-748	XP_011561443|ras-related protein Rab-8A isoform X2 *Plutella xylostella*	2.00E-139
-	Tag_4654 CATGCTGGTGGCGGCTATGGCCGAGG	1	3	0.254	2.998	7.05E-03	-3.56	no hit	-	-	-	-
-	Tag_4687 CATGGTGCGCCGGCGGCGAGGTGGTG	1	3	0.254	2.998	7.05E-03	-3.56	no hit	-	-	-	-
-	Tag_4708 CATGCCCGTACAGAAGCGGACCCGTG	1	3	0.254	2.998	7.05E-03	-3.56	no hit	-	-	-	-
-	Tag_4735 CATGACTCGCAAACGCCGTAATGGAA	1	3	0.254	2.998	7.05E-03	-3.56	no hit	-	-	-	-
-	Tag_4739 CATGTGCCGCTACGTGCGCGCGCACA	1	3	0.254	2.998	7.05E-03	-3.56	no hit	-	-	-	-
-	Tag_4745 CATGATTCCACACAAGACTGGGCGCG	1	3	0.254	2.998	7.05E-03	-3.56	no hit	-	-	-	-
-	Tag_4756 CATGCCTGTACAGAAGCAGACCCGTG	1	3	0.254	2.998	7.05E-03	-3.56	no hit	-	-	-	-
-	Tag_4777 CATGATTGCCGTCGTGGTTGGAAGCA	1	3	0.254	2.998	7.05E-03	-3.56	no hit	-	-	-	-
-	Tag_4781 CATGGGACACGGGATACGCGTGATGT	1	3	0.254	2.998	7.05E-03	-3.56	no hit	-	-	-	-
-	Tag_4783 CATGGGCGTAAGTGGCTAATGCCCCA	1	3	0.254	2.998	7.05E-03	-3.56	no hit	-	-	-	-
-	Tag_4792 CATGGCGCTGGTCGAGGAGAGAGGGA	1	3	0.254	2.998	7.05E-03	-3.56	no hit	-	-	-	-
-	Tag_4821 CATGCGCCTGTGCGGCGGCCTGGCCA	1	3	0.254	2.998	7.05E-03	-3.56	no hit	-	-	-	-
-	Tag_4836 CATGGGGAAGAGGCTGCGCTGATGGG	1	3	0.254	2.998	7.05E-03	-3.56	no hit	-	-	-	-
-	Tag_4842 CATGCCCGGCTTCCCCGGCCGCGCGC	1	3	0.254	2.998	7.05E-03	-3.56	no hit	-	-	-	-
-	Tag_4843 CATGATCTGAGTGCAAACCGGTGTAA	1	3	0.254	2.998	7.05E-03	-3.56	no hit	-	-	-	-
-	Tag_4904 CATGGAGCCCGTCGTCATCGGCACCC	1	3	0.254	2.998	7.05E-03	-3.56	no hit	-	-	-	-
-	Tag_4934 CATGGCAGTCGCGCTATGCGACCACT	1	3	0.254	2.998	7.05E-03	-3.56	no hit	-	-	-	-
-	Tag_4944 CATGCCGTCGGTGCTGGGCTACGACC	1	3	0.254	2.998	7.05E-03	-3.56	no hit	-	-	-	-
-	Tag_4962 CATGGGGAAGAGACTGCGCTGATGCG	1	3	0.254	2.998	7.05E-03	-3.56	no hit	-	-	-	-
-	Tag_4973 CATGGAGGGCGGCGCGTACGGCGGCC	1	3	0.254	2.998	7.05E-03	-3.56	no hit	-	-	-	-
-	Tag_4983 CATGCCAGGGTGCATCACCTCGATGA	1	3	0.254	2.998	7.05E-03	-3.56	no hit	-	-	-	-
-	Tag_4984 CATGCTGCGCTGTGACGAGCTATGCT	1	3	0.254	2.998	7.05E-03	-3.56	no hit	-	-	-	-
-	Tag_4985 CATGCACCGCGCTACTCGTGAATGCA	1	3	0.254	2.998	7.05E-03	-3.56	no hit	-	-	-	-
-	Tag_5016 CATGACCGGACCGAGTCGCGACATAG	1	3	0.254	2.998	7.05E-03	-3.56	no hit	-	-	-	-
-	Tag_5028 CATGATTGTGTGAGCCCGTGCGGCCG	1	3	0.254	2.998	7.05E-03	-3.56	no hit	-	-	-	-
-	Tag_5034 CATGATTGTGTGAGCCTGTGCGGTCG	1	3	0.254	2.998	7.05E-03	-3.56	no hit	-	-	-	-
-	Tag_5040 CATGCACTCGGCCGAGCGGCCGGAAA	1	3	0.254	2.998	7.05E-03	-3.56	no hit	-	-	-	-
-	Tag_5060 CATGAGCAAGCAGCAGCCCACGCCGG	1	3	0.254	2.998	7.05E-03	-3.56	no hit	-	-	-	-
-	Tag_5065 CATGCGGGCGTGAGGCCTCTCACGTG	1	3	0.254	2.998	7.05E-03	-3.56	no hit	-	-	-	-
-	Tag_5147 CATGAGGCAGACGGTCGCCGTGGGTA	1	3	0.254	2.998	7.05E-03	-3.56	no hit	-	-	-	-
-	Tag_5150 CATGTCCGTGCGCTCTAGCGACCGTG	1	3	0.254	2.998	7.05E-03	-3.56	no hit	-	-	-	-
-	Tag_5151 CATGCCACTACTTCCCGCACCCGGAC	1	3	0.254	2.998	7.05E-03	-3.56	no hit	-	-	-	-
-	Tag_5155 CATGGGCTTCGGTCTGTTTGACTAAC	1	3	0.254	2.998	7.05E-03	-3.56	no hit	-	-	-	-

-	Tag_5166 CATGAGGCAGGCGGTCGCTGTGGGTA	1	3	0.254	2.998	7.05E-03	-3.56	no hit	-	-	-	-
-	Tag_5171 CATGTCGCTCTCGCGCCATTGACCGA	1	3	0.254	2.998	7.05E-03	-3.56	no hit	-	-	-	-
-	Tag_5191 CATGCTGGAGTCCGTCGGCAAGACTG	1	3	0.254	2.998	7.05E-03	-3.56	no hit	-	-	-	-
-	Tag_5197 CATGGAAATTTATTGTAAATGACAAG	1	3	0.254	2.998	7.05E-03	-3.56	no hit	-	-	-	-
Primary metabolic process/transferase activity	Tag_5198 CATGTTATTCCTTTTTGTACTGCGTT	1	3	0.254	2.998	7.05E-03	-3.56	Hv_Contig_190	4750	4623-4648	XP_013188102|glycerol-3-phosphate acyltransferase 1, mitochondrial isoform X1 *Amyelois transitella*	0.00E+00
-	Tag_2253 CATGTTGTGTGCTGGTGTCTTAGACG	7	21	1.776	20.987	1.91E-10	-3.56	no hit	-	-	-	-
-	Tag_2291 CATGGGCGTAAGTGGCTAATGCCCCG	7	21	1.776	20.987	1.91E-10	-3.56	no hit	-	-	-	-
-	Tag_2338 CATGCTGCCCCCGACTGCCGCGCGTA	7	21	1.776	20.987	1.91E-10	-3.56	no hit	-	-	-	-
Primary metabolic process/hydrolase activity	Tag_522 CATGGGTGGTGCACCTCCGCGTTGCC	102	303	25.884	302.806	1.67E-123	-3.55	Hv_Contig_10076	1132	193-218	ABR88240|serine protease SP1 *Heliothis virescens*	5.00E-146
-	Tag_1262 CATGGGACTCACCGAGTACGACGCCC	22	63	5.583	62.960	5.24E-27	-3.50	no hit	-	-	-	-
-	Tag_2347 CATGCTCCCAAACATCCCGATCTGGG	6	17	1.523	16.989	1.23E-08	-3.48	no hit	-	-	-	-
-	Tag_2416 CATGCCCGTACAGAAGCAGACCCGGA	6	17	1.523	16.989	1.23E-08	-3.48	no hit	-	-	-	-
-	Tag_2462 CATGCCCACACTGACGGTTTCTGCGC	6	17	1.523	16.989	1.23E-08	-3.48	no hit	-	-	-	-
-	Tag_1853 CATGTGCGAGTTGCTGCCGGGCGAGC	11	31	2.791	30.980	3.33E-14	-3.47	no hit	-	-	-	-
-	Tag_2730 CATGGGTACGAGGTGGAAGGCCAATT	4	11	1.015	10.993	3.79E-06	-3.44	no hit	-	-	-	-
Cytoskeleton	Tag_2860 CATGGACGCGCCCATCGACTCGCTGT	4	11	1.015	10.993	3.79E-06	-3.44	Hv_Contig_6559	1428	746-771	XP_004926949|E3 ubiquitin-protein ligase MYLIP *Bombyx mori*	8.00E-94
-	Tag_2938 CATGAAATAAAACAAAACAATTATGG	4	11	1.015	10.993	3.79E-06	-3.44	no hit	-	-	-	-
-	Tag_2957 CATGGGGCTCACCGAGTACGACGCCG	4	11	1.015	10.993	3.79E-06	-3.44	no hit	-	-	-	-
Chitin-binding	Tag_2963 CATGGTAGCGAGCGTGCGCCCCATCT	4	11	1.015	10.993	3.79E-06	-3.44	Hv_Contig_8337	1256	519-544	ACB54954|insect intestinal mucin 3 *Helicoverpa armigera*	2.00E-133
-	Tag_2985 CATGAAGATTGGCCGCATTGAGGAGG	4	11	1.015	10.993	3.79E-06	-3.44	no hit	-	-	-	-
-	Tag_2424 CATGATCTGTGCTGCCCAACTCGGGA	6	16	1.523	15.990	4.59E-08	-3.39	no hit	-	-	-	-
-	Tag_3091 CATGCTGCAGCACGCGCTGGGCGTTG	3	8	0.761	7.995	6.90E-05	-3.39	no hit	-	-	-	-
-	Tag_3122 CATGGGACCCACCGAGTACGACGCCG	3	8	0.761	7.995	6.90E-05	-3.39	no hit	-	-	-	-
-	Tag_3237 CATGGACCTTACCAGGCTAGGACCTT	3	8	0.761	7.995	6.90E-05	-3.39	no hit	-	-	-	-
Unknown	Tag_3333 CATGGACTGAGGAGCCAGCTAAGGCT	3	8	0.761	7.995	6.90E-05	-3.39	Hv_Contig_17093	809	495-520	EHJ69021|16 kDa salivary protein *Danaus plexippus*	4.00E-94
Protein kinases	Tag_3403 CATGTGTATAATGAACTTAAAGGGGG	3	8	0.761	7.995	6.90E-05	-3.39	Hv_Contig_11242	1063	881-906	XP_013199995|serine-threonine kinase receptor-associated protein isoform X1 *Amyelois transitella*	3.00E-177
-	Tag_371 CATGCTGCCCCCGACTGCCGCGCGCA	164	435	41.618	434.721	8.68E-168	-3.38	no hit	-	-	-	-
Nucleic acid binding	Tag_698 CATGGCGCTGCAGGTGGACGTGTCCG	63	167	15.987	166.893	7.73E-66	-3.38	Hv_Contig_1683	2531	2089-2114	AAC60499|Transcription factor ecdysone-inducible E75 isoform X3 (E75 B) *Manduca sexta*	0.00E+00
Unknown	Tag_2585 CATGTCGGTGCCGTCGTTCGTGGCGG	5	13	1.269	12.992	7.98E-07	-3.36	Hv_Contig_2402	2230	1726-1751	XP_013194181|uncharacterized protein LOC106137813 *Amyelois transitella*	0.00E+00
-	Tag_2675 CATGTGCGCACGCGACGCGTGGCTCG	5	13	1.269	12.992	7.98E-07	-3.36	no hit	-	-	-	-
Primary metabolic process/transferase activity	Tag_1315 CATGGGTCCCGAGTGTATGGGGTGGG	20	52	5.075	51.967	8.95E-22	-3.36	Hv_Contig_7667	1317	1097-1122	XP_014364198|beta-1,3-galactosyltransferase 4-like, partial *Papilio machaon*	4.00E-159
-	Tag_1963 CATGGAGGCTATCGCCGTCGCCCTCA	10	26	2.538	25.983	7.48E-12	-3.36	no hit	-	-	-	-
-	Tag_2206 CATGCGCCGCAATCGCCAACAGTCTG	7	18	1.776	17.988	9.82E-09	-3.34	no hit	-	-	-	-
mRNA processing/splicing	Tag_2017 CATGGAGGCCGAAACGACGGTAGAGG	9	23	2.284	22.985	1.25E-10	-3.33	Hv_Contig_743	3306	1496-1521	XP_013184329|H/ACA ribonucleoprotein complex subunit 4 *Amyelois transitella*	0.00E+00
-	Tag_703 CATGCTGCCCCCGACTGCCGCGCGTG	62	158	15.734	157.899	2.58E-61	-3.33	no hit	-	-	-	-
-	Tag_1706 CATGCGGCTCGTGCGGCGGGCGCTAT	13	33	3.299	32.979	2.15E-14	-3.32	no hit	-	-	-	-
-	Tag_1194 CATGCGTGCCGCGTCAGACGCGGTCA	24	60	6.090	59.962	2.14E-24	-3.30	no hit	-	-	-	-
Calcium homeostasis	Tag_3425 CATGTTTCCTGCACCTCGTGTTGGAC	2	5	0.508	4.997	1.32E-03	-3.30	Hv_Contig_6408	1445	1196-1221	ACY69027|mutant cadherin *Helicoverpa armigera*	2.00E-19
-	Tag_3447 CATGATTCCACACAAGACTGAGCGCC	2	5	0.508	4.997	1.32E-03	-3.30	no hit	-	-	-	-
-	Tag_3456 CATGAGTTTCATTCAACAGTACCTGG	2	5	0.508	4.997	1.32E-03	-3.30	no hit	-	-	-	-
-	Tag_3479 CATGGTAATGATTATTTTCAAAGATG	2	5	0.508	4.997	1.32E-03	-3.30	no hit	-	-	-	-
-	Tag_3512 CATGCATGTTAATCAGCAAACTAGTA	2	5	0.508	4.997	1.32E-03	-3.30	no hit	-	-	-	-
Translation/Ribosome biogenesis	Tag_3561 CATGTAATATATAGGGATACGATCGG	2	5	0.508	4.997	1.32E-03	-3.30	Hv_Contig_3502	1922	1788-1813	XP_013196635|eukaryotic translation initiation factor 5 *Amyelois transitella*	2.00E-30
-	Tag_3567 CATGGGGCCGTGGCTACGCTGATGCT	2	5	0.508	4.997	1.32E-03	-3.30	no hit	-	-	-	-
-	Tag_3585 CATGTTGAGTCTGGCGACCGCGCTTA	2	5	0.508	4.997	1.32E-03	-3.30	no hit	-	-	-	-
-	Tag_3598 CATGTCTCCCGTATATTGTAAGCGAG	2	5	0.508	4.997	1.32E-03	-3.30	no hit	-	-	-	-
Primary metabolic process/oxidoreductase activity	Tag_3606 CATGTTCTTACTTTGTGAACCCTATT	2	5	0.508	4.997	1.32E-03	-3.30	Hv_Contig_3857	1848	1788-1813	XP_013133544|selT-like protein *Papilio polytes*	7.00E-99
-	Tag_3617 CATGCGCGATTCCACTCGGCCGGTTT	2	5	0.508	4.997	1.32E-03	-3.30	no hit	-	-	-	-
-	Tag_3634 CATGGCTCGCAGCCGCCCCCTGGTGA	2	5	0.508	4.997	1.32E-03	-3.30	no hit	-	-	-	-
-	Tag_3668 CATGATGCCGCGCCGCGCCGCGCCGC	2	5	0.508	4.997	1.32E-03	-3.30	no hit	-	-	-	-
-	Tag_3675 CATGATTGAAATATGCATCTATTTGT	2	5	0.508	4.997	1.32E-03	-3.30	no hit	-	-	-	-
Unknown	Tag_3681 CATGCAAAAATCTGAAATAAACTTGA	2	5	0.508	4.997	1.32E-03	-3.30	Hv_Contig_1114	2915	2794-2819	XP_012550425|protein LSM14 homolog B-A isoform X1 *Bombyx mori*	6.00E-113
Primary metabolic process/oxidoreductase activity	Tag_3697 CATGTTGGAGACCTCGGCAACATTGA	2	5	0.508	4.997	1.32E-03	-3.30	Hv_Contig_11183	1067	396-421	AFC98366|Cu/Zn superoxide dismutase *Helicoverpa armigera*	9.00E-90
-	Tag_3714 CATGATCGTCCGAGACACGCCCCTCG	2	5	0.508	4.997	1.32E-03	-3.30	no hit	-	-	-	-
-	Tag_3736 CATGGACGAGCTCCCGGTGCCGCGGG	2	5	0.508	4.997	1.32E-03	-3.30	no hit	-	-	-	-
-	Tag_3747 CATGGGGCCGTGGCTGCGTTGATGCT	2	5	0.508	4.997	1.32E-03	-3.30	no hit	-	-	-	-
-	Tag_3784 CATGTGGTCAGGCGCACGCGGCGGCG	2	5	0.508	4.997	1.32E-03	-3.30	no hit	-	-	-	-
Translation/Ribosome biogenesis	Tag_3825 CATGAACAGAAGATACGGATGGGATG	2	5	0.508	4.997	1.32E-03	-3.30	Hv_Contig_27612	569	71-46	KPJ11126|39S ribosomal protein L38, mitochondrial *Papilio machaon*	4.00E-90

-	Tag_3829 CATGGATGCGTTGCAGCGCTCGCGTT	2	5	0.508	4.997	1.32E-03	-3.30	no hit	-	-	-	-
-	Tag_3831 CATGGCTGTGAGGATCGTGAAGCAAG	2	5	0.508	4.997	1.32E-03	-3.30	no hit	-	-	-	-
-	Tag_3833 CATGGGACTCACCAAGTACGACGCCG	2	5	0.508	4.997	1.32E-03	-3.30	no hit	-	-	-	-
-	Tag_3882 CATGGGGCTGTGGCTGCGCTGATGCT	2	5	0.508	4.997	1.32E-03	-3.30	no hit	-	-	-	-
-	Tag_3887 CATGACACGTCGCGGCGCTCTGTTGC	2	5	0.508	4.997	1.32E-03	-3.30	no hit	-	-	-	-
Protein folding/Recycling	Tag_3942 CATGCTGGACCTCAAGATGAACCCCG	2	5	0.508	4.997	1.32E-03	-3.30	Hv_Contig_15909	850	637-662	NP_001073120|cytochrome c oxidase polypeptide IV *Bombyx mori*	6.00E-95
-	Tag_3944 CATGGGCTTCGGTCTGTTTGACTAAT	2	5	0.508	4.997	1.32E-03	-3.30	no hit	-	-	-	-
Translation/Ribosome biogenesis	Tag_3952 CATGCGAAAGCGCCAACTAGTTTTAT	2	5	0.508	4.997	1.32E-03	-3.30	Hv_Contig_29740	532	322-347	XP_011560948|transcription elongation factor 1 homolog *Plutella xylostella*	2.00E-40
Unknown	Tag_3987 CATGCGCCAAGTCCAGGCGAAGCCCT	2	5	0.508	4.997	1.32E-03	-3.30	Hv_Contig_19298	744	323-298	XP_004922642.2|uncharacterized protein LOC101738833 *Bombyx mori*	4.00E-41
Signal transduction	Tag_4026 CATGTGCGACCTGGCGGCGCCGCTGC	2	5	0.508	4.997	1.32E-03	-3.30	Hv_Contig_481	3790	3214-3239	XP_014363106|small G protein signaling modulator 2-like *Papilio machaon*	0.00E+00
-	Tag_1898 CATGGGCCCGCGCCGGTACTAGCGGA	10	25	2.538	24.984	2.71E-11	-3.30	no hit	-	-	-	-
-	Tag_1906 CATGTCTGTCTGTCTGTCTGTCTGTC	10	25	2.538	24.984	2.71E-11	-3.30	no hit	-	-	-	-
-	Tag_2123 CATGCTGTTGGCTGCGGTAGCTACCG	8	20	2.030	19.987	2.10E-09	-3.30	no hit	-	-	-	-
-	Tag_2352 CATGTCATTGCGGCCGTTCACGAACC	6	15	1.523	14.990	1.68E-07	-3.30	no hit	-	-	-	-
-	Tag_2760 CATGGCCCTCGGCATCCGCTCACCCG	4	10	1.015	9.994	1.41E-05	-3.30	no hit	-	-	-	-
-	Tag_2974 CATGCGGTGCGCTTGCGCACGTCGCG	4	10	1.015	9.994	1.41E-05	-3.30	no hit	-	-	-	-
-	Tag_2994 CATGGCCGACTATCCGCAGCGCGTGA	4	10	1.015	9.994	1.41E-05	-3.30	no hit	-	-	-	-
-	Tag_1578 CATGGTTGGTGCGCGCGGGGGGCTTT	15	37	3.807	36.976	1.03E-15	-3.28	no hit	-	-	-	-
-	Tag_2070 CATGCGCCCGCAGGTGCACACCTACC	9	22	2.284	21.986	4.51E-10	-3.27	no hit	-	-	-	-
-	Tag_449 CATGTGCGCGCTGGCTCCCTGTGGGG	124	300	31.467	299.808	1.45E-111	-3.25	no hit	-	-	-	-
Protein kinases	Tag_2625 CATGGCGAACCACTCGTCCTGGCTGA	5	12	1.269	11.992	2.92E-06	-3.24	Hv_Contig_13089	966	174-149	AKN89772|juvenile hormone diol kinase *Spodoptera litura*	1.00E-60
-	Tag_1957 CATGCCCAGCGTCAGCGCCGCGCCCA	10	24	2.538	23.985	9.69E-11	-3.24	no hit	-	-	-	-
-	Tag_2166 CATGATTGTGTGAGCCTGTGCGGCTG	8	19	2.030	18.988	7.56E-09	-3.23	no hit	-	-	-	-
-	Tag_1246 CATGTGGTGGACTCCGTTGCTGAGCA	22	52	5.583	51.967	6.91E-21	-3.22	no hit	-	-	-	-
-	Tag_1757 CATGGAGGGCGGCGCGTACGGCGGCA	12	28	3.045	27.982	4.48E-12	-3.20	no hit	-	-	-	-
-	Tag_1557 CATGTTGTGCTCTGGCTGGCCCACTA	15	35	3.807	34.978	1.28E-14	-3.20	no hit	-	-	-	-
-	Tag_1561 CATGCTGTCGGTGCTGGGCTACGACA	15	35	3.807	34.978	1.28E-14	-3.20	no hit	-	-	-	-
-	Tag_3153 CATGCCCGTACAGAAGCAGACCCGGT	3	7	0.761	6.996	2.58E-04	-3.20	no hit	-	-	-	-
-	Tag_3164 CATGCTGCCCCCGACTACCGCGCGCG	3	7	0.761	6.996	2.58E-04	-3.20	no hit	-	-	-	-
-	Tag_3189 CATGTTCTACGCGCTGTCGTTCCTGG	3	7	0.761	6.996	2.58E-04	-3.20	no hit	-	-	-	-
-	Tag_3197 CATGATTGTGTGAGCCTGTGCGGCGA	3	7	0.761	6.996	2.58E-04	-3.20	no hit	-	-	-	-
-	Tag_3289 CATGCCCGTACAGAAGCAGACCCGGC	3	7	0.761	6.996	2.58E-04	-3.20	no hit	-	-	-	-
-	Tag_3398 CATGCGATCACGTGGCCGACCTTCCA	3	7	0.761	6.996	2.58E-04	-3.20	no hit	-	-	-	-
-	Tag_2340 CATGGGTTGGCGCGGTGGCTTCAACC	6	14	1.523	13.991	6.08E-07	-3.20	no hit	-	-	-	-
-	Tag_941 CATGGCCGACTATCCGCAGCGCGTAA	35	81	8.882	80.948	4.42E-31	-3.19	no hit	-	-	-	-
-	Tag_1219 CATGCCCGTACAGAAGCAGACCCGCG	23	53	5.837	52.966	5.27E-21	-3.18	no hit	-	-	-	-
-	Tag_837 CATGCCCGTACAGAAGCAGACCCGGG	44	101	11.166	100.935	4.42E-38	-3.18	no hit	-	-	-	-
Protein kinases	Tag_45 CATGGGACTCACCGAGTACGACGCCG	2223	5004	564.128	5000.791	0.00E+00	-3.15	Hv_Contig_8820	1220	854-879	ABU98622|arginine kinase *Helicoverpa armigera*	1.00E-76
-	Tag_1732 CATGGACAGACGCTAAGCTGACCGAG	12	27	3.045	26.983	1.57E-11	-3.15	no hit	-	-	-	-
-	Tag_2781 CATGAGAGGAGGCCTGTGCCCAGCGG	4	9	1.015	8.994	5.16E-05	-3.15	no hit	-	-	-	-
-	Tag_2790 CATGCCCGGCTTCCCCGGCGGCGCCG	4	9	1.015	8.994	5.16E-05	-3.15	no hit	-	-	-	-
-	Tag_2808 CATGACGGCTCCTTGTAGCTCGTGTA	4	9	1.015	8.994	5.16E-05	-3.15	no hit	-	-	-	-
Cytoskeleton	Tag_2884 CATGCAGTGCACGCCGACCTTGCCGA	4	9	1.015	8.994	5.16E-05	-3.15	Hv_Contig_6139	1479	395-370	AFP36379|profilin, partial *Spodoptera frugiperda*	5.00E-80
-	Tag_2917 CATGGGGCCGTGGCTGCGCTGACGCT	4	9	1.015	8.994	5.16E-05	-3.15	no hit	-	-	-	-
-	Tag_2960 CATGGGGCCGTGGCTGCGCTGGTGCT	4	9	1.015	8.994	5.16E-05	-3.15	no hit	-	-	-	-
-	Tag_3003 CATGGGCCCGCGCCGGTACTAGCGGG	4	9	1.015	8.994	5.16E-05	-3.15	no hit	-	-	-	-
-	Tag_1302 CATGATTGTGTGAGCCTGTGCGGCTA	21	47	5.329	46.970	1.34E-18	-3.14	no hit	-	-	-	-
-	Tag_1022 CATGCTGCCCCCGACTGCCGCGCGCC	30	67	7.613	66.957	1.25E-25	-3.14	no hit	-	-	-	-
Signal transduction	Tag_2619 CATGCTCTGGGACCTGAATGATGGCA	5	11	1.269	10.993	1.05E-05	-3.11	Hv_Contig_9229	1192	846-871	AEB26318|receptor for activated protein kinase C *Helicoverpa armigera*	0.00E+00
-	Tag_2673 CATGGGACTCGCCGAGTACGACGCCG	5	11	1.269	10.993	1.05E-05	-3.11	no hit	-	-	-	-
-	Tag_2702 CATGAAGCTCGGCGCGGGTCTCCCAA	5	11	1.269	10.993	1.05E-05	-3.11	no hit	-	-	-	-
-	Tag_1960 CATGATCTGAGTTCAAACCGGTGTTG	10	22	2.538	21.986	1.20E-09	-3.11	no hit	-	-	-	-
-	Tag_2442 CATGAGCTCAGACGTCGCGGCCTCGA	6	13	1.523	12.992	2.16E-06	-3.09	no hit	-	-	-	-
Transport/Trafficking	Tag_2307 CATGGATGCCGGCTGGAGATATTGGA	7	15	1.776	14.990	4.48E-07	-3.08	Hv_Contig_571	3615	3571-3596	XP_014357019|exocyst complex component 1 *Papilio machaon*	5.00E-117
Unknown	Tag_1159 CATGGCCGACGCTACGCTGCTGCCCG	25	53	6.344	52.966	3.48E-20	-3.06	Hv_Contig_1395	2709	1684-1709	EHJ70796|hypothetical protein KGM_19480 *Danaus plexippus*	0.00E+00
Primary metabolic process/oxidoreductase activity	Tag_1990 CATGAATGAAACCAAAATATATTAAA	9	19	2.284	18.988	1.97E-08	-3.06	Hv_Contig_30226	523	379-404	ALF04080|NADH dehydrogenase subunit 3 (mitochondrion) *Helicoverpa assulta*	1.00E-36
-	Tag_2061 CATGATCGTCCGAGACACGCCCCCCC	9	19	2.284	18.988	1.97E-08	-3.06	no hit	-	-	-	-
Primary metabolic process/oxidoreductase activity	Tag_1869 CATGTTGCTTCGCTGTGACCGCCGTT	11	23	2.791	22.985	8.80E-10	-3.04	Hv_Contig_7387	1343	1075-1100	NP_001106230|cytochrome c1 *Bombyx mori*	8.00E-175

Primary metabolic process/hydrolase activity	Tag_298 CATGCACGGCAGCTGGACCCCTAGCT	212	440	53.799	439.718	4.41E-150	-3.03	Hv_Contig_26040	591	404-429	AFM28262|chymotrypsin, partial *Heliothis virescens*	3.00E-121
Unknown	Tag_495 CATGCGCCGCAATCGCCAACAGTCCG	109	224	27.661	223.856	3.74E-77	-3.02	Hv_Contig_3049	2027	580-605	XP_014362375|uncharacterized protein LOC106713977 *Papilio machaon*	3.00E-168
Transport/Trafficking	Tag_2351 CATGTCGCTTATGCCAGCGACAATGG	6	12	1.523	11.992	7.52E-06	-2.98	Hv_Contig_19780	729	549-574	XP_004925267|synaptic vesicle glycoprotein 2A-like *Bombyx mori*	2.00E-47
-	Tag_2382 CATGATCTGAGTTCAAACCGGTGCAG	6	12	1.523	11.992	7.52E-06	-2.98	no hit	-	-	-	-
-	Tag_2406 CATGCCTGCTGCAAAAAACTGCTGGG	6	12	1.523	11.992	7.52E-06	-2.98	no hit	-	-	-	-
-	Tag_2552 CATGCGCTGTTGAGTTTGCCGCCTGG	5	10	1.269	9.994	3.69E-05	-2.98	no hit	-	-	-	-
-	Tag_2621 CATGGCGGCCGCCTTCCGCAACCTGA	5	10	1.269	9.994	3.69E-05	-2.98	no hit	-	-	-	-
-	Tag_2639 CATGGTCCGCGAACGAAGTCGCTGGC	5	10	1.269	9.994	3.69E-05	-2.98	no hit	-	-	-	-
Unknown	Tag_2687 CATGGGACCACCTGTGAAGTATACCC	5	10	1.269	9.994	3.69E-05	-2.98	Hv_Contig_2062	2361	1986-2011	EHJ68796|hypothetical protein KGM_04834 *Danaus plexippus*	5.00E-139
-	Tag_2699 CATGCTGGACCTGGCGCAGGTGGCTA	5	10	1.269	9.994	3.69E-05	-2.98	no hit	-	-	-	-
-	Tag_2071 CATGCTGCGGCCCCTGCGGCGGGTGC	9	18	2.284	17.988	6.74E-08	-2.98	no hit	-	-	-	-
-	Tag_4045 CATGCCGGGCGCGGGCCGCGCGGCGT	1	2	0.254	1.999	2.82E-02	-2.98	no hit	-	-	-	-
-	Tag_4049 CATGCGCGCGAGCGAGACGCGCGATC	1	2	0.254	1.999	2.82E-02	-2.98	no hit	-	-	-	-
-	Tag_4055 CATGAACCAGTGTATGTAAGGCGTAC	1	2	0.254	1.999	2.82E-02	-2.98	no hit	-	-	-	-
-	Tag_4057 CATGTTGGTGCTGTTGATTGCCGCAC	1	2	0.254	1.999	2.82E-02	-2.98	no hit	-	-	-	-
-	Tag_4059 CATGCCGTACGCGCGCACGTTGGAGG	1	2	0.254	1.999	2.82E-02	-2.98	no hit	-	-	-	-
-	Tag_4062 CATGCTCACCGCCGGCGCTGAGGAGG	1	2	0.254	1.999	2.82E-02	-2.98	no hit	-	-	-	-
-	Tag_4070 CATGCTGCTCCGGCTTGCTCGGATCA	1	2	0.254	1.999	2.82E-02	-2.98	no hit	-	-	-	-
-	Tag_4073 CATGGGGAGGTTCTGCTCGGGACTGG	1	2	0.254	1.999	2.82E-02	-2.98	no hit	-	-	-	-
-	Tag_4076 CATGAATCCAATAAGCAGATCGCTGC	1	2	0.254	1.999	2.82E-02	-2.98	no hit	-	-	-	-
-	Tag_4078 CATGTCAGTTTCTGAAAAAAAAAAAA	1	2	0.254	1.999	2.82E-02	-2.98	no hit	-	-	-	-
-	Tag_4080 CATGATTGTGTGAGCCTGTGCAGCCA	1	2	0.254	1.999	2.82E-02	-2.98	no hit	-	-	-	-
-	Tag_4082 CATGGAGCGGCCGCGAGGGAATCTCC	1	2	0.254	1.999	2.82E-02	-2.98	no hit	-	-	-	-
-	Tag_4086 CATGATCTGAGTTCAAACCGGTGCGA	1	2	0.254	1.999	2.82E-02	-2.98	no hit	-	-	-	-
-	Tag_4093 CATGATCTGAGTTCAAACCGGTGTTT	1	2	0.254	1.999	2.82E-02	-2.98	no hit	-	-	-	-
-	Tag_4099 CATGACCGACTATCCGCAGCGCGTAG	1	2	0.254	1.999	2.82E-02	-2.98	no hit	-	-	-	-
-	Tag_4106 CATGAGGCAGTCGGTCGCTGTGGGTA	1	2	0.254	1.999	2.82E-02	-2.98	no hit	-	-	-	-
-	Tag_4107 CATGCCCGGCTTCCCCGGCGGCGAGA	1	2	0.254	1.999	2.82E-02	-2.98	no hit	-	-	-	-
Primary metabolic process/hydrolase activity	Tag_4121 CATGAGGGCGGTGCGCACGCGCCGGT	1	2	0.254	1.999	2.82E-02	-2.98	Hv_Contig_1736	2502	2156-2181	XP_004922014|N-sulphoglucosamine sulphohydrolase *Bombyx mori*	0.00E+00
-	Tag_4132 CATGTTGCCCCCGACTGCCGCGCGCG	1	2	0.254	1.999	2.82E-02	-2.98	no hit	-	-	-	-
-	Tag_4138 CATGTTAGGTCGTTCAAGACTGTCTG	1	2	0.254	1.999	2.82E-02	-2.98	no hit	-	-	-	-
-	Tag_4147 CATGCGCATCAGCTCCGTCAGCTTAG	1	2	0.254	1.999	2.82E-02	-2.98	no hit	-	-	-	-
-	Tag_4175 CATGTTCCCGATGCCGGTGGGAGACC	1	2	0.254	1.999	2.82E-02	-2.98	no hit	-	-	-	-
-	Tag_4184 CATGTTGAGTCCGGCGACCGCGCCAC	1	2	0.254	1.999	2.82E-02	-2.98	no hit	-	-	-	-
-	Tag_4187 CATGCCGCAGGCGCTGGCCGCCACGG	1	2	0.254	1.999	2.82E-02	-2.98	no hit	-	-	-	-
-	Tag_4189 CATGATCACTGGTGCACACCTACCCG	1	2	0.254	1.999	2.82E-02	-2.98	no hit	-	-	-	-
-	Tag_4192 CATGCTGCAGCACGCGCTGGGCGTCC	1	2	0.254	1.999	2.82E-02	-2.98	no hit	-	-	-	-
-	Tag_4193 CATGGAAATATCTCTGTCCGATTAGG	1	2	0.254	1.999	2.82E-02	-2.98	no hit	-	-	-	-
-	Tag_4194 CATGATTGTGTGAGCCAGTGCGGCCA	1	2	0.254	1.999	2.82E-02	-2.98	no hit	-	-	-	-
-	Tag_4208 CATGAATAGGGGGCGAGGTGGGGTAG	1	2	0.254	1.999	2.82E-02	-2.98	no hit	-	-	-	-
Primary metabolic process/isomerase activity	Tag_4215 CATGTAGAATGCAGCAAAAAATGGGC	1	2	0.254	1.999	2.82E-02	-2.98	Hv_Contig_2999	2039	980-1005	NP_001299654|phosphoglycerate mutase 1 *Papilio xuthus*	1.00E-169
Translation/Ribosome biogenesis	Tag_4219 CATGAGACGCGGCTGCGCGGTCAGCA	1	2	0.254	1.999	2.82E-02	-2.98	Hv_Contig_1033	2992	2305-2280	AAL83698|translation elongation factor 2 *Spodoptera exigua*	0.00E+00
-	Tag_4221 CATGCTGCAGCACGTCGCGCGCACGC	1	2	0.254	1.999	2.82E-02	-2.98	no hit	-	-	-	-
-	Tag_4226 CATGGCCGACTATCCGCAGCGCGCAG	1	2	0.254	1.999	2.82E-02	-2.98	no hit	-	-	-	-
-	Tag_4230 CATGAGGACCAACAACTTCAAGGACA	1	2	0.254	1.999	2.82E-02	-2.98	no hit	-	-	-	-
-	Tag_4231 CATGTTGCCGGAGACGAAGGGCAAGG	1	2	0.254	1.999	2.82E-02	-2.98	no hit	-	-	-	-
-	Tag_4241 CATGGGGAGGTTCTGCTCAGGGCTGA	1	2	0.254	1.999	2.82E-02	-2.98	no hit	-	-	-	-
-	Tag_4246 CATGTTGAGTCTGGCGACTGCGCCGC	1	2	0.254	1.999	2.82E-02	-2.98	no hit	-	-	-	-
mRNA processing/splicing	Tag_4250 CATGACGTGGTTTTATGAGGATGGGA	1	2	0.254	1.999	2.82E-02	-2.98	Hv_Contig_226	4560	4128-4153	XP_012550563|LOW QUALITY PROTEIN: protein split ends *Bombyx mori*	1.00E-89
-	Tag_4256 CATGTTGTGCTCGGGCTGGCCCAACA	1	2	0.254	1.999	2.82E-02	-2.98	no hit	-	-	-	-
Translation/Ribosome biogenesis	Tag_4262 CATGTTACGTCTGTGGCTAGAAAACT	1	2	0.254	1.999	2.82E-02	-2.98	Hv_Contig_7505	1333	1052-1077	AAK94897|mRNA cap-binding protein eIF4E *Spodoptera frugiperda*	8.00E-149
-	Tag_4265 CATGCTCACCGCCAGCGCTGTGGAGG	1	2	0.254	1.999	2.82E-02	-2.98	no hit	-	-	-	-
-	Tag_4277 CATGTCTGTCATCAATTGATGAATCG	1	2	0.254	1.999	2.82E-02	-2.98	no hit	-	-	-	-
-	Tag_4288 CATGACCTTGGAGGCTGCCAGCGCAC	1	2	0.254	1.999	2.82E-02	-2.98	no hit	-	-	-	-
-	Tag_4292 CATGCGCCGCCACCACACCACGCGCA	1	2	0.254	1.999	2.82E-02	-2.98	no hit	-	-	-	-
-	Tag_4296 CATGAGCCCCCCCCTGCCCGACACCT	1	2	0.254	1.999	2.82E-02	-2.98	no hit	-	-	-	-
-	Tag_4298 CATGCTGTTGGCTGCTACAGCCACCG	1	2	0.254	1.999	2.82E-02	-2.98	no hit	-	-	-	-
-	Tag_4302 CATGCAGTTGCTGCGAGACAACCTCG	1	2	0.254	1.999	2.82E-02	-2.98	no hit	-	-	-	-

-	Tag_4304 CATGATTGCCGTCGTGGCCGGAAGCA	1	2	0.254	1.999	2.82E-02	-2.98	no hit	-	-	-	-
-	Tag_4307 CATGGCTCGCAGCCACCCCCTGGTTC	1	2	0.254	1.999	2.82E-02	-2.98	no hit	-	-	-	-
Primary metabolic process/hydrolase activity	Tag_4313 CATGAAGTTTCTGGTAGTGTTAGCTG	1	2	0.254	1.999	2.82E-02	-2.98	Hv_Contig_10204	1124	26-51	AFI64307|neutral lipase *Helicoverpa armigera*	0.00E+00
-	Tag_4318 CATGTTCAGGATACGGCAAAAGCAAG	1	2	0.254	1.999	2.82E-02	-2.98	no hit	-	-	-	-
-	Tag_4320 CATGGGATCACCTGTGAAGAACACCC	1	2	0.254	1.999	2.82E-02	-2.98	no hit	-	-	-	-
-	Tag_4331 CATGCTGGCCGCCTGGGGGTACACCG	1	2	0.254	1.999	2.82E-02	-2.98	no hit	-	-	-	-
-	Tag_4334 CATGTCGTCATTTAATCAAGTCAACT	1	2	0.254	1.999	2.82E-02	-2.98	no hit	-	-	-	-
Primary metabolic process/hydrolase activity	Tag_4350 CATGAATTTATTTAGTTGGGTGTGTG	1	2	0.254	1.999	2.82E-02	-2.98	Hv_Contig_10468	1108	939-964	XP_011563249|6-phosphogluconolactonase *Plutella xylostella*	1.00E-108
-	Tag_4362 CATGGGGCCGTGGCTGCGCTGATACA	1	2	0.254	1.999	2.82E-02	-2.98	no hit	-	-	-	-
-	Tag_4364 CATGATTGTGTGAGTCTGTGCGGCCA	1	2	0.254	1.999	2.82E-02	-2.98	no hit	-	-	-	-
-	Tag_4370 CATGAAAGGCTAAGCCTGTATCTGAT	1	2	0.254	1.999	2.82E-02	-2.98	no hit	-	-	-	-
-	Tag_4378 CATGATCTGTGCTGCCCAACTCGGTC	1	2	0.254	1.999	2.82E-02	-2.98	no hit	-	-	-	-
-	Tag_4379 CATGTCCATGCGCTCCAGCGACCGGT	1	2	0.254	1.999	2.82E-02	-2.98	no hit	-	-	-	-
-	Tag_4381 CATGTTGAGCAGCCGCGCGGCTATAA	1	2	0.254	1.999	2.82E-02	-2.98	no hit	-	-	-	-
-	Tag_4385 CATGATCGTCCGAGACACGCCCCCAT	1	2	0.254	1.999	2.82E-02	-2.98	no hit	-	-	-	-
-	Tag_4386 CATGCGCCCGCAGGTGCACACCTACA	1	2	0.254	1.999	2.82E-02	-2.98	no hit	-	-	-	-
-	Tag_4387 CATGAGGACCAACAACTTCAAGGACG	1	2	0.254	1.999	2.82E-02	-2.98	no hit	-	-	-	-
Nucleic acid binding	Tag_4390 CATGCGCACGTGCCTGCTGCAGACGC	1	2	0.254	1.999	2.82E-02	-2.98	Hv_Contig_10449	1109	1047-1072	XP_013188719|lysine-specific demethylase 6A isoform X1 *Amyelois transitella*	0.00E+00
-	Tag_4396 CATGATCTGTGCTGGTTGGTCCAGCA	1	2	0.254	1.999	2.82E-02	-2.98	no hit	-	-	-	-
-	Tag_4398 CATGAGCGTGATCGCTACTCCCGCGA	1	2	0.254	1.999	2.82E-02	-2.98	no hit	-	-	-	-
-	Tag_4401 CATGGGGAGGTTCTGCTCAGGGCTAG	1	2	0.254	1.999	2.82E-02	-2.98	no hit	-	-	-	-
-	Tag_4409 CATGAGGAAGCACTTGGAACGCAATG	1	2	0.254	1.999	2.82E-02	-2.98	no hit	-	-	-	-
-	Tag_4415 CATGCTATCGCTGATGAATTCCTCCT	1	2	0.254	1.999	2.82E-02	-2.98	no hit	-	-	-	-
-	Tag_4421 CATGTCCGTGCGCTCCAGCGACCTGT	1	2	0.254	1.999	2.82E-02	-2.98	no hit	-	-	-	-
-	Tag_4423 CATGGTCGTCCGAGACACGCCCCCCA	1	2	0.254	1.999	2.82E-02	-2.98	no hit	-	-	-	-
-	Tag_4433 CATGCACGGCGGCTGGACCCCTAGCT	1	2	0.254	1.999	2.82E-02	-2.98	no hit	-	-	-	-
-	Tag_4439 CATGCCGCGCGAGGCTGCGCGCTCAG	1	2	0.254	1.999	2.82E-02	-2.98	no hit	-	-	-	-
-	Tag_4460 CATGGATCTCTGTTCAGTAGGTCTTG	1	2	0.254	1.999	2.82E-02	-2.98	no hit	-	-	-	-
-	Tag_4480 CATGTGCACCACCGCGCTTGGAAGAG	1	2	0.254	1.999	2.82E-02	-2.98	no hit	-	-	-	-
-	Tag_4482 CATGTGCAACATCATTATGTAACATC	1	2	0.254	1.999	2.82E-02	-2.98	no hit	-	-	-	-
-	Tag_4483 CATGTCCGGCATCGTGCAGAGCGTCG	1	2	0.254	1.999	2.82E-02	-2.98	no hit	-	-	-	-
-	Tag_4492 CATGGAGCGACTGCCTGCCTGGCCTC	1	2	0.254	1.999	2.82E-02	-2.98	no hit	-	-	-	-
-	Tag_4498 CATGGACGCGTCGTCGCTGCTGTCGA	1	2	0.254	1.999	2.82E-02	-2.98	no hit	-	-	-	-
-	Tag_4504 CATGCTCAAAAACCTTCGTTGTGAAG	1	2	0.254	1.999	2.82E-02	-2.98	no hit	-	-	-	-
-	Tag_4515 CATGCTGGACCTAGCGCAGGTGGCCG	1	2	0.254	1.999	2.82E-02	-2.98	no hit	-	-	-	-
-	Tag_4519 CATGCAGCGCCGCTGACCACCTCACA	1	2	0.254	1.999	2.82E-02	-2.98	no hit	-	-	-	-
-	Tag_4525 CATGGGGAGATTCTGCTCAGGACTGG	1	2	0.254	1.999	2.82E-02	-2.98	no hit	-	-	-	-
-	Tag_4533 CATGGAGGGCGGCGCGTACGGCGACG	1	2	0.254	1.999	2.82E-02	-2.98	no hit	-	-	-	-
-	Tag_4534 CATGTGTCCCGGTGACGAGCTTTCTG	1	2	0.254	1.999	2.82E-02	-2.98	no hit	-	-	-	-
Protein kinases	Tag_4546 CATGTCGCCCATCGACCTCAAGGCGG	1	2	0.254	1.999	2.82E-02	-2.98	Hv_Contig_15860	852	296-321	XP_012551487|tyrosine-protein kinase Abl isoform X3 *Bombyx mori*	2.00E-79
-	Tag_4549 CATGGGACTCACCGAGTATGACGCCG	1	2	0.254	1.999	2.82E-02	-2.98	no hit	-	-	-	-
-	Tag_4560 CATGAAATGAGTACAACAAATGGCTG	1	2	0.254	1.999	2.82E-02	-2.98	no hit	-	-	-	-
-	Tag_4564 CATGTTGGGTCTGGCGACCGCGCCTG	1	2	0.254	1.999	2.82E-02	-2.98	no hit	-	-	-	-
-	Tag_4566 CATGTTCACCCGAGGCCGGGCGCGGC	1	2	0.254	1.999	2.82E-02	-2.98	no hit	-	-	-	-
-	Tag_4567 CATGGCGGTGGCGTGCGCGACGGCCA	1	2	0.254	1.999	2.82E-02	-2.98	no hit	-	-	-	-
-	Tag_4569 CATGGAACTCGAACGCTCAGGCAGAC	1	2	0.254	1.999	2.82E-02	-2.98	no hit	-	-	-	-
-	Tag_4582 CATGAGCGGGAGCCCTGGCCGTAACC	1	2	0.254	1.999	2.82E-02	-2.98	no hit	-	-	-	-
-	Tag_4593 CATGTGTCCAAACGGTCACACTTTTG	1	2	0.254	1.999	2.82E-02	-2.98	no hit	-	-	-	-
-	Tag_4597 CATGTGTGCGCTGGTTCCTTGTGGGT	1	2	0.254	1.999	2.82E-02	-2.98	no hit	-	-	-	-
-	Tag_4598 CATGACACGCCCGTGTCGGGCGGTGA	1	2	0.254	1.999	2.82E-02	-2.98	no hit	-	-	-	-
-	Tag_4602 CATGCTGGCGGCGCGCTACTCGCTGC	1	2	0.254	1.999	2.82E-02	-2.98	no hit	-	-	-	-
-	Tag_4605 CATGGCCCTCGGCATCCGCTCACCCT	1	2	0.254	1.999	2.82E-02	-2.98	no hit	-	-	-	-
-	Tag_4613 CATGGGACCTGTGAAGAACACCCTTC	1	2	0.254	1.999	2.82E-02	-2.98	no hit	-	-	-	-
-	Tag_4621 CATGAGGCAGACGGTCACTGTGGGTA	1	2	0.254	1.999	2.82E-02	-2.98	no hit	-	-	-	-
-	Tag_4626 CATGCTGGGATGCGCTACCTCTACAG	1	2	0.254	1.999	2.82E-02	-2.98	no hit	-	-	-	-
-	Tag_4641 CATGGGACCTGTGAAGAACACCCTTT	1	2	0.254	1.999	2.82E-02	-2.98	no hit	-	-	-	-
-	Tag_4644 CATGGTGGGCGGCACGTCTACGTCAG	1	2	0.254	1.999	2.82E-02	-2.98	no hit	-	-	-	-
-	Tag_4647 CATGATCTGAGTTCAAACCGGCGTAG	1	2	0.254	1.999	2.82E-02	-2.98	no hit	-	-	-	-

-	Tag_4648 CATGTTAGAAGACGATAATAAAACGG	1	2	0.254	1.999	2.82E-02	-2.98	no hit	-	-	-	-
-	Tag_4649 CATGTGGGACACCCGCTCGATCCATT	1	2	0.254	1.999	2.82E-02	-2.98	no hit	-	-	-	-
-	Tag_4659 CATGTACAACGCGACCGTATCGACAG	1	2	0.254	1.999	2.82E-02	-2.98	no hit	-	-	-	-
-	Tag_4667 CATGCCCGGCTTACCCGGCGGCGCGA	1	2	0.254	1.999	2.82E-02	-2.98	no hit	-	-	-	-
-	Tag_4685 CATGTGCAGTGTGAGGATTCTGTGGG	1	2	0.254	1.999	2.82E-02	-2.98	no hit	-	-	-	-
-	Tag_4689 CATGATCTGAGTTCAGACCGGTGTGG	1	2	0.254	1.999	2.82E-02	-2.98	no hit	-	-	-	-
-	Tag_4690 CATGCGCCTGTGCGGCGGCTTGGGCC	1	2	0.254	1.999	2.82E-02	-2.98	no hit	-	-	-	-
-	Tag_4695 CATGTTGAGCAGCCGCGCGGCTATAC	1	2	0.254	1.999	2.82E-02	-2.98	no hit	-	-	-	-
-	Tag_4696 CATGGGGCAGACGGTCGCTGTGGGGG	1	2	0.254	1.999	2.82E-02	-2.98	no hit	-	-	-	-
-	Tag_4700 CATGCCGTCGACCACAGGTGAAGCAC	1	2	0.254	1.999	2.82E-02	-2.98	no hit	-	-	-	-
-	Tag_4705 CATGGCACGCAAACTTTGGGATGTGG	1	2	0.254	1.999	2.82E-02	-2.98	no hit	-	-	-	-
-	Tag_4710 CATGCGCGGGCGCGCCGGCCCGCCAG	1	2	0.254	1.999	2.82E-02	-2.98	no hit	-	-	-	-
-	Tag_4720 CATGAGGCAGACGGTCGCTGCGGGGG	1	2	0.254	1.999	2.82E-02	-2.98	no hit	-	-	-	-
-	Tag_4726 CATGGACCGCGGGGAGGGCGGTCAGG	1	2	0.254	1.999	2.82E-02	-2.98	no hit	-	-	-	-
-	Tag_4728 CATGCTGAACCTGGCGCAGGTGGCCG	1	2	0.254	1.999	2.82E-02	-2.98	no hit	-	-	-	-
-	Tag_4742 CATGCAGGCAGCCGCAGCTGAAGTAA	1	2	0.254	1.999	2.82E-02	-2.98	no hit	-	-	-	-
-	Tag_4748 CATGAGCCAGGAGGTGGACGTGCTAA	1	2	0.254	1.999	2.82E-02	-2.98	no hit	-	-	-	-
-	Tag_4766 CATGGTTGCACTGGCGACGGTGGCGG	1	2	0.254	1.999	2.82E-02	-2.98	no hit	-	-	-	-
-	Tag_4776 CATGAGGATTTCCCCCCGAAAACAAA	1	2	0.254	1.999	2.82E-02	-2.98	no hit	-	-	-	-
-	Tag_4794 CATGTGGACATCTTGTCGTTAGTGTG	1	2	0.254	1.999	2.82E-02	-2.98	no hit	-	-	-	-
-	Tag_4801 CATGGGGAAGAGGCTGCGCTGGTGCG	1	2	0.254	1.999	2.82E-02	-2.98	no hit	-	-	-	-
Unknown	Tag_4802 CATGTTAAACCTTGGCTCGAATGCAC	1	2	0.254	1.999	2.82E-02	-2.98	Hv_Contig_26862	579	267-292	XP_012156112|uncharacterized protein LOC105664733 *Ceratitis capitata*	2.00E-27
-	Tag_4812 CATGTTCGTGGTGAGGGCGTACCTGC	1	2	0.254	1.999	2.82E-02	-2.98	no hit	-	-	-	-
-	Tag_4813 CATGGATCTCTGTTCAGTAGGTCTGG	1	2	0.254	1.999	2.82E-02	-2.98	no hit	-	-	-	-
-	Tag_4816 CATGCTGGACCTGGCGCAGGTGGCAA	1	2	0.254	1.999	2.82E-02	-2.98	no hit	-	-	-	-
-	Tag_4817 CATGGACGAGTATAAAAGCAGTCCGG	1	2	0.254	1.999	2.82E-02	-2.98	no hit	-	-	-	-
-	Tag_4828 CATGACCTGTGCCGGCTGGCCCAGCA	1	2	0.254	1.999	2.82E-02	-2.98	no hit	-	-	-	-
-	Tag_4838 CATGGGCAGACGCTAAGCTGACCGAA	1	2	0.254	1.999	2.82E-02	-2.98	no hit	-	-	-	-
-	Tag_4840 CATGGCGCGCTCGGTTCCGCGATGCA	1	2	0.254	1.999	2.82E-02	-2.98	no hit	-	-	-	-
-	Tag_4841 CATGAACAAGAAGAGAAGTAATTCTA	1	2	0.254	1.999	2.82E-02	-2.98	no hit	-	-	-	-
-	Tag_4850 CATGCGCGCAAGCGAGACGCGCGAAC	1	2	0.254	1.999	2.82E-02	-2.98	no hit	-	-	-	-
-	Tag_4860 CATGTACCTGATGCCGGTGGGAGACG	1	2	0.254	1.999	2.82E-02	-2.98	no hit	-	-	-	-
-	Tag_4862 CATGTGGCGGACTCCGTTGCTGAGCC	1	2	0.254	1.999	2.82E-02	-2.98	no hit	-	-	-	-
-	Tag_4896 CATGCGGATCCTCGCTGAGAAACTGA	1	2	0.254	1.999	2.82E-02	-2.98	no hit	-	-	-	-
-	Tag_4897 CATGCGTATCAGCTCCGTCAGCTCCG	1	2	0.254	1.999	2.82E-02	-2.98	no hit	-	-	-	-
-	Tag_4901 CATGTGTATAATGAACTTAAAGGGGA	1	2	0.254	1.999	2.82E-02	-2.98	no hit	-	-	-	-
-	Tag_4919 CATGGTAATGATTATTTTCAAAGAGG	1	2	0.254	1.999	2.82E-02	-2.98	no hit	-	-	-	-
-	Tag_4926 CATGGTGGGCCGCATCGGCTCCATGA	1	2	0.254	1.999	2.82E-02	-2.98	no hit	-	-	-	-
Nucleic acid binding	Tag_4930 CATGTCAAAAGTTGATGAAGTGACCG	1	2	0.254	1.999	2.82E-02	-2.98	Hv_Contig_2368	2242	469-494	XP_013165204|RNA-binding motif protein, X-linked 2 *Papilio xuthus*	9.00E-84
-	Tag_4935 CATGTGCCCACACTTGCTGCCTTGCG	1	2	0.254	1.999	2.82E-02	-2.98	no hit	-	-	-	-
-	Tag_4936 CATGTGCGCGCTGGCTCCCCGTGGGC	1	2	0.254	1.999	2.82E-02	-2.98	no hit	-	-	-	-
-	Tag_4942 CATGTACCTATTTCTCTCGATGATGT	1	2	0.254	1.999	2.82E-02	-2.98	no hit	-	-	-	-
-	Tag_4946 CATGGCCGACGCTACGCTGCTGCCTG	1	2	0.254	1.999	2.82E-02	-2.98	no hit	-	-	-	-
-	Tag_4967 CATGCGCGCGCGCAGCCAGTTAGCTC	1	2	0.254	1.999	2.82E-02	-2.98	no hit	-	-	-	-
-	Tag_4971 CATGTCCGTGCGCTCCTGCGACCGTA	1	2	0.254	1.999	2.82E-02	-2.98	no hit	-	-	-	-
-	Tag_4979 CATGTCAAAAGTCGGCGGCAATATTA	1	2	0.254	1.999	2.82E-02	-2.98	no hit	-	-	-	-
-	Tag_4981 CATGGCAGTAGCGGCGAGGCCGCGTG	1	2	0.254	1.999	2.82E-02	-2.98	no hit	-	-	-	-
-	Tag_4990 CATGGGCCCAACGTGGACCCATTGGG	1	2	0.254	1.999	2.82E-02	-2.98	no hit	-	-	-	-
-	Tag_4994 CATGGTGCAGCTACTCAGCGTGGTGG	1	2	0.254	1.999	2.82E-02	-2.98	no hit	-	-	-	-
-	Tag_4997 CATGGGTTGGCGCGGTGGCTTCAACA	1	2	0.254	1.999	2.82E-02	-2.98	no hit	-	-	-	-
-	Tag_4998 CATGTGACGTCAGTTGTATGAAGAAG	1	2	0.254	1.999	2.82E-02	-2.98	no hit	-	-	-	-
-	Tag_5001 CATGATCTGTGCTGGTATCTTGGATA	1	2	0.254	1.999	2.82E-02	-2.98	no hit	-	-	-	-
-	Tag_5004 CATGTGCAATAAAGTACAACTTCATA	1	2	0.254	1.999	2.82E-02	-2.98	no hit	-	-	-	-
-	Tag_5006 CATGTTGTAATGGTGTTTATGTGACG	1	2	0.254	1.999	2.82E-02	-2.98	no hit	-	-	-	-
-	Tag_5007 CATGGCCGCGTTGAACTTTGCTATGC	1	2	0.254	1.999	2.82E-02	-2.98	no hit	-	-	-	-
-	Tag_5018 CATGCACAAGCTACGGCGCTGACGCT	1	2	0.254	1.999	2.82E-02	-2.98	no hit	-	-	-	-
-	Tag_5023 CATGGCCCTCGGCATCCGCTCACTCA	1	2	0.254	1.999	2.82E-02	-2.98	no hit	-	-	-	-
-	Tag_5025 CATGTCCGTGCGCTCAGCGACCGTTG	1	2	0.254	1.999	2.82E-02	-2.98	no hit	-	-	-	-

Cytoskeleton	Tag_5027 CATGCCCACCAGGCCCAACAAGGACC	1	2	0.254	1.999	2.82E-02	-2.98	Hv_Contig_28756	549	277-302	XP_013187968|WAS/WASL-interacting protein family member 3-like isoform X1 *Amyelois transitella*	1.00E-75
-	Tag_5032 CATGATCTGTGCCGGCTGGCCCAGAC	1	2	0.254	1.999	2.82E-02	-2.98	no hit	-	-	-	-
-	Tag_5035 CATGGACGGCGGCGTGCAGGCGCAGT	1	2	0.254	1.999	2.82E-02	-2.98	no hit	-	-	-	-
-	Tag_5036 CATGATTTGAGTTCAAACCGGTGTAG	1	2	0.254	1.999	2.82E-02	-2.98	no hit	-	-	-	-
-	Tag_5059 CATGGCTCTCGGTATCCGCTCACCCT	1	2	0.254	1.999	2.82E-02	-2.98	no hit	-	-	-	-
-	Tag_5061 CATGTCGACTTGCCCGTTATCACAGC	1	2	0.254	1.999	2.82E-02	-2.98	no hit	-	-	-	-
-	Tag_5063 CATGGAGTTTCTTGCCCGTTCTTCTA	1	2	0.254	1.999	2.82E-02	-2.98	no hit	-	-	-	-
-	Tag_5071 CATGATCTGAGTTCAAGCCGGTGTAC	1	2	0.254	1.999	2.82E-02	-2.98	no hit	-	-	-	-
-	Tag_5072 CATGTGCGACGTTCCTTGTGACGTCA	1	2	0.254	1.999	2.82E-02	-2.98	no hit	-	-	-	-
-	Tag_5079 CATGGCTCTCGGTATCCGCTCACTCA	1	2	0.254	1.999	2.82E-02	-2.98	no hit	-	-	-	-
Nucleic acid binding	Tag_5083 CATGTTAATTCTAGCTGTGCCACATA	1	2	0.254	1.999	2.82E-02	-2.98	Hv_Contig_5897	1507	971-996	XP_013189481|Y-box factor homolog *Amyelois transitella*	2.00E-53
-	Tag_5086 CATGCGACAGGCACGCGTGTAGGCCG	1	2	0.254	1.999	2.82E-02	-2.98	no hit	-	-	-	-
-	Tag_5088 CATGCGTCCCGGCCGTCTCGACCGTG	1	2	0.254	1.999	2.82E-02	-2.98	no hit	-	-	-	-
-	Tag_5101 CATGGAAAAAAAAAAAAACTGCTGGG	1	2	0.254	1.999	2.82E-02	-2.98	no hit	-	-	-	-
-	Tag_5104 CATGCACGGCAGATGGAGCCCTAGCT	1	2	0.254	1.999	2.82E-02	-2.98	no hit	-	-	-	-
-	Tag_5113 CATGGGGCAGTGGCTGCGCTGATGCT	1	2	0.254	1.999	2.82E-02	-2.98	no hit	-	-	-	-
-	Tag_5130 CATGCTCACCGTCAGCGCTGAGGAGG	1	2	0.254	1.999	2.82E-02	-2.98	no hit	-	-	-	-
-	Tag_5132 CATGTCCGTGCGCTCCAGCGACCAGG	1	2	0.254	1.999	2.82E-02	-2.98	no hit	-	-	-	-
-	Tag_5133 CATGGGAATCAGAATGCTCCACCCGC	1	2	0.254	1.999	2.82E-02	-2.98	no hit	-	-	-	-
-	Tag_5136 CATGTTGAGTCTGGCGACCGCGCTTT	1	2	0.254	1.999	2.82E-02	-2.98	no hit	-	-	-	-
-	Tag_5139 CATGATTGTGTGAACCTGTGCGGCCA	1	2	0.254	1.999	2.82E-02	-2.98	no hit	-	-	-	-
-	Tag_5141 CATGGGGGCTATCGCCGTCGCCCTCC	1	2	0.254	1.999	2.82E-02	-2.98	no hit	-	-	-	-
-	Tag_5154 CATGCCGCAGCACGCGCTGGGCGTGA	1	2	0.254	1.999	2.82E-02	-2.98	no hit	-	-	-	-
-	Tag_5157 CATGGTTCCAAGATGTGACCGGCATC	1	2	0.254	1.999	2.82E-02	-2.98	no hit	-	-	-	-
-	Tag_5172 CATGCCCGTACAGAAGCAGACCCATA	1	2	0.254	1.999	2.82E-02	-2.98	no hit	-	-	-	-
-	Tag_5180 CATGCTGGCGGTGCGCTACTCGCCGC	1	2	0.254	1.999	2.82E-02	-2.98	no hit	-	-	-	-
-	Tag_5181 CATGTCTAGTTGCTGGTTGACTATAA	1	2	0.254	1.999	2.82E-02	-2.98	no hit	-	-	-	-
-	Tag_5182 CATGGAGTGCGGACTGCGCTTCGAGT	1	2	0.254	1.999	2.82E-02	-2.98	no hit	-	-	-	-
-	Tag_5184 CATGTAAGTGTCGGGCGGTCAGCCCA	1	2	0.254	1.999	2.82E-02	-2.98	no hit	-	-	-	-
-	Tag_5186 CATGATCTGAGCTCAAACCGGTGTAG	1	2	0.254	1.999	2.82E-02	-2.98	no hit	-	-	-	-
-	Tag_5201 CATGTTCGTGCGCTCCAGCGACCGTG	1	2	0.254	1.999	2.82E-02	-2.98	no hit	-	-	-	-
-	Tag_5210 CATGGGACCACCTGTGAAGAGCACCC	1	2	0.254	1.999	2.82E-02	-2.98	no hit	-	-	-	-
-	Tag_5211 CATGAGCGGGAGCCCTGGCCGTAAGG	1	2	0.254	1.999	2.82E-02	-2.98	no hit	-	-	-	-
-	Tag_5219 CATGCACCATCGAGAAGACACTGCAC	1	2	0.254	1.999	2.82E-02	-2.98	no hit	-	-	-	-
mRNA processing/splicing	Tag_5224 CATGCCACAACCTCACTGAGGAGAGC	1	2	0.254	1.999	2.82E-02	-2.98	Hv_Contig_8289	1260	695-720	XP_001986295|GH20604 *Drosophila grimshawi*	6.00E-58
-	Tag_5228 CATGCCTTGCCCCGCGCCCCGCGACC	1	2	0.254	1.999	2.82E-02	-2.98	no hit	-	-	-	-
-	Tag_5232 CATGTTATACTTTTAGGGAGTCACCG	1	2	0.254	1.999	2.82E-02	-2.98	no hit	-	-	-	-
-	Tag_1178 CATGGGACCAACAAGGCAAGAACGGA	24	48	6.090	47.969	6.12E-18	-2.98	no hit	-	-	-	-
-	Tag_1751 CATGGCTCGCAGCCGCCCCCTGGTTG	12	24	3.045	23.985	6.35E-10	-2.98	no hit	-	-	-	-
-	Tag_1681 CATGATTCCACACAAGACTGAGCGCA	13	26	3.299	25.983	1.35E-10	-2.98	no hit	-	-	-	-
-	Tag_1697 CATGCGCCGCAATCGCCAACAGTCCA	13	26	3.299	25.983	1.35E-10	-2.98	no hit	-	-	-	-
-	Tag_3027 CATGCCCAGCGCCGCCACCACCTTCG	3	6	0.761	5.996	9.36E-04	-2.98	no hit	-	-	-	-
-	Tag_3050 CATGATCTGTGCCGGTTGGTCCAGGG	3	6	0.761	5.996	9.36E-04	-2.98	no hit	-	-	-	-
-	Tag_3053 CATGGGGTGACTTTGCGGTCGCTGGT	3	6	0.761	5.996	9.36E-04	-2.98	no hit	-	-	-	-
-	Tag_3068 CATGCGGCCCGCGGGCCGCGCGTTGT	3	6	0.761	5.996	9.36E-04	-2.98	no hit	-	-	-	-
-	Tag_3074 CATGTGTGCGCTGGCTCCCTGTGGGT	3	6	0.761	5.996	9.36E-04	-2.98	no hit	-	-	-	-
-	Tag_3152 CATGGGGCCGTGGCTGTGCTGATGCT	3	6	0.761	5.996	9.36E-04	-2.98	no hit	-	-	-	-
-	Tag_3216 CATGTACCGGTAGCTTATTGCCAGTC	3	6	0.761	5.996	9.36E-04	-2.98	no hit	-	-	-	-
-	Tag_3222 CATGGGACTCACCGAGTGCGACGCCG	3	6	0.761	5.996	9.36E-04	-2.98	no hit	-	-	-	-
Nucleic acid binding	Tag_3236 CATGGAAATCAATAAAATTATCTTAT	3	6	0.761	5.996	9.36E-04	-2.98	Hv_Contig_2048	2366	2288-2313	XP_012551457|protein Jumonji isoform X1 *Bombyx mori*	2.00E-29
Nucleic acid binding	Tag_3241 CATGAGGCCGAACAACAACGGAAGTT	3	6	0.761	5.996	9.36E-04	-2.98	Hv_Contig_10158	1127	1041-1066	AFD01656|mod(mdg4) protein *Helicoverpa armigera*	0.00E+00
Translation/Ribosome biogenesis	Tag_3293 CATGCAGGGCACCGTCAGTGACTTAG	3	6	0.761	5.996	9.36E-04	-2.98	Hv_Contig_17452	798	343-318	ACY95340|ribosomal protein L10A *Manduca sexta*	4.00E-138
-	Tag_3340 CATGCGTGCCGCGTCAGACGCGGTTG	3	6	0.761	5.996	9.36E-04	-2.98	no hit	-	-	-	-
-	Tag_3350 CATGAGGAGACAGGGCGTCGTCGTAG	3	6	0.761	5.996	9.36E-04	-2.98	no hit	-	-	-	-
-	Tag_3377 CATGGACCACCACCTGGAGGCCCCCG	3	6	0.761	5.996	9.36E-04	-2.98	no hit	-	-	-	-
Protein folding/Recycling	Tag_3397 CATGTACCGGCGCCACGACACGCTGC	3	6	0.761	5.996	9.36E-04	-2.98	Hv_Contig_11266	1062	884-909	XP_004931583.2|ubiquitin carboxyl-terminal hydrolase 10 *Bombyx mori*	9.00E-137
-	Tag_3417 CATGTTCTTACTTTGTGAACCCTATG	2	4	0.508	3.997	4.95E-03	-2.98	no hit	-	-	-	-
-	Tag_3418 CATGGTCTGAGTTCAAACCGGTGTAG	2	4	0.508	3.997	4.95E-03	-2.98	no hit	-	-	-	-

-	Tag_3426 CATGGTTGGTGCGCGCGGGGGCCTGT	2	4	0.508	3.997	4.95E-03	-2.98	no hit	-	-	-	-
-	Tag_3436 CATGGTTGGTGCGCGCGGGGGGCTTG	2	4	0.508	3.997	4.95E-03	-2.98	no hit	-	-	-	-
-	Tag_3442 CATGATCGTCCGGGACACGCCCCCCA	2	4	0.508	3.997	4.95E-03	-2.98	no hit	-	-	-	-
-	Tag_3457 CATGGCCTACGCCGCCCGCATCGGCC	2	4	0.508	3.997	4.95E-03	-2.98	no hit	-	-	-	-
-	Tag_3486 CATGCGACAGGCACGCGTGTAGGCTA	2	4	0.508	3.997	4.95E-03	-2.98	no hit	-	-	-	-
-	Tag_3495 CATGCCCCTACACACACTAAGTGAAG	2	4	0.508	3.997	4.95E-03	-2.98	no hit	-	-	-	-
-	Tag_3502 CATGGAAATCAATAAAATTATCTTAG	2	4	0.508	3.997	4.95E-03	-2.98	no hit	-	-	-	-
-	Tag_3505 CATGTGCGACGTTCTTTGTGACGTAG	2	4	0.508	3.997	4.95E-03	-2.98	no hit	-	-	-	-
-	Tag_3509 CATGCTGCAGCACGCGCTGGGCGTCA	2	4	0.508	3.997	4.95E-03	-2.98	no hit	-	-	-	-
-	Tag_3513 CATGCGTCATCTGAAAATCGTGAGAG	2	4	0.508	3.997	4.95E-03	-2.98	no hit	-	-	-	-
-	Tag_3525 CATGAGGCAGACGGTCGCTGTGGACG	2	4	0.508	3.997	4.95E-03	-2.98	no hit	-	-	-	-
-	Tag_3578 CATGGAGCGACTGCCTGTCTGGCCAC	2	4	0.508	3.997	4.95E-03	-2.98	no hit	-	-	-	-
Transport/Trafficking	Tag_3594 CATGGAACTCAAATTAATTATTCCCC	2	4	0.508	3.997	4.95E-03	-2.98	Hv_Contig_36788	370	40-15	ABK29476|Titin-like protein *Helicoverpa armigera*	5.00E-18
Nucleic acid binding	Tag_3599 CATGTTTAACAAGTCTTATTTGTAAT	2	4	0.508	3.997	4.95E-03	-2.98	Hv_Contig_5897	1507	1390-1415	XP_013189481|Y-box factor homolog *Amyelois transitella*	2.00E-53
-	Tag_3600 CATGGGCGGCAGTCGCGCAGAAAGAG	2	4	0.508	3.997	4.95E-03	-2.98	no hit	-	-	-	-
-	Tag_3615 CATGGGAACACTCAAGAAGGACCGAG	2	4	0.508	3.997	4.95E-03	-2.98	no hit	-	-	-	-
-	Tag_3626 CATGTTGTGATGGGGGCTCAACCTTG	2	4	0.508	3.997	4.95E-03	-2.98	no hit	-	-	-	-
-	Tag_3638 CATGAGGGCGCCAGACCCCGCGCTCA	2	4	0.508	3.997	4.95E-03	-2.98	no hit	-	-	-	-
Primary metabolic process/hydrolase activity	Tag_3671 CATGACAGCGAGCAAGAAACCAAAAA	2	4	0.508	3.997	4.95E-03	-2.98	Hv_Contig_4605	1706	1669-1694	AFI64313|acidic lipase *Helicoverpa armigera*	0.00E+00
Primary metabolic process/hydrolase activity	Tag_3678 CATGATGCTGCCTGGCACCTCAGTCA	2	4	0.508	3.997	4.95E-03	-2.98	Hv_Contig_2921	2063	1294-1319	KPI98467|Maltase 1 *Papilio xuthus*	0.00E+00
-	Tag_3692 CATGAGGCCGTGGCTGCGCTGATGCT	2	4	0.508	3.997	4.95E-03	-2.98	no hit	-	-	-	-
Signal transduction	Tag_3728 CATGTAGTTAGTGGGGCTGCGGTAGG	2	4	0.508	3.997	4.95E-03	-2.98	Hv_Contig_3300	1964	1655-1680	XP_004933453|ras-like GTP-binding protein Rho1 isoform X1 *Bombyx mori*	2.00E-128
-	Tag_3730 CATGCGTTGCACTACAGCACGGGCCT	2	4	0.508	3.997	4.95E-03	-2.98	no hit	-	-	-	-
-	Tag_3748 CATGCTCACCGCTAGCGCTGAGGAGG	2	4	0.508	3.997	4.95E-03	-2.98	no hit	-	-	-	-
-	Tag_3755 CATGTCCGTGCGCTCCAGCGGCCGTA	2	4	0.508	3.997	4.95E-03	-2.98	no hit	-	-	-	-
-	Tag_3765 CATGACTGGCTCCAGCATCATCAACG	2	4	0.508	3.997	4.95E-03	-2.98	no hit	-	-	-	-
-	Tag_3802 CATGACGGCTCCTTGTAGCTCGTGTG	2	4	0.508	3.997	4.95E-03	-2.98	no hit	-	-	-	-
-	Tag_3803 CATGTCCGAGGACTGCCGCCGTCGCG	2	4	0.508	3.997	4.95E-03	-2.98	no hit	-	-	-	-
-	Tag_3807 CATGTGCCCACACTTGCTGTCTTGTG	2	4	0.508	3.997	4.95E-03	-2.98	no hit	-	-	-	-
-	Tag_3813 CATGCCCGGCGCGCAGTCTTCGTCTG	2	4	0.508	3.997	4.95E-03	-2.98	no hit	-	-	-	-
-	Tag_3814 CATGCGCATCAGCTCCGTCAGCTTAA	2	4	0.508	3.997	4.95E-03	-2.98	no hit	-	-	-	-
Unknown	Tag_3819 CATGCGCGGCGAAGGCGGCGCGCGCC	2	4	0.508	3.997	4.95E-03	-2.98	Hv_Contig_525	3714	1673-1648	XP_004924044|uncharacterized protein LOC101745370 isoform X2 *Bombyx mori*	0.00E+00
-	Tag_3838 CATGAAGATTGGCCGCATCGAGGAGA	2	4	0.508	3.997	4.95E-03	-2.98	no hit	-	-	-	-
-	Tag_3862 CATGCGGCTCGTGCGGCGGGCGCTAG	2	4	0.508	3.997	4.95E-03	-2.98	no hit	-	-	-	-
-	Tag_3941 CATGTCGCTCTCGCGCCATTGACCAG	2	4	0.508	3.997	4.95E-03	-2.98	no hit	-	-	-	-
-	Tag_3950 CATGGGACTCACCTAGTACGACGCCG	2	4	0.508	3.997	4.95E-03	-2.98	no hit	-	-	-	-
-	Tag_3957 CATGATTGAAATATGCATCTATTTAG	2	4	0.508	3.997	4.95E-03	-2.98	no hit	-	-	-	-
-	Tag_3978 CATGGATTGACACCCCCATCGCTCCA	2	4	0.508	3.997	4.95E-03	-2.98	no hit	-	-	-	-
-	Tag_3984 CATGCTGCCCCTGACTGCCGCGCGCG	2	4	0.508	3.997	4.95E-03	-2.98	no hit	-	-	-	-
-	Tag_4003 CATGATCTGTGGCGGCTGGCCCAGCG	2	4	0.508	3.997	4.95E-03	-2.98	no hit	-	-	-	-
-	Tag_4009 CATGGGGACCCTCGCCACTGCCTACT	2	4	0.508	3.997	4.95E-03	-2.98	no hit	-	-	-	-
-	Tag_2816 CATGAGGCAGTCGGTCGCTGTGGGTG	4	8	1.015	7.995	1.84E-04	-2.98	no hit	-	-	-	-
-	Tag_2880 CATGGGACTCACTGAGTACGACGCCG	4	8	1.015	7.995	1.84E-04	-2.98	no hit	-	-	-	-
-	Tag_2899 CATGGCCTACGCCGCCTGCATCGGCG	4	8	1.015	7.995	1.84E-04	-2.98	no hit	-	-	-	-
-	Tag_2939 CATGCTGCCCCCGACTGCCGCACGCG	4	8	1.015	7.995	1.84E-04	-2.98	no hit	-	-	-	-
Signal transduction	Tag_2973 CATGTGCTGCGTGAAGGCGCTGTTCT	4	8	1.015	7.995	1.84E-04	-2.98	Hv_Contig_7015	1380	662-687	NP_001296482|protein sprouty *Bombyx mori*	7.00E-52
-	Tag_3024 CATGTGTACGGCACAGTGCCGGCGGG	4	8	1.015	7.995	1.84E-04	-2.98	no hit	-	-	-	-
-	Tag_1941 CATGTTCTGAGGGCGTGGCGCCATTA	10	20	2.538	19.987	1.42E-08	-2.98	no hit	-	-	-	-
-	Tag_1789 CATGGGGAAGAGGCTGCGCTGATGTG	11	21	2.791	20.987	1.01E-08	-2.91	no hit	-	-	-	-
-	Tag_1387 CATGCTGTTGGCTGCTGCAGCCACCA	19	36	4.822	35.977	1.44E-13	-2.90	no hit	-	-	-	-
Translation/Ribosome biogenesis	Tag_410 CATGATTCCACACAAGACTGAGCGCG	147	275	37.304	274.824	2.88E-89	-2.88	Hv_Contig_22805	648	608-633	AGA17991|ribosomal protein L13A, partial *Agrotis ipsilon*	4.00E-56
-	Tag_1559 CATGGGGAGCGACGCCGCCGTGGGCG	15	28	3.807	27.982	6.80E-11	-2.88	no hit	-	-	-	-
Primary metabolic process/hydrolase activity	Tag_2204 CATGCCAGGGTGCATCACCTCGATGT	7	13	1.776	12.992	5.26E-06	-2.87	Hv_Contig_27031	576	357-382	ACB54944|lipase *Helicoverpa armigera*	7.00E-31
Primary metabolic process/hydrolase activity	Tag_1320 CATGAGGAGACAGGGCGTCGTCGTAA	20	37	5.075	36.976	1.01E-13	-2.87	Hv_Contig_4495	1723	1654-1679	XP_004928875|apyrase-like *Bombyx mori*	0.00E+00
-	Tag_1163 CATGGCCGACTATCCGCAGCGCGTGG	25	46	6.344	45.970	1.55E-16	-2.86	no hit	-	-	-	-
-	Tag_2369 CATGCCCGTACAGAAGCAGACCCGCA	6	11	1.523	10.993	2.56E-05	-2.85	no hit	-	-	-	-
Transport/Trafficking	Tag_2392 CATGTTTCAACTGTTGTATAAGTTTT	6	11	1.523	10.993	2.56E-05	-2.85	Hv_Contig_26487	585	524-549	ACB54950|fatty acid-binding protein 3 *Helicoverpa armigera*	7.00E-42
Unknown	Tag_2399 CATGCCATTGTCAAGCAAAGATTGGC	6	11	1.523	10.993	2.56E-05	-2.85	Hv_Contig_16767	819	110-135	ELU10640|hypothetical protein CAPTEDRAFT_215177 *Capitella teleta*	1.00E-05

-	Tag_1402 CATGATTGTGTGAGCCTGTGCGGCCC	18	33	4.568	32.979	2.19E-12	-2.85	no hit	-	-	-	-
-	Tag_870 CATGTGTGGGGAGGAGTGAGCGAGGC	40	73	10.151	72.953	6.07E-25	-2.85	no hit	-	-	-	-
Translation/Ribosome biogenesis	Tag_1830 CATGGCGCTGCTGCGAGAACCCTGGA	11	20	2.791	19.987	3.33E-08	-2.84	Hv_Contig_37974	344	207-232	XP_013137760|phenylalanine--tRNA ligase beta subunit *Papilio polytes*	7.00E-51
Unknown	Tag_1129 CATGATCCGCGAACGAAGTCGCTGGC	26	47	6.598	46.970	1.09E-16	-2.83	Hv_Contig_17264	804	126-151	KOB63195|Uncharacterized protein OBRU01_24925, partial *Operophtera brumata*	5.00E-25
-	Tag_2501 CATGGGGCCGTGGCTGCGCTGATACT	5	9	1.269	8.994	1.26E-04	-2.83	no hit	-	-	-	-
-	Tag_2566 CATGATCTGCGTACGAAGTCGCGGGC	5	9	1.269	8.994	1.26E-04	-2.83	no hit	-	-	-	-
-	Tag_2688 CATGTGCGCGCTGGCTCCCCGTGGGT	5	9	1.269	8.994	1.26E-04	-2.83	no hit	-	-	-	-
-	Tag_1566 CATGAGGCTGCCAAGGCCCTTGACGA	15	27	3.807	26.983	2.22E-10	-2.83	no hit	-	-	-	-
Cytoskeleton	Tag_1911 CATGAAATAAAACAAAACAATTATGT	10	18	2.538	17.988	1.58E-07	-2.83	Hv_Contig_18901	755	722-747	XP_015215669|tubulin alpha-1C chain-like *Lepisosteus oculatus*	9.00E-67
-	Tag_1967 CATGCTGTCGGTGCTGGGCTACGACT	10	18	2.538	17.988	1.58E-07	-2.83	no hit	-	-	-	-
Cytoskeleton	Tag_64 CATGCTGCCCCCGACTGCCGCGCGCG	1386	2481	351.723	2479.409	0.00E+00	-2.82	Hv_Contig_4093	1802	1747-1772	XP_013187103|nesprin-1 isoform X1 *Amyelois transitella*	1.00E-180
Unknown	Tag_1408 CATGAGGACCAACAACTTCAAGGACC	18	32	4.568	31.979	7.10E-12	-2.81	Hv_Contig_23240	639	136-161	ABX39545|single domain major allergen 2 protein, partial *Helicoverpa armigera*	3.00E-106
-	Tag_2046 CATGGCGCTGCAGGTGGACGTGTCCA	9	16	2.284	15.990	7.54E-07	-2.81	no hit	-	-	-	-
Primary metabolic process/hydrolase activity	Tag_591 CATGCACGTTCGAGGCGTGCGCCGCG	84	149	21.317	148.904	7.17E-48	-2.80	Hv_Contig_16058	845	365-390	XP_008180263|putative nuclease HARBI1 *Acyrthosiphon pisum*	2.00E-08
-	Tag_1499 CATGCCCGGCTTCCCCGGCGGCGCAG	16	28	4.060	27.982	1.54E-10	-2.78	no hit	-	-	-	-
Translation/Ribosome biogenesis	Tag_2080 CATGGAAGACACCCTCAAAGGAGAAG	8	14	2.030	13.991	3.62E-06	-2.78	Hv_Contig_6644	1418	901-926	ACB12079|ribosomal protein L4 *Heliothis virescens*	0.00E+00
-	Tag_925 CATGCGCGCAAGCGAGACGCGCGATT	36	63	9.136	62.960	2.73E-21	-2.78	no hit	-	-	-	-
-	Tag_1749 CATGAGGCAGACGGTCGCTGTGGGCC	12	21	3.045	20.987	2.30E-08	-2.78	no hit	-	-	-	-
-	Tag_2764 CATGGGCGGCGGGCACGGCGGCGTGT	4	7	1.015	6.996	6.35E-04	-2.78	no hit	-	-	-	-
-	Tag_2766 CATGATTCCACACAAGACTGAGCGAG	4	7	1.015	6.996	6.35E-04	-2.78	no hit	-	-	-	-
-	Tag_2778 CATGCCCGGCTTCCCCAGCGGCGCGA	4	7	1.015	6.996	6.35E-04	-2.78	no hit	-	-	-	-
-	Tag_2824 CATGTCCGTGCGCTCCAGTGACCGTA	4	7	1.015	6.996	6.35E-04	-2.78	no hit	-	-	-	-
-	Tag_2852 CATGAGCCCCCTCCTGCCCGACACTT	4	7	1.015	6.996	6.35E-04	-2.78	no hit	-	-	-	-
-	Tag_2854 CATGCCGTCGGCCACAGGTGAAGCGT	4	7	1.015	6.996	6.35E-04	-2.78	no hit	-	-	-	-
-	Tag_2881 CATGCCCGGCTTCCCCGGCGGCGCTA	4	7	1.015	6.996	6.35E-04	-2.78	no hit	-	-	-	-
-	Tag_2886 CATGCTGCCCCCAACTGCCGCGCGCG	4	7	1.015	6.996	6.35E-04	-2.78	no hit	-	-	-	-
-	Tag_2943 CATGCTGCCCCCGACTGCCACGCGCG	4	7	1.015	6.996	6.35E-04	-2.78	no hit	-	-	-	-
Nucleic acid binding	Tag_2956 CATGCCGATGGTGGAGAAGACGTCTA	4	7	1.015	6.996	6.35E-04	-2.78	Hv_Contig_21394	685	512-537	EHJ78214|CCR4-NOT transcription complex subunit 2 *Danaus plexippus*	2.00E-147
Translation/Ribosome biogenesis	Tag_465 CATGGACAGACGCTAAGCTGACCGAA	119	207	30.198	206.867	7.66E-65	-2.78	Hv_Contig_27306	573	270-295	AAK92157|ribosomal protein L14 *Spodoptera frugiperda*	2.00E-85
Unknown	Tag_1861 CATGACCATCCGCCGCAATCCCCGCT	11	19	2.791	18.988	1.09E-07	-2.77	Hv_Contig_20304	714	390-415	EHJ74534|Bm8 interacting protein 2d-2 *Danaus plexippus*	1.00E-89
-	Tag_1240 CATGTGGTGGACTCCGTTGCTGAGCT	22	38	5.583	37.976	1.58E-13	-2.77	no hit	-	-	-	-
-	Tag_2220 CATGGGGCCGTGGCTGCGCCGATGCT	7	12	1.776	11.992	1.75E-05	-2.76	no hit	-	-	-	-
-	Tag_2275 CATGTGCGACGTTCTTTGTGACGTTA	7	12	1.776	11.992	1.75E-05	-2.76	no hit	-	-	-	-
-	Tag_1605 CATGGGGCTAGCCGACCGTGTGTGAA	14	24	3.553	23.985	3.36E-09	-2.76	no hit	-	-	-	-
-	Tag_1672 CATGGGCTTCGGTCTGTTCGACTAAA	13	22	3.299	21.986	1.58E-08	-2.74	no hit	-	-	-	-
-	Tag_1292 CATGGGCTTCGGTCTGTTTGACTAAG	21	35	5.329	34.978	2.31E-12	-2.71	no hit	-	-	-	-
-	Tag_2425 CATGGGGAAGAGGCTGCGCTGATGCC	6	10	1.523	9.994	8.53E-05	-2.71	no hit	-	-	-	-
-	Tag_2439 CATGGGACTCACCGAGTACGACACCG	6	10	1.523	9.994	8.53E-05	-2.71	no hit	-	-	-	-
-	Tag_2453 CATGTTCGTGGTGAAGGCGTACCTGT	6	10	1.523	9.994	8.53E-05	-2.71	no hit	-	-	-	-
Protein folding/Recycling	Tag_2466 CATGAAGCTGCAGCCGCGCAGCCCGC	6	10	1.523	9.994	8.53E-05	-2.71	Hv_Contig_34199	432	159-184	XP_013197280|E3 ubiquitin-protein ligase TRIM33 isoform X2 *Amyelois transitella*	4.00E-59
-	Tag_3030 CATGACGCCGCAAGACCAGCTCACGG	3	5	0.761	4.997	3.28E-03	-2.71	no hit	-	-	-	-
-	Tag_3041 CATGGCCGACTATCCGCAGCGCGTGC	3	5	0.761	4.997	3.28E-03	-2.71	no hit	-	-	-	-
-	Tag_3110 CATGTGCGCGCTAGCTCCCTGTGGGT	3	5	0.761	4.997	3.28E-03	-2.71	no hit	-	-	-	-
-	Tag_3117 CATGAGGACCAACAACTTCAAGGACT	3	5	0.761	4.997	3.28E-03	-2.71	no hit	-	-	-	-
-	Tag_3127 CATGCGCGATTCTCCTTGTCACCCTA	3	5	0.761	4.997	3.28E-03	-2.71	no hit	-	-	-	-
-	Tag_3131 CATGCGGGCGTGAGGCCTCTCACGTT	3	5	0.761	4.997	3.28E-03	-2.71	no hit	-	-	-	-
-	Tag_3135 CATGTCCATGCGCTCCAGCGACCGTA	3	5	0.761	4.997	3.28E-03	-2.71	no hit	-	-	-	-
-	Tag_3159 CATGAAGCGTGGCCACCACGCGCACG	3	5	0.761	4.997	3.28E-03	-2.71	no hit	-	-	-	-
-	Tag_3205 CATGCCGGGCCGCGCCGGTGCCGCCA	3	5	0.761	4.997	3.28E-03	-2.71	no hit	-	-	-	-
Transport/Trafficking	Tag_3208 CATGGGGCTCGACGCGCCGCCACACA	3	5	0.761	4.997	3.28E-03	-2.71	Hv_Contig_6580	1426	557-582	EHJ63133|staufen *Danaus plexippus*	5.00E-73
Primary metabolic process/hydrolase activity	Tag_3211 CATGGGCAACGGCATCATTTCAAAAC	3	5	0.761	4.997	3.28E-03	-2.71	Hv_Contig_13515	949	825-850	AFI64315|neutral lipase *Helicoverpa armigera*	0.00E+00
Unknown	Tag_3242 CATGTCGTCGTCGGAGCCGCGCGCGC	3	5	0.761	4.997	3.28E-03	-2.71	Hv_Contig_10241	1122	574-549	XP_013188445|uncharacterized protein LOC106133304 *Amyelois transitella*	3.00E-63
-	Tag_3259 CATGGGACCGCCGGAGCCCCGCAGCG	3	5	0.761	4.997	3.28E-03	-2.71	no hit	-	-	-	-
Iron homeostasis	Tag_3280 CATGTACTCCACTTTAGATTGTATCC	3	5	0.761	4.997	3.28E-03	-2.71	Hv_Contig_23698	630	436-461	ABM92425|ferritin HCH *Pieris rapae*	3.00E-18
-	Tag_3303 CATGCCCGGCTTCCCCGGCGGCGTGG	3	5	0.761	4.997	3.28E-03	-2.71	no hit	-	-	-	-
-	Tag_3338 CATGCCGTCGGCCACAGGTGATGCAG	3	5	0.761	4.997	3.28E-03	-2.71	no hit	-	-	-	-
-	Tag_3341 CATGTAGGAGCGTGCGGCGTGCTGCA	3	5	0.761	4.997	3.28E-03	-2.71	no hit	-	-	-	-
-	Tag_3346 CATGCCCCGGTGGCACTTTTTAGGGG	3	5	0.761	4.997	3.28E-03	-2.71	no hit	-	-	-	-

-	Tag_3378 CATGCCCGGCTTCCCCGGCGGTGCGG	3	5	0.761	4.997	3.28E-03	-2.71	no hit	-	-	-	-
-	Tag_3382 CATGCGCGCACCCACCGACACGTGCG	3	5	0.761	4.997	3.28E-03	-2.71	no hit	-	-	-	-
-	Tag_3389 CATGCCCGGCTTCCCCGGCAGCGCGA	3	5	0.761	4.997	3.28E-03	-2.71	no hit	-	-	-	-
-	Tag_3392 CATGTTCGCGGTGCTGGCGTTCACGC	3	5	0.761	4.997	3.28E-03	-2.71	no hit	-	-	-	-
-	Tag_3411 CATGGCGCAGTCCCCGCTGATGCAGG	3	5	0.761	4.997	3.28E-03	-2.71	no hit	-	-	-	-
Primary metabolic process/hydrolase activity	Tag_673 CATGCCGTACGCCAGGACGCTGGAGG	67	111	17.002	110.929	9.96E-35	-2.71	Hv_Contig_7410	1341	1155-1180	XP_012546310|pyruvate dehydrogenase E1 component beta subunit isoform X1 *Bombyx mori*	0.00E+00
-	Tag_1048 CATGGCCCCTGCGCAAGGATGACACG	29	48	7.359	47.969	3.61E-16	-2.70	no hit	-	-	-	-
-	Tag_372 CATGTGCGCGCTGGCTCCCTGTGGGC	164	270	41.618	269.827	5.90E-81	-2.70	no hit	-	-	-	-
-	Tag_1519 CATGGCCGACTATCCGCAGCGCGTAC	16	26	4.060	25.983	1.56E-09	-2.68	no hit	-	-	-	-
Transport/Trafficking	Tag_1197 CATGTTCGCGGTGCTGGCGTTCACGT	24	39	6.090	38.975	2.33E-13	-2.68	Hv_Contig_3824	1854	1582-1607	KOB56709|Organic cation transporter *Operophtera brumata*	0.00E+00
Unknown	Tag_2104 CATGAGCGTGATCGCTACTCCCGCCA	8	13	2.030	12.992	1.18E-05	-2.68	Hv_Contig_30047	526	143-168	XP_004922253|uncharacterized protein LOC101741939 *Bombyx mori*	6.00E-26
Primary metabolic process/oxidoreductase activity	Tag_2180 CATGGGCCCAACGTGGACCCATTGGA	8	13	2.030	12.992	1.18E-05	-2.68	Hv_Contig_10574	1102	581-606	AIN39485|POX-A *Spodoptera exigua*	9.00E-129
-	Tag_2521 CATGATCTGTGCCGGCTGGCCCAGAA	5	8	1.269	7.995	4.21E-04	-2.66	no hit	-	-	-	-
-	Tag_2549 CATGGCTCGCAGCCGCCCCCTGGCTC	5	8	1.269	7.995	4.21E-04	-2.66	no hit	-	-	-	-
-	Tag_2558 CATGCTGGACCCGGCGCAGGTGGCCG	5	8	1.269	7.995	4.21E-04	-2.66	no hit	-	-	-	-
-	Tag_2602 CATGCTGCAGCACGCGCTGAGCGTGG	5	8	1.269	7.995	4.21E-04	-2.66	no hit	-	-	-	-
Unknown	Tag_2606 CATGCGCGCGCACGGCGAGCGCGCGT	5	8	1.269	7.995	4.21E-04	-2.66	Hv_Contig_2899	2068	1962-1937	XP_012545191|myosin heavy chain, muscle *Bombyx mori*	0.00E+00
-	Tag_2707 CATGTTGAGTCTGGCGACCGCGCTGC	5	8	1.269	7.995	4.21E-04	-2.66	no hit	-	-	-	-
Nucleic acid binding	Tag_850 CATGTGGTCAGGCGCACGCGGCGGTG	42	67	10.658	66.957	2.77E-21	-2.65	Hv_Contig_822	3222	670-645	XP_013188738|myocyte-specific enhancer factor 2C isoform X1 *Amyelois transitella*	2.00E-125
-	Tag_2200 CATGGGACCACCTGTGAAGAACACTC	7	11	1.776	10.993	5.67E-05	-2.63	no hit	-	-	-	-
-	Tag_2223 CATGATTGTGTGAGCCTGTGCGGTCA	7	11	1.776	10.993	5.67E-05	-2.63	no hit	-	-	-	-
Primary metabolic process/hydratase activity	Tag_2232 CATGTCTCCCGTATATTGTAAGCGAA	7	11	1.776	10.993	5.67E-05	-2.63	Hv_Contig_21762	674	564-589	AID66689|enoyl-CoA hydratase *Agrotis segetum*	2.00E-82
-	Tag_2235 CATGATCTGAGCTCAAACCGGTGTAA	7	11	1.776	10.993	5.67E-05	-2.63	no hit	-	-	-	-
-	Tag_2269 CATGAACGGAACCGGCGCGTGGCCTT	7	11	1.776	10.993	5.67E-05	-2.63	no hit	-	-	-	-
-	Tag_2332 CATGGGCGGGCAGCAGCGCCGGCGCG	7	11	1.776	10.993	5.67E-05	-2.63	no hit	-	-	-	-
Primary metabolic process/oxidoreductase activity	Tag_1489 CATGGCCCTCGGCATCCGCTCACCCA	16	25	4.060	24.984	4.85E-09	-2.62	Hv_Contig_11352	1057	884-909	XP_013199964|NAD-dependent L-serine dehydrogenase *Amyelois transitella*	0.00E+00
-	Tag_1428 CATGATCTGTGCTGCCCAACTCGGTG	18	28	4.568	27.982	7.05E-10	-2.61	no hit	-	-	-	-
-	Tag_2057 CATGATTCCACACAAGACTGAGCGTG	9	14	2.284	13.991	7.83E-06	-2.61	no hit	-	-	-	-
-	Tag_891 CATGCGCGCAAGCGAGACGCGCGATA	38	59	9.643	58.962	1.15E-18	-2.61	no hit	-	-	-	-
-	Tag_1857 CATGAGGCCTCACAAGGGCAAGGCGA	11	17	2.791	16.989	1.10E-06	-2.61	no hit	-	-	-	-
-	Tag_1876 CATGTTGTGCTCTGGCTGGCCCACTC	11	17	2.791	16.989	1.10E-06	-2.61	no hit	-	-	-	-
-	Tag_709 CATGAGGCAGACGGTCGCTGTGGGGA	61	94	15.480	93.940	2.22E-28	-2.60	no hit	-	-	-	-
Primary metabolic process/transferase activity	Tag_614 CATGAGCTCAGACGTCGCGGCCTCAA	80	123	20.301	122.921	2.01E-36	-2.60	Hv_Contig_5987	1495	1288-1313	AGG55002|thiolase 4 *Heliothis virescens*	0.00E+00
Primary metabolic process/hydrolase activity	Tag_590 CATGTCGGCGCCGCTATGGGAGCTGC	85	128	21.570	127.918	2.78E-37	-2.57	Hv_Contig_20636	706	328-303	XP_013142996|carboxypeptidase B-like *Papilio polytes*	1.00E-101
-	Tag_2140 CATGAGACAGACGGTCGCTGTGGGTG	8	12	2.030	11.992	3.73E-05	-2.56	no hit	-	-	-	-
-	Tag_1773 CATGAAAGTGTACAAAGTCGGCGACG	12	18	3.045	17.988	7.29E-07	-2.56	no hit	-	-	-	-
-	Tag_2361 CATGTCCGTGCGCTCCGGCGACCGTA	6	9	1.523	8.994	2.76E-04	-2.56	no hit	-	-	-	-
-	Tag_2428 CATGGGACTCACCGGGTACGACGCCG	6	9	1.523	8.994	2.76E-04	-2.56	no hit	-	-	-	-
-	Tag_3433 CATGGAAATTTATTGTAAATGACAGA	2	3	0.508	2.998	1.77E-02	-2.56	no hit	-	-	-	-
-	Tag_3441 CATGAACCCCGTAGAGCGTCCTCACG	2	3	0.508	2.998	1.77E-02	-2.56	no hit	-	-	-	-
-	Tag_3446 CATGAATCAATCTGCAGCCAATCGTA	2	3	0.508	2.998	1.77E-02	-2.56	no hit	-	-	-	-
-	Tag_3449 CATGCCCACTGCCAAACAGAAGCTGC	2	3	0.508	2.998	1.77E-02	-2.56	no hit	-	-	-	-
-	Tag_3458 CATGTTGAAGCGCGGCCGCGGCGCAG	2	3	0.508	2.998	1.77E-02	-2.56	no hit	-	-	-	-
-	Tag_3470 CATGAGGCAGACGGTCGCTGGGGGTG	2	3	0.508	2.998	1.77E-02	-2.56	no hit	-	-	-	-
-	Tag_3472 CATGCTCGCCGCCAGCGCTGAGGAGG	2	3	0.508	2.998	1.77E-02	-2.56	no hit	-	-	-	-
-	Tag_3487 CATGCGCCTGTGCAGCGGCCTGGGCC	2	3	0.508	2.998	1.77E-02	-2.56	no hit	-	-	-	-
-	Tag_3489 CATGAGGCAGACGGTCGCAGTGGGTG	2	3	0.508	2.998	1.77E-02	-2.56	no hit	-	-	-	-
Transport/Trafficking	Tag_3511 CATGGCTGTTGCGGGCTGCAGCCTGA	2	3	0.508	2.998	1.77E-02	-2.56	Hv_Contig_9838	1149	772-797	XP_013198729|mitochondrial carrier homolog 2-like *Amyelois transitella*	0.00E+00
-	Tag_3521 CATGTGGTAGACTCCGTTGCTGAGCC	2	3	0.508	2.998	1.77E-02	-2.56	no hit	-	-	-	-
Signal transduction	Tag_3530 CATGTTTAAGCGCCTAGCAGTTGCCT	2	3	0.508	2.998	1.77E-02	-2.56	Hv_Contig_12290	1006	130-155	XP_004934372.2|multiple epidermal growth factor-like domains protein 11 *Bombyx mori*	3.00E-77
-	Tag_3533 CATGGGGGGGTTCTGCTCAGGACTGG	2	3	0.508	2.998	1.77E-02	-2.56	no hit	-	-	-	-
-	Tag_3553 CATGATCAACTTGCCTTCCAACGTAG	2	3	0.508	2.998	1.77E-02	-2.56	no hit	-	-	-	-
-	Tag_3560 CATGGCGGCCGCCTTCCGCAACCTGC	2	3	0.508	2.998	1.77E-02	-2.56	no hit	-	-	-	-
-	Tag_3562 CATGTGGCCGGGCTCGCATCAAAAAA	2	3	0.508	2.998	1.77E-02	-2.56	no hit	-	-	-	-
-	Tag_3587 CATGGCCCTGGCGGCGCTGGGCGTGT	2	3	0.508	2.998	1.77E-02	-2.56	no hit	-	-	-	-
-	Tag_3591 CATGAAAACCAACATTGTAAAAGCGA	2	3	0.508	2.998	1.77E-02	-2.56	no hit	-	-	-	-
-	Tag_3650 CATGCGGCCCGCGAACGTCTTACTCG	2	3	0.508	2.998	1.77E-02	-2.56	no hit	-	-	-	-
-	Tag_3658 CATGCCCGGCTTCCTCGGCGGCGCGT	2	3	0.508	2.998	1.77E-02	-2.56	no hit	-	-	-	-

-	Tag_3659 CATGTCAATCTGCGCATCGGCTGAAA	2	3	0.508	2.998	1.77E-02	-2.56	no hit	-	-	-	-
-	Tag_3670 CATGCCTGCTGCAAAAAACTGCTGGA	2	3	0.508	2.998	1.77E-02	-2.56	no hit	-	-	-	-
Primary metabolic process/oxidoreductase activity	Tag_3677 CATGTAAGCAACGACCGCAGCGGTGC	2	3	0.508	2.998	1.77E-02	-2.56	Hv_Contig_14534	906	706-731	NP_001106739|cytochrome b5 *Bombyx mori*	2.00E-31
-	Tag_3682 CATGCTGCCCCCGACTGCCGCGCGTT	2	3	0.508	2.998	1.77E-02	-2.56	no hit	-	-	-	-
Primary metabolic process/hydrolase activity	Tag_3696 CATGGCGATGGCTGCCGCAGCACAAG	2	3	0.508	2.998	1.77E-02	-2.56	Hv_Contig_3295	1965	1535-1560	KPJ16236|Retrovirus-related Pol polyprotein from transposon TNT 1-94 *Papilio machaon*	0.00E+00
Transport/Trafficking	Tag_3701 CATGCATTTTCTGTGGTACCTTACTT	2	3	0.508	2.998	1.77E-02	-2.56	Hv_Contig_977	3039	2836-2861	NP_001036846|nonclathrin coat protein gamma1-COP *Bombyx mori*	0.00E+00
-	Tag_3706 CATGATCTGAGTTCAAACCGATGTAA	2	3	0.508	2.998	1.77E-02	-2.56	no hit	-	-	-	-
-	Tag_3709 CATGTCTGTCTGTCTGTCCGTATGTC	2	3	0.508	2.998	1.77E-02	-2.56	no hit	-	-	-	-
-	Tag_3712 CATGTAAATAAGACCTGAATTGCATT	2	3	0.508	2.998	1.77E-02	-2.56	no hit	-	-	-	-
-	Tag_3719 CATGATCAACTTGCCTTCCAACGTGG	2	3	0.508	2.998	1.77E-02	-2.56	no hit	-	-	-	-
-	Tag_3726 CATGGCTCGCGGCCGCCCCCTGGTTC	2	3	0.508	2.998	1.77E-02	-2.56	no hit	-	-	-	-
Unknown	Tag_3727 CATGCGCCTCGAGCGGGGTCGAGGCG	2	3	0.508	2.998	1.77E-02	-2.56	Hv_Contig_10928	1081	400-425	XP_013133947|uncharacterized protein LOC106099826 *Papilio polytes*	4.00E-12
-	Tag_3750 CATGATTCCACACAAGACTGAGCGTA	2	3	0.508	2.998	1.77E-02	-2.56	no hit	-	-	-	-
-	Tag_3754 CATGAGGATTTCCCCCCGAAAAAAAA	2	3	0.508	2.998	1.77E-02	-2.56	no hit	-	-	-	-
-	Tag_3756 CATGCTGCCCCCTACTGCCGCGCGCG	2	3	0.508	2.998	1.77E-02	-2.56	no hit	-	-	-	-
-	Tag_3757 CATGTACGCGCTGGCTCCCTGTGGGT	2	3	0.508	2.998	1.77E-02	-2.56	no hit	-	-	-	-
-	Tag_3762 CATGGGGAGGTTCTGCTCAGGACTAC	2	3	0.508	2.998	1.77E-02	-2.56	no hit	-	-	-	-
Protein kinases	Tag_3763 CATGGAGCGCGCGTCGCGCGTGCAGC	2	3	0.508	2.998	1.77E-02	-2.56	Hv_Contig_8818	1221	925-950	XP_004923503|putative hexokinase HKDC1 *Bombyx mori*	5.00E-168
-	Tag_3776 CATGCCGCGGAGCGTGAAGTGGATGA	2	3	0.508	2.998	1.77E-02	-2.56	no hit	-	-	-	-
-	Tag_3780 CATGATGATGACGCGGAGGAAGATGA	2	3	0.508	2.998	1.77E-02	-2.56	no hit	-	-	-	-
-	Tag_3781 CATGCGCGCGCCACCGCTGCACTTCG	2	3	0.508	2.998	1.77E-02	-2.56	no hit	-	-	-	-
-	Tag_3790 CATGCCCGTACAGAAGCAGACCCGAA	2	3	0.508	2.998	1.77E-02	-2.56	no hit	-	-	-	-
-	Tag_3794 CATGCAGGGCGACGGCGAGTCCGGTC	2	3	0.508	2.998	1.77E-02	-2.56	no hit	-	-	-	-
-	Tag_3805 CATGAGGCTGCCGAGGCCCTTGACAA	2	3	0.508	2.998	1.77E-02	-2.56	no hit	-	-	-	-
Nucleic acid binding	Tag_3849 CATGCGCGAGTCCAACTCGCACTTAG	2	3	0.508	2.998	1.77E-02	-2.56	Hv_Contig_3299	1964	1888-1863	XP_013201214|repressor of RNA polymerase III transcription MAF1 homolog *Amyelois transitella*	1.00E-139
-	Tag_3859 CATGCGCGCACCGCCGCTGCACTTCG	2	3	0.508	2.998	1.77E-02	-2.56	no hit	-	-	-	-
-	Tag_3879 CATGTTGATCAGTCAATTAAATATTA	2	3	0.508	2.998	1.77E-02	-2.56	no hit	-	-	-	-
-	Tag_3880 CATGGCGGCCGCCTTCCGCAACCTAG	2	3	0.508	2.998	1.77E-02	-2.56	no hit	-	-	-	-
-	Tag_3893 CATGGCGCTCATCCGCAAGACCATCG	2	3	0.508	2.998	1.77E-02	-2.56	no hit	-	-	-	-
-	Tag_3924 CATGGGCTTCGGTCTGTTTGACTAGA	2	3	0.508	2.998	1.77E-02	-2.56	no hit	-	-	-	-
-	Tag_3929 CATGTGGGTTCCTTTGCCTCAAAACG	2	3	0.508	2.998	1.77E-02	-2.56	no hit	-	-	-	-
Transport/Trafficking	Tag_3949 CATGGTTGATATTTTCTGAATATATT	2	3	0.508	2.998	1.77E-02	-2.56	Hv_Contig_3328	1958	1895-1920	AFY62975|innexin 2 *Spodoptera litura*	0.00E+00
-	Tag_3953 CATGAGCGGGAGCCCTGGCCGTAATG	2	3	0.508	2.998	1.77E-02	-2.56	no hit	-	-	-	-
Protein folding/Recycling	Tag_3954 CATGCTCCCGATCAGGACGCCGCTTC	2	3	0.508	2.998	1.77E-02	-2.56	Hv_Contig_42221	266	117-142	XP_013188200|E3 ubiquitin-protein ligase RNF19B-like isoform X1 *Amyelois transitella*	6.00E-17
-	Tag_3955 CATGGAGCTCGGCGCGGGTCTCCCGA	2	3	0.508	2.998	1.77E-02	-2.56	no hit	-	-	-	-
-	Tag_3964 CATGTCCACCCGAGGCCGGGCGCGTC	2	3	0.508	2.998	1.77E-02	-2.56	no hit	-	-	-	-
-	Tag_3990 CATGAACCGCGGCAGCGGGCAGCACC	2	3	0.508	2.998	1.77E-02	-2.56	no hit	-	-	-	-
-	Tag_4005 CATGGACCGGCCGCGCCAAGCGTTGC	2	3	0.508	2.998	1.77E-02	-2.56	no hit	-	-	-	-
Nucleic acid binding	Tag_4008 CATGAACATAGCACCGAGATTAATGG	2	3	0.508	2.998	1.77E-02	-2.56	Hv_Contig_485	3787	3620-3645	XP_014361591|DNA-directed RNA polymerase II subunit RPB2 *Papilio machaon*	0.00E+00
-	Tag_4011 CATGGTTGGTGCGCGCGGGGGCCTTA	2	3	0.508	2.998	1.77E-02	-2.56	no hit	-	-	-	-
-	Tag_4017 CATGTGGTGGGCTCCGTTGCTGAGCC	2	3	0.508	2.998	1.77E-02	-2.56	no hit	-	-	-	-
-	Tag_4033 CATGGGACCAGCAAGGCAAGAACGGC	2	3	0.508	2.998	1.77E-02	-2.56	no hit	-	-	-	-
-	Tag_4035 CATGCTGTTGACTGCTGCAGCCACCG	2	3	0.508	2.998	1.77E-02	-2.56	no hit	-	-	-	-
-	Tag_1919 CATGCGCACGGGGCAGGCCGTGCAGA	10	15	2.538	14.990	5.18E-06	-2.56	no hit	-	-	-	-
-	Tag_2756 CATGTCGACTTGCCCGTTATCACAGG	4	6	1.015	5.996	2.12E-03	-2.56	no hit	-	-	-	-
-	Tag_2759 CATGGGACCAACAAGGCAAGAACGAC	4	6	1.015	5.996	2.12E-03	-2.56	no hit	-	-	-	-
-	Tag_2774 CATGATCTGTGCTGGTATCTTGGATG	4	6	1.015	5.996	2.12E-03	-2.56	no hit	-	-	-	-
-	Tag_2785 CATGCCCAGAAGCAACGCGATGTAAA	4	6	1.015	5.996	2.12E-03	-2.56	no hit	-	-	-	-
-	Tag_2818 CATGGTATGGACCTCACCACTGACAG	4	6	1.015	5.996	2.12E-03	-2.56	no hit	-	-	-	-
-	Tag_2870 CATGCGCAGTGCCCGCGTCGCGTGAG	4	6	1.015	5.996	2.12E-03	-2.56	no hit	-	-	-	-
-	Tag_2914 CATGAACCCCGTAGAGCATCCTCAAG	4	6	1.015	5.996	2.12E-03	-2.56	no hit	-	-	-	-
Calcium homeostasis	Tag_2991 CATGAAGGGACTGGGCACCAACGACA	4	6	1.015	5.996	2.12E-03	-2.56	Hv_Contig_12421	1000	668-693	CAI06089|putative annexin IX-B *Manduca sexta*	3.00E-136
-	Tag_3025 CATGGGTACGAGGTGGAAGGCCAATG	4	6	1.015	5.996	2.12E-03	-2.56	no hit	-	-	-	-
-	Tag_756 CATGCCCGTACAGAAGCAGACCCGTA	54	81	13.704	80.948	2.65E-24	-2.56	no hit	-	-	-	-
-	Tag_1628 CATGGAGGCTATCGCCGTCGCCCTCT	14	21	3.553	20.987	1.04E-07	-2.56	no hit	-	-	-	-
Primary metabolic process/oxidoreductase activity	Tag_66 CATGTGCGCGCTGGCTCCCTGTGGGT	1382	2071	350.708	2069.672	0.00E+00	-2.56	Hv_Contig_11894	1027	846-871	XP_013183095|aldehyde dehydrogenase X, mitochondrial-like *Amyelois transitella*	0.00E+00
Transport/Trafficking	Tag_171 CATGCTGTCGGTGCTGGGCTACGACC	405	606	102.776	605.611	3.36E-167	-2.56	Hv_Contig_885	3131	2996-3021	XP_013187665|potassium/sodium hyperpolarization-activated cyclic nucleotide-gated channel 2 isoform X3 *Amyelois transitella*	0.00E+00
-	Tag_635 CATGATCTGAGTTCAAACCGGTGTAC	77	114	19.540	113.927	4.74E-33	-2.54	no hit	-	-	-	-

-	Tag_1370 CATGCGCGCAAGCGAGACGCGCGATG	19	28	4.822	27.982	1.43E-09	-2.54	no hit	-	-	-	-
-	Tag_921 CATGAGCCCCCTCCTGCCCGACACCA	36	53	9.136	52.966	2.12E-16	-2.54	no hit	-	-	-	-
-	Tag_1461 CATGCCCGTACAGAAGCAGACCCGTC	17	25	4.314	24.984	9.90E-09	-2.53	no hit	-	-	-	-
Transport/Trafficking	Tag_1669 CATGTCCGCGAACGGGTCGGCGGCGA	13	19	3.299	18.988	4.81E-07	-2.52	Hv_Contig_11910	1026	54-79	XP_004923504|Na(+)/H(+) exchange regulatory cofactor NHE-RF1 *Bombyx mori*	3.00E-77
Unknown	Tag_1044 CATGGATTGACACCCCCATCGCTCCT	29	42	7.359	41.973	2.92E-13	-2.51	Hv_Contig_34661	421	257-282	XP_004922436|36.4 kDa proline-rich protein-like *Bombyx mori*	3.00E-10
Primary metabolic process/hydrolase activity	Tag_1975 CATGTTGGTATTTGCCAAGGCGACTC	9	13	2.284	12.992	2.44E-05	-2.51	Hv_Contig_12597	992	738-763	AFM28258|chymotrypsin *Heliothis virescens*	3.00E-152
-	Tag_1980 CATGCGCGCAAGCGAGACGCGCGAGC	9	13	2.284	12.992	2.44E-05	-2.51	no hit	-	-	-	-
-	Tag_876 CATGAGGCAGACGGTCGCTGTGGGCA	39	56	9.897	55.964	6.23E-17	-2.50	no hit	-	-	-	-
-	Tag_2209 CATGCTGTTAAGATGCGGATATTGTA	7	10	1.776	9.994	1.79E-04	-2.49	no hit	-	-	-	-
Protein folding/Recycling	Tag_2211 CATGTCCACGCGCCGCAAGCACCTGG	7	10	1.776	9.994	1.79E-04	-2.49	Hv_Contig_3728	1875	1389-1414	XP_013161276|kelch-like protein diablo *Papilio xuthus*	0.00E+00
-	Tag_2285 CATGGCTCTAGTCGGTGTCTGCAGGT	7	10	1.776	9.994	1.79E-04	-2.49	no hit	-	-	-	-
-	Tag_2317 CATGCGCCTGCCACCTCCTCCCGGGA	7	10	1.776	9.994	1.79E-04	-2.49	no hit	-	-	-	-
-	Tag_198 CATGAGGCAGACGGTCGCTGTGGGGG	334	472	84.759	471.697	2.35E-125	-2.48	no hit	-	-	-	-
-	Tag_2567 CATGATCTGGGTTCAAACCGGTGTAA	5	7	1.269	6.996	1.36E-03	-2.46	no hit	-	-	-	-
Nucleic acid binding	Tag_2618 CATGCACCCTAGTGTGATATTTGTAG	5	7	1.269	6.996	1.36E-03	-2.46	Hv_Contig_10710	1093	981-1006	KPJ18235|DNA-directed RNA polymerases I and III subunit RPAC1 *Papilio machaon*	0.00E+00
-	Tag_2689 CATGTCCCTCACGGACCAGTCTGCTA	5	7	1.269	6.996	1.36E-03	-2.46	no hit	-	-	-	-
-	Tag_2695 CATGCTCCAGTCCTCCTCGCTCACCA	5	7	1.269	6.996	1.36E-03	-2.46	no hit	-	-	-	-
Nucleic acid binding	Tag_2696 CATGAAACTGAACAACATACCCGTGG	5	7	1.269	6.996	1.36E-03	-2.46	Hv_Contig_22564	654	168-143	AEB54580|OBP1 *Helicoverpa armigera*	4.00E-56
-	Tag_718 CATGCTGGACCTCAAGATGAACCCTG	60	84	15.226	83.946	5.90E-24	-2.46	no hit	-	-	-	-
Nucleic acid binding	Tag_1531 CATGTGGGGAAACAGCACCGACGCAC	15	21	3.807	20.987	2.07E-07	-2.46	Hv_Contig_3610	1900	1162-1187	WP_047149320|RNA-directed DNA polymerase from mobile element jockey-like *Acropora digitifera*	1.00E-27
Unknown	Tag_1966 CATGTTCCCGATGCCGGTGGGAGACT	10	14	2.538	13.991	1.59E-05	-2.46	Hv_Contig_25599	598	503-528	KPJ20651|hypothetical protein RR46_00002 *Papilio xuthus*	3.00E-16
-	Tag_1287 CATGCTGCCCCCGACTGCCGCGCGCT	21	29	5.329	28.981	1.86E-09	-2.44	no hit	-	-	-	-
Translation/Ribosome biogenesis	Tag_220 CATGCCCGTACAGAAGCAGACCCGTG	298	410	75.623	409.737	5.43E-107	-2.44	Hv_Contig_29944	528	80-105	EHJ64657|ribsomal protein S2e *Danaus plexippus*	1.00E-77
Protein kinases	Tag_2143 CATGCGCAGTGCGCGCCGCGTGCCCG	8	11	2.030	10.993	1.16E-04	-2.44	Hv_Contig_19906	725	413-438	XP_012553210|epidermal growth factor receptor kinase substrate 8-like isoform X3 *Bombyx mori*	2.00E-65
mRNA processing/splicing	Tag_2154 CATGAAACCTGGAGCACTGCCACTGT	8	11	2.030	10.993	1.16E-04	-2.44	Hv_Contig_8635	1234	665-690	XP_013186601|corepressor interacting with RBPJ 1 *Amyelois transitella*	1.00E-139
Primary metabolic process/oxidoreductase activity	Tag_2156 CATGCGCAGCGGTGACGCCGGACGCG	8	11	2.030	10.993	1.16E-04	-2.44	Hv_Contig_5151	1620	1049-1074	AID66678|peroxisomal acyl-CoA oxidase 3 *Agrotis segetum*	0.00E+00
-	Tag_2161 CATGCTGCAGCACGCGCTGGGCGTAC	8	11	2.030	10.993	1.16E-04	-2.44	no hit	-	-	-	-
Unknown	Tag_2164 CATGCGAAGATCCTCACAGCTGCTAT	8	11	2.030	10.993	1.16E-04	-2.44	Hv_Contig_26036	591	179-154	XP_013193542|transmembrane protein 147 *Amyelois transitella*	9.00E-98
-	Tag_1179 CATGGCTCGCAGCCGCCCCCTGGTTT	24	33	6.090	32.979	1.78E-10	-2.44	no hit	-	-	-	-
Primary metabolic process/hydrolase activity	Tag_1866 CATGCCCAGCGCCGCCACCACCTTCA	11	15	2.791	14.990	1.03E-05	-2.42	Hv_Contig_6879	1394	661-636	XP_011568740|putative aminopeptidase W07G4.4 *Plutella xylostella*	5.00E-158
-	Tag_932 CATGCCTGGGTGTGCCACCGCGCTAG	36	49	9.136	48.969	1.58E-14	-2.42	no hit	-	-	-	-
-	Tag_1583 CATGATCTGAGTTCAAACCGGTGCAA	14	19	3.553	18.988	9.45E-07	-2.42	no hit	-	-	-	-
-	Tag_1594 CATGAAGATTGGCCGCATCGAGGAGG	14	19	3.553	18.988	9.45E-07	-2.42	no hit	-	-	-	-
-	Tag_1646 CATGGGCGGCGGGCACGGCGGCGTGA	14	19	3.553	18.988	9.45E-07	-2.42	no hit	-	-	-	-
-	Tag_1474 CATGGACCACCACCTGGAGGCCCCCA	17	23	4.314	22.985	8.82E-08	-2.41	no hit	-	-	-	-
-	Tag_1054 CATGCCCGGCTTCCCCGGCGGCGCAA	29	39	7.359	38.975	7.37E-12	-2.40	no hit	-	-	-	-
Translation/Ribosome biogenesis	Tag_863 CATGTCGCCCAGGTGTATGCTATCAG	41	55	10.405	54.965	6.69E-16	-2.40	Hv_Contig_11745	1035	302-327	KOB78727|Ribosomal protein S16 *Operophtera brumata*	6.00E-22
-	Tag_1988 CATGCTGTTGGCTGCTGCAGCCACCC	9	12	2.284	11.992	7.43E-05	-2.39	no hit	-	-	-	-
Transport/Trafficking	Tag_2016 CATGCCAAGGTCAACAACAAATCTCT	9	12	2.284	11.992	7.43E-05	-2.39	Hv_Contig_6787	1404	800-825	AEB26320|voltage-dependent anion-selective channel *Helicoverpa armigera*	0.00E+00
-	Tag_2024 CATGGGCCTGTGCCGCGGCCGCGAGC	9	12	2.284	11.992	7.43E-05	-2.39	no hit	-	-	-	-
-	Tag_2033 CATGGGCTTCGGTCTGTTCGACTAGG	9	12	2.284	11.992	7.43E-05	-2.39	no hit	-	-	-	-
-	Tag_2036 CATGCGCGCGCCGCCGCTGCACTTAG	9	12	2.284	11.992	7.43E-05	-2.39	no hit	-	-	-	-
Cytoskeleton	Tag_2062 CATGAAGGCCGAAGCAACGGCGGAGG	9	12	2.284	11.992	7.43E-05	-2.39	Hv_Contig_6331	1454	248-273	XP_013196505|kinetochore null protein 1 *Amyelois transitella*	2.00E-82
Primary metabolic process/hydrolase activity	Tag_2443 CATGAATACAAAGCTGAAGATGACGC	6	8	1.523	7.995	8.64E-04	-2.39	Hv_Contig_37459	337	210-235	AEA76313|astacin *Mamestra configurata*	1.00E-41
-	Tag_2475 CATGGCCGCTCCAGGGGCGACAACCC	6	8	1.523	7.995	8.64E-04	-2.39	no hit	-	-	-	-
unknown	Tag_2484 CATGAAGCTCGCGGCCACTTGAACTA	6	8	1.523	7.995	8.64E-04	-2.39	Hv_Contig_2646	2140	99-74	XP_013190958|uncharacterized protein LOC106135256 *Amyelois transitella*	0.00E+00
-	Tag_3032 CATGGGACTCACCGAGTACGACGCTC	3	4	0.761	3.997	1.10E-02	-2.39	no hit	-	-	-	-
Calcium homeostasis	Tag_3040 CATGGTGCCGGGCTCGCGGCCGCCGC	3	4	0.761	3.997	1.10E-02	-2.39	Hv_Contig_209	4645	527-502	XP_013174707|plasma membrane calcium-transporting ATPase 3 isoform X4 *Papilio xuthus*	0.00E+00
-	Tag_3042 CATGAGCCCGTGGTCCACGTGGGATG	3	4	0.761	3.997	1.10E-02	-2.39	no hit	-	-	-	-
-	Tag_3054 CATGAGAAACGTTGAAGTGTGGACAC	3	4	0.761	3.997	1.10E-02	-2.39	no hit	-	-	-	-
-	Tag_3069 CATGTCCGTGCGCCCCAGCGACCGTA	3	4	0.761	3.997	1.10E-02	-2.39	no hit	-	-	-	-
-	Tag_3081 CATGACACGTCGCGGCGCTCTGTTAG	3	4	0.761	3.997	1.10E-02	-2.39	no hit	-	-	-	-
-	Tag_3086 CATGGAGCGGCCGCAAGGGAATCTAT	3	4	0.761	3.997	1.10E-02	-2.39	no hit	-	-	-	-
-	Tag_3088 CATGCGCCTTCACCCCGCACGAGACA	3	4	0.761	3.997	1.10E-02	-2.39	no hit	-	-	-	-
-	Tag_3090 CATGCCCAGCGCGCTGGTGGCTGCAG	3	4	0.761	3.997	1.10E-02	-2.39	no hit	-	-	-	-
Protein folding/Recycling	Tag_3118 CATGAATTTGTTGTAGGACTATCGTT	3	4	0.761	3.997	1.10E-02	-2.39	Hv_Contig_2236	2297	2077-2102	EHJ76767|putative seven in absentia *Danaus plexippus*	0.00E+00
-	Tag_3149 CATGGAGTGCGGGCTGCGCTTCGACA	3	4	0.761	3.997	1.10E-02	-2.39	no hit	-	-	-	-
-	Tag_3183 CATGGGACTCACCGAGTACAACGCCG	3	4	0.761	3.997	1.10E-02	-2.39	no hit	-	-	-	-

Signal transduction	Tag_3184 CATGTCGGCCGGGGCGCGCGCCTGTC	3	4	0.761	3.997	1.10E-02	-2.39	Hv_Contig_2386	2235	5_30	AAD09810|nicotinic acetylcholine receptor beta-1 subunit *Heliothis virescens*	0.00E+00
-	Tag_3190 CATGTGGTCAGGCGCACGCGGCGGTC	3	4	0.761	3.997	1.10E-02	-2.39	no hit	-	-	-	-
-	Tag_3192 CATGCCCAGCTTCCCCGGCGGCGCGA	3	4	0.761	3.997	1.10E-02	-2.39	no hit	-	-	-	-
-	Tag_3193 CATGTCCGGCATCGTGCAGAGCGTCT	3	4	0.761	3.997	1.10E-02	-2.39	no hit	-	-	-	-
Primary metabolic process/hydrolase activity	Tag_3195 CATGGACATCGGCCGCGCCACCGTCA	3	4	0.761	3.997	1.10E-02	-2.39	Hv_Contig_4065	1807	847-872	KPJ11631|Fructose-bisphosphate aldolase *Papilio machaon*	0.00E+00
-	Tag_3215 CATGTCGGCGCCGCTATGGGAGCTGT	3	4	0.761	3.997	1.10E-02	-2.39	no hit	-	-	-	-
-	Tag_3224 CATGCTGGACCTCAAGATGAACCCGG	3	4	0.761	3.997	1.10E-02	-2.39	no hit	-	-	-	-
Unknown	Tag_3249 CATGGTGGCGCGCGCGCGCGTGCGCG	3	4	0.761	3.997	1.10E-02	-2.39	Hv_Contig_6085	1484	258-233	XP_004924047|fasciclin-1 isoform X2 *Bombyx mori*	0.00E+00
-	Tag_3253 CATGGGACACAGGATACGCGTGATAG	3	4	0.761	3.997	1.10E-02	-2.39	no hit	-	-	-	-
-	Tag_3256 CATGATTGTGTGAGCCCGTGCGGCCA	3	4	0.761	3.997	1.10E-02	-2.39	no hit	-	-	-	-
-	Tag_3275 CATGCCCGGCTTCCCCGGCGGCGTAC	3	4	0.761	3.997	1.10E-02	-2.39	no hit	-	-	-	-
-	Tag_3278 CATGAGATAGGGTTGTCGCTGTCGCC	3	4	0.761	3.997	1.10E-02	-2.39	no hit	-	-	-	-
Translation/Ribosome biogenesis	Tag_3307 CATGGAGGCGGTGGCTTTGTCCAGTT	3	4	0.761	3.997	1.10E-02	-2.39	Hv_Contig_14324	915	132-107	NP_001299429|nascent polypeptide-associated complex subunit alpha *Papilio xuthus*	1.00E-100
-	Tag_3312 CATGTTGTGCTCTGGCTGGCCCACAG	3	4	0.761	3.997	1.10E-02	-2.39	no hit	-	-	-	-
-	Tag_3320 CATGTTGTGCTCTGGCTGGCCCACTT	3	4	0.761	3.997	1.10E-02	-2.39	no hit	-	-	-	-
Protein folding/Recycling	Tag_3325 CATGTCCGTGACGCGGCCGCGCCGCA	3	4	0.761	3.997	1.10E-02	-2.39	Hv_Contig_21577	679	40-15	NP_001040107|chaperonin subunit 4 delta *Bombyx mori*	1.00E-18
unknown	Tag_3331 CATGCGCCGGTCGTCGGCCTGGCTGC	3	4	0.761	3.997	1.10E-02	-2.39	Hv_Contig_29171	542	180-155	XP_013196541|uncharacterized protein LOC106139608 isoform X4 *Amyelois transitella*	5.00E-61
Primary metabolic process/oxidoreductase activity	Tag_3345 CATGGAATCCTATAGAAGGGAGATAT	3	4	0.761	3.997	1.10E-02	-2.39	Hv_Contig_21233	689	215-240	KPJ14522|Chorion peroxidase *Papilio machaon*	7.00E-70
-	Tag_3351 CATGTCGGCGGGCCGCGAGGACGTTG	3	4	0.761	3.997	1.10E-02	-2.39	no hit	-	-	-	-
-	Tag_3365 CATGTTCGCGGTGCTGGCGTTCACGG	3	4	0.761	3.997	1.10E-02	-2.39	no hit	-	-	-	-
unknown	Tag_3368 CATGATGTAATGCGAATTTTGATAGG	3	4	0.761	3.997	1.10E-02	-2.39	Hv_Contig_2815	2091	1846-1871	XP_013185347|uncharacterized protein LOC106130942 *Amyelois transitella*	2.00E-179
-	Tag_3376 CATGACTCGTACGAGCTGCGCTACGC	3	4	0.761	3.997	1.10E-02	-2.39	no hit	-	-	-	-
Protein kinases	Tag_3379 CATGCTGGAGTACGAGCCCGCCCAGC	3	4	0.761	3.997	1.10E-02	-2.39	Hv_Contig_7110	1371	1139-1164	XP_013195225|serine/threonine-protein kinase PRP4 homolog isoform X2 *Amyelois transitella*	0.00E+00
-	Tag_3386 CATGGGGCAGACGGTCGCTGTGGGTA	3	4	0.761	3.997	1.10E-02	-2.39	no hit	-	-	-	-
Protein kinases	Tag_3393 CATGAGGAGGCTGAACCTCAACCCTG	3	4	0.761	3.997	1.10E-02	-2.39	Hv_Contig_5310	1594	984-1009	ALL42054|ecdysteroid 22-kinase *Antheraea pernyi*	3.00E-108
-	Tag_3405 CATGTTAATCAGCAAACTAGTTTTAG	3	4	0.761	3.997	1.10E-02	-2.39	no hit	-	-	-	-
-	Tag_289 CATGATTGTGTGAGCCTGTGCGGCCG	218	289	55.322	288.815	6.15E-74	-2.38	no hit	-	-	-	-
-	Tag_1079 CATGTCCGTGCGCTCCAGCGACCGAG	28	37	7.106	36.976	3.25E-11	-2.38	no hit	-	-	-	-
Translation/Ribosome biogenesis	Tag_770 CATGAAAGTGTACAAAGTCGGCGACA	53	70	13.450	69.955	1.80E-19	-2.38	Hv_Contig_13554	947	281-306	AAV32453|ribosomal protein L21 *Helicoverpa zea*	2.00E-107
-	Tag_1694 CATGCCCGGATTCCCCGGCGGCGCGC	13	17	3.299	16.989	4.30E-06	-2.36	no hit	-	-	-	-
-	Tag_1699 CATGAGGCTGCCAAGGCCCTTGACAT	13	17	3.299	16.989	4.30E-06	-2.36	no hit	-	-	-	-
-	Tag_738 CATGAGCGGGATCTCTCTCAGCCCCG	56	73	14.211	72.953	5.05E-20	-2.36	no hit	-	-	-	-
Protein folding/Recycling	Tag_1925 CATGCGTCCCGGCCGTCTCGACCGTA	10	13	2.538	12.992	4.76E-05	-2.36	Hv_Contig_14477	908	345-370	XP_011564450|26S protease regulatory subunit 7-like *Plutella xylostella*	6.00E-135
-	Tag_1449 CATGTCGGTCGGGGGCGGCAACTCAG	17	22	4.314	21.986	2.57E-07	-2.35	no hit	-	-	-	-
-	Tag_1078 CATGATCGTCCGAGACACGCCCCCCT	28	36	7.106	35.977	9.31E-11	-2.34	no hit	-	-	-	-
-	Tag_1654 CATGCTGGCGGCGCGCTACTCGCCGT	14	18	3.553	17.988	2.77E-06	-2.34	no hit	-	-	-	-
-	Tag_2244 CATGCCCGACTTCCCCGGCGGCGCGA	7	9	1.776	8.994	5.48E-04	-2.34	no hit	-	-	-	-
-	Tag_2261 CATGTCGACTTGCCCGTTATCACAAC	7	9	1.776	8.994	5.48E-04	-2.34	no hit	-	-	-	-
-	Tag_2265 CATGCTGGACCTCAAGATGAACCCTA	7	9	1.776	8.994	5.48E-04	-2.34	no hit	-	-	-	-
-	Tag_2303 CATGTCCGCGCGCTCCAGCGACCGTA	7	9	1.776	8.994	5.48E-04	-2.34	no hit	-	-	-	-
-	Tag_1788 CATGCCCGGCTTCCCCGGTGGCGCGA	11	14	2.791	13.991	3.05E-05	-2.33	no hit	-	-	-	-
-	Tag_1860 CATGAGGCAGATGGTCGCTGTGGGTG	11	14	2.791	13.991	3.05E-05	-2.33	no hit	-	-	-	-
-	Tag_806 CATGGCTCGCAGCCGCCCCCTGGTTA	48	61	12.181	60.961	9.64E-17	-2.32	no hit	-	-	-	-
Nucleic acid binding	Tag_630 CATGGCTCGTGGTCACCTTGCTGCCA	77	97	19.540	96.938	2.98E-25	-2.31	Hv_Contig_6429	1443	821-846	CAR92522|putative dsRNase *Spodoptera littoralis*	0.00E+00
-	Tag_2129 CATGCCGCGGAGCGTGAAGTGGATAA	8	10	2.030	9.994	3.47E-04	-2.30	no hit	-	-	-	-
-	Tag_2148 CATGAATGCCGCTACGCTGATTTCTT	8	10	2.030	9.994	3.47E-04	-2.30	no hit	-	-	-	-
-	Tag_2170 CATGCGGCCCGCGAACGTCTTACTTG	8	10	2.030	9.994	3.47E-04	-2.30	no hit	-	-	-	-
-	Tag_2187 CATGTTGTGCTCTGGCTGGCCCACCG	8	10	2.030	9.994	3.47E-04	-2.30	no hit	-	-	-	-
-	Tag_2188 CATGGGTACGAGGTGGAAGGCCAAGC	8	10	2.030	9.994	3.47E-04	-2.30	no hit	-	-	-	-
Primary metabolic process/oxidoreductase activity	Tag_1740 CATGGTGAGGAACCACTCCCACGGTG	12	15	3.045	14.990	1.95E-05	-2.30	Hv_Contig_11027	959	699-724	XP_004933259|cytochrome c oxidase subunit 7C, mitochondrial-like *Bombyx mori*	2.00E-29
-	Tag_1747 CATGATCTGAGTTCGAACCGGTGTAA	12	15	3.045	14.990	1.95E-05	-2.30	no hit	-	-	-	-
-	Tag_2727 CATGCCCGGCTTCCCCGGCGGCTCGC	4	5	1.015	4.997	6.79E-03	-2.30	no hit	-	-	-	-
-	Tag_2729 CATGGCGGCCGCTGCTGACGTCCTCG	4	5	1.015	4.997	6.79E-03	-2.30	no hit	-	-	-	-
-	Tag_2741 CATGCCCGGCTTCCCCGGCGGCACGA	4	5	1.015	4.997	6.79E-03	-2.30	no hit	-	-	-	-
-	Tag_2777 CATGCGCCTGTGCGGCGGCCCGGGCC	4	5	1.015	4.997	6.79E-03	-2.30	no hit	-	-	-	-
Transport/Trafficking	Tag_2810 CATGGCACATTGTACCGGCACCGCTC	4	5	1.015	4.997	6.79E-03	-2.30	Hv_Contig_34630	421	46-71	AII21955|fatty acid transport protein 4 *Sesamia inferens*	8.00E-25
-	Tag_2817 CATGATCGTGTGAGCCTGTGCGGCCA	4	5	1.015	4.997	6.79E-03	-2.30	no hit	-	-	-	-
Translation/Ribosome biogenesis	Tag_2857 CATGGTGATGTTTTGTTTAATGTTAG	4	5	1.015	4.997	6.79E-03	-2.30	Hv_Contig_5962	1499	1432-1457	XP_013186962|complement component 1 Q subcomponent-binding protein, mitochondrial *Amyelois transitella*	5.00E-159

-	Tag_2873 CATGCTGCCCCCGACTGCTGCGCGCG	4	5	1.015	4.997	6.79E-03	-2.30	no hit	-	-	-	-
-	Tag_2875 CATGGGGTCGTGGCTGCGCTGATGCT	4	5	1.015	4.997	6.79E-03	-2.30	no hit	-	-	-	-
-	Tag_2898 CATGTGCGCGCTGGTTCCCTGTGGGT	4	5	1.015	4.997	6.79E-03	-2.30	no hit	-	-	-	-
-	Tag_2903 CATGGTGGTGGAGGTGCCGCGCTGAA	4	5	1.015	4.997	6.79E-03	-2.30	no hit	-	-	-	-
-	Tag_2922 CATGATCTGAGGTCAAACCGGTGTAG	4	5	1.015	4.997	6.79E-03	-2.30	no hit	-	-	-	-
-	Tag_2934 CATGAGGAGACAGGGAGTCGTTGTAA	4	5	1.015	4.997	6.79E-03	-2.30	no hit	-	-	-	-
-	Tag_2966 CATGACAATTTGTTCGCATTCATACC	4	5	1.015	4.997	6.79E-03	-2.30	no hit	-	-	-	-
Translation/Ribosome biogenesis	Tag_2979 CATGGGGCAGGCTGTTGCTCGGGGCC	4	5	1.015	4.997	6.79E-03	-2.30	Hv_Contig_16230	839	200-175	ABX54737|elongation factor 1 beta *Spodoptera exigua*	3.00E-114
-	Tag_2997 CATGTTCCGTTTTCCACATTCCGATC	4	5	1.015	4.997	6.79E-03	-2.30	no hit	-	-	-	-
Translation/Ribosome biogenesis	Tag_1517 CATGCTCCCAAACATCCCGATCTGGA	16	20	4.060	19.987	1.15E-06	-2.30	Hv_Contig_33229	456	41-66	XP_013185598|40S ribosomal protein S10 *Amyelois transitella*	9.00E-69
-	Tag_705 CATGCGGCGCTGGCTCAGCCGCCACC	61	76	15.480	75.951	4.67E-20	-2.29	no hit	-	-	-	-
Primary metabolic process/oxidoreductase activity	Tag_1656 CATGGAAAATATTATTCAGGTATTTA	13	16	3.299	15.990	1.25E-05	-2.28	Hv_Contig_8452	1247	1097-1122	YP_009183760|NADH dehydrogenase subunit 4 (mitochondrion) *Heliothis subflexa*	1.00E-134
Calcium homeostasis	Tag_2002 CATGGTGCCCGTCGACAGAAGCAGTG	9	11	2.284	10.993	2.20E-04	-2.27	Hv_Contig_390	4006	3708-3733	AAD09820|sarco(endo)plasmic reticulum-type calcium ATPase *Heliothis virescens*	0.00E+00
Signal transduction	Tag_2012 CATGCCAAATGCCATATCCACACACG	9	11	2.284	10.993	2.20E-04	-2.27	Hv_Contig_25900	593	431-456	KOB77539|Tryptophan-rich protein *Operophtera brumata*	5.00E-81
-	Tag_975 CATGATCTGAGTTCAAACCGGTGTGC	33	40	8.374	39.974	2.97E-11	-2.26	no hit	-	-	-	-
-	Tag_83 CATGATCTGAGTTCAAACCGGTGTAG	985	1193	249.962	1192.235	1.03E-275	-2.25	no hit	-	-	-	-
-	Tag_1357 CATGGGCCCGCGCCGGTACTAGCGGT	19	23	4.822	22.985	3.03E-07	-2.25	no hit	-	-	-	-
-	Tag_2528 CATGTCCGTGCGCTCCAGCGACTGTA	5	6	1.269	5.996	4.20E-03	-2.24	no hit	-	-	-	-
Primary metabolic process/hydrolase activity	Tag_2548 CATGCAGCCCACGCAGCACCTGCTCA	5	6	1.269	5.996	4.20E-03	-2.24	Hv_Contig_1503	2634	1926-1901	XP_013140832|serine/threonine-protein phosphatase 2A catalytic subunit beta isoform isoform X1 *Papilio polytes*	0.00E+00
Translation/Ribosome biogenesis	Tag_2579 CATGCCCAAGCTGAAGGCATTCCAGA	5	6	1.269	5.996	4.20E-03	-2.24	Hv_Contig_21293	687	455-430	Q962S0|40S ribosomal protein S7 *Spodoptera frugiperda*	1.00E-131
-	Tag_2592 CATGCGCCGCCACCACACCACGCGCG	5	6	1.269	5.996	4.20E-03	-2.24	no hit	-	-	-	-
-	Tag_2594 CATGCCGCGGAGCGTGAAGTGGATGG	5	6	1.269	5.996	4.20E-03	-2.24	no hit	-	-	-	-
Transport/Trafficking	Tag_2604 CATGTCCACTTGCAATCTAACAGATA	5	6	1.269	5.996	4.20E-03	-2.24	Hv_Contig_4269	1769	1590-1615	XP_001845188|V-type ATP synthase beta chain *Culex quinquefasciatus*	4.00E-90
Protein folding/Recycling	Tag_2610 CATGGGGTTCCCGGCTGATGCTGGCG	5	6	1.269	5.996	4.20E-03	-2.24	Hv_Contig_3541	1913	1835-1860	XP_013194217|protein sel-1 homolog 1 *Amyelois transitella*	0.00E+00
-	Tag_2656 CATGAGGCAGACGGTCGTTGTGGGTG	5	6	1.269	5.996	4.20E-03	-2.24	no hit	-	-	-	-
-	Tag_2660 CATGAGGCAGACGGTCGCTGTGGATA	5	6	1.269	5.996	4.20E-03	-2.24	no hit	-	-	-	-
-	Tag_2669 CATGTGCGCGCCGGCTCCCTGTGGGT	5	6	1.269	5.996	4.20E-03	-2.24	no hit	-	-	-	-
Unknown	Tag_2670 CATGGGACACCATGAAAGGCGTTGGT	5	6	1.269	5.996	4.20E-03	-2.24	Hv_Contig_37821	346	168-143	XP_001624571|predicted protein *Nematostella vectensis*	1.00E-17
-	Tag_1883 CATGCCCGTACAGAAGCAGACCCGTT	10	12	2.538	11.992	1.39E-04	-2.24	no hit	-	-	-	-
Nucleic acid binding	Tag_1579 CATGTACTTCGCGTTGCCGGCTGTTC	15	18	3.807	17.988	5.08E-06	-2.24	Hv_Contig_31984	484	217-242	BAA34219|MBF2 *Samia cynthia*	1.00E-52
-	Tag_922 CATGCCCGGCTTCCCCGGCGGCGCAT	36	43	9.136	42.972	7.99E-12	-2.23	no hit	-	-	-	-
-	Tag_995 CATGATTGTGTGAGCCTGTGCGGCCT	32	38	8.121	37.976	1.27E-10	-2.23	no hit	-	-	-	-
Nucleic acid binding	Tag_1840 CATGATCGACGGGCGCTGGTGCGACG	11	13	2.791	12.992	8.80E-05	-2.22	Hv_Contig_12397	977	621-646	XP_013188449|TAR DNA-binding protein 43-like *Amyelois transitella*	2.00E-110
-	Tag_1842 CATGTTGAGTCTGGCGACCGCGCCAG	11	13	2.791	12.992	8.80E-05	-2.22	no hit	-	-	-	-
-	Tag_524 CATGATCGTCCGAGACACGCCCCCCG	102	120	25.884	119.923	4.63E-29	-2.21	no hit	-	-	-	-
Primary metabolic process/transferase activity	Tag_1210 CATGATCCGCGTACGAAGTCGCGGGC	23	27	5.837	26.983	5.13E-08	-2.21	Hv_Contig_281	4352	2130-2105	AGG36456|dolichyl-diphosphooligosaccharide-glycosyltransferase 1 *Chilo suppressalis*	0.00E+00
Cytoskeleton	Tag_1181 CATGGGCGACTATCGTGCGGGCAAGG	24	28	6.090	27.982	3.29E-08	-2.20	Hv_Contig_19329	744	112-137	ADM47613|calponin *Helicoverpa armigera*	1.00E-129
Calcium homeostasis	Tag_2346 CATGCTCAAAAACCTTCGTTGTGAAT	6	7	1.523	6.996	2.61E-03	-2.20	Hv_Contig_1880	2434	2328-2353	XP_011548273|calcium-transporting ATPase sarcoplasmic/endoplasmic reticulum type isoform X1 *Plutella xylostella*	6.00E-11
-	Tag_2384 CATGCCTGGGTGTGCCACCGCGCTAA	6	7	1.523	6.996	2.61E-03	-2.20	no hit	-	-	-	-
Primary metabolic process/oxidoreductase activity	Tag_2433 CATGTCTAGTTGCTGGTTGACTATAC	6	7	1.523	6.996	2.61E-03	-2.20	Hv_Contig_13838	935	500-525	AGC39043|thioredoxin *Helicoverpa armigera*	2.00E-64
-	Tag_2435 CATGGCAGTAGCGGCGAGGCCGCGCA	6	7	1.523	6.996	2.61E-03	-2.20	no hit	-	-	-	-
-	Tag_2471 CATGCCCATCCCTGCCGGAGCTGTGG	6	7	1.523	6.996	2.61E-03	-2.20	no hit	-	-	-	-
-	Tag_2485 CATGCTGCAACACGCGCTGGGCGTGG	6	7	1.523	6.996	2.61E-03	-2.20	no hit	-	-	-	-
-	Tag_1396 CATGAGGCAGACGGTCACTGTGGGTG	18	21	4.568	20.987	1.32E-06	-2.20	no hit	-	-	-	-
-	Tag_990 CATGCACCGCGGTGGGGCCGCGCCTT	32	37	8.121	36.976	3.46E-10	-2.19	no hit	-	-	-	-
-	Tag_1130 CATGAGGCAGACGGTCGCTGTGGGGT	26	30	6.598	29.981	1.35E-08	-2.18	no hit	-	-	-	-
Unknown	Tag_1660 CATGACTGGCTCCAGCATCATCAACA	13	15	3.299	14.990	3.53E-05	-2.18	Hv_Contig_22264	662	416-441	XP_013193209|uncharacterized protein LOC106137022 isoform X1 *Amyelois transitella*	2.00E-51
Translation/Ribosome biogenesis	Tag_99 CATGATTGTGTGAGCCTGTGCGGCCA	781	900	198.193	899.423	1.67E-199	-2.18	Hv_Contig_6221	1468	1410-1435	NP_001298516|elongation factor 1-gamma *Papilio polytes*	0.00E+00
-	Tag_1596 CATGGCTCGCGGACCACTTGAGGTGC	14	16	3.553	15.990	2.24E-05	-2.17	no hit	-	-	-	-
-	Tag_1631 CATGTTGCTTCGCTGTGACCGCCGTG	14	16	3.553	15.990	2.24E-05	-2.17	no hit	-	-	-	-
-	Tag_1641 CATGCAGTCGCTGCGCGTTTGGTCGG	14	16	3.553	15.990	2.24E-05	-2.17	no hit	-	-	-	-
-	Tag_2198 CATGCTGCAGCACGCGCTGGGTGTGG	7	8	1.776	7.995	1.62E-03	-2.17	no hit	-	-	-	-
-	Tag_2245 CATGGCGGACGTTGAGCTCGTGTCGG	7	8	1.776	7.995	1.62E-03	-2.17	no hit	-	-	-	-
-	Tag_2249 CATGCTGGCGGGACGGGCGCTGCTGC	7	8	1.776	7.995	1.62E-03	-2.17	no hit	-	-	-	-
-	Tag_2294 CATGGGCGGCGGGCACGGCGGCGTAG	7	8	1.776	7.995	1.62E-03	-2.17	no hit	-	-	-	-
Translation/Ribosome biogenesis	Tag_1245 CATGAGTGCGGAATCTGCGGGCGAAC	22	25	5.583	24.984	2.20E-07	-2.16	Hv_Contig_26047	591	131-106	AAV32453|ribosomal protein L21 *Helicoverpa zea*	3.00E-109
-	Tag_369 CATGATCTGAGTTCAAACCGGTGTGG	164	186	41.618	185.881	1.82E-42	-2.16	no hit	-	-	-	-
-	Tag_1562 CATGTGGTGCGACAGGACCCCACCGG	15	17	3.807	16.989	1.42E-05	-2.16	no hit	-	-	-	-

-	Tag_2074 CATGTCCGTGCGTTCCAGCGACCGTG	8	9	2.030	8.994	1.01E-03	-2.15	no hit	-	-	-	-
-	Tag_2117 CATGCGTCGGCCGGGAGGCGCTCAGG	8	9	2.030	8.994	1.01E-03	-2.15	no hit	-	-	-	-
-	Tag_2181 CATGCGCGCGCTGGCTCCCTGTGGGT	8	9	2.030	8.994	1.01E-03	-2.15	no hit	-	-	-	-
Nucleic acid binding	Tag_1470 CATGGGCACTGTTGCGAGATGCCTTC	17	19	4.314	18.988	5.71E-06	-2.14	Hv_Contig_19186	747	713-688	XP_013180494|transcription factor Adf-1-like *Papilio xuthus*	7.00E-42
Primary metabolic process/oxidoreductase activity	Tag_936 CATGCTAATGGAGCATCATTTTTTTT	35	39	8.882	38.975	2.46E-10	-2.13	Hv_Contig_18394	769	75-100	YP_009183763|cytochrome b (mitochondrion) *Heliothis subflexa*	8.00E-77
-	Tag_2039 CATGGCTCGCAGCCGCCCCCTGGTAC	9	10	2.284	9.994	6.30E-04	-2.13	no hit	-	-	-	-
-	Tag_1088 CATGGCTCGCAGCCGCCCCCTGGTCC	27	30	6.852	29.981	2.33E-08	-2.13	no hit	-	-	-	-
Unknown	Tag_919 CATGCATCCGCTTCAGCTTCACCGGC	36	40	9.136	39.974	1.58E-10	-2.13	Hv_Contig_23342	637	224-249	XP_012146604|uncharacterized protein LOC105663301 *Megachile rotundata*	1.00E-14
-	Tag_1912 CATGAGGCTGCCAAGGCCCTTGACGG	10	11	2.538	10.993	3.94E-04	-2.11	no hit	-	-	-	-
-	Tag_1928 CATGCACCGCGGTGGGGCCGCGCCTG	10	11	2.538	10.993	3.94E-04	-2.11	no hit	-	-	-	-
-	Tag_749 CATGCTCACCGCCAGCGCTGAGGAGA	55	60	13.957	59.962	1.32E-14	-2.10	no hit	-	-	-	-
-	Tag_1822 CATGGGTACGAGGTGGAAGGCCAATA	11	12	2.791	11.992	2.47E-04	-2.10	no hit	-	-	-	-
-	Tag_1846 CATGTCAAAGCTGCGCCCTTGCTCCG	11	12	2.791	11.992	2.47E-04	-2.10	no hit	-	-	-	-
-	Tag_1237 CATGGCTCGCAGCCGCCCCCTGGTGC	22	24	5.583	23.985	5.93E-07	-2.10	no hit	-	-	-	-
-	Tag_1783 CATGCAGCCAACGGTCACCAGCTGGA	12	13	3.045	12.992	1.55E-04	-2.09	no hit	-	-	-	-
Translation/Ribosome biogenesis	Tag_1668 CATGTCCGTGGCTCACGTGTCCTCGG	13	14	3.299	13.991	9.76E-05	-2.08	Hv_Contig_26679	582	142-117	AAK92158|ribosomal protein L27A *Spodoptera frugiperda*	3.00E-74
Defense	Tag_153 CATGGCCGACTATCCGCAGCGCGTAG	458	493	116.226	492.684	1.54E-103	-2.08	Hv_Contig_7687	1316	832-857	XP_013195286|plasminogen activator inhibitor 1 RNA-binding protein-like *Amyelois transitella*	2.00E-86
Primary metabolic process/hydrolase activity	Tag_510 CATGATCTGTGCTGCCCAACTCGGTA	105	113	26.646	112.928	2.02E-25	-2.08	Hv_Contig_14872	893	628-653	ABR88251|trypsin SP2b *Heliothis virescens*	3.00E-173
-	Tag_1643 CATGCTGGCCGGCGGTGAGTACCGCG	14	15	3.553	14.990	6.14E-05	-2.08	no hit	-	-	-	-
Primary metabolic process/hydrolase activity	Tag_267 CATGTTGTGCTCTGGCTGGCCCACTG	236	252	59.889	251.838	1.22E-53	-2.07	Hv_Contig_40046	302	14-39	ABR88253|trypsin T7 *Heliothis virescens*	1.00E-35
-	Tag_1007 CATGATCTGAGTTCAAACCGGTGTTA	31	33	7.867	32.979	1.03E-08	-2.07	no hit	-	-	-	-
Unknown	Tag_197 CATGATCGTCCGAGACACGCCCCCCA	337	358	85.520	357.770	7.23E-75	-2.06	Hv_Contig_2899	2068	1959-1984	XP_012545191|myosin heavy chain, muscle *Bombyx mori*	0.00E+00
Unknown	Tag_116 CATGTGGTGGACTCCGTTGCTGAGCC	634	669	160.889	668.571	6.33E-137	-2.06	Hv_Contig_37609	352	310-335	KOB65604|putative cuticle protein CPH38 *Operophtera brumata*	2.00E-11
-	Tag_91 CATGAGGCAGACGGTCGCTGTGGGTA	845	881	214.435	880.435	2.25E-177	-2.04	no hit	-	-	-	-
-	Tag_1147 CATGGGGATCAGAATGCTCCACCCGT	25	26	6.344	25.983	4.03E-07	-2.03	no hit	-	-	-	-
Primary metabolic process/transferase activity	Tag_609 CATGAGGGCGCCAGACCCCGCGCTCG	80	82	20.301	81.947	3.49E-18	-2.01	Hv_Contig_2039	2368	220-195	XP_004933765|chondroitin sulfate synthase 2 *Bombyx mori*	0.00E+00
-	Tag_857 CATGAGCCCCCTCCTGCCCGACACCC	42	43	10.658	42.972	1.92E-10	-2.01	no hit	-	-	-	-
Primary metabolic process/hydrolase activity	Tag_229 CATGGCCTACGCCGCCTGCATCGGCC	290	294	73.593	293.811	4.33E-59	-2.00	Hv_Contig_4802	1673	1509-1534	ACP39714|alkaline phosphatase *Heliothis virescens*	0.00E+00
-	Tag_501 CATGAGGCTGCCAAGGCCCTTGACAG	107	108	27.153	107.931	5.78E-23	-1.99	no hit	-	-	-	-
-	Tag_226 CATGAGGCAGACGGTCGCTGTGGGCG	293	295	74.354	294.811	7.21E-59	-1.99	no hit	-	-	-	-
Primary metabolic process/oxidoreductase activity	Tag_2505 CATGACTCGGGGCGCCGGCACGATTT	5	5	1.269	4.997	1.24E-02	-1.98	Hv_Contig_32521	473	291-316	KPJ05804|Carbonyl reductase [NADPH] 1 *Papilio xuthus*	3.00E-60
-	Tag_2513 CATGGCTCTCGGTATCCGCTCACCTA	5	5	1.269	4.997	1.24E-02	-1.98	no hit	-	-	-	-
-	Tag_2545 CATGGGGCCGTGGTTGCGCTGATGCT	5	5	1.269	4.997	1.24E-02	-1.98	no hit	-	-	-	-
-	Tag_2547 CATGAACCCCGTAGAGCATCCTCAGG	5	5	1.269	4.997	1.24E-02	-1.98	no hit	-	-	-	-
-	Tag_2570 CATGCCCGGCTTCCCCGACGGCGCGA	5	5	1.269	4.997	1.24E-02	-1.98	no hit	-	-	-	-
-	Tag_2572 CATGCATCATCAGCTGAGCAGCGGCG	5	5	1.269	4.997	1.24E-02	-1.98	no hit	-	-	-	-
-	Tag_2576 CATGCCCGGCTTCCCCGGCGGTGCGA	5	5	1.269	4.997	1.24E-02	-1.98	no hit	-	-	-	-
-	Tag_2581 CATGCCGTCGGCTACAGGTGAAGCAG	5	5	1.269	4.997	1.24E-02	-1.98	no hit	-	-	-	-
-	Tag_2583 CATGCAGCGCTCTGACGTCACGCGCA	5	5	1.269	4.997	1.24E-02	-1.98	no hit	-	-	-	-
-	Tag_2605 CATGATCTGAGTTCAAGCCGGTGTAA	5	5	1.269	4.997	1.24E-02	-1.98	no hit	-	-	-	-
-	Tag_2638 CATGTATGTGTAGGGCCGGCTGCTGA	5	5	1.269	4.997	1.24E-02	-1.98	no hit	-	-	-	-
-	Tag_2645 CATGGCCGCGCCCGCGCACTGCGGTA	5	5	1.269	4.997	1.24E-02	-1.98	no hit	-	-	-	-
-	Tag_2665 CATGGAGGACGCCGCAGCGCCCACAA	5	5	1.269	4.997	1.24E-02	-1.98	no hit	-	-	-	-
Unknown	Tag_2677 CATGGCATCCGCATTCACACCAACAC	5	5	1.269	4.997	1.24E-02	-1.98	Hv_Contig_14969	889	690-715	NP_001040378|bolA-like 3 *Bombyx mori*	2.00E-27
-	Tag_2694 CATGTCGCTCTCGCGCCATTGACCGG	5	5	1.269	4.997	1.24E-02	-1.98	no hit	-	-	-	-
-	Tag_2717 CATGCCCGTCTTCCCCGGCGGCGCGC	5	5	1.269	4.997	1.24E-02	-1.98	no hit	-	-	-	-
-	Tag_2719 CATGCACGGTAGCTGGAGCCCTAGCT	5	5	1.269	4.997	1.24E-02	-1.98	no hit	-	-	-	-
-	Tag_2722 CATGTGGATGTACCTGTGATCTCAGG	5	5	1.269	4.997	1.24E-02	-1.98	no hit	-	-	-	-
-	Tag_2090 CATGTGTTGATGCGGATGCCATTCCA	8	8	2.030	7.995	2.83E-03	-1.98	no hit	-	-	-	-
-	Tag_1086 CATGGGGCAGACGGTCGCTGTGGGTG	27	27	6.852	26.983	4.21E-07	-1.98	no hit	-	-	-	-
-	Tag_1111 CATGGAGGACGCCGCGGCACCCACGC	27	27	6.852	26.983	4.21E-07	-1.98	no hit	-	-	-	-
-	Tag_1377 CATGCCTGGGTGTGCCACCGCGCTAC	19	19	4.822	18.988	1.62E-05	-1.98	no hit	-	-	-	-
-	Tag_1816 CATGTCGCTCTCGCGCCATTGACCTA	11	11	2.791	10.993	6.73E-04	-1.98	no hit	-	-	-	-
Translation/Ribosome biogenesis	Tag_1848 CATGACTCGCAAACGCCGTAATGGAG	11	11	2.791	10.993	6.73E-04	-1.98	Hv_Contig_31571	493	93-118	AAK92194|ribosomal protein S26 *Spodoptera frugiperda*	5.00E-65
-	Tag_1874 CATGCCCGGCTTCCCCGGCGGCGTGA	11	11	2.791	10.993	6.73E-04	-1.98	no hit	-	-	-	-
Signal transduction	Tag_2345 CATGTTAGAGCGCGCCTGCCTCACTG	6	6	1.523	5.996	7.52E-03	-1.98	Hv_Contig_4034	1814	120-145	XP_013200700|zinc finger SWIM domain-containing protein 8-like *Amyelois transitella*	2.00E-111
Primary metabolic process/hydrolase activity	Tag_2359 CATGCAGGTTGTGATGGTACCCGACC	6	6	1.523	5.996	7.52E-03	-1.98	Hv_Contig_11981	1023	699-724	XP_013186138|pseudouridine-5'-phosphatase-like *Amyelois transitella*	1.00E-124
-	Tag_2363 CATGATCAGTGCCGGCTGGCCCAGCG	6	6	1.523	5.996	7.52E-03	-1.98	no hit	-	-	-	-

-	Tag_1973 CATGCTCAGGCACGCCAAGACGCAGG	9	9	2.284	8.994	1.75E-03	-1.98	no hit	-	-	-	-
-	Tag_2041 CATGAAGGTGCTGAAGAAGGCGGCCA	9	9	2.284	8.994	1.75E-03	-1.98	no hit	-	-	-	-
-	Tag_2056 CATGCTGTGCGCCACCGCGCTGCTGT	9	9	2.284	8.994	1.75E-03	-1.98	no hit	-	-	-	-
-	Tag_1485 CATGGCGGCCGCTGCTGACGTCCTAC	16	16	4.060	15.990	6.48E-05	-1.98	no hit	-	-	-	-
-	Tag_1510 CATGTTGTGCTCTGGCTGGCCCACGG	16	16	4.060	15.990	6.48E-05	-1.98	no hit	-	-	-	-
-	Tag_1524 CATGATCGTCCGAGACACGCCCCCTA	16	16	4.060	15.990	6.48E-05	-1.98	no hit	-	-	-	-
-	Tag_1897 CATGCGCGCAAGCGAGACGCGCGACC	10	10	2.538	9.994	1.08E-03	-1.98	no hit	-	-	-	-
-	Tag_3031 CATGCCGCGGAGCGTGAAGTGGATAC	3	3	0.761	2.998	3.48E-02	-1.98	no hit	-	-	-	-
Primary metabolic process/oxidoreductase activity	Tag_3052 CATGATCAAGCAAATCTGGGCTATCA	3	3	0.761	2.998	3.48E-02	-1.98	Hv_Contig_18073	779	536-561	XP_014355774|succinate dehydrogenase [ubiquinone] cytochrome b small subunit, mitochondrial *Papilio machaon*	2.00E-35
-	Tag_3058 CATGCACGGCAGCTGGAGCCCTAGAT	3	3	0.761	2.998	3.48E-02	-1.98	no hit	-	-	-	-
-	Tag_3060 CATGCCCGCACCCGCTGTGGTCATTC	3	3	0.761	2.998	3.48E-02	-1.98	no hit	-	-	-	-
Primary metabolic process/transferase activity	Tag_3089 CATGGTAACCATTGCGCCAACGAGGT	3	3	0.761	2.998	3.48E-02	-1.98	Hv_Contig_2081	2353	2339-2314	ALJ30257|putative acetyltransferase ACT10 *Spodoptera litura*	0.00E+00
-	Tag_3098 CATGCCGTCGGCCACAGGTGAAGTAC	3	3	0.761	2.998	3.48E-02	-1.98	no hit	-	-	-	-
-	Tag_3107 CATGATCACGAGCGGGCGGAACTTGG	3	3	0.761	2.998	3.48E-02	-1.98	no hit	-	-	-	-
-	Tag_3125 CATGGCGTATCCTGGCCCCGGCGCTG	3	3	0.761	2.998	3.48E-02	-1.98	no hit	-	-	-	-
-	Tag_3140 CATGAAGGTGCTGAAGAAGGCGGCTG	3	3	0.761	2.998	3.48E-02	-1.98	no hit	-	-	-	-
-	Tag_3141 CATGGCCGCTGCGCTCAGACAAGCTG	3	3	0.761	2.998	3.48E-02	-1.98	no hit	-	-	-	-
-	Tag_3148 CATGCGGCGGTGACTCCGGCGGCCCA	3	3	0.761	2.998	3.48E-02	-1.98	no hit	-	-	-	-
-	Tag_3150 CATGGACCTGGTGAAGACCGGTCGTT	3	3	0.761	2.998	3.48E-02	-1.98	no hit	-	-	-	-
-	Tag_3160 CATGGATGCCGGCTGGAGATATTGGG	3	3	0.761	2.998	3.48E-02	-1.98	no hit	-	-	-	-
-	Tag_3163 CATGCACGGCAGCTGGAGCCCTAGTA	3	3	0.761	2.998	3.48E-02	-1.98	no hit	-	-	-	-
-	Tag_3171 CATGTCGCTCTCGCGCCATTGACCCG	3	3	0.761	2.998	3.48E-02	-1.98	no hit	-	-	-	-
-	Tag_3174 CATGATCATTGTTCCTGAGATGGTAG	3	3	0.761	2.998	3.48E-02	-1.98	no hit	-	-	-	-
-	Tag_3177 CATGGAGCAAGCAGCGCGGAGTCTCT	3	3	0.761	2.998	3.48E-02	-1.98	no hit	-	-	-	-
Transport/Trafficking	Tag_3196 CATGGGGTACCTGGCAAAAGCTTTAT	3	3	0.761	2.998	3.48E-02	-1.98	Hv_Contig_29321	539	322-347	XP_013192564|mitochondrial import receptor subunit TOM40 homolog *Amyelois transitella*	2.00E-24
-	Tag_3210 CATGCCGTCGGCCACAGGTGAAGCTG	3	3	0.761	2.998	3.48E-02	-1.98	no hit	-	-	-	-
-	Tag_3220 CATGTGGGCGGATGCCACGCTCGCCA	3	3	0.761	2.998	3.48E-02	-1.98	no hit	-	-	-	-
Primary metabolic process/oxidoreductase activity	Tag_3221 CATGAAGAAGTACGTGGTGGGTGAGG	3	3	0.761	2.998	3.48E-02	-1.98	Hv_Contig_28973	546	267-292	ADU02195|cytochrome b5 *Helicoverpa armigera*	9.00E-83
-	Tag_3227 CATGCGAAGGCTAAAGCGTAAAAGAA	3	3	0.761	2.998	3.48E-02	-1.98	no hit	-	-	-	-
-	Tag_3239 CATGGACCGCGGGGAGGGCAGTCGGG	3	3	0.761	2.998	3.48E-02	-1.98	no hit	-	-	-	-
-	Tag_3247 CATGATCTGAGTTCAAACCGGTATAA	3	3	0.761	2.998	3.48E-02	-1.98	no hit	-	-	-	-
-	Tag_3251 CATGTGCCACGCGCACCTCGCGTAAC	3	3	0.761	2.998	3.48E-02	-1.98	no hit	-	-	-	-
-	Tag_3270 CATGACCATCCGCCGCAATCCCCGCG	3	3	0.761	2.998	3.48E-02	-1.98	no hit	-	-	-	-
Protein folding/Recycling	Tag_3274 CATGCCACGAGGGCCGAGGGTCGTGC	3	3	0.761	2.998	3.48E-02	-1.98	Hv_Contig_3445	1934	180-155	EHJ64371|chaperonin *Danaus plexippus*	0.00E+00
-	Tag_3284 CATGTCCGGCTTCCCCGGCGGCGCGA	3	3	0.761	2.998	3.48E-02	-1.98	no hit	-	-	-	-
-	Tag_3285 CATGATCTGTGCTGCCCAACTCGGAA	3	3	0.761	2.998	3.48E-02	-1.98	no hit	-	-	-	-
-	Tag_3286 CATGGCGACCTACCCACGAGACCAGA	3	3	0.761	2.998	3.48E-02	-1.98	no hit	-	-	-	-
-	Tag_3288 CATGATCTGAGTTCAAACCGGTGTCT	3	3	0.761	2.998	3.48E-02	-1.98	no hit	-	-	-	-
-	Tag_3296 CATGGAGCGGCGGCTGCTACGCTGAT	3	3	0.761	2.998	3.48E-02	-1.98	no hit	-	-	-	-
-	Tag_3302 CATGAAAGTGAGACTCGACACGGCAG	3	3	0.761	2.998	3.48E-02	-1.98	no hit	-	-	-	-
-	Tag_3309 CATGATTTGAGTTCAAACCGGTGTAA	3	3	0.761	2.998	3.48E-02	-1.98	no hit	-	-	-	-
Signal transduction	Tag_3310 CATGATTAAAATTGTTAAAATGAGCT	3	3	0.761	2.998	3.48E-02	-1.98	Hv_Contig_5908	1505	1429-1454	ADQ86007|death-associated LIM-only protein *Helicoverpa armigera*	6.00E-118
-	Tag_3344 CATGCGTCGTCCCACAGGCTTCCCTA	3	3	0.761	2.998	3.48E-02	-1.98	no hit	-	-	-	-
-	Tag_3352 CATGGGGAGGAGGCTGCGCTGATGCG	3	3	0.761	2.998	3.48E-02	-1.98	no hit	-	-	-	-
-	Tag_3357 CATGTTGTGCTCTGGCTGGCCCACGA	3	3	0.761	2.998	3.48E-02	-1.98	no hit	-	-	-	-
-	Tag_3363 CATGAGGCAGAGGGTCGCTGTGGGTG	3	3	0.761	2.998	3.48E-02	-1.98	no hit	-	-	-	-
-	Tag_3374 CATGTGTGCCACGACCTTTAGACAAA	3	3	0.761	2.998	3.48E-02	-1.98	no hit	-	-	-	-
-	Tag_3402 CATGGAGCGGCCGCAAGGGAATCTAG	3	3	0.761	2.998	3.48E-02	-1.98	no hit	-	-	-	-
-	Tag_3407 CATGGTTGTGTGAGCCTGTGCGGCCA	3	3	0.761	2.998	3.48E-02	-1.98	no hit	-	-	-	-
-	Tag_2196 CATGGCTGCGGCCCAGGGAGGACGGG	7	7	1.776	6.996	4.60E-03	-1.98	no hit	-	-	-	-
-	Tag_2203 CATGCCCGGCTTCCCAGGCGGCGCGC	7	7	1.776	6.996	4.60E-03	-1.98	no hit	-	-	-	-
-	Tag_2222 CATGTGCGACGTTCTTTGTGACGTAA	7	7	1.776	6.996	4.60E-03	-1.98	no hit	-	-	-	-
Cytoskeleton	Tag_2226 CATGTGTGCAGTGTGACTGTCTGAGT	7	7	1.776	6.996	4.60E-03	-1.98	Hv_Contig_19287	745	480-505	KPJ13413|Dynein light chain 2, cytoplasmic *Papilio machaon*	2.00E-58
Translation/Ribosome biogenesis	Tag_2271 CATGTCCCCTGATCCGGCAGCGGGAA	7	7	1.776	6.996	4.60E-03	-1.98	Hv_Contig_33391	452	202-177	NP_001037227|ribosomal protein L23 *Bombyx mori*	4.00E-87
-	Tag_2273 CATGATCGTCCGAGACACGCCCCTCA	7	7	1.776	6.996	4.60E-03	-1.98	no hit	-	-	-	-
-	Tag_2278 CATGTTCGTGGTGAAGGCGTACCTGA	7	7	1.776	6.996	4.60E-03	-1.98	no hit	-	-	-	-
-	Tag_2305 CATGTCCGTGTGCTCCAGCGACCGTC	7	7	1.776	6.996	4.60E-03	-1.98	no hit	-	-	-	-
-	Tag_2336 CATGGCCGTGTTAGCCGCGAACAACA	7	7	1.776	6.996	4.60E-03	-1.98	no hit	-	-	-	-

Transport/Trafficking	Tag_2733 CATGATGGCCACGTTGAACCGCGCCG	4	4	1.015	3.997	2.06E-02	-1.98	Hv_Contig_17783	788	188-163	XP_013199343|putative inorganic phosphate cotransporter *Amyelois transitella*	8.00E-127
-	Tag_2763 CATGCAGTTGCTGCGAGACAACCTAG	4	4	1.015	3.997	2.06E-02	-1.98	no hit	-	-	-	-
-	Tag_2783 CATGCACCGCGCCGATCCCCGACCCG	4	4	1.015	3.997	2.06E-02	-1.98	no hit	-	-	-	-
-	Tag_2806 CATGTACAGCCGAGCGGACCCGGCTA	4	4	1.015	3.997	2.06E-02	-1.98	no hit	-	-	-	-
Transport/Trafficking	Tag_2835 CATGGTTTGTACATTCCATATTATTT	4	4	1.015	3.997	2.06E-02	-1.98	Hv_Contig_3682	1816	1440-1465	P55277|H+-ATPase V-type subunit *Heliothis virescens*	4.00E-93
Primary metabolic process/oxidoreductase activity	Tag_2848 CATGGAAATTTATTGTAAATGACAGT	4	4	1.015	3.997	2.06E-02	-1.98	Hv_Contig_1985	2393	2324-2349	XP_013167730|14-3-3 protein epsilon *Papilio xuthus*	2.00E-163
-	Tag_2849 CATGGCTCGCAGCCGCCCCCTGGTCA	4	4	1.015	3.997	2.06E-02	-1.98	no hit	-	-	-	-
-	Tag_2858 CATGCGCCGCCACCACACCACGCGCC	4	4	1.015	3.997	2.06E-02	-1.98	no hit	-	-	-	-
-	Tag_2864 CATGCTGCGTATCGCTGAGCCCTACT	4	4	1.015	3.997	2.06E-02	-1.98	no hit	-	-	-	-
-	Tag_2890 CATGCGCCTGTGCGGCGGCCTAGGCC	4	4	1.015	3.997	2.06E-02	-1.98	no hit	-	-	-	-
-	Tag_2901 CATGGCCGACTATCCGCAGCGCGTTG	4	4	1.015	3.997	2.06E-02	-1.98	no hit	-	-	-	-
-	Tag_2907 CATGGGCTTCGGTCTGTTCGACTAAC	4	4	1.015	3.997	2.06E-02	-1.98	no hit	-	-	-	-
-	Tag_2908 CATGTGGTGGACTCCGTTGCTGAGCG	4	4	1.015	3.997	2.06E-02	-1.98	no hit	-	-	-	-
-	Tag_2928 CATGTTCATCCCGTCGCTGTCAGGCT	4	4	1.015	3.997	2.06E-02	-1.98	no hit	-	-	-	-
-	Tag_2933 CATGGATTGACACCCCCATCGCTCCG	4	4	1.015	3.997	2.06E-02	-1.98	no hit	-	-	-	-
-	Tag_2936 CATGAGGCTGCCAAGGCCCTTGACGT	4	4	1.015	3.997	2.06E-02	-1.98	no hit	-	-	-	-
-	Tag_2946 CATGCCCGACTTCCCCGGCGGCGCGT	4	4	1.015	3.997	2.06E-02	-1.98	no hit	-	-	-	-
-	Tag_2947 CATGCCCGGCTTCCTCGGCGGCGCGA	4	4	1.015	3.997	2.06E-02	-1.98	no hit	-	-	-	-
-	Tag_2955 CATGTGCGCGCTGGCCCCCTGTGGGT	4	4	1.015	3.997	2.06E-02	-1.98	no hit	-	-	-	-
Unknown	Tag_2961 CATGAAATGAGTACAACAAATGGCTT	4	4	1.015	3.997	2.06E-02	-1.98	Hv_Contig_12445	999	512-537	KOB53759|UPF0327 protein *Operophtera brumata*	4.00E-11
-	Tag_2969 CATGGGGAGGTTCCGCTCAGGACTGG	4	4	1.015	3.997	2.06E-02	-1.98	no hit	-	-	-	-
Translation/Ribosome biogenesis	Tag_2987 CATGTTCCCGATGCCGGTGGGAGACG	4	4	1.015	3.997	2.06E-02	-1.98	Hv_Contig_641	3477	3143-3168	XP_013182964|Tryptophan--tRNA ligase, cytoplasmic *Amyelois transitella*	0.00E+00
-	Tag_3004 CATGCACCCGCGTCACCCACTCCCCG	4	4	1.015	3.997	2.06E-02	-1.98	no hit	-	-	-	-
-	Tag_3005 CATGGTGGGCGGCACGTCTACGTCGA	4	4	1.015	3.997	2.06E-02	-1.98	no hit	-	-	-	-
-	Tag_3009 CATGGCCCGCAGCCGCCCCCTGGTTC	4	4	1.015	3.997	2.06E-02	-1.98	no hit	-	-	-	-
-	Tag_1593 CATGCCCCAACGCAGCACCGAGGACG	14	14	3.553	13.991	1.64E-04	-1.98	no hit	-	-	-	-
-	Tag_1623 CATGTCCACCCGAGGCCGGGCGCGGG	14	14	3.553	13.991	1.64E-04	-1.98	no hit	-	-	-	-
-	Tag_1708 CATGCGCGCGCCGCCGCTGCACTTTG	13	13	3.299	12.992	2.62E-04	-1.98	no hit	-	-	-	-
Nucleic acid binding	Tag_1170 CATGTTCCGCGTACGAAGTCGCGGGC	25	24	6.344	23.985	2.66E-06	-1.92	Hv_Contig_143	5100	4654-4679	EHJ65667|putative YLP motif containing 1 *Danaus plexippus*	2.00E-142
-	Tag_1304 CATGCGCCTGTGCGGCGGCCTGGGCG	21	20	5.329	19.987	1.66E-05	-1.91	no hit	-	-	-	-
-	Tag_1358 CATGGCTCGTGGTCACCTTGCTGCCG	19	18	4.822	17.988	4.16E-05	-1.90	no hit	-	-	-	-
Defense	Tag_1603 CATGGTGCGGCTGCTACAAAACGCAG	14	13	3.553	12.992	4.27E-04	-1.87	Hv_Contig_3864	1846	1634-1659	KOB65205|C-type lectin 17 *Operophtera brumata*	5.00E-171
-	Tag_760 CATGCTGCAGCACGTCACGCGCACGA	54	50	13.704	49.968	8.57E-11	-1.87	no hit	-	-	-	-
Transport/Trafficking	Tag_1698 CATGGTCCGCGTACGAAGTCGCGGGC	13	12	3.299	11.992	6.84E-04	-1.86	Hv_Contig_19780	729	552-527	XP_004925267|synaptic vesicle glycoprotein 2A-like *Bombyx mori*	2.00E-47
-	Tag_1700 CATGCGGTTCATCCCGGGCGGAGCGA	13	12	3.299	11.992	6.84E-04	-1.86	no hit	-	-	-	-
-	Tag_1754 CATGTGCCCACACTTGCTGTCTTGCA	12	11	3.045	10.993	1.10E-03	-1.85	no hit	-	-	-	-
-	Tag_1756 CATGCACGCGCGCGCGGGCGACGCGA	12	11	3.045	10.993	1.10E-03	-1.85	no hit	-	-	-	-
-	Tag_1771 CATGATCTGTGCCGGCTGGCCCAGGG	12	11	3.045	10.993	1.10E-03	-1.85	no hit	-	-	-	-
-	Tag_599 CATGAACCGCGGCAGCGAGCAGCACA	82	75	20.809	74.952	4.81E-15	-1.85	no hit	-	-	-	-
-	Tag_397 CATGCGCCTGTGCGGCGGCCTGGGCA	152	139	38.573	138.911	3.68E-26	-1.85	no hit	-	-	-	-
-	Tag_1813 CATGAAAGTAGCCTGATACCTACGTG	11	10	2.791	9.994	1.77E-03	-1.84	no hit	-	-	-	-
Primary metabolic process/hydrolase activity	Tag_1264 CATGTGCTGGCGTCTGTAACCTGAGC	22	20	5.583	19.987	2.62E-05	-1.84	Hv_Contig_9364	1183	907-932	AFO68320|trypsin *Heliothis virescens*	3.00E-107
-	Tag_448 CATGCTGGACCTGGCGCAGGTGGCCA	124	112	31.467	111.928	3.38E-21	-1.83	no hit	-	-	-	-
-	Tag_1907 CATGGCCTACGCCGCCTGCATCGGCT	10	9	2.538	8.994	2.86E-03	-1.83	no hit	-	-	-	-
Unknown	Tag_1942 CATGATAATCGAGCAAGAGGAGGCTG	10	9	2.538	8.994	2.86E-03	-1.83	Hv_Contig_29039	544	417-442	BAM18860|unknown unsecreted protein *Papilio xuthus*	9.00E-12
-	Tag_237 CATGATCTGAGTTCAAACCGGTGTAT	273	245	69.279	244.843	1.49E-43	-1.82	no hit	-	-	-	-
-	Tag_90 CATGCGCGCAAGCGAGACGCGCGATC	851	761	215.957	760.512	6.55E-130	-1.82	no hit	-	-	-	-
Primary metabolic process/hydrolase activity	Tag_823 CATGCACGGCAGCTGGACCCCTAGCC	46	41	11.673	40.974	7.33E-09	-1.81	Hv_Contig_10149	1128	433-458	CAA72958|chymotrypsin-like protease *Helicoverpa armigera*	5.00E-165
Primary metabolic process/oxidoreductase activity	Tag_1114 CATGGAGGAAGATCAGTAGATTTAGC	27	24	6.852	23.985	6.55E-06	-1.81	Hv_Contig_4748	1681	513-538	YP_009019855|cytochrome c oxidase subunit I, partial (mitochondrion) *Helicoverpa punctigera*	0.00E+00
-	Tag_1994 CATGCCCGGCCTCCCCGGCGGCGCGT	9	8	2.284	7.995	4.64E-03	-1.81	no hit	-	-	-	-
-	Tag_2005 CATGAGGTAGACGGTCGCTGTGGGTG	9	8	2.284	7.995	4.64E-03	-1.81	no hit	-	-	-	-
-	Tag_2025 CATGCAGTTGCTGCGAGACAACCTGC	9	8	2.284	7.995	4.64E-03	-1.81	no hit	-	-	-	-
Signal transduction	Tag_2026 CATGCTCTGGGATCTGAATGATGGCA	9	8	2.284	7.995	4.64E-03	-1.81	Hv_Contig_7985	1286	752-777	AEB26318|receptor for activated protein kinase C *Helicoverpa armigera*	0.00E+00
-	Tag_2027 CATGTCGACTTGCCCGTTATCACAGA	9	8	2.284	7.995	4.64E-03	-1.81	no hit	-	-	-	-
-	Tag_2045 CATGCCGTTGGCCACAGGTGAAGCAG	9	8	2.284	7.995	4.64E-03	-1.81	no hit	-	-	-	-
-	Tag_2065 CATGCGCCGCGCAGTGCGCGCACACA	9	8	2.284	7.995	4.64E-03	-1.81	no hit	-	-	-	-
-	Tag_214 CATGAGGCAGACGGTCGCTGTGGGTC	308	273	78.161	272.825	1.35E-47	-1.80	no hit	-	-	-	-
-	Tag_708 CATGTGCGACGTTCTTTGTGACGTCG	61	54	15.480	53.965	5.24E-11	-1.80	no hit	-	-	-	-

-	Tag_639 CATGCTGGACCTGGCGCAGGTGGCTG	76	67	19.286	66.957	3.84E-13	-1.80	no hit	-	-	-	-
-	Tag_34 CATGCCCGGCTTCCCCGGCGGCGCGA	3329	2920	844.796	2918.127	0.00E+00	-1.79	no hit	-	-	-	-
-	Tag_2073 CATGGTATCGCCATCAGTGCCCGACC	8	7	2.030	6.996	7.58E-03	-1.78	no hit	-	-	-	-
-	Tag_2075 CATGCTTCAGTAGAGGGAAGTAGGGC	8	7	2.030	6.996	7.58E-03	-1.78	no hit	-	-	-	-
-	Tag_2135 CATGCCCGGCTTCCCCGGCGGCGCCA	8	7	2.030	6.996	7.58E-03	-1.78	no hit	-	-	-	-
-	Tag_1500 CATGTCCGTGCGCTCCAGCGACCATG	16	14	4.060	13.991	4.21E-04	-1.78	no hit	-	-	-	-
-	Tag_671 CATGTCCGTGCGCTCCAGCGACCGGA	67	58	17.002	57.963	2.04E-11	-1.77	no hit	-	-	-	-
-	Tag_1613 CATGCCCTTGCCCCGCGCCCCGCGAG	14	12	3.553	11.992	1.08E-03	-1.76	no hit	-	-	-	-
-	Tag_1295 CATGAACCGCGGCAGCGAGCAGCACG	21	18	5.329	17.988	1.02E-04	-1.76	no hit	-	-	-	-
Translation/Ribosome biogenesis	Tag_2251 CATGCACGACGCACAGCAAGTCCTCC	7	6	1.776	5.996	1.24E-02	-1.76	Hv_Contig_16686	822	409-384	ADT80643|ribosomal protein S3 *Euphydryas aurinia*	7.00E-158
-	Tag_2279 CATGATCTGAGTTCAGACCGGTGTAA	7	6	1.776	5.996	1.24E-02	-1.76	no hit	-	-	-	-
-	Tag_2304 CATGTGCGCGGCGAGCCTCGCCTCCG	7	6	1.776	5.996	1.24E-02	-1.76	no hit	-	-	-	-
-	Tag_2306 CATGATCGTCCGAGACACGCCCCCTG	7	6	1.776	5.996	1.24E-02	-1.76	no hit	-	-	-	-
-	Tag_51 CATGTCCGTGCGCTCCAGCGACCGTA	2001	1715	507.791	1713.900	4.56E-275	-1.75	no hit	-	-	-	-
-	Tag_1103 CATGAGGCAGGCGGTCGCTGTGGGTG	27	23	6.852	22.985	1.57E-05	-1.75	no hit	-	-	-	-
-	Tag_669 CATGTCCACCCGAGGCCGGGCGCGGA	68	57	17.256	56.963	7.06E-11	-1.72	no hit	-	-	-	-
-	Tag_493 CATGAGGCAGACGGTCGCTGTGGGTT	110	92	27.915	91.941	2.45E-16	-1.72	no hit	-	-	-	-
-	Tag_1032 CATGGTGGAGCCGTACAACCTGCGGC	30	25	7.613	24.984	9.59E-06	-1.71	no hit	-	-	-	-
-	Tag_2355 CATGAAGCTCGGCGCGGGTCTCCTGA	6	5	1.523	4.997	2.06E-02	-1.71	no hit	-	-	-	-
-	Tag_2367 CATGTCCGTGCGCTTCAGCGACCGTG	6	5	1.523	4.997	2.06E-02	-1.71	no hit	-	-	-	-
-	Tag_2401 CATGCTGGCGGCGCGCTACTCGCCAC	6	5	1.523	4.997	2.06E-02	-1.71	no hit	-	-	-	-
-	Tag_2413 CATGAACCGCGGCAGCGAGCAGCGCC	6	5	1.523	4.997	2.06E-02	-1.71	no hit	-	-	-	-
-	Tag_2419 CATGAAGGCGCTGGCTGAGAAGCTCG	6	5	1.523	4.997	2.06E-02	-1.71	no hit	-	-	-	-
-	Tag_2420 CATGTGCGCGCTGACTCCCTGTGGGT	6	5	1.523	4.997	2.06E-02	-1.71	no hit	-	-	-	-
-	Tag_2457 CATGCACGGCAGCTGGAGCCCTAGTG	6	5	1.523	4.997	2.06E-02	-1.71	no hit	-	-	-	-
Protein kinases	Tag_1720 CATGCAGATGGGTGGTGACCTGAAGC	12	10	3.045	9.994	2.77E-03	-1.71	Hv_Contig_8820	1220	560-585	ABU98622|arginine kinase *Helicoverpa armigera*	1.00E-76
-	Tag_1755 CATGGTTTGGGAAGGCCTCAACGCAA	12	10	3.045	9.994	2.77E-03	-1.71	no hit	-	-	-	-
-	Tag_1759 CATGATCTGAGTTCAAACCGGCGTAA	12	10	3.045	9.994	2.77E-03	-1.71	no hit	-	-	-	-
-	Tag_1776 CATGCGCGCGCCGCCGCTGCACTTCC	12	10	3.045	9.994	2.77E-03	-1.71	no hit	-	-	-	-
-	Tag_1176 CATGTCCGTGCGCTCCAGCGACCGAC	24	20	6.090	19.987	6.16E-05	-1.71	no hit	-	-	-	-
-	Tag_111 CATGGGGAAGAGGCTGCGCTGATGCG	686	569	174.085	568.635	3.41E-89	-1.71	no hit	-	-	-	-
-	Tag_963 CATGTGCCGCTACGTGCGCGCGCACG	34	28	8.628	27.982	3.71E-06	-1.70	no hit	-	-	-	-
-	Tag_1455 CATGGTATCGCCATCAGTGCCCGACG	17	14	4.314	13.991	6.45E-04	-1.70	no hit	-	-	-	-
-	Tag_1456 CATGGCCAATGCCACTTACGCGCCGC	17	14	4.314	13.991	6.45E-04	-1.70	no hit	-	-	-	-
Translation/Ribosome biogenesis	Tag_1460 CATGTTGTGATGGGGGCTCAACCTTT	17	14	4.314	13.991	6.45E-04	-1.70	Hv_Contig_4865	1662	1299-1324	XP_013197196|eukaryotic translation initiation factor 3 subunit E *Amyelois transitella*	0.00E+00
-	Tag_911 CATGAGGCAGACGGTCGCTGTGGGGC	37	30	9.389	29.981	2.25E-06	-1.67	no hit	-	-	-	-
-	Tag_1543 CATGCCGTCGGCCACAGGTGAAGCGC	15	12	3.807	11.992	1.64E-03	-1.66	no hit	-	-	-	-
-	Tag_2504 CATGGAGGGCGGCGCGTACGGCGGTG	5	4	1.269	3.997	3.45E-02	-1.66	no hit	-	-	-	-
Protein folding/Recycling	Tag_2526 CATGTGTCATTCCAAGCTCTCATTGT	5	4	1.269	3.997	3.45E-02	-1.66	Hv_Contig_26413	586	478-503	AGQ42773|small heat shock protein G16 *Helicoverpa armigera*	1.00E-59
-	Tag_2551 CATGCCCCTACACACACTAAGTGAAT	5	4	1.269	3.997	3.45E-02	-1.66	no hit	-	-	-	-
-	Tag_2595 CATGCGCCTGTGCGGCGGCCTGGACC	5	4	1.269	3.997	3.45E-02	-1.66	no hit	-	-	-	-
-	Tag_2607 CATGTGGATGTACCTGTGATCTCAGA	5	4	1.269	3.997	3.45E-02	-1.66	no hit	-	-	-	-
-	Tag_2628 CATGTCCGTGCGCACCAGCGACCGTT	5	4	1.269	3.997	3.45E-02	-1.66	no hit	-	-	-	-
-	Tag_2629 CATGCCGCGCTCGCCCGAGCGGCGGA	5	4	1.269	3.997	3.45E-02	-1.66	no hit	-	-	-	-
-	Tag_2631 CATGCCCAGCTTCCCCGGCGGCGCGT	5	4	1.269	3.997	3.45E-02	-1.66	no hit	-	-	-	-
-	Tag_2654 CATGCGCCTGTGCGGCAGCCTGGGCC	5	4	1.269	3.997	3.45E-02	-1.66	no hit	-	-	-	-
-	Tag_2666 CATGCGTGACCCCACGCCCGCGCCAT	5	4	1.269	3.997	3.45E-02	-1.66	no hit	-	-	-	-
-	Tag_2680 CATGTTTGTCAGGGTCAGCGCCTACC	5	4	1.269	3.997	3.45E-02	-1.66	no hit	-	-	-	-
-	Tag_2684 CATGGAAATTTATTGTAAATGACAGG	5	4	1.269	3.997	3.45E-02	-1.66	no hit	-	-	-	-
-	Tag_2693 CATGCCCGGCTTGCCCGGCGGCGCGA	5	4	1.269	3.997	3.45E-02	-1.66	no hit	-	-	-	-
-	Tag_937 CATGGGGAAGAGGCTGCGCTGATGCA	35	28	8.882	27.982	5.47E-06	-1.66	no hit	-	-	-	-
Cytoskeleton	Tag_1892 CATGGTCTACAACGACCTGCTGCAGA	10	8	2.538	7.995	7.25E-03	-1.66	Hv_Contig_107	5450	5261-5286	XP_013193227|lissencephaly-1 homolog isoform X1 *Amyelois transitella*	0.00E+00
-	Tag_1918 CATGTCAAAAGTCGGCGACAATATTG	10	8	2.538	7.995	7.25E-03	-1.66	no hit	-	-	-	-
-	Tag_1970 CATGCGGCAGTTCCGTACGGGATCCA	10	8	2.538	7.995	7.25E-03	-1.66	no hit	-	-	-	-
Translation/Ribosome biogenesis	Tag_194 CATGGGGATCAGAATGCTCCACCCGC	341	272	86.535	271.826	1.04E-41	-1.65	Hv_Contig_18762	759	275-300	Q8WQI5|40S ribosomal protein S8 *Spodoptera frugiperda*	1.00E-147
-	Tag_721 CATGATCTGTGCCGGCTGGCCCAGAG	59	47	14.972	46.970	8.31E-09	-1.65	no hit	-	-	-	-
-	Tag_1192 CATGGGGATCAGAATGCTCCACCCGA	24	19	6.090	18.988	1.44E-04	-1.64	no hit	-	-	-	-
-	Tag_532 CATGAGCCCCCTCCTGCCCGACACCG	100	79	25.377	78.949	2.13E-13	-1.64	no hit	-	-	-	-

-	Tag_1611 CATGGCCATGACCGTGGCCTTGTGTG	14	11	3.553	10.993	2.62E-03	-1.63	no hit	-	-	-	-
Signal transduction	Tag_667 CATGCGGCGCCTCGCGCACCCCGACC	68	53	17.256	52.966	1.83E-09	-1.62	Hv_Contig_10645	1098	373-398	XP_012545559|signal transducing adapter molecule 2 *Bombyx mori*	1.00E-55
-	Tag_1984 CATGCCTGGCTTCCCCGGCGGCGCGA	9	7	2.284	6.996	1.18E-02	-1.61	no hit	-	-	-	-
-	Tag_2004 CATGCCCGGCTCCCCCGGCGGCGCGA	9	7	2.284	6.996	1.18E-02	-1.61	no hit	-	-	-	-
-	Tag_175 CATGGAGGCTATCGCCGTCGCCCTCC	399	310	101.254	309.801	1.26E-45	-1.61	no hit	-	-	-	-
-	Tag_201 CATGATCTGAGTTCAAACCGGTGTGA	327	254	82.982	253.837	9.12E-38	-1.61	no hit	-	-	-	-
-	Tag_1674 CATGCAAGCGCTGCAAGAGGACCGAA	13	10	3.299	9.994	4.19E-03	-1.60	no hit	-	-	-	-
-	Tag_1142 CATGGACGAGCTCCCGGTGCCGCGAG	26	20	6.598	19.987	1.34E-04	-1.60	no hit	-	-	-	-
-	Tag_334 CATGTCCGTGCGCTCCAGCGACCGGG	184	141	46.693	140.910	1.62E-21	-1.59	no hit	-	-	-	-
-	Tag_1294 CATGGAGGCGCTGCTCGGCTGAGCCA	21	16	5.329	15.990	5.64E-04	-1.59	no hit	-	-	-	-
Unknown	Tag_641 CATGCGCCGCCACCACACCACGCGCT	75	57	19.033	56.963	9.47E-10	-1.58	Hv_Contig_305	4221	3843-3868	XP_013186533|guanylate cyclase 32E-like isoform X1 *Amyelois transitella*	0.00E+00
-	Tag_595 CATGCGACAGGCACGCGTGTAGGCTG	83	63	21.063	62.960	1.45E-10	-1.58	no hit	-	-	-	-
-	Tag_1483 CATGGCGCCCGGCCCGCTCGCCGGCC	16	12	4.060	11.992	2.42E-03	-1.56	no hit	-	-	-	-
-	Tag_1501 CATGAAACAGGGAGTCCTCACCAACG	16	12	4.060	11.992	2.42E-03	-1.56	no hit	-	-	-	-
-	Tag_2084 CATGCCCGGCTTCCCTGGCGGCGCGA	8	6	2.030	5.996	1.93E-02	-1.56	no hit	-	-	-	-
-	Tag_2095 CATGATTGCCGTCGTGGTCGGAAGAA	8	6	2.030	5.996	1.93E-02	-1.56	no hit	-	-	-	-
-	Tag_2100 CATGTTGAGTCTGGCGACCGCGTCTC	8	6	2.030	5.996	1.93E-02	-1.56	no hit	-	-	-	-
-	Tag_2108 CATGACCGCGGCCGAGATGCGCGCGA	8	6	2.030	5.996	1.93E-02	-1.56	no hit	-	-	-	-
-	Tag_2115 CATGGAGCGACTGCCTGTCTGGCCTG	8	6	2.030	5.996	1.93E-02	-1.56	no hit	-	-	-	-
-	Tag_2134 CATGGCGTGCGCAGATTCCGGCTACT	8	6	2.030	5.996	1.93E-02	-1.56	no hit	-	-	-	-
-	Tag_2142 CATGTCCATGCGCTCCAGCGACCGTG	8	6	2.030	5.996	1.93E-02	-1.56	no hit	-	-	-	-
Unknown	Tag_2150 CATGGCTTGAACCAGGGCTGGGCGCG	8	6	2.030	5.996	1.93E-02	-1.56	Hv_Contig_40611	291	49-24	EHJ73698|hypothetical protein KGM_17703 *Danaus plexippus*	8.00E-10
-	Tag_2153 CATGTCCGTGCGCTCCAGTGACCGTC	8	6	2.030	5.996	1.93E-02	-1.56	no hit	-	-	-	-
-	Tag_2165 CATGCTGTCGCGTGGCGCACTCAATC	8	6	2.030	5.996	1.93E-02	-1.56	no hit	-	-	-	-
-	Tag_1203 CATGCCGTCGGCCACAGGTGAAGCGA	24	18	6.090	17.988	3.30E-04	-1.56	no hit	-	-	-	-
Unknown	Tag_930 CATGGCCGTTCTTAGTTGGTGGAGCG	36	27	9.136	26.983	1.78E-05	-1.56	Hv_Contig_43519	248	135-110	CDW75723|UNKNOWN *Stylonychia lemnae*	7.00E-25
-	Tag_1782 CATGGGGCTACGGCTGTGCCAATGCC	12	9	3.045	8.994	6.73E-03	-1.56	no hit	-	-	-	-
Unknown	Tag_1784 CATGTGACACGCCCTATCTGCCAAAT	12	9	3.045	8.994	6.73E-03	-1.56	Hv_Contig_2540	2176	1872-1897	XP_013186294|uncharacterized protein LOC106131668 *Amyelois transitella*	6.00E-161
signal transduction	Tag_141 CATGCTGGCGGCGCGCTACTCGCCGC	510	380	129.422	379.756	2.48E-52	-1.55	Hv_Contig_1832	2455	1930-1955	XP_013191803|guanine nucleotide-binding protein G(s) subunit alpha isoform X1 *Amyelois transitella*	0.00E+00
-	Tag_1100 CATGGTCGAGATGCCGCCCACCGTCA	27	20	6.852	19.987	1.93E-04	-1.54	no hit	-	-	-	-
Translation/Ribosome biogenesis	Tag_16 CATGAGGCAGACGGTCGCTGTGGGTG	9229	6819	2342.031	6814.626	0.00E+00	-1.54	Hv_Contig_35659	396	77-102	AAG23839|elongation factor-1 alpha *Corcyra cephalonica*	2.00E-27
-	Tag_1390 CATGTCCAACCCCAGGCAATACAAAG	19	14	4.822	13.991	1.40E-03	-1.54	no hit	-	-	-	-
Unknown	Tag_1546 CATGCCCCAGTCCTCCTCGCTCACCA	15	11	3.807	10.993	3.85E-03	-1.53	Hv_Contig_24277	619	504-529	BAM18243|cuticular protein PxutCPR103c *Papilio xuthus*	1.00E-36
-	Tag_717 CATGAAGCTCGGCGCGGGTCTCCCGG	60	44	15.226	43.972	1.26E-07	-1.53	no hit	-	-	-	-
-	Tag_834 CATGGGACCAACAAGGCAAGAACGGT	45	33	11.420	32.979	3.69E-06	-1.53	no hit	-	-	-	-
-	Tag_1269 CATGAGGCAGACGGTCGCCGTGGGTG	22	16	5.583	15.990	8.12E-04	-1.52	no hit	-	-	-	-
-	Tag_1793 CATGGTAGGCTACAACGTACACGCAC	11	8	2.791	7.995	1.08E-02	-1.52	no hit	-	-	-	-
-	Tag_1837 CATGGCGGCCGCTGCTGACGTCCTAT	11	8	2.791	7.995	1.08E-02	-1.52	no hit	-	-	-	-
-	Tag_1849 CATGCCCGTGCGCTCCAGCGACCGTA	11	8	2.791	7.995	1.08E-02	-1.52	no hit	-	-	-	-
-	Tag_1855 CATGATCTGAGTTCAAACCGGTGTCG	11	8	2.791	7.995	1.08E-02	-1.52	no hit	-	-	-	-
Translation/Ribosome biogenesis	Tag_1856 CATGAGAGGAAGAACAAGCTGTTCAA	11	8	2.791	7.995	1.08E-02	-1.52	Hv_Contig_24143	622	244-269	AAK92158|ribosomal protein L27A *Spodoptera frugiperda*	8.00E-74
-	Tag_1873 CATGTCCGTGCGCCCCAGCGACCGTC	11	8	2.791	7.995	1.08E-02	-1.52	no hit	-	-	-	-
-	Tag_1880 CATGCACGACAGCTGGAGCCCTAGCT	11	8	2.791	7.995	1.08E-02	-1.52	no hit	-	-	-	-
Primary metabolic process/oxidoreductase activity	Tag_133 CATGAAGCTCGGCGCGGGTCTCCCGA	535	389	135.766	388.751	1.10E-51	-1.52	Hv_Contig_9651	1164	915-940	AID66695|putative 3-hydroxyacyl-CoA dehydrogenase *Agrotis segetum*	0.00E+00
Transport/Trafficking	Tag_462 CATGGCGGCCGCCTTCCGCAACCTGG	120	87	30.452	86.944	4.39E-13	-1.51	Hv_Contig_2174	2289	1573-1598	AJQ81220|V-type ATP synthase catalytic subunit A *Helicoverpa armigera*	0.00E+00
Translation/Ribosome biogenesis	Tag_339 CATGTTCGTGGTGAAGGCGTACCTGC	181	131	45.932	130.916	1.13E-18	-1.51	Hv_Contig_1033	2992	2422-2447	AAL83698|translation elongation factor 2 *Spodoptera exigua*	0.00E+00
-	Tag_923 CATGTCCCTCACGGACCAGTCTGCTG	36	26	9.136	25.983	3.91E-05	-1.51	no hit	-	-	-	-
Unknown	Tag_39 CATGATCTGAGTTCAAACCGGTGTAA	2867	2070	727.555	2068.672	4.38E-262	-1.51	Hv_Contig_12866	978	178-153	ABK26259|unknown *Picea sitchensis*	2.00E-13
-	Tag_1143 CATGCTGCAGCACGCGCTGGGCGTCG	25	18	6.344	17.988	4.71E-04	-1.50	no hit	-	-	-	-
-	Tag_992 CATGTTGAGCAGCCGCGCGGCTATAG	32	23	8.121	22.985	1.03E-04	-1.50	no hit	-	-	-	-
Protein kinases	Tag_2214 CATGGAATCTGGGGTTGGGATGAATA	7	5	1.776	4.997	3.18E-02	-1.49	Hv_Contig_5416	1578	1550-1575	XP_013140780|serine/threonine-protein kinase RIO2 *Papilio polytes*	0.00E+00
-	Tag_2216 CATGTCCACAACTGAATATACCTCGG	7	5	1.776	4.997	3.18E-02	-1.49	no hit	-	-	-	-
-	Tag_2239 CATGGCGATGACGGCCCGGGCCTATG	7	5	1.776	4.997	3.18E-02	-1.49	no hit	-	-	-	-
-	Tag_2257 CATGTACGGCAGCTGGAGCCCTAGCT	7	5	1.776	4.997	3.18E-02	-1.49	no hit	-	-	-	-
-	Tag_2259 CATGCTCACCGCCAGCGCTGAGGAAG	7	5	1.776	4.997	3.18E-02	-1.49	no hit	-	-	-	-
-	Tag_2289 CATGTCGCCCAGGTGTATGCTATCAA	7	5	1.776	4.997	3.18E-02	-1.49	no hit	-	-	-	-
-	Tag_2300 CATGGCTCGTGGTCACCTTGCTGCTA	7	5	1.776	4.997	3.18E-02	-1.49	no hit	-	-	-	-
-	Tag_2315 CATGAGCTGGATTCAAAGACACCTTG	7	5	1.776	4.997	3.18E-02	-1.49	no hit	-	-	-	-

-	Tag_2327 CATGTCCACCCGAGGCCGGGCGTGGC	7	5	1.776	4.997	3.18E-02	-1.49	no hit	-	-	-	-
-	Tag_2335 CATGGGCCCACCCCAGAGATTGTATT	7	5	1.776	4.997	3.18E-02	-1.49	no hit	-	-	-	-
-	Tag_1584 CATGCTTCGTCTTCCTGTTGCGTATG	14	10	3.553	9.994	6.13E-03	-1.49	no hit	-	-	-	-
-	Tag_1586 CATGTCTGTGCGCTCCAGCGACCGTG	14	10	3.553	9.994	6.13E-03	-1.49	no hit	-	-	-	-
-	Tag_1589 CATGAAACAGGGAGTCCTCACCAACT	14	10	3.553	9.994	6.13E-03	-1.49	no hit	-	-	-	-
Primary metabolic process/oxidoreductase activity	Tag_1070 CATGAACAATTAAAAAATACTGTAAC	28	20	7.106	19.987	2.74E-04	-1.49	Hv_Contig_31027	506	423-448	KPJ10949|Cytochrome c oxidase subunit 7C, mitochondrial *Papilio machaon*	5.00E-30
-	Tag_1472 CATGTCCGTGCGCTCCAGCGGCCGTG	17	12	4.314	11.992	3.49E-03	-1.47	no hit	-	-	-	-
-	Tag_1473 CATGTCCGTGCGCCCCAGCGACCGTG	17	12	4.314	11.992	3.49E-03	-1.47	no hit	-	-	-	-
-	Tag_1098 CATGCGCCTGTGCGGCGGCCTGGGTC	27	19	6.852	18.988	4.26E-04	-1.47	no hit	-	-	-	-
-	Tag_914 CATGAGGCGGACGGTCGCTGTGGGTG	37	26	9.389	25.983	5.46E-05	-1.47	no hit	-	-	-	-
Unknown	Tag_173 CATGTGCGACGTTCTTTGTGACGTCA	402	282	102.015	281.819	3.93E-36	-1.47	Hv_Contig_46846	208	152-127	KPJ04392|hypothetical protein RR46_01761 *Papilio xuthus*	1.00E-17
Unknown	Tag_1884 CATGGACCAGCTCACCAACCAGCTCA	10	7	2.538	6.996	1.75E-02	-1.46	Hv_Contig_7280	1352	576-601	XP_014357512|tropomyosin-1 isoform X1 *Papilio machaon*	2.00E-156
-	Tag_1940 CATGATGGTGGGCGTGTTCCTGTTCC	10	7	2.538	6.996	1.75E-02	-1.46	no hit	-	-	-	-
-	Tag_1335 CATGGATGTCAAGTGCCCTGGCTGCA	20	14	5.075	13.991	2.00E-03	-1.46	no hit	-	-	-	-
-	Tag_927 CATGGAGTGCGGGCTGCGCTTCGACC	36	25	9.136	24.984	8.41E-05	-1.45	no hit	-	-	-	-
-	Tag_1133 CATGTACAGCCGAGCGGACCCGGCCG	26	18	6.598	17.988	6.62E-04	-1.45	no hit	-	-	-	-
-	Tag_1138 CATGGAGTGCGGACTGCGCTTCGAGC	26	18	6.598	17.988	6.62E-04	-1.45	no hit	-	-	-	-
-	Tag_1657 CATGCGCGCGCCGCACCACGACAACA	13	9	3.299	8.994	9.78E-03	-1.45	no hit	-	-	-	-
-	Tag_1690 CATGCCCGGCCTCCCCGGCGGCGCGA	13	9	3.299	8.994	9.78E-03	-1.45	no hit	-	-	-	-
-	Tag_123 CATGCGTGCCGCGTCAGACGCGGTCG	572	395	145.156	394.747	1.45E-48	-1.44	no hit	-	-	-	-
-	Tag_856 CATGCGCCGCGCAGTGCGCGCACACG	42	29	10.658	28.981	2.88E-05	-1.44	no hit	-	-	-	-
Unknown	Tag_456 CATGGGCGGCAGTCGCGCAGAAAGAA	122	84	30.960	83.946	7.40E-12	-1.44	Hv_Contig_20913	699	623-648	XP_013142804|uncharacterized protein LOC106106714 *Papilio polytes*	1.00E-101
-	Tag_1518 CATGATCTGAGTTCAAACCGGTGTCA	16	11	4.060	10.993	5.51E-03	-1.44	no hit	-	-	-	-
-	Tag_1000 CATGGCGGCCGCTGCTGACGTCCTAA	32	22	8.121	21.986	2.22E-04	-1.44	no hit	-	-	-	-
-	Tag_1250 CATGGGCCCGGGGCCGGGGCCGGGCG	22	15	5.583	14.990	1.79E-03	-1.42	no hit	-	-	-	-
Translation/Ribosome biogenesis	Tag_60 CATGGGACCAACAAGGCAAGAACGGC	1559	1062	395.625	1061.319	5.56E-124	-1.42	Hv_Contig_21415	684	556-581	AAL26578|ribosomal protein S3 *Spodoptera frugiperda*	7.00E-17
-	Tag_426 CATGTCCGTGCGCTCCAGCGACCGCG	137	93	34.766	92.940	1.13E-12	-1.42	no hit	-	-	-	-
-	Tag_408 CATGCGCGGCCCGCCCGCCTACGTGA	148	100	37.558	99.936	2.15E-13	-1.41	no hit	-	-	-	-
-	Tag_844 CATGAGGCAGACGGTCGCTGTGGATG	43	29	10.912	28.981	3.94E-05	-1.41	no hit	-	-	-	-
-	Tag_286 CATGCCTGGGTGTGCCACCGCGCTAT	223	150	56.590	149.904	5.48E-19	-1.41	no hit	-	-	-	-
Nucleic acid binding	Tag_1429 CATGACCTTGCATACCAGCTCGGCCT	18	12	4.568	11.992	4.91E-03	-1.39	Hv_Contig_34628	421	281-306	EHJ73915|Myb-MuvB complex subunit Lin-52 *Danaus plexippus*	1.00E-41
-	Tag_1110 CATGTTGAGTCTGGCGACCGCGCCAT	27	18	6.852	17.988	9.15E-04	-1.39	no hit	-	-	-	-
-	Tag_1974 CATGCCCGGCTTCCCCGGTGGCGCGT	9	6	2.284	5.996	2.84E-02	-1.39	no hit	-	-	-	-
-	Tag_1979 CATGGCGGCCGATGACGTCACAATAG	9	6	2.284	5.996	2.84E-02	-1.39	no hit	-	-	-	-
-	Tag_1983 CATGGTTGACTGGTTCGGCGGCGCTG	9	6	2.284	5.996	2.84E-02	-1.39	no hit	-	-	-	-
-	Tag_1991 CATGTCAAAGCTGCGCCCTTGCTCCC	9	6	2.284	5.996	2.84E-02	-1.39	no hit	-	-	-	-
-	Tag_2008 CATGATCGCCCACCTGGCCAGCGACT	9	6	2.284	5.996	2.84E-02	-1.39	no hit	-	-	-	-
-	Tag_2013 CATGATCACGGGCGGGCGGAACTTCG	9	6	2.284	5.996	2.84E-02	-1.39	no hit	-	-	-	-
-	Tag_2053 CATGTGCGACGTTCTTTGTGACGTCT	9	6	2.284	5.996	2.84E-02	-1.39	no hit	-	-	-	-
Nucleic acid binding	Tag_2060 CATGCACAAGCTACGGCGCTGACGCG	9	6	2.284	5.996	2.84E-02	-1.39	Hv_Contig_240	4485	3622-3647	EHJ77800|putative Rho guanine nucleotide exchange factor 11 *Danaus plexippus*	0.00E+00
Unknown	Tag_858 CATGAGGCGAGCCCGGGATGGGTACC	42	28	10.658	27.982	6.01E-05	-1.39	Hv_Contig_29239	541	253-278	XP_014358180|BET1 homolog *Papilio machaon*	5.00E-54
-	Tag_1575 CATGCGCGCGCCGCACCACGACAACT	15	10	3.807	9.994	8.71E-03	-1.39	no hit	-	-	-	-
-	Tag_1273 CATGAAGATTGGCCGCATCGAGGATA	21	14	5.329	13.991	2.79E-03	-1.39	no hit	-	-	-	-
-	Tag_681 CATGATCTGAGTTCAAACCGGTGTGT	66	44	16.749	43.972	8.48E-07	-1.39	no hit	-	-	-	-
Translation/Ribosome biogenesis	Tag_305 CATGGGCTTCGGTCTGTTTGACTAAA	208	138	52.784	137.911	2.86E-17	-1.39	Hv_Contig_33232	456	331-356	ABS57437|ribosomal protein P2 *Heliconius melpomene*	4.00E-17
-	Tag_88 CATGCCCGGCTTCCCCGGCGGCGCGG	862	565	218.749	564.638	5.38E-63	-1.37	no hit	-	-	-	-
-	Tag_434 CATGCGCCTGTGCGGCGGCCTGGGCT	133	87	33.751	86.944	2.31E-11	-1.37	no hit	-	-	-	-
Translation/Ribosome biogenesis	Tag_626 CATGTTCGAGCTGCCGCGCCAGCGCG	78	51	19.794	50.967	2.08E-07	-1.36	Hv_Contig_19425	740	333-358	XP_011552505|eukaryotic translation initiation factor 3 subunit C-like *Plutella xylostella*	3.00E-122
signal transduction	Tag_417 CATGCGCACGGGGCAGGCCGTGCAGG	142	92	36.035	91.941	9.64E-12	-1.35	Hv_Contig_5376	1585	955-980	XP_013195998|guanine nucleotide-binding protein subunit beta-5 *Amyelois transitella*	0.00E+00
Protein folding/Recycling	Tag_282 CATGAAGGCGCTGGCCGAGAAGCTCA	227	147	57.605	146.906	1.34E-17	-1.35	Hv_Contig_8105	1275	984-1009	XP_013143717|26S proteasome non-ATPase regulatory subunit 13 *Papilio polytes*	0.00E+00
Primary metabolic process/hydrolase activity	Tag_482 CATGCACCCTAACTGGACTCCTGCAC	113	73	28.676	72.953	1.16E-09	-1.35	Hv_Contig_29753	531	415-440	ABR88238|chymotrypsin-like protease C8 *Heliothis virescens*	2.00E-106
-	Tag_1599 CATGAAACGCTTGTACGTCCAGTGGG	14	9	3.553	8.994	1.38E-02	-1.34	no hit	-	-	-	-
-	Tag_1612 CATGATCACGGGCGGGCGGAACTCGG	14	9	3.553	8.994	1.38E-02	-1.34	no hit	-	-	-	-
-	Tag_1629 CATGTGTTGCTGGGGAGTTTGTTGCG	14	9	3.553	8.994	1.38E-02	-1.34	no hit	-	-	-	-
Protein folding/Recycling	Tag_1640 CATGCTTTTCACTTCCACTGCATCTC	14	9	3.553	8.994	1.38E-02	-1.34	Hv_Contig_23173	640	335-360	NP_001040275|ring box protein *Bombyx mori*	3.00E-62
-	Tag_1156 CATGCTGCAGCACGTCACGCGCACGG	25	16	6.344	15.990	2.18E-03	-1.33	no hit	-	-	-	-
-	Tag_707 CATGTCGACTTGCCCGTTATCACAAG	61	39	15.480	38.975	6.61E-06	-1.33	no hit	-	-	-	-
Primary metabolic process/hydrolase activity	Tag_1818 CATGGCTAGCTCCGTCAAATATGATC	11	7	2.791	6.996	2.50E-02	-1.33	Hv_Contig_24782	611	396-421	XP_011556586|pancreatic lipase-related protein 2-like *Plutella xylostella*	6.00E-93

-	Tag_1820 CATGTCCGTGCGCTCCAGCGACTGTG	11	7	2.791	6.996	2.50E-02	-1.33	no hit	-	-	-	-
Primary metabolic process/hydrolase activity	Tag_1825 CATGAGTTCAGGGCCGGCCCAGCTAT	11	7	2.791	6.996	2.50E-02	-1.33	Hv_Contig_5687	1539	1199-1224	KPI91438|putative allantoinase 1 *Papilio xuthus*	0.00E+00
-	Tag_1879 CATGCTGGACCTGGCGCAGGTGGCCT	11	7	2.791	6.996	2.50E-02	-1.33	no hit	-	-	-	-
